# Regioselective Synthesis of Fluorescent Alkoxy-Substituted Phenazines and *N*‑Alkyl Phenazinium Salts

**DOI:** 10.1021/acs.joc.5c00923

**Published:** 2025-06-24

**Authors:** Paweł Ręka, Katarzyna Ostrowska, Jarosław Grolik, Olga Kotula, Grzegorz Urant, Maria Pikoń

**Affiliations:** † Department of Organic Chemistry, Faculty of Chemistry, 201871 Jagiellonian University, Gronostajowa 2, 30-387 Kraków, Poland; ‡ Doctoral School of Exact and Natural Sciences, Jagiellonian University in Kraków, Prof. S. Łojasiewicza 11, 30-348 Kraków, Poland

## Abstract

A method for the regioselective synthesis of 2,3,7,8-tetraalkoxyphenazines and *N*-alkyl phenazinium salts was developed. The protocol allows the synthesis of compounds with up to four different substituents at the designed positions. The synthesis was achieved using nonsymmetrically substituted 4,5-dialkoxy-2-nitroanilines and 1-bromo-2-nitrobenzenes via Buchwald–Hartwig amination, followed by tandem catalytic reduction and oxidative cyclization. The synthesized derivatives exhibit intense fluorescence in solution, with significant changes in spectroscopic properties induced by solvent polarity, the presence of acids, and changing the counterion in *N*-alkyl phenazinium salts.

## Graphical abstract

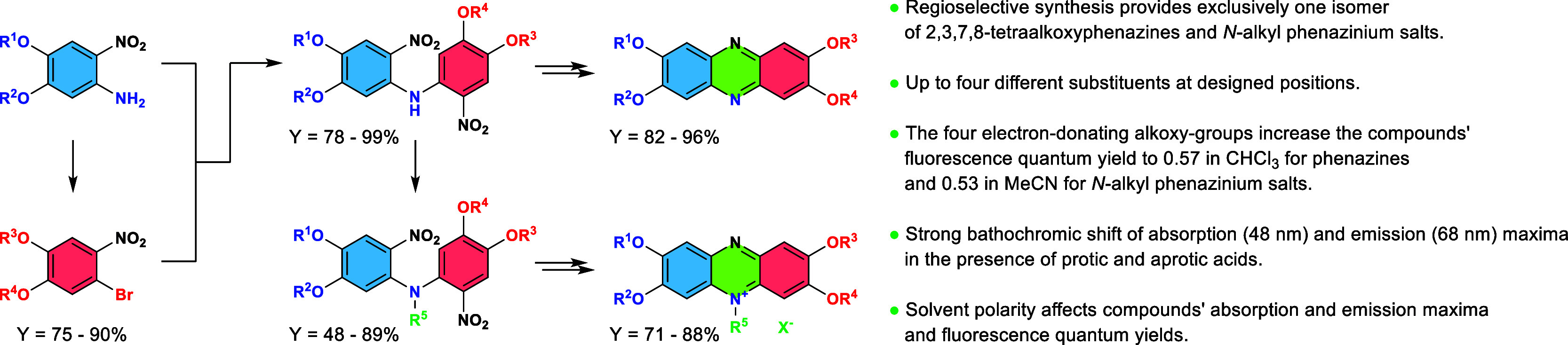

## Introduction

In the large family of phenazine-core compounds, there are many which exhibit insecticidal, antimicrobial,^1^ antifungal,^2^ antiviral,^3^ and antitumor^4^ activities. The application of phenazine derivatives, especially with an extended aromatic system, was investigated in the field of organic semiconductors^5^ and OLEDs.^6^ The *N*-substituted phenazinium salts have a strong affinity to the negatively charged nucleic acids, which can be applied for DNA photocleavage.^7^ The *N*-methyl phenazinium salt conjugate with pheophorbide was investigated as a fluorescent light-up probe capable of distinguishing between duplex and quadruplex polynucleotides.^8^ Despite biochemical applications, the *N*-alkylated phenazinium salts can also be used as redox photocatalysts.^9^ The synthetic 2,3-alkoxy derivatives of phenazine have been investigated as anticancer agents against pancreas cancer^10^ and castration-resistant prostate cancer.^11^ Our previous research also investigated the cytotoxicity of nonsymmetrically substituted 2,3-dialkoxy phenazines on the LoVo cell line (colorectal cancer).^12^ The 2,3-dialkoxyphenazine derivatives, especially with further extended aromatic systems, were developed as a promising dye for application in OLEDs.^13^ The 1,2,3,6,7,8-hexamethoxyphenazine and 1,4,6,9-tetramethoxyphenazine were patented for application in secondary batteries with enhanced energy densities.^14^

Traditional phenazine synthesis methods such as Wohl–Aue,^15^ Nietzki–Ernst,^15^ or Waterman–Vivian^15^ often produce a mixture of isomers and lower yields for alkoxy-substituted substrates.^15^ The most common synthetic strategy to approach alkoxy-phenazines is cyclocondensation of substituted *o*-phenylenediamines with *o*-quinones.^16^ This method uses alkoxy-substituted *o*-phenylenediamines^17^ and *o*-quinones^18^ or initially synthesizes a hydroxyphenazine derivative, followed by alkylation to obtain the target compound (Scheme 1a).^10^ This approach was used to synthesize cytotoxic 2,3-dialkoxyphenazines investigated as anticancer agents.^10^ A relatively novel method for synthesizing phenazines and their *N*-oxides is the reaction between benzoxadiazoles and diaryliodonium salts,^19^ where the inductive effect influences the ratio of the obtained isomers. Methods that provide the selectivity of nonsymmetrically substituted phenazines are based on Buchwald–Hartwig amination^20^ (Scheme 1b) and Ecker–Stainer cyclization (Scheme 1c).^21^ In our previous research,^12^ we presented synthetic protocols where nonsymmetrically substituted 2-nitroanilines (Scheme 1d) are coupled with 1-bromo-2-nitrobenzene derivatives in Buchwald–Hartwig amination. The obtained compound is then reduced to bis­(2-aminophenyl)­amine derivative and cyclized to phenazine (Scheme 1e) similarly as in the Ecker–Stiner method,^21^ but in the tandem scheme, i.e., without separation of bis­(2-aminophenyl)­amine intermediate. In this research, we developed an extension of the described synthetic protocol that allows the regioselective synthesis of 2,3,7,8-tetraalkoxyphenazines and *N*-alkyl phenazinium salts bearing up to four alkoxy groups at designed positions.

**Scheme 1:**
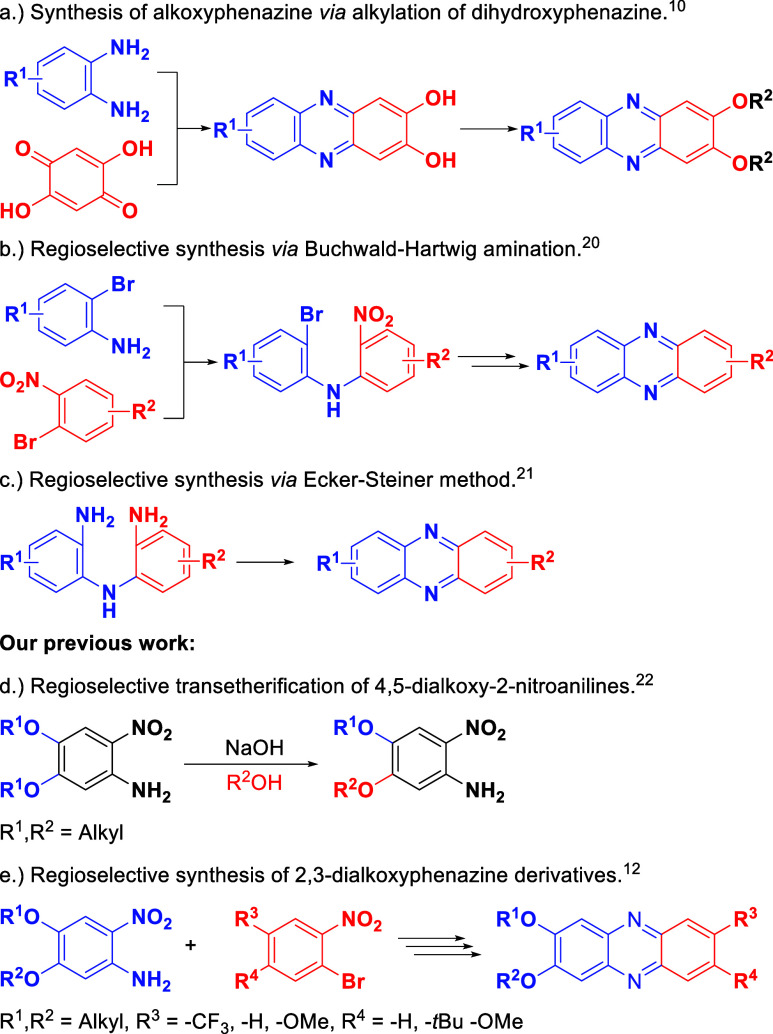
Synthesis of Phenazine Derivatives

## Results and Discussion

The starting substrate for the presented synthesis is 4,5-dialkoxy-2-nitroaniline, which can be substituted with two different alkoxy chains in designed positions. The synthetic protocol developed in our earlier research^22^ allows the substitution of an alkoxy-chain at position para regarding the nitro group (Scheme 1d) with the alkoxy group from alcohol used as the reaction solvent. The preparation of 2,3,7,8-tetraalkoxyphenazine was possible using 1-bromo-4,5-dialkoxy-2-nitrobenzenes, which were synthesized from adequate 2-nitroaniline derivatives by diazotization, yielding nonsymmetrically substituted compounds. The synthesis was performed using *tert*-butyl nitrite and copper­(II) bromide^23^ in organic solvent—acetonitrile (Scheme 2a). The reaction time was optimized for 1 h, and the reaction yields are up to 90% for the investigated compounds (all listed in Table 1). The attempts of diazotization with sodium nitrite in aqueous conditions did not yield the expected 1-bromo-2-nitrobenzene in preliminary experiments; therefore, the reaction in an organic solvent was chosen and further optimized.

**Scheme 2:**
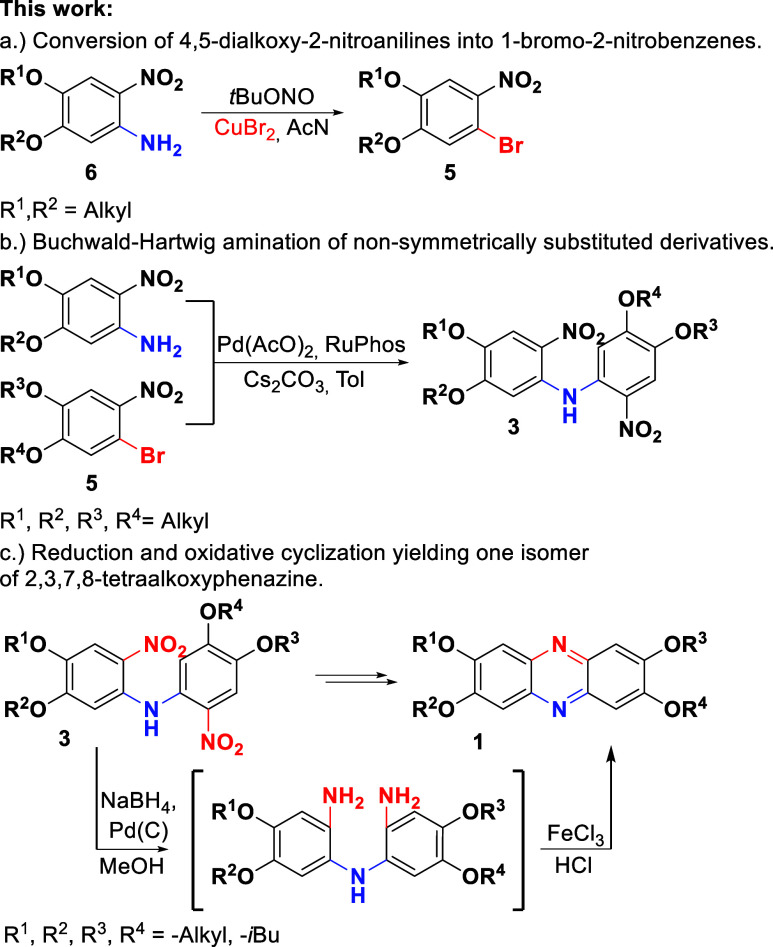
Regioselective Synthesis of 2,3,7,8-Tetraalkoxyphenazines

**Table 1:** Synthesis of 1-Bromo-4,5-dialkoxy-2-nitrobenzenes

entry	compound	R1	R2	yield [%]^a^
1	**5a**	–(CH_2_)_5_CH_3_	–(CH_2_)_5_CH_3_	85
2	**5b**	–(CH_2_)_5_CH_3_	–*i*Bu	87
3	**5c**	–CH_2_CH_3_	–*i*Bu	90
4	**5d**	–CH_2_CH_3_	–CH_3_	83
5	**5e**	–CH_3_	–(CH_2_)_10_OAc	75

a Reaction conditions: 4,5-dialkoxy-2-nitroaniline, CuBr_2_ (1.5 equiv), *tert*-butyl nitrite (2.2 equiv), MeCN, 1.5 h. Isolated yield.

The Buchwald–Hartwig coupling of 2-nitroaniline with 1-bromo-2-nitrobenzene yields exclusively one isomer of bis­(2-nitrophenyl)­amine, which serves directly as a substrate for the synthesis of phenazine. The reaction was performed in toluene using cesium carbonate as the base and palladium acetate and RuPhos as the catalyst for synthesis with all investigated compounds (Scheme 2b).

The reaction with alkoxy-substituted 1-bromo-2-nitrobenzenes was performed at 90 °C with the optimal reaction time of 90 min. Obtained compounds (**3a**–**3c**) were then used for phenazine synthesis (Scheme 2c), which gives 2,3,7,8-tetraalkoxy-substituted derivatives (Figure 1: **1a**–**1c**). The reduction of bis­(2-nitrophenyl)­amines and oxidation of the intermediate was performed in a tandem-like scheme.^12^ For the bis­(2-nitrophenyl)­amines not very soluble in hot methanol, ethyl acetate serves well as a cosolvent. The intermediate bis­(2-aminophenyl)­amine oxidation to phenazine was achieved using ferric chloride as a mild oxidant, providing a regioselective ring enclosure. Within the applied synthetic protocol, selection of substrates with intended substituents yields a designed product to be formed exclusively as a single isomer. The representative examples of this method’s utility are phenazines **1a** and **1b**, substituted with four different alkoxy chains at designed positions. Reaction yields of both synthesis steps are given in Table 2.

**Figure 1:**
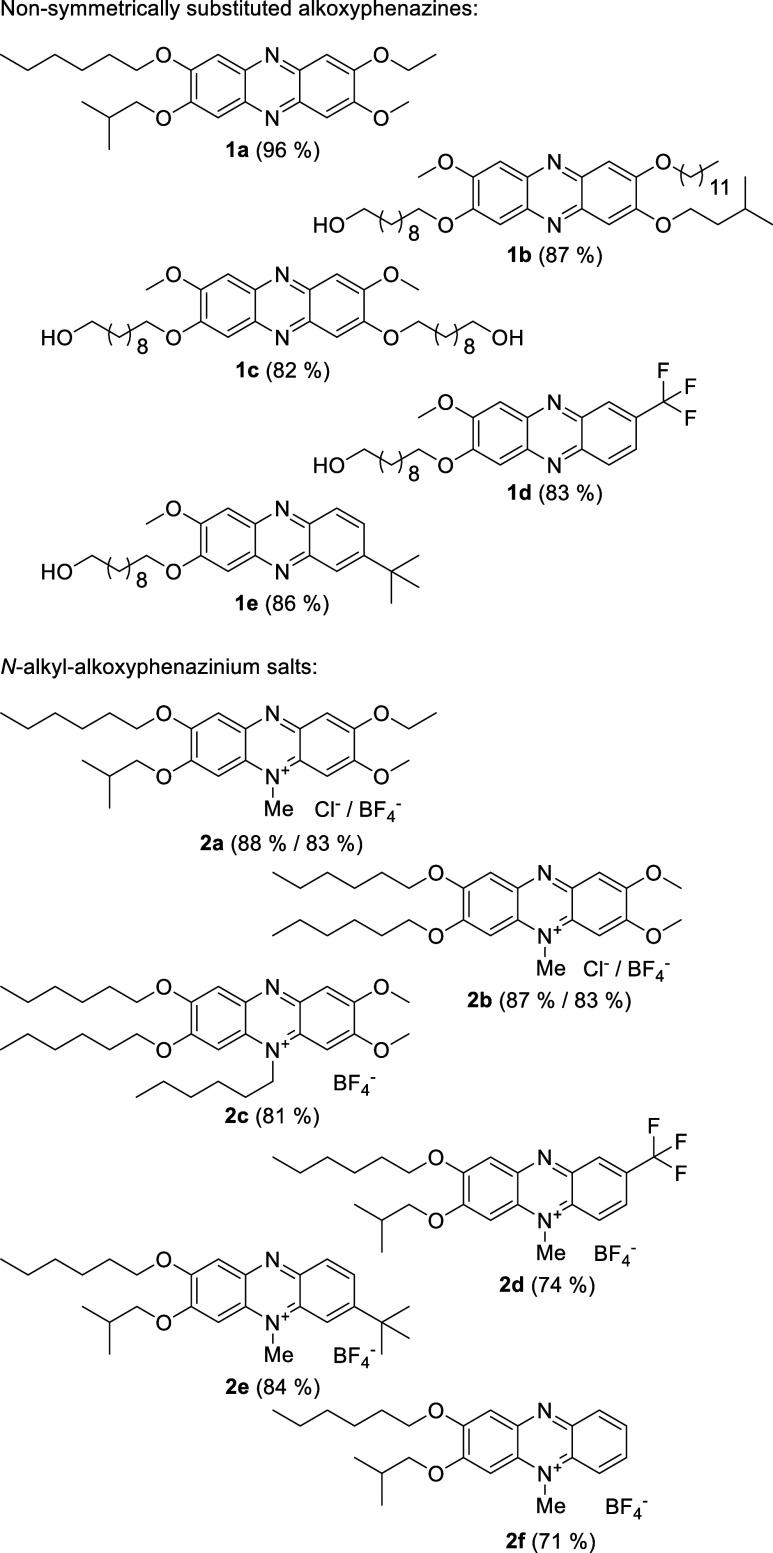
Synthesized phenazines and *N*-alkyl phenazinium salts.

**Table 2:** Reaction Yields of Phenazine Synthesis

bis(2-nitrophenyl)amine	yield [%]^a^ ^,^ ^b^	phenazine	yield [%]^c^
**3a**	98^a^/96^a^ ^,^ ^d^	**1a**	96/94^d^
**3b**	81^a^	**1b**	87
**3c**	84^a^	**1c**	82
**3d**	78^a^	**1d**	83
**3e**	99^a^	**1e**	86
**4a**	89^a^	**2a** Cl^–^/BF_4_^–^	88/83^e^
**4b**	83^b^/89^b^ ^,^ ^d^	**2b** Cl^–^/BF_4_^–^	85/87^d^/83^e^
**4c**	48^b^	**2c** BF_4_^–^	81
**4d**	83^b^	**2d** BF_4_^–^	74
**4e**	75^b^	**2e** BF_4_^–^	84
**4f**	79^b^	**2f** BF_4_^–^	71

a Reaction conditions: 4,5-dialkoxy-2-nitroaniline, 1-bromo-2-nitrobenzene derivative (1.05 equiv), Pd­(OAc)_2_ (0.05 equiv) RuPhos (0.075 equiv), Cs_2_CO_3_ (1.2 equiv), toluene, 90 °C, 1.5 h for **3a**–**3c**, 110 °C, 24 h for **3d**–**3e**. Isolated yield.

b Reaction conditions: bis­(2-nitrophenyl)­amine derivative, NaH (1.5 equiv), iodomethane (8 equiv) or iodohexane (8 equiv, only for **4c**), DMF, RT, 24 h. Isolated yield.

c Reaction conditions: **3a**–**3e** or **4a**–**4f**, palladium on carbon (10% Pd, 0.04 equiv), NaBH_4_ (added up to discoloration of mixture), MeOH, AcOEt, b.p., filtered through silica gel, then HCl (36%, ∼20 equiv), FeCl_3_ (water solution, 4 equiv), RT, 0.5 h. Isolated yield.

d Reaction on a gram scale.

e Yield including ion exchange of Cl^–^ to BF_4_^–^. Reaction conditions: *N*-alkyl phenazinium salt, NaBF_4_ (10 equiv), H_2_O, CHCl_3_, RT, 24 h.

*N*-Alkyl phenazinium salts are typically synthesized by alkylation of phenazine with alkyl halides or dialkyl sulfates,^15^ resulting in an isomeric mixture with regioisomeric ratios influenced by electronic effects and steric hindrance (Scheme 3a). The innovative synthesis of nonsymmetrically substituted compounds involves further derivatization of *N*-alkylated phenazinium salts, in the direct amination with primary and secondary amines.^24^ The *N*-substituted phenazinium salts can be obtained by condensing *o*-quinone with *N*-substituted 2-nitroanilines. The nonsymmetrically substituted 2-nitroanilines retain substituent locations in synthesis, as demonstrated for 9-diethylaminoalkylphenazines.^25^ Nevertheless, condensation of *o*-quinone, which encloses the phenazine aromatic system, is not restricted to one possibility.

**Scheme 3:**
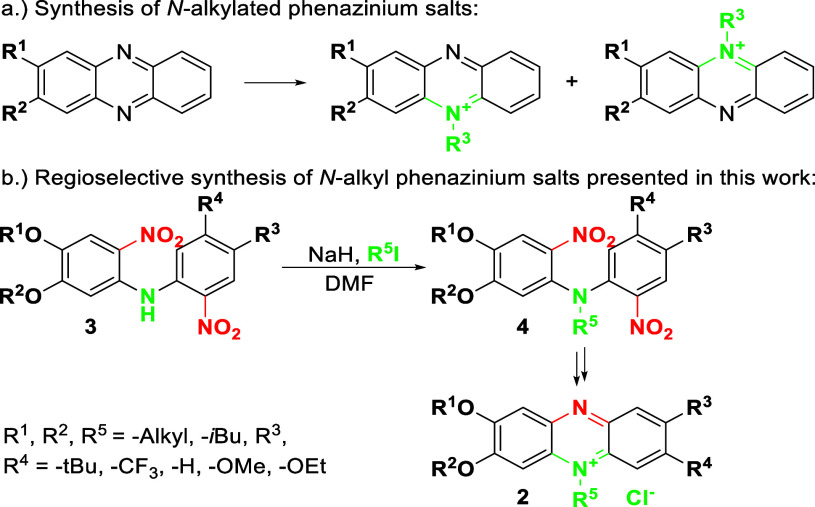
Regioselective Synthesis of *N*-Alkyl Phenazinium Salts

The synthetic protocol for *N*-alkyl phenazinium salts from *N*-alkyl-bis­(2-nitrophenyl)­amine investigated in our research is an approach that, to the best of our knowledge, has not been reported so far and provides reaction regioselectivity with high product yield. Alkylation of bis­(2-nitrophenyl)­amine, obtained by the Buchwald–Hartwig amination, was investigated in reaction with alkyl iodides: iodomethane and 1-iodohexane. Obtained compounds form *N*-alkyl phenazinium salts after reduction and oxidative cyclization (Scheme 3b) with good yields (Table 2) under the same conditions as for the described phenazines. The product is isolated as the chloride from the reaction mixture, which originates from hydrochloric acid and ferric chloride used in the synthesis. The exchange of the counterion for tetrafluoroborate was accomplished by overnight stirring of a chloroform solution of the *N*-alkyl phenazinium salt with a water solution of sodium tetrafluoroborate. The impact of chloride and tetrafluoroborate counterions on the spectroscopic properties of compounds **2a** and **2b** was investigated; all other phenazinium salts (**2c**–**2f**) were prepared as tetrafluoroborates.

The *N*-alkyl substituent position in phenazinium salt depends solely on the substrate’s molecular structure, which was confirmed by detailed NMR analysis. The described protocol applies well to 2,3,7,8-tetraalkoxy substituted compounds (Figure 1: **2a**–**2c**), for both the *N*-methyl (**2b**) and *N*-hexyl substituent (**2c**). The regioselective reaction yields a single isomer, as evidenced in the example of a derivative substituted with four different alkoxy chains (**2a**). The reaction versatility was proved by the synthesis of phenazinium salts with a bulky alkyl substituent (**2e**), an electron-withdrawing trifluoromethyl group (**2d**), and a nonsubstituted benzene ring (**2f**) as examples. Details of the experiments and compound characterization are contained in the Supporting Information.

The synthetic protocol for phenazines substituted with a 10-hydroxydecyloxy group was described with four examples (**1b**–**1e**). The synthesis of those derivatives was achieved by using 4,5-dialkoxy-2-nitroanilines (**6a**–**6c**) obtained in a modified version of the transetherification protocol.^22^ For this synthesis, we used solid-state 1,10-decanediol, which melts at the reaction temperature and can be crystallized from chloroform and reused in subsequent syntheses. Details of the synthetic protocol and compound characterization are contained in the Supporting Information. The rest of the synthetic protocol, i.e., Buchwald–Hartwig amination and reduction-cyclization, can be applied for this synthesis without significant modification.

The 2,3,7,8-tetraalkoxyphenazines and *N*-alkyl phenazinium salts, synthesized using the described protocol, exhibit intense fluorescence with emission maxima at approximately 410 and 490 nm, respectively. The 2,3-dialkoxyphenazines and phenazinium salts were used for comparison to demonstrate the effect of substituents on spectroscopic properties. The influence of solvent on the spectroscopic properties in acetonitrile and chloroform was investigated for all compounds. For phenazine **1a**, the qualitative effect of different acid presence was examined, and fluorescence titration was performed. From the examined phenazines **1a**–**1e**, the 2,3,7,8-tetraalkoxy-substituted compounds (**1a**–**1c**) have high fluorescent quantum yields (Table 3), where the highest value is Φ = 0.57 for **1b** in CHCl_3_. In comparison, 2,3-dialkoxy compounds bearing *tert*-butyl or trifluoromethyl groups have Φ < 0.01 in both solvents. For all investigated phenazines, fluorescence quantum yield is higher in chloroform than in acetonitrile. Both absorption and emission maxima are bathochromically shifted for the compound dissolved in chloroform compared to acetonitrile. The shifts are 4 and 6 nm, respectively, for **1a** (Chart 1). The increase of the Stokes shift for the **1a** solution in chloroform results in the increase of the fluorescence quantum yield from 0.28 to 0.48 (Table 3). Details of spectroscopic measurements and all spectra are contained in the Supporting Information. For the investigated *N*-alkyl phenazinium salts, there is a significant difference in fluorescence quantum yields depending on counterions in different solvents, which might find application in anion recognition. The **2a** and **2b** chlorides have high Φ values in acetonitrile (Table 3); however, the values are 7 times lower in chloroform. For tetrafluoroborates, fluorescence quantum yields are only ∼15% lower in chloroform than in acetonitrile. Similarly, for phenazines, the *N*-alkyl phenazinium salts have weak fluorescence if the compound is substituted with only two electron-donating alkoxy groups (**2d**–**2f**). The highest value of Φ = 0.53 was determined for **2a** tetrafluoroborate in acetonitrile. The absorption maximum of **2a** tetrafluoroborate in chloroform is bathochromically shifted by 8 nm regarding solution in acetonitrile (Chart 1). The emission maximum is shifted by <1 nm, resulting in the decrease of Stokes shift in chloroform, which corresponds to the lower fluorescence quantum yield of **2a** tetrafluoroborate in chloroform (Table 3). This effect can be observed for all investigated *N*-alkyl phenazinium salts; however, not all of them results in the decrease of fluorescence quantum yield (Table 3: **2c**, **2d**, and **2f**). For these compounds, the effect of solvation seems to have a stronger impact on fluorescence quantum yield as it explains the significant difference observed for investigated **2a** and **2b** chlorides, better solvated in polar acetonitrile, and the behavior of bearing a long alkyl chain, **2c** tetrafluoroborate, better solvated in nonpolar chloroform.

**Table 3:** Fluorescence Quantum Yields (Φ) of the Investigated Compounds in Acetonitrile and Chloroform

phenazine	Φ^a^ in MeCN	Φ^a^ in CHCl_3_	phenazinium salt	Φ^b^ in MeCN	Φ^b^ in CHCl_3_
**1a**	0.28^c^	0.48	**2a** Cl^–^	0.42	0.06
**1b**	0.15	0.57	**2a** BF_4_^–^	0.53	0.46
**1c**	0.12	0.49	**2b** Cl^–^	0.42	0.06
**1d**	0.002	0.003	**2b** BF_4_^–^	0.53	0.45
**1e**	0.003	0.008	**2c** BF_4_^–^	0.26	0.43
			**2d** BF_4_^–^	0.008^d^	0.013
			**2e** BF_4_^–^	0.011^e^	0.004
			**2f** BF_4_^–^	0.025	0.034

a Fluorescence quantum yield was determined using a 375 nm excitation wavelength and 9,10-diphenylanthracene (DPA) as the reference standard unless stated otherwise.

b Fluorescence quantum yield was determined using a 430 nm excitation wavelength and 9,10-bis­(phenylethynyl)­anthracene (BPEA) as the reference standard unless stated otherwise.

c 380 nm excitation wavelength was used.

d 370 nm excitation wavelength was used with DPA as the reference standard.

e 375 nm excitation wavelength was used with DPA as the reference standard.

**Chart 1:**
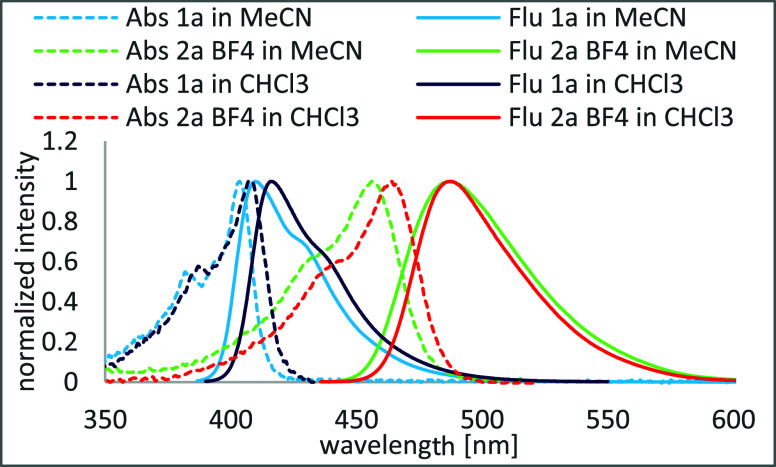
Comparison of Absorption and Emission Spectra of **1a** and **2a** BF_4_ in Acetonitrile (MeCN) and Chloroform (CHCl_3_)

The effect of the presence of different acids was also investigated for a representative example of phenazine **1a**. The absorption and emission spectrum of **1a** in acetonitrile is shifted bathochromically by the influence of methanesulfonic acid (CH_3_SO_2_OH) by 38 and 68 nm, respectively, which increases the Stokes shift from about 6 to 36 nm and fluorescence quantum yield from Φ = 0.28 to Φ = 0.36. Fluorescence titration of **1a** with methanesulfonic acid was performed in acetonitrile (Chart S1) since **1a** is insoluble in water. Equilibrium was observed at a strongly acidic, apparent pH of about 1.7. The qualitative effect of the addition of 10 equiv of acids shows that strong Brønsted and Lewis acids cause a bathochromic shift of the absorption and emission spectra (Table S1). The results show that in general, both strong protic and aprotic acids shift fluorescent spectrum maxima bathochromically over 70 nm and absorption maxima 38–48 nm. In concentrated strong acid, absorption maxima shift beyond 500 nm, causing fluorescence extinction, as seen in samples with 100 equiv BF_3_ (Table S1), similar to the effects observed in the methanesulfonic acid solution of **1a** in preliminary experiments.

## Conclusions

In summary, we presented a synthetic protocol that allows the regioselective synthesis of 2,3,7,8-tetraalkoxyphenazines and *N*-alkyl-2,3-dialkoxyphenazinium salts with high total yields of up to 95% and 78%, respectively. We also included a synthetic method for required substrates: nonsymmetrically substituted 4,5-dialkoxy-1-bromo-2-nitrobenzenes and 4,5-dialkoxy-2-nitroanilines bearing 10-hydroxydecyloxy groups. The molecular structure of all compounds was characterized and carefully analyzed with ^1^H, ^13^C, and, if needed, ^19^F NMR spectroscopy, with additional information from gCOSY, gHSQC, and gHMBC experiments. The obtained compounds have intense fluorescence with a fluorescence quantum yield in solution of up to 0.57 and 0.53 for phenazines and *N*-alkyl phenazinium salts, respectively. The influence of solvent polarity and acid presence was shown in several representative examples. The presented method allows the synthesis of a broad scope of compounds that remain regioselective and efficient. The interesting spectroscopic properties of the obtained compounds suggest their potential applications in the optical sensing of protic and aprotic acids and anion recognition. The high fluorescence quantum yield suggests a potential application in organic light-emitting materials, which we might explore in future research. The synthetic protocol described in this article opens the route for new types of compounds with possible biological activity, which is currently under our investigation.

## Supplementary Material



## Data Availability

The data underlying this study are available in the published article and its Supporting Information.

## Notes

General information, methods, and used equipment; all details of synthetic procedures; characterization of obtained compounds; and NMR, HRMS, and UV–vis spectra (PDF)
