# Supplementary material for: Regioselective Synthesis of Fluorescent Alkoxy-Substituted Phenazines and N‑Alkyl Phenazinium Salts
Source: J Org Chem. 2025 Jun 24;90(26):9118–23. doi: 10.1021/acs.joc.5c00923 (PMC12235614; doi:10.1021/acs.joc.5c00923)
Supplement: Supplementary file 1 [file jo5c00923_si_001.pdf]

Supporting Information

Regioselective Synthesis of Fluorescent Alkoxy-Substituted Phenazines and *N*-alkyl Phenazinium Salts

Paweł Ręka,[a,b] Katarzyna Ostrowska,[a] Jarosław Grolik,*,[a] Olga Kotula,[a] Grzegorz Urant[a] and Maria Pikoń[a]

* jaroslaw.grolik@uj.edu.pl

a – Department of Organic Chemistry, Faculty of Chemistry, Jagiellonian University, 30-387 Kraków, Gronostajowa 2, Poland;

b – Doctoral School of Exact and Natural Sciences, Jagiellonian University in Kraków, Prof. S. Łojasiewicza 11, 30-348 Kraków, Poland.

List of contents:

General information S3
Starting materials S4
Synthesis of phenazines (1a – 1e) and *N*-alkyl phenazinium salts (2a – 2f) S4
Characterisation, ^{1}H NMR, ^{13}C{^{1}H} NMR, HRMS and UV-Vis spectra of phenazines:
2-ethoxy-8-(hexyloxy)-7-isobutoxy-3-methoxyphenazine (**1a**) S5
Chart S1. Fluorescence titration of **1a** solution in acetonitrile S9
Table S1. The qualitative effect of acid addition on 1a spectra in acetonitrile S11
10-((7-(dodecyloxy)-8-(isopentyloxy)-3-methoxyphenazin-2-yl)oxy)decan-1-ol (**1b**) S11
10,10'-((3,7-dimethoxyphenazine-2,8-diyl)bis(oxy))bis(decan-1-ol) (**1c**) S15
10-((3-methoxy-7-(trifluoromethyl)phenazin-2-yl)oxy)decan-1-ol (**1d**) S19
10-((8-(*tert*-butyl)-3-methoxyphenazin-2-yl)oxy)decan-1-ol (**1e**) S23
Exchange of chloride counter ion for tetrafluoroborate in phenazinium salts (2a – 2f) **S27**
Characterisation, ^{1}H NMR, ^{13}C{^{1}H} NMR, HRMS and UV-Vis spectra of *N*-alkyl phenazinium salts:
2-ethoxy-8-(hexyloxy)-7-isobutoxy-3-methoxy-5-methylphenazin-5-ium chloride (**2a Cl^{-}**) S27
2-ethoxy-8-(hexyloxy)-7-isobutoxy-3-methoxy-5-methylphenazin-5-ium tetrafluoroborate (**2a BF$_4^-$**) S30
2,3-bis(hexyloxy)-7,8-dimethoxy-5-methylphenazin-5-ium chloride (**2b Cl^{-}**) S33
2,3-bis(hexyloxy)-7,8-dimethoxy-5-methylphenazin-5-ium tetrafluoroborate (**2b BF$_4^-$**) S37
5-hexyl-2,3-bis(hexyloxy)-7,8-dimethoxyphenazin-5-ium tetrafluoroborate (**2c**) S40
2-(hexyloxy)-3-isobutoxy-5-methyl-8-(trifluoromethyl)phenazin-5-ium tetrafluoroborate (**2d**) ...S43
7-(*tert*-butyl)-2-(hexyloxy)-3-isobutoxy-5-methylphenazin-5-ium tetrafluoroborate (**2e**) S47
2,3-bis(hexyloxy)-5-methylphenazin-5-ium tetrafluoroborate (**2f**) S50
Buchwald Hartwig coupling of 2-nitroanilines with 1-bromo-2-nitrobenzene derivatives (3a – 3e) .S53
Characterisation, ^{1}H NMR, ^{13}C{^{1}H} NMR and HRMS spectra of bis(2-nitrophenyl)amine derivatives:
4-ethoxy-*N*-(4-(hexyloxy)-5-isobutoxy-2-nitrophenyl)-5-methoxy-2-nitroaniline (**3a**) S53
10-(5-((4-(dodecyloxy)-5-(isopentyloxy)-2-nitrophenyl)amino)-2-methoxy-4-nitrophenoxy)decyl acetate (**3b**) S56
((azanediylbis(6-methoxy-4-nitro-3,1-phenylene))bis(oxy))bis(decane-10,1-diyl) diacetate (**3c**) ...S59
10-(2-methoxy-4-nitro-5-((2-nitro-4-(trifluoromethyl)phenyl)amino)phenoxy)decyl acetate (**3d**) S61
10-(5-((5-(*tert*-butyl)-2-nitrophenyl)amino)-2-methoxy-4-nitrophenoxy)decyl acetate (**3e**) S63
Synthesis, characterisation, ^{1}H NMR, ^{13}C{^{1}H} NMR and HRMS spectra of 4,5-bis(hexyloxy)-2-nitro-*N*-(2-nitrophenyl)aniline (3f) **S66**
***N*-alkylation of bis(2-nitrophenyl)amine derivatives (4a – 4f):** **S68**
Characterisation, ^{1}H NMR, ^{13}C{^{1}H} NMR and HRMS spectra of *N*-alkyl-bis(2-nitrophenyl)amine derivatives:
4-ethoxy-*N*-(4-(hexyloxy)-5-isobutoxy-2-nitrophenyl)-5-methoxy-*N*-methyl-2-nitroaniline (**4a**) ...S69
N-(4,5-bis(hexyloxy)-2-nitrophenyl)-4,5-dimethoxy-*N*-methyl-2-nitroaniline (**4b**) S71
N-(4,5-bis(hexyloxy)-2-nitrophenyl)-*N*-hexyl-4,5-dimethoxy-2-nitroaniline (**4c**) S73
4-(hexyloxy)-5-isobutoxy-*N*-methyl-2-nitro-*N*-(2-nitro-4-(trifluoromethyl)phenyl)aniline (**4d**) S75
N-(5-(*tert*-butyl)-2-nitrophenyl)-4-(hexyloxy)-5-isobutoxy-*N*-methyl-2-nitroaniline (**4e**) S78
4,5-bis(hexyloxy)-*N*-methyl-2-nitro-*N*-(2-nitrophenyl)aniline (**4f**) S81
Synthesis 1-bromo-4,5-dialkoxy-2-nitrobenzene derivatives (5a – 5e) **S83**
Characterisation, ^{1}H NMR, ^{13}C{^{1}H} NMR and HRMS spectra of 1-bromo-4,5-dialkoxy-2-nitrobenzene derivatives:
1-bromo-4,5-bis(hexyloxy)-2-nitrobenzene (**5a**) S84
1-bromo-4-(hexyloxy)-5-isobutoxy-2-nitrobenzene (**5b**) S86

1-bromo-4-ethoxy-5-isobutoxy-2-nitrobenzene (**5c**) S88
1-bromo-4-ethoxy-5-methoxy-2-nitrobenzene (**5d**) S90
10-(5-bromo-2-methoxy-4-nitrophenoxy)decyl acetate (**5e**) S92
Synthesis, characterisation, ^{1}H NMR, ^{13}C{^{1}H} NMR and HRMS spectra of 10-(5-amino-2-methoxy-4-nitrophenoxy)decan-1-ol (6a) S94
Synthesis, characterisation, ^{1}H NMR, ^{13}C{^{1}H} NMR and HRMS spectra of 10-(5-amino-2-methoxy-4-nitrophenoxy)decyl acetate (6b) S96
Synthesis, characterisation, ^{1}H NMR, ^{13}C{^{1}H} NMR and HRMS spectra of 4-(dodecyloxy)-5-(isopentyloxy)-2-nitroaniline (6c) S98
References S101

General information.

Structural assignments were made using additional information from gHSQC and gHMBC experiments for selected compounds. The NMR spectra were collected on: Bruker AVANCE III 300 MHz, JEOL JNM-ECZ400S 400 MHz, and JEOL JNM-ECZ600R 600 MHz spectrometers. Melting points were measured on a polarised light microscope, an Axioscope A1 Pol with a thermostatic interface - LINKAM LTSE420. The HRMS data were determined on a Bruker Daltonics micrOTOF-Q II spectrometer. All the UV-Vis spectra were recorded on a Hitachi U-3900H spectrophotometer with tungsten and deuterium lamps, and all of the fluorescence spectra were recorded on a Hitachi F-7000 fluorometer with a xenon lamp. The apparent pH of compounds acetonitrile solutions for spectroscopic measurements was measured using an ELMETRON pH-meter CP-411 with an ELMETRON EPS-1 probe.

Samples for melting point were prepared by evaporation of the solvent (DCM) and drying under vacuum.

All the NMR spectra were collected in 5mm diameter, borosilicate glass cuvettes suitable for the used spectrometer. The compounds' solutions for the measurements were prepared in $CDCl_3$ or DMSO-d_6. All chemical shifts (δ) were reported in parts per million (ppm) regarding tetramethylsilane (TMS, δ = 0 ppm for ^{1}H and ^{13}C NMR). For the ^{1}H and ^{13}C NMR spectra, the residual solvent signal was used as the internal chemical shift standard for $CDCl_3$ solutions: 7.26 and 77.16, respectively, and for DMSO-d_6 solutions: 2.50 and 39.52, respectively.

The spectroscopic measurements were conducted in the quartz cuvettes with an optical path length of 10 mm. The concentration of the compound solutions was adjusted to keep the absorbance around 0.1 at the excitation wavelength. For the fluorescence measurement, the excitation slit was set to 2.5 nm, the emission slit to 5 nm and the photomultiplier voltage was set to 600 V. Fluorescence quantum yield (Φ) in solution was determined using the relative method according to literature protocol[1] and calculated according to formula bellow:

$$\Phi = \Phi_R \times \frac{Int}{Int_R} \times \frac{1 - 10^{-Abs_R}}{1 - 10^{-Abs}} \times \frac{n^2}{{n_R}^2}$$

Where: Φ – is fluorescence quantum yield, Int – is an integration of the area under emission peak, Abs – is an absorbance at the excitation wavelength, n – is the refractive index of the solvent and R – corresponds to the value for reference compound solution.

The refractive indexes are taken from literature[2] and listed as follows: n_{CHCl3} = 1.443, n_{MeCN} = 1.344, n_{cHex} = 1.426. Where $CHCl_3$ is chloroform, MeCN is acetonitrile, and cHex is cyclohexane.

The literature fluorescence quantum yields of the reference compounds were taken from the PhotochemCAD™ database[3] and are listed as follows: DPA (9,10-diphenylanthracene) Φ = 1.00, and BPEA (9,10-bis(phenylethynyl)anthracene) Φ = 1.00, both measured in cyclohexane.

Fluorescence titration of a **1a** solution in acetonitrile was performed by measuring solution emission spectra (with excitation wavelengths 380 and 430 nm) at apparent pH, which was measured with a pH meter and adjusted by the addition of diluted methanesulfonic acid (MsOH).

Starting materials.

Substrates for the synthesis of **3a** and **5b**–**5d** were obtained as described in the literature.[4] Substrates for the synthesis of **4a**–**4e** were obtained according to the previously reported protocol.[5] Toluene for the synthesis of **3a**–**3e** was dried over sodium, distilled and stored over 4Å molecular sieves before use. DMF (dimethylformamide) for the synthesis of **3f** and **4a**–**4e** was collected directly from the Innovative Technologies PureSolv™ Micro solvent purification system. All the remaining solvents and reagents were used as obtained from suppliers. For all of the chromatography, SILICYCLE SilicaFlash P60 silica gel (particle size 40-63 µm, 230-400 mesh) was used.

A general protocol for the synthesis of phenazine derivatives (1a-1e and 2a-2f).

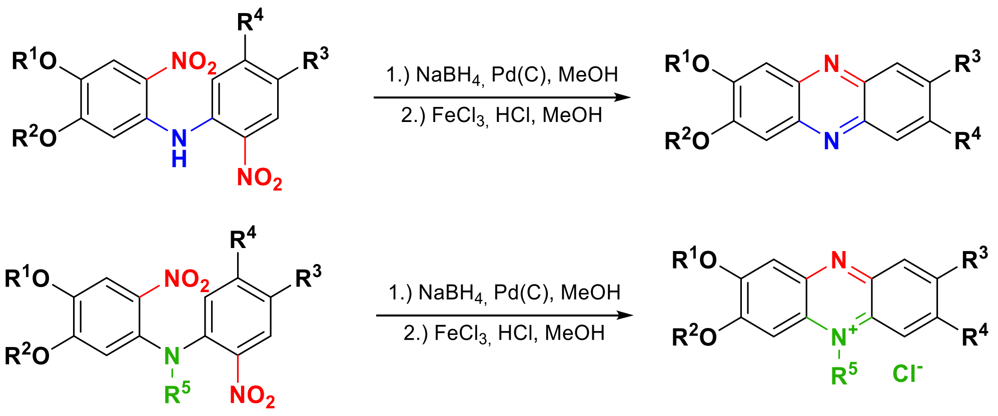

R^1,R^2 = Alkyl, R^3 = -OAlkyl, -CF_3, -H, R^4 = -OAlkyl, -H, -*t*Bu

In the round-bottom flask (100 mL of volume), substrate (0.40 mmol, **3a-3e** or **4a-4f**), palladium on carbon catalyst (10% Pd, 15 mg), and methanol (30 mL) were placed. The mixture was heated on an aluminium heating block to gentle boiling, and in the case of low-soluble compounds, an additional 30 mL of ethyl acetate was added. To the boiling mixture, sodium borohydride was added in small portions until the solution became completely colourless. Then the mixture was filtrated through a short pad of silica gel directly into a flask containing hydrochloric acid (1 mL, 36%). A solution of ferric chloride hexahydrate (440 mg, 4 eq.) in water (3 mL) was then added to the mixture. After stirring for 30 minutes mixture was diluted with DCM (50 mL) and extracted with water (400 mL). The water phase was then extracted with DCM (2x 30 mL). The combined organic phases were extracted with brine and dried over anhydrous magnesium sulphate. The solvent was removed under reduced pressure on the rotary evaporator and the crude product was purified by gradient elution column chromatography (silica gel, DCM / MeOH: 0.5-4% for **1a-1e**, 2-10% for **2a-2f**).

For the gram scale reaction (**1a** and **2b**) reagents were scaled proportionally, and the reaction was performed in a 250 mL round bottom flask using 75 mL of methanol and 75 mL of ethyl acetate. The reaction was diluted with 100 mL of DCM instead of 50 mL. The rest of the protocol remains the same.

2-ethoxy-8-(hexyloxy)-7-isobutoxy-3-methoxyphenazine (1a)

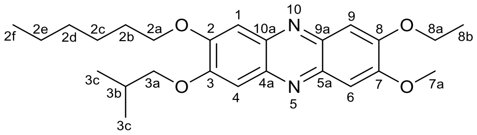

Compound synthesised from **3a**. Received pale yellow solid. M.p. = 119.2–121.7 °C. Yield = 164 mg (96 %). Yield = 802 mg (94 %, gram scale reaction performed on 1.01 g of the substrate).

^{1}H NMR (300 MHz, Chloroform-*d*) δ: 7.29 (s, 1H, H_1), 7.28 (s, 1H, H_9), 7.25 (s, 1H, H_4), 7.25 (s, 1H, H_6), 4.27 (q, J = 7.0 Hz, 2H, H_{8a}), 4.15 (t, J = 6.5 Hz, 2H, H_{2a}), 4.03 (s, 3H, H_{7a}), 3.92 (d, J = 6.6 Hz, 2H, H_{3a}), 2.32 – 2.17 (m, 1H, H_{3b}), 1.98 – 1.82 (m, 2H, H_{2b}), 1.55 (t, J = 7.0 Hz, 3H, H_{8b}), 1.61 – 1.47 (m, 2H, H_{2c}), 1.42 – 1.18 (m, 4H, $H_{2d,2e}$), 1.07 (d, J = 6.7 Hz, 6H, H_{3c}), 0.88 (d, J = 7.2 Hz, 3H, H_{2f}).

^{13}C{^{1}H} NMR (75 MHz, Chloroform-*d*) δ: 153.24 (C_8), 153.20 (C_7), 153.19 (C_3), 152.3 (C_2), 139.9 (C_{9a}), 139.8 (C_{5a}), 139.7 (C_{10a}), 139.5 (C_{4a}), 105.92 (C_4), 105.85 (C_1), 105.8 (C_6), 105.4 (C_9), 75.2 (C_{3a}), 69.0 (C_{2a}), 64.7 (C_{8a}), 56.3 (C_{7a}), 31.6 (C_{2d}), 28.9 (C_{2b}), 28.1 (C_{3b}), 25.8 (C_{2c}), 22.7 (C_{2e}), 19.3 (C_{3c}), 14.5 (C_{8b}), 14.1 (C_{2f}).

HRMS (ESI) *m/z* Calculated for $C_{25}H_{35}N_2O_4$ [M+H]$^+$: 427.2591, found: 427.2588.

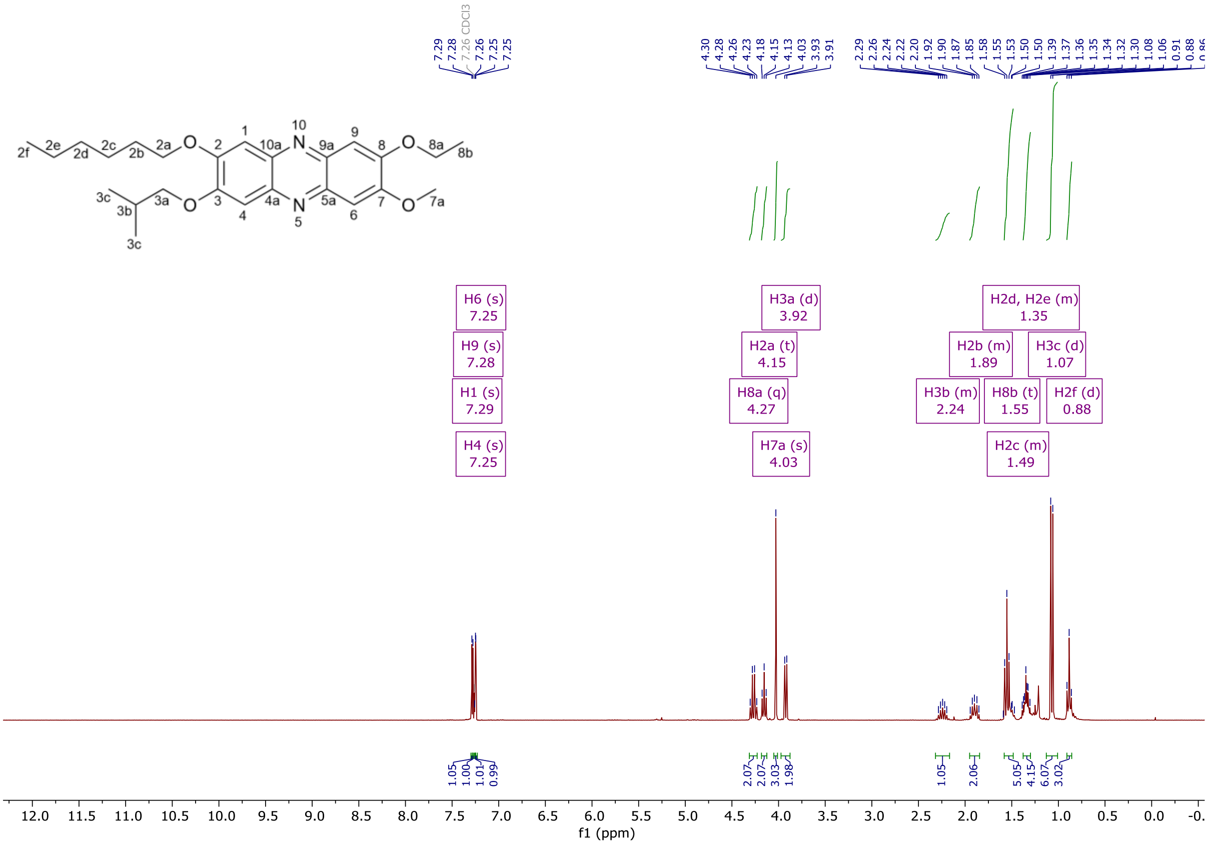

^{1}H NMR (300 MHz, Chloroform-*d*) spectrum of **1a**.

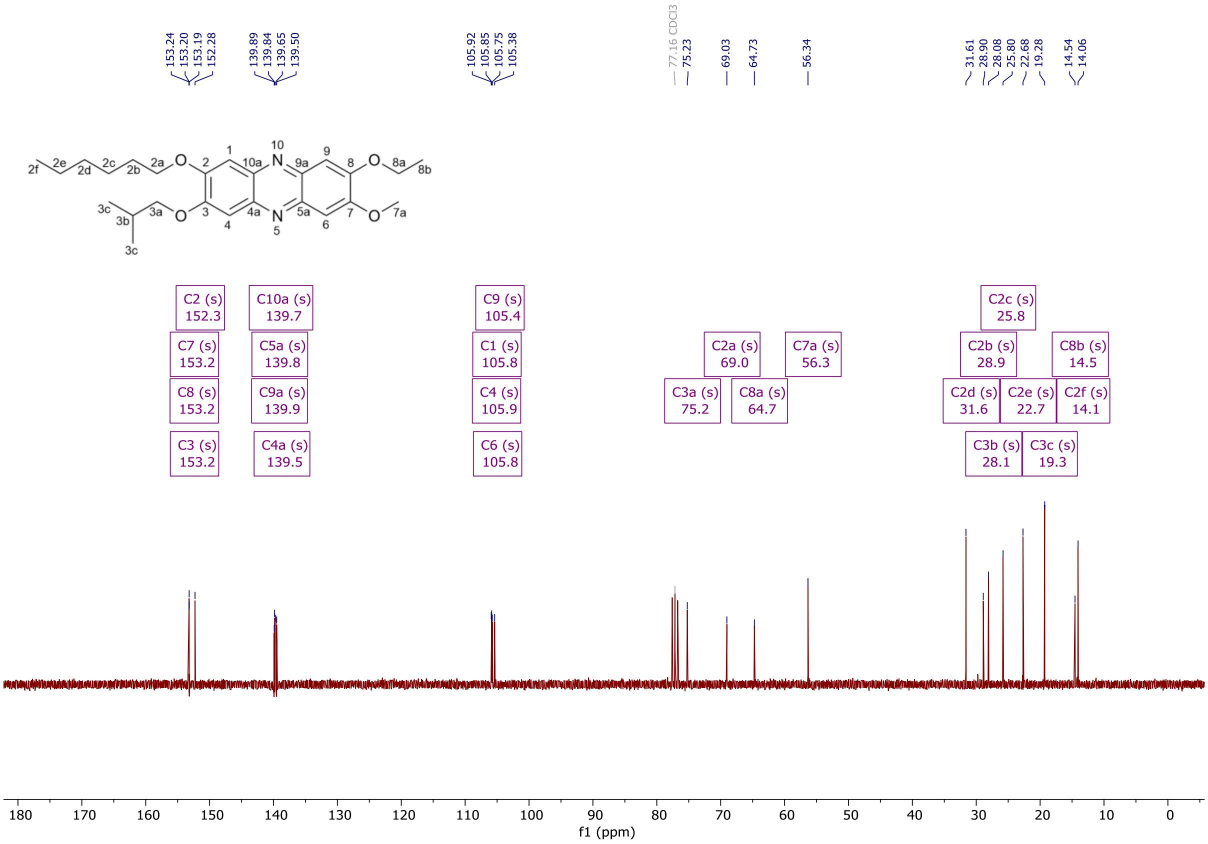

$^{13}C\{^{1}H\}$ NMR (75 MHz, Chloroform-*d*) spectrum of **1a**.

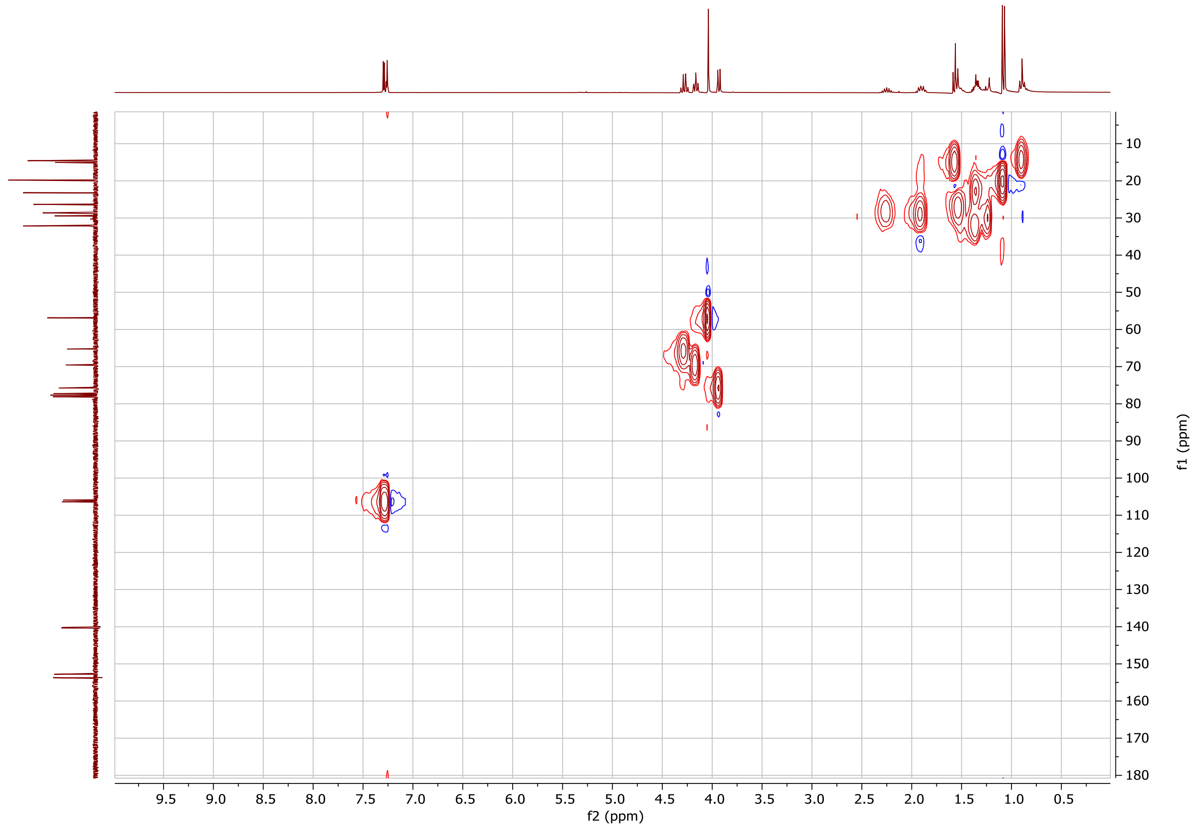

g-HSQC NMR (Chloroform-*d*) spectrum of **1a**.

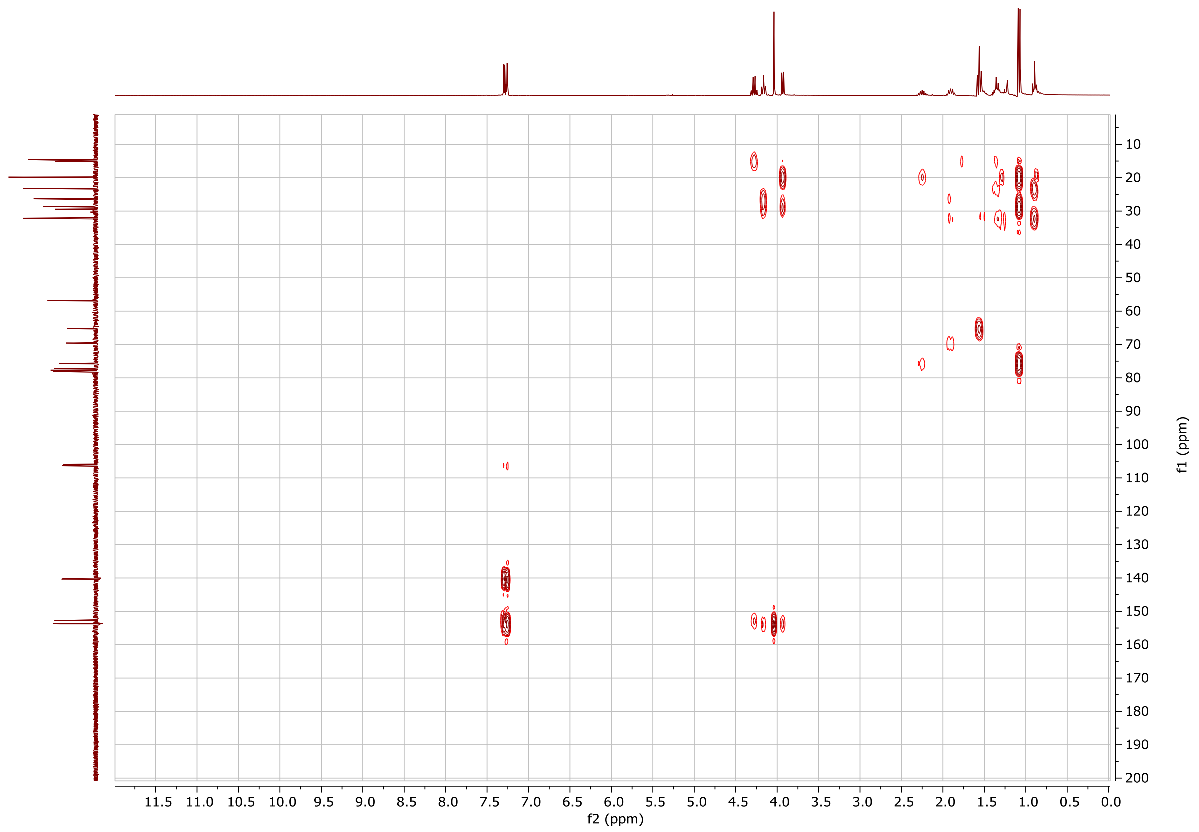

g-HMBC NMR (Chloroform-*d*) spectrum of **1a**.

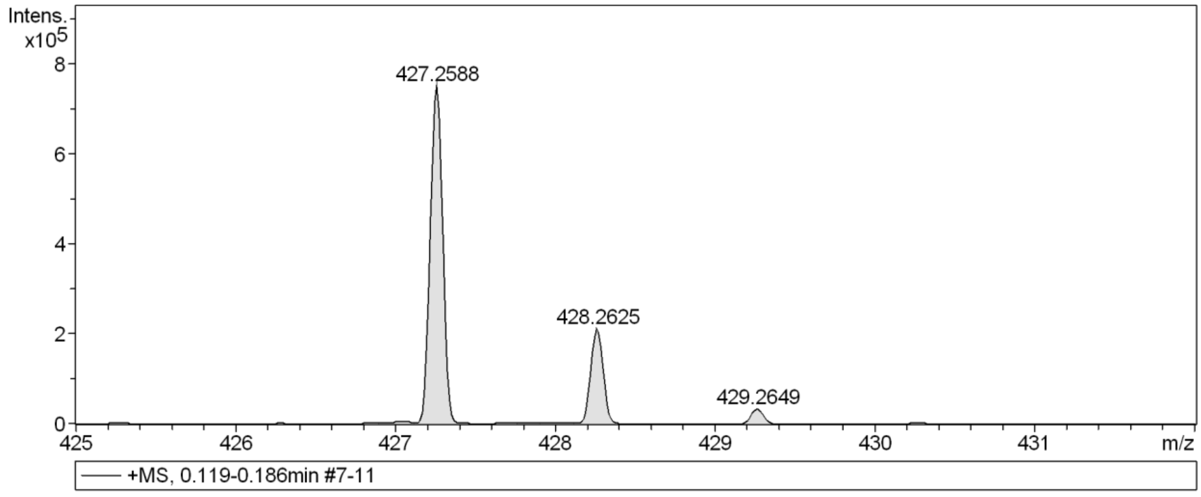

HRMS (ESI+) spectrum of **1a**.

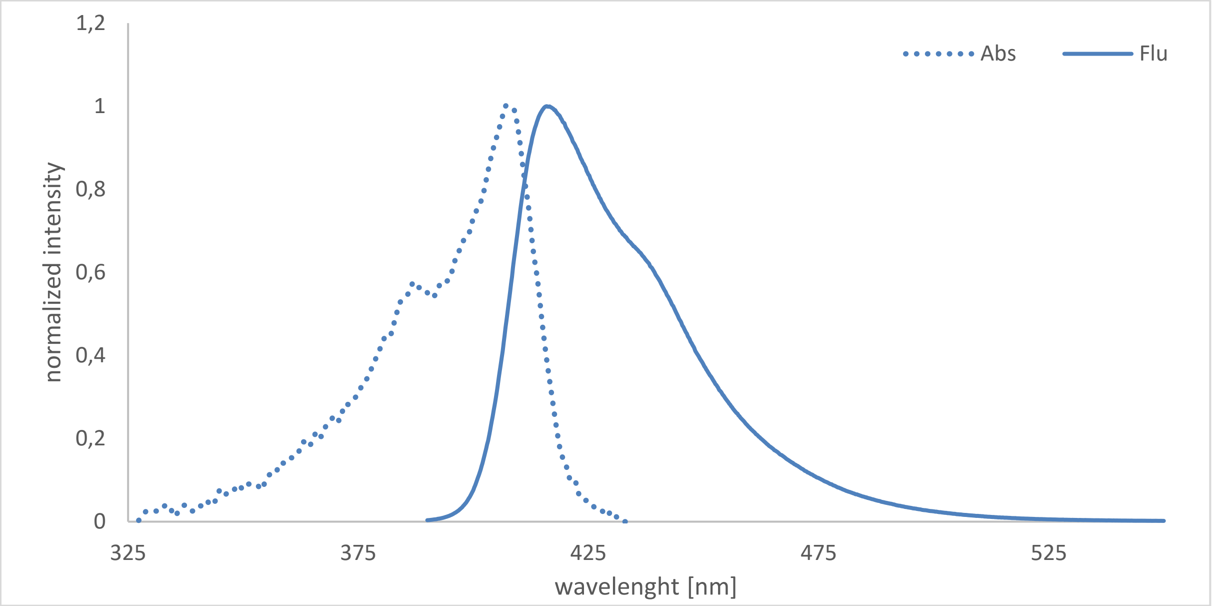

The absorbance and fluorescence (λ_{EX} = 375 nm) spectrum of **1a** in chloroform. The compound has Φ = 0.48 in chloroform solution.

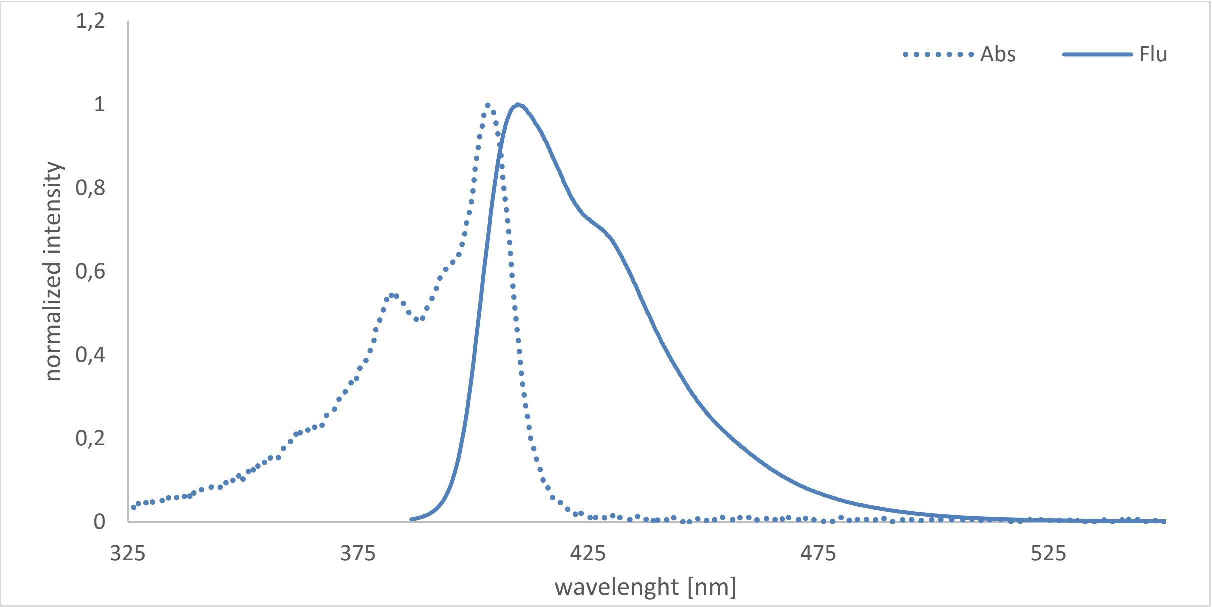

The absorbance and fluorescence (λ_{EX} = 380 nm) spectrum of **1a** in acetonitrile. The compound has Φ = 0.28 in acetonitrile solution.

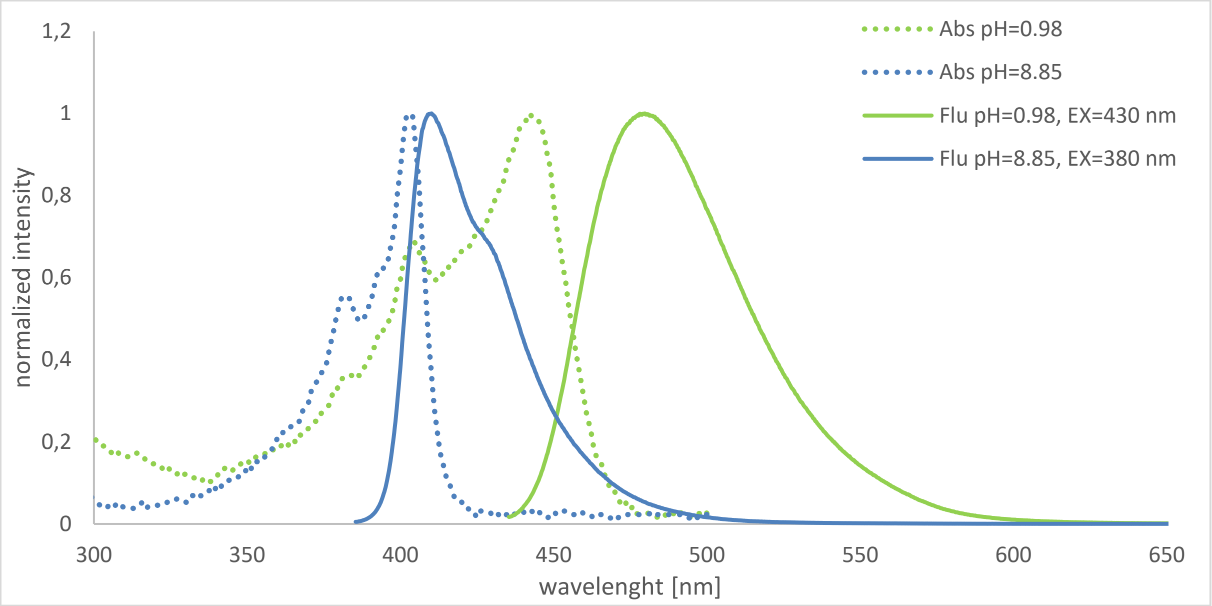

The absorbance and fluorescence spectra of **1a** in acetonitrile solution registered at apparent pH = 0.98 and 8.85. The compound has Φ = 0.36 (λ_{EX} = 430 nm) in acetonitrile solution at apparent pH = 0.98 and Φ = 0.28 (λ_{EX} = 380 nm) at apparent pH = 8.85.

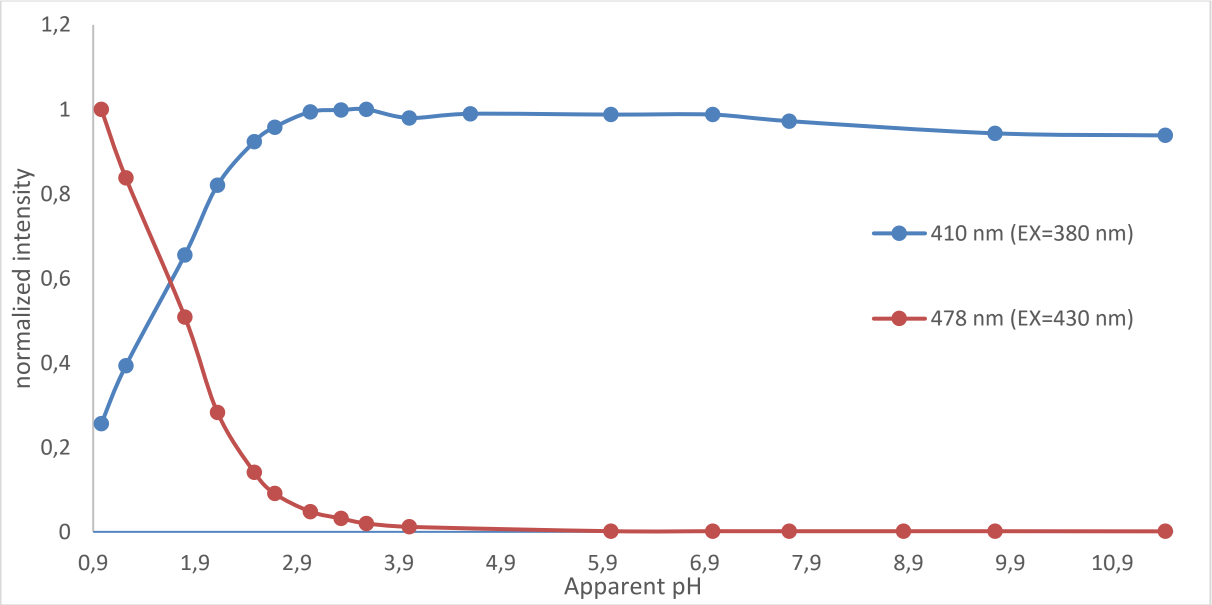

Chart S1. Fluorescence titration of **1a** solution in acetonitrile. The graph presented the change of two fluorescence band intensities, one with maxima at: 410 nm (λ_{EX} = 380 nm) and the second with maxima at 478 nm (λ_{EX} = 430 nm).

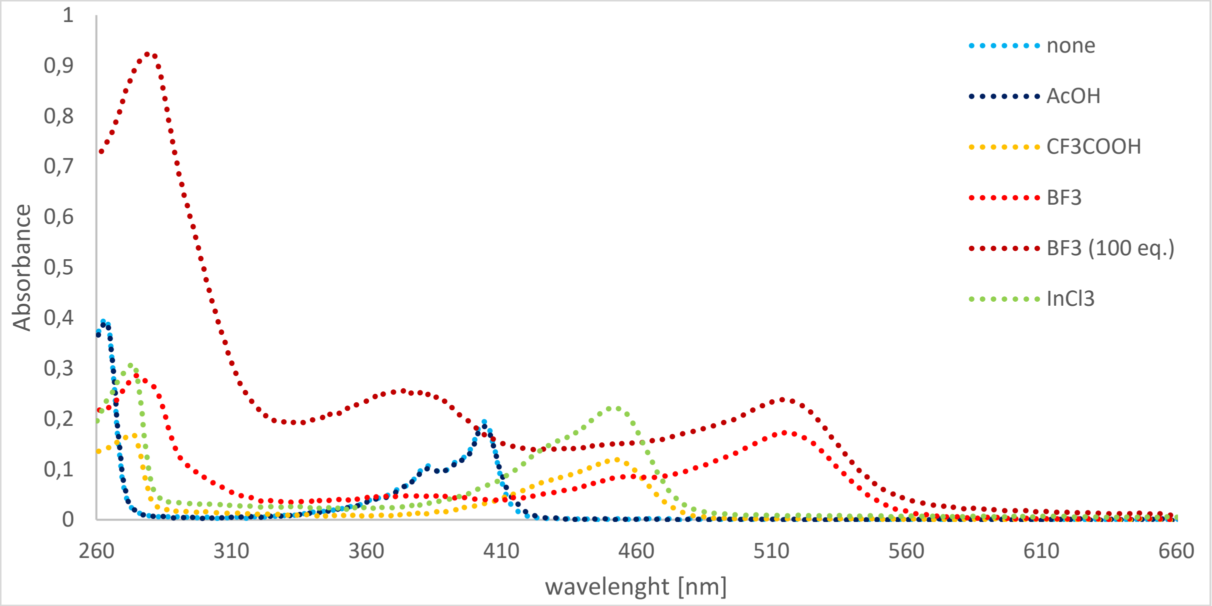

The absorption spectra of the **1a** compound solution in acetonitrile with the addition of acids: acetic acid (AcOH), trifluoroacetic acid (CF_3COOH), trifluoroborate etherate (BF_3) and Indium chloride ($InCl_3$). All samples were made using one solution of **1a** with the same dilution thus band intensities are quantitatively comparable.

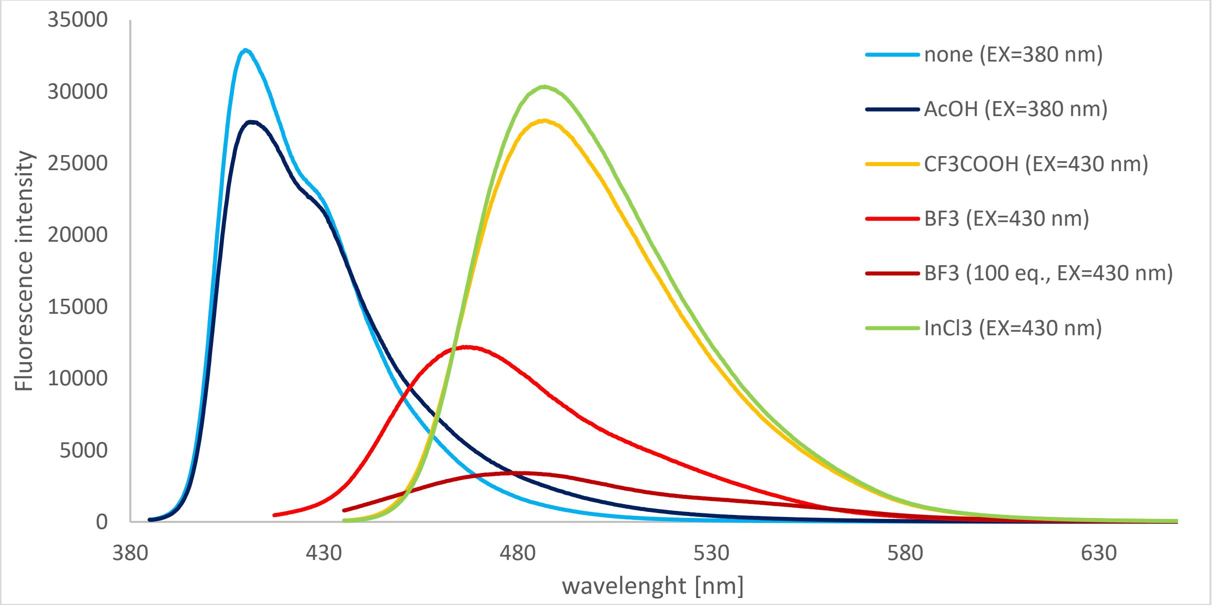

The fluorescence spectra of the **1a** compound solution in acetonitrile with the addition of acids: acetic acid (AcOH), trifluoroacetic acid (CF_3COOH), trifluoroborate etherate (BF_3) and Indium chloride ($InCl_3$). All samples were made using one solution of **1a** with the same dilution thus band intensities are quantitatively comparable. The sample with acetic acid has Φ = 0.27 (λ_{EX} = 380 nm), with trifluoroacetic acid Φ = 0.36 (λ_{EX} = 430 nm), with trifluoroborate etherate Φ = 0.16 (10 eq., λ_{EX} = 430 nm) and Φ = 0.06 (100 eq., λ_{EX} = 430 nm), with indium chloride Φ = 0.42 (λ_{EX} = 430 nm).

Table S1. The qualitative effect of acid addition on 1a spectra in acetonitrile.

Acid [10 eq.]	Abs max [nm]	Flu max [nm]	ΔAbs [nm]	ΔFlu [nm]	Φ
-	404	410	-	-	0.28
CH_3COOH	404	412	0	2	0.27
CF_3COOH	451	487	47	77	0.36
CH_3SO_2OH	442	480	38	70	0.36
BF_3	516	486	113	76	0.16
BF_3 [100 eq.]	516	480	113	70	0.06
$InCl_3$	452	486	48	77	0.42

10-((7-(dodecyloxy)-8-(isopentyloxy)-3-methoxyphenazin-2-yl)oxy)decan-1-ol (1b)

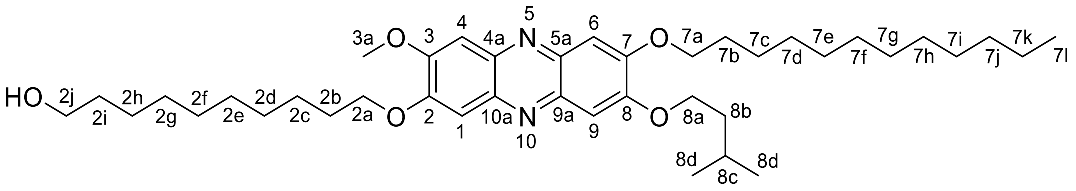

The compound was synthesised from **3b**. Received pale yellow solid. M.p. = 85.0 – 86.5 °C. Yield = 227 mg (87 %).

^{1}H NMR (600 MHz, Chloroform-*d*) δ: 7.31 – 7.25 (m, 4H, $H_{1,4,6,9}$), 4.23 – 4.11 (m, 6H, $H_{2a,7a,8a}$), 4.04 – 4.00 (m, 3H, H_{3a}), 3.60 (tt, *J* = 6.6, 2.1 Hz, 2H, H_{2j}), 1.98 – 1.83 (m, 5H, $H_{2b,7b,8c}$), 1.85 – 1.76 (m, 2H, H_{8b}), 1.55 – 1.50 (m, 2H, H_{2i}), 1.51 – 1.44 (m, 4H, $H_{2c,7c}$), 1.39 – 1.11 (m, 26H, $H_{2d,2e,2f,2g,2h,7d,7e,7f,7g,7h,7i,7j,7k}$), 0.98 (dt, *J* = 6.6, 2.1 Hz, 6H, H_{8d}), 0.87 – 0.79 (m, 3H, H_{7l}).

^{13}C{^{1}H} NMR (151 MHz, Chloroform-*d*) δ: 153.4 (C_2), 153.14 (C_3), 153.10 (C_7), 152.6 (C_8), 139.74 – 139.66 (m, C_{5a}), 139.70 – 139.61 (m, C_{9a}), 139.61 – 139.52 (m, C_{4a}), 139.45 – 139.36 (m, C_{10a}), 105.75 – 105.68 (m, C_4), 105.63 – 105.53 (m, $C_{2,6}$), 105.17 – 105.08 (m, C_9), 69.3 (C_{2a}), 69.1 (C_{7a}), 67.7 (C_{8a}), 63.0 – 62.8 (m, C_{2j}), 56.3 (C_{3a}), 37.5 (C_{8b}), 32.8 (C_{2i}), 32.0 (C_{7j}), 29.7 (C_{2b}), 29.69 (C_{7b}), 29.67 (C_{2d}), 29.64 (C_{7d}), 29.55 (C_{2f}), 29.48 (C_{7e}), 29.4 ($C_{2e,7h}$), 29.41 (C_{7g}), 29.36 (C_{7f}), 28.9 (C_{7i}), 28.8 (C_{2g}), 26.1 (C_{8c}), 26.0 (C_{2h}), 25.8 (C_{2c}), 25.5 (C_{7c}), 22.73 (C_{7k}), 22.70 (C_{8d}), 14.2 (C_{7l}).

HRMS (ESI) *m/z* Calculated for $C_{40}H_{65}N_2O_5$ $[M+H]^+$: 653.4888, found: 653.4889.

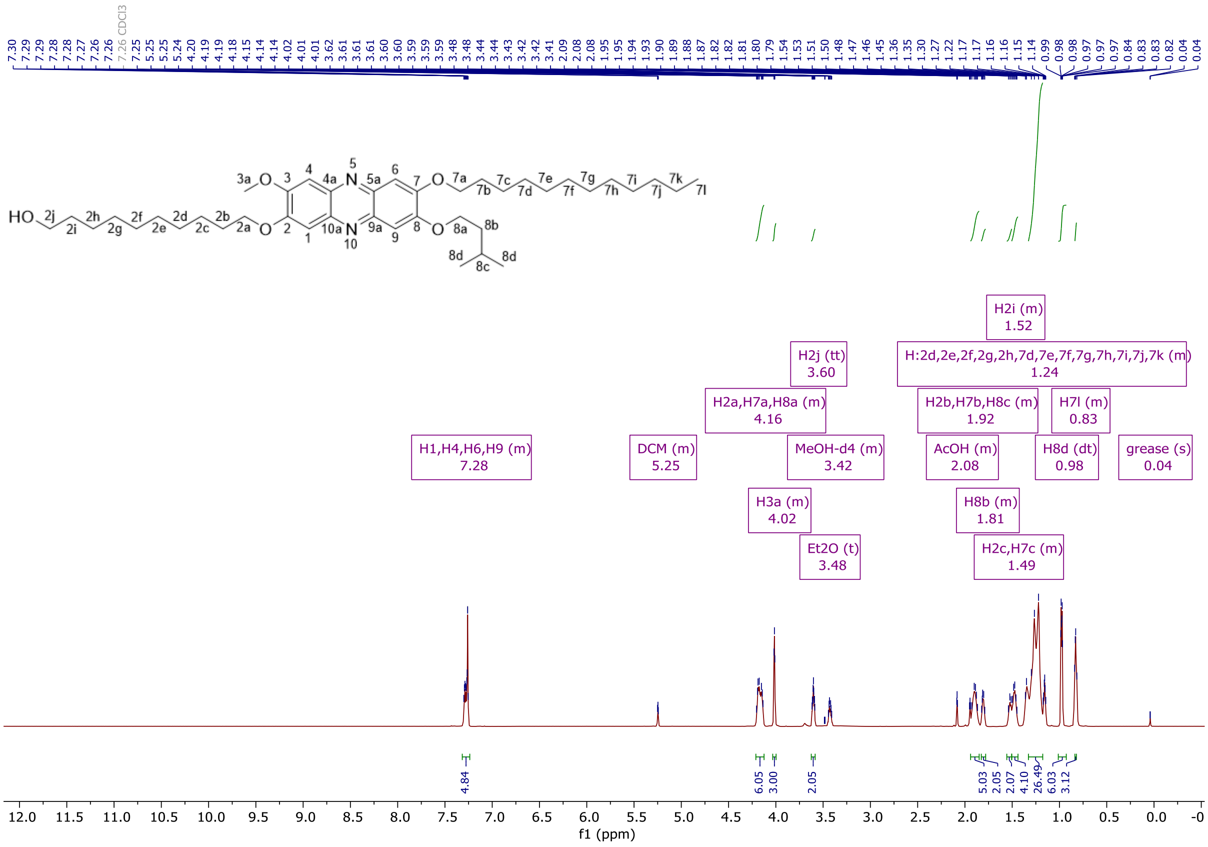

^{1}H NMR (600 MHz, Chloroform-*d*) spectrum of **1b**.

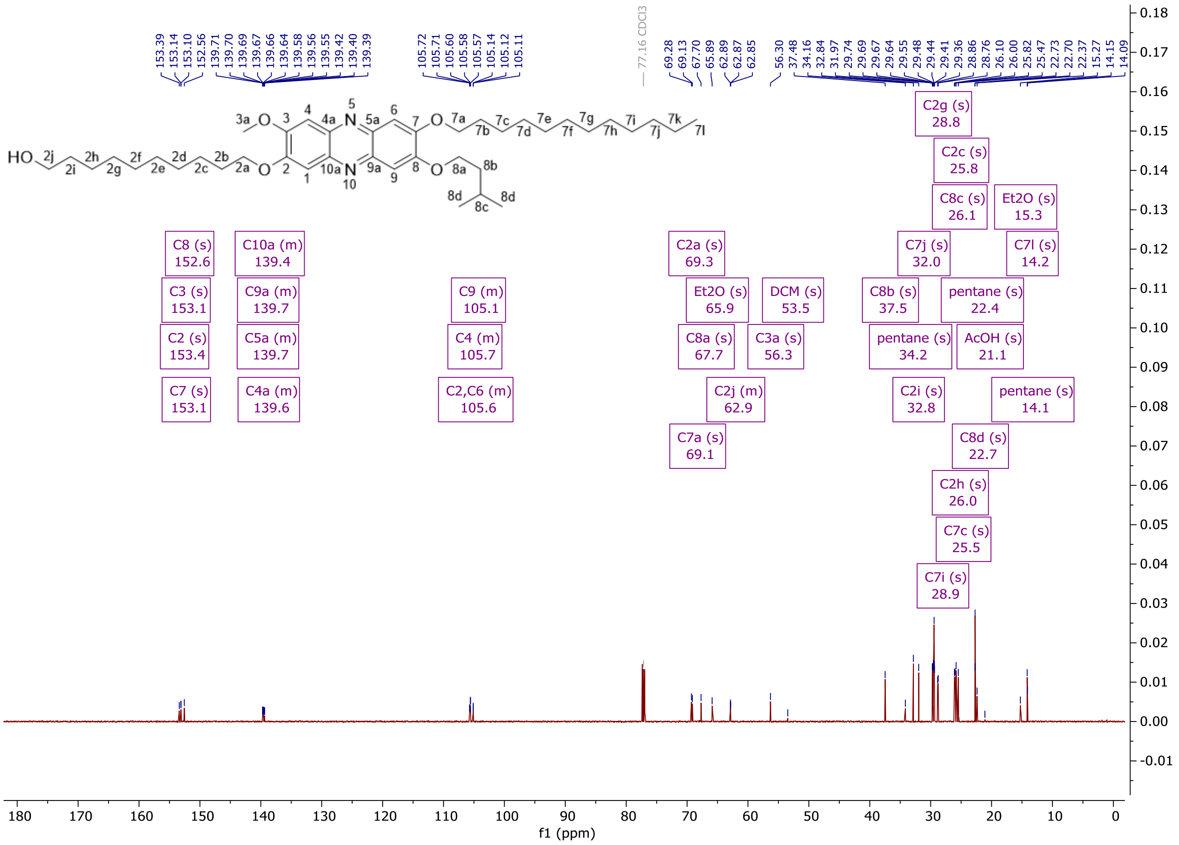

$^{13}C\{^{1}H\}$ NMR (151 MHz, Chloroform-*d*) spectrum of **1b**.

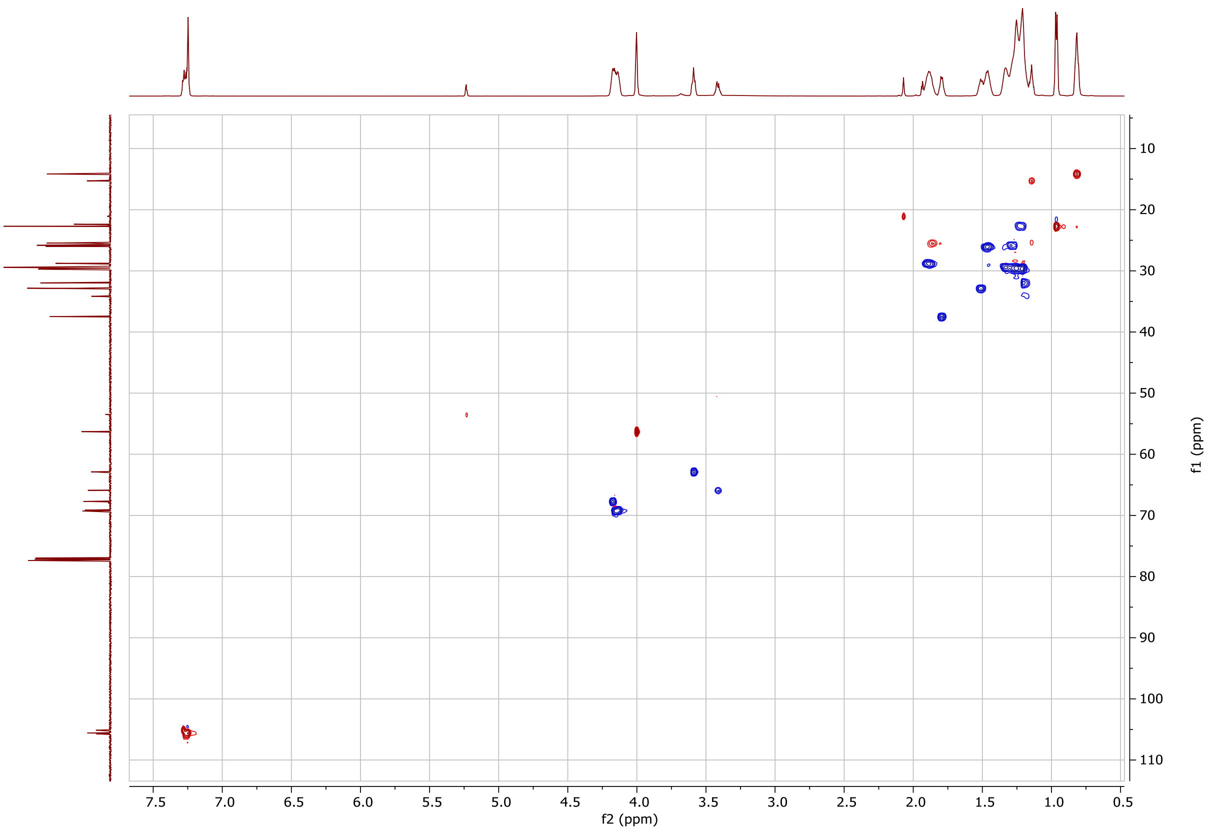

g-HSQC NMR (Chloroform-*d*) spectrum of **1b**.

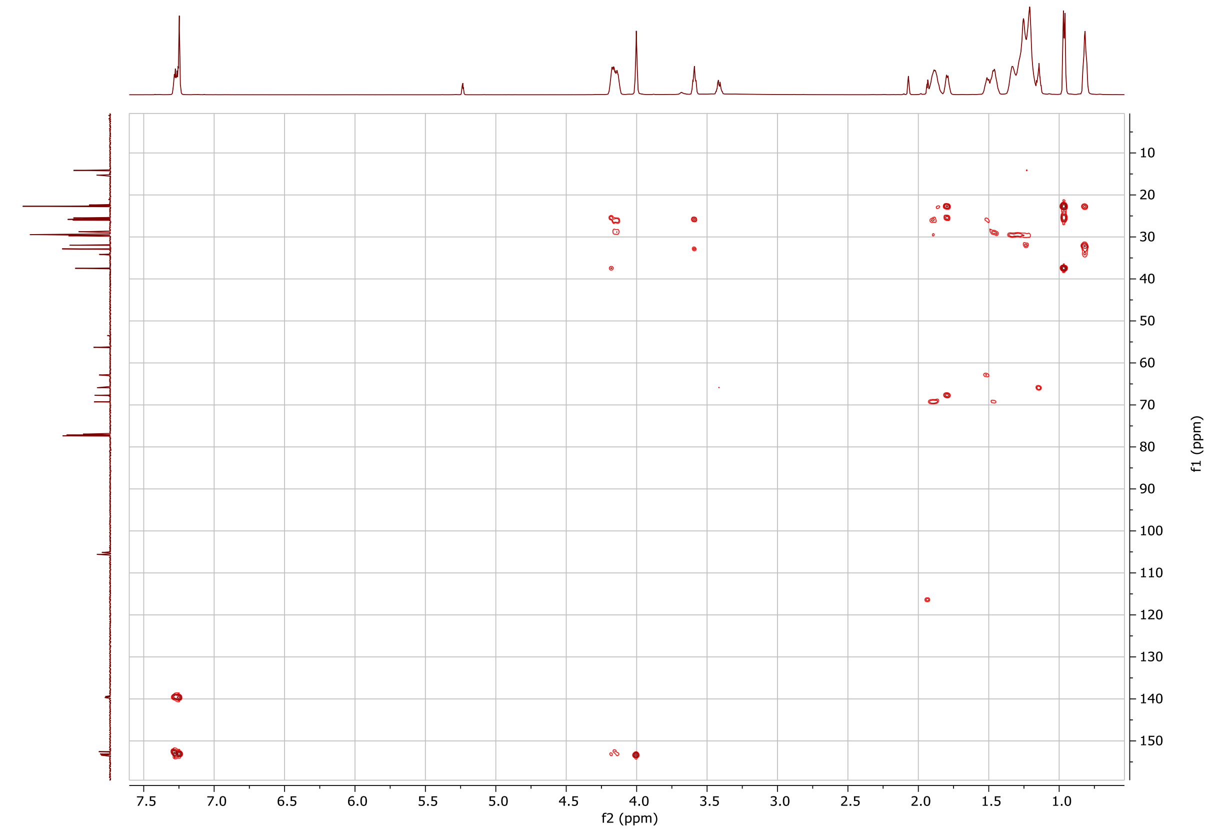

g-HMBC NMR (Chloroform-*d*) spectrum of **1b**.

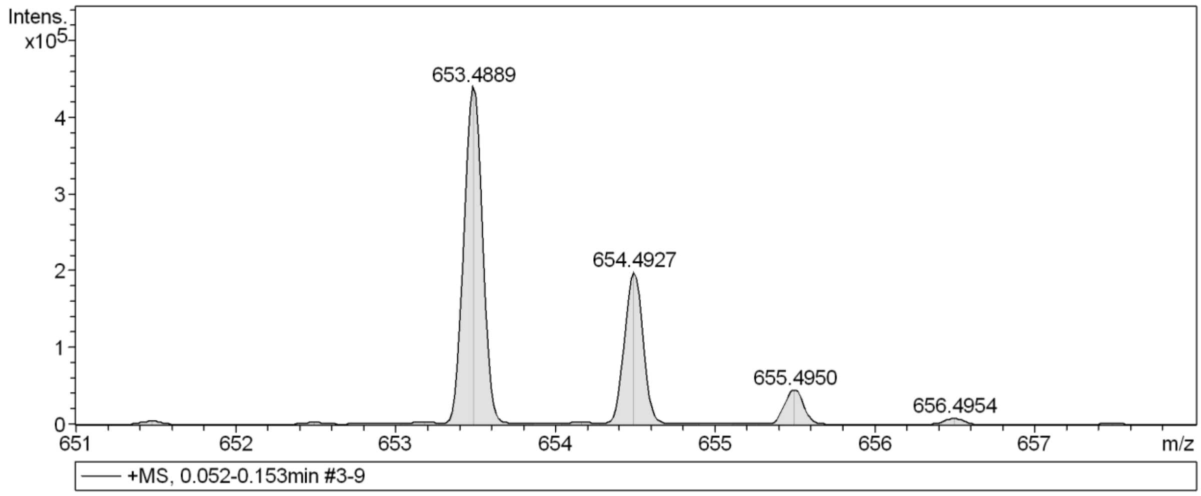

HRMS (ESI+) spectrum of **1b**.

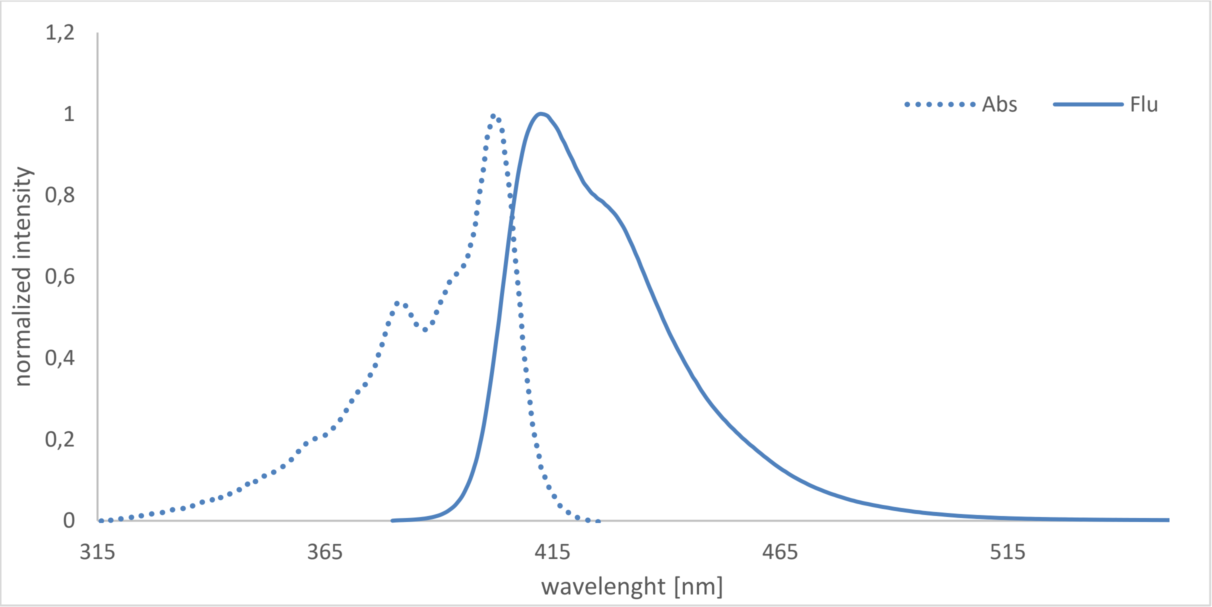

The absorbance and fluorescence (λ_{EX} = 375 nm) spectrum of **1b** in acetonitrile. The compound has Φ = 0.15 in acetonitrile solution.

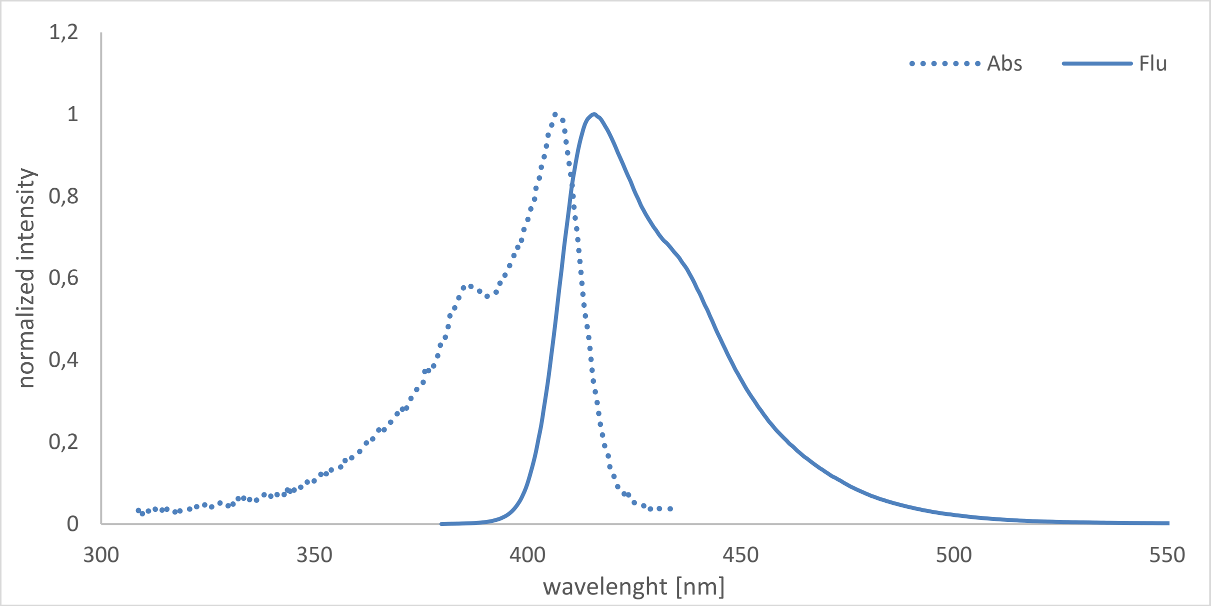

The absorbance and fluorescence (λ_{EX} = 375 nm) spectrum of **1b** in chloroform. The compound has Φ = 0.57 in chloroform solution.

10,10'-((3,7-dimethoxyphenazine-2,8-diyl)bis(oxy))bis(decan-1-ol) (1c)

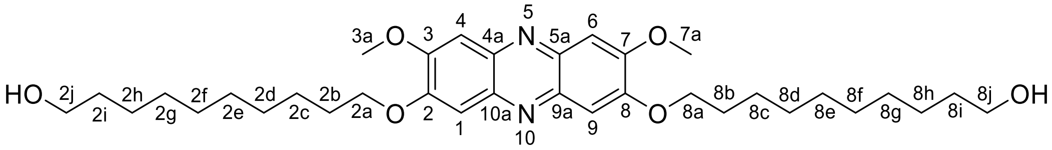

The compound was synthesised from **3c**. Received pale yellow solid. M.p. = 133.4 – 135.1 °C. Yield = 192 mg (82 %).

^{1}H NMR (600 MHz, Chloroform-*d*) δ: 7.34 (s, 2H, $H_{1,9}$), 7.33 (s, 2H, $H_{4,6}$), 4.22 (t, *J* = 6.9 Hz, 4H, $H_{2a,8a}$), 4.07 (s, 6H, $H_{3a,7a}$), 3.64 (t, *J* = 6.6 Hz, 4H, $H_{2j,8j}$), 2.00 – 1.93 (m, 4H, $H_{2b,8b}$), 1.62 – 1.53 (m, 4H, $H_{2i,8i}$), 1.56 – 1.46 (m, 4H, $H_{2c,8c}$), 1.43 – 1.29 (m, 20H, $H_{2d,2e,2f,2g,2h,8d,8e,8f,8g,8h}$).

^{13}C{^{1}H} NMR (151 MHz, Chloroform-*d*) δ: 153.6 ($C_{3,7}$), 152.8 ($C_{2,8}$), 139.9 ($C_{4a,5a}$), 139.8 ($C_{9a,10a}$), 105.9 ($C_{4,6}$), 105.4 ($C_{1,9}$), 69.4 ($C_{2a,8a}$), 63.2 ($C_{2j,8j}$), 56.5 ($C_{3a,7a}$), 32.9 ($C_{2i,8i}$), 29.6 ($C_{2b,8b}$), 29.6 ($C_{2e,8e}$), 29.5 ($C_{2d,8d}$), 29.4 ($C_{2f,8f}$), 28.8 ($C_{2g,8g}$), 26.1 ($C_{2h,8h}$), 25.9 ($C_{2c,8c}$).

HRMS (ESI) *m/z* Calculated for $C_{34}H_{53}N_2O_6$ $[M+H]^+$: 585.3898, found: 585.3897.

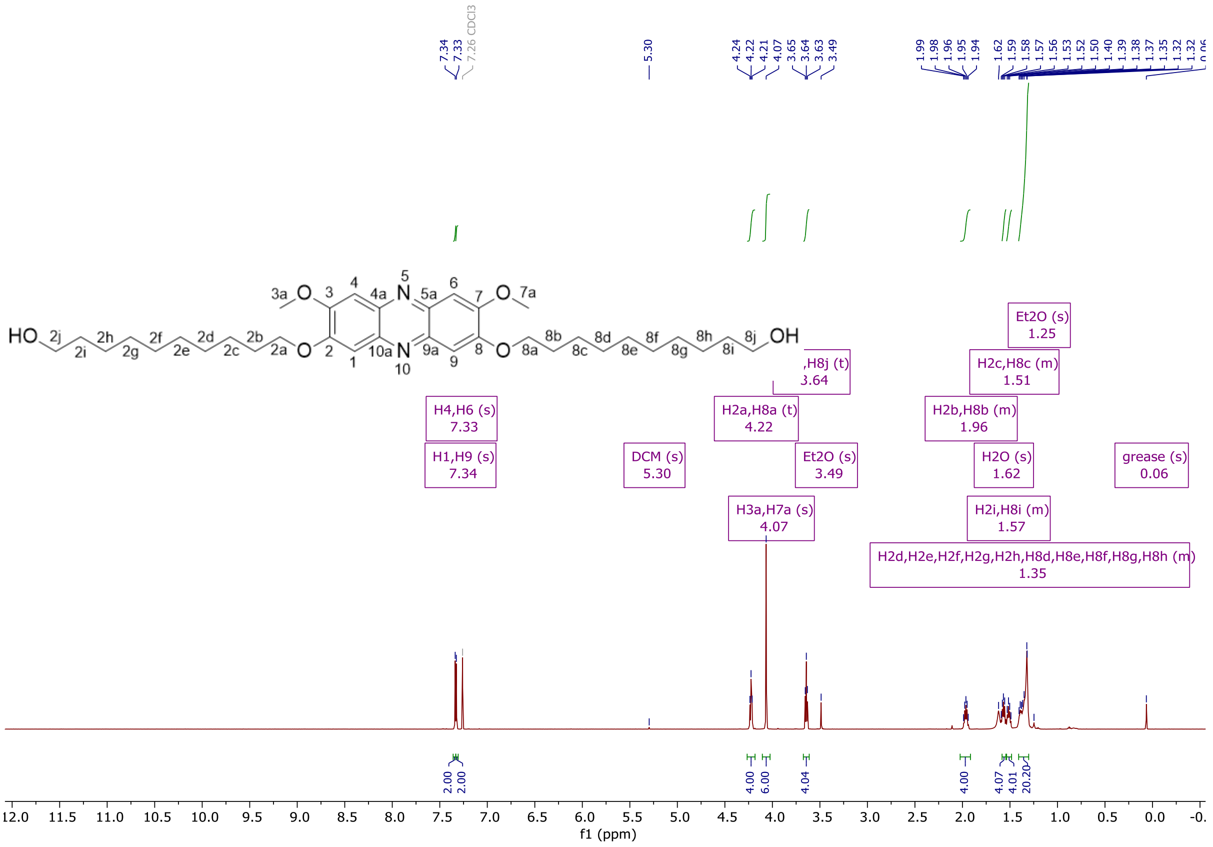

^{1}H NMR (600 MHz, Chloroform-*d*) spectrum of **1c**.

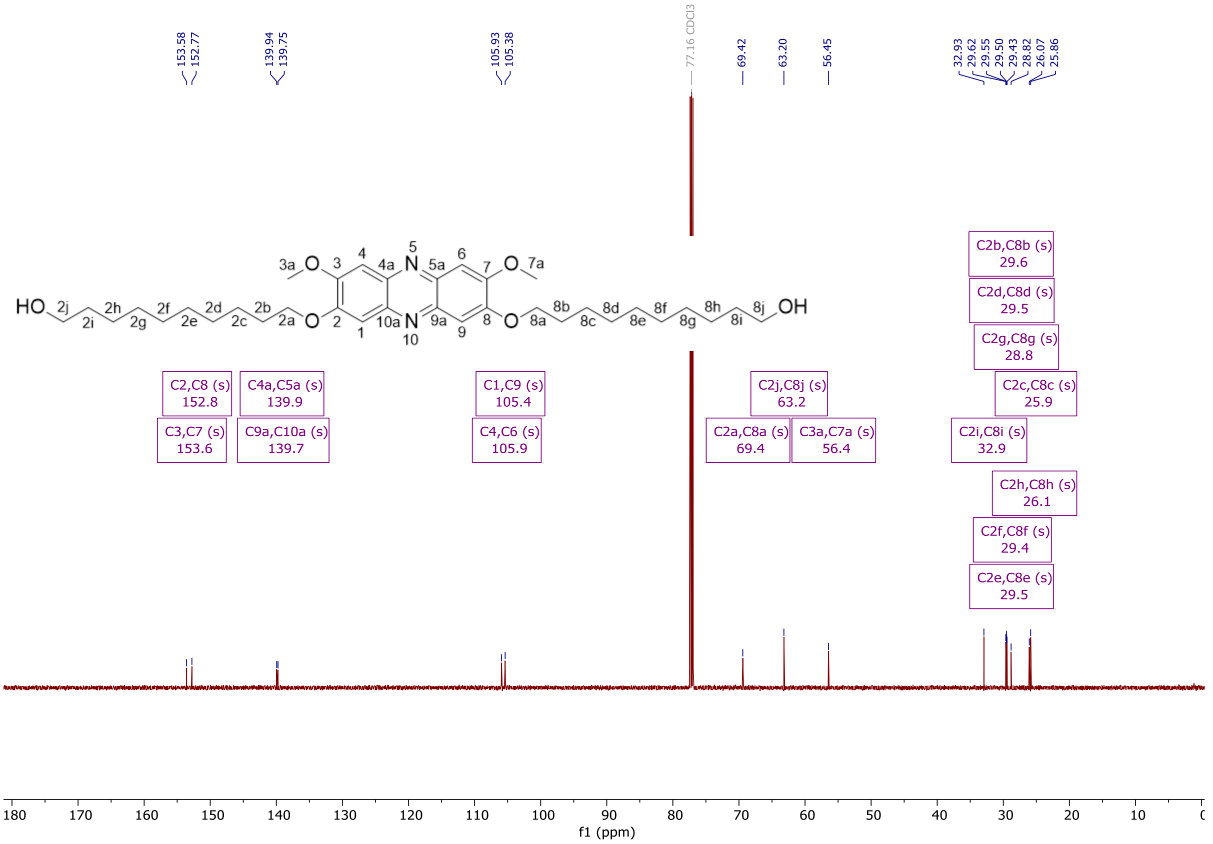

^{13}C{^{1}H} NMR (151 MHz, Chloroform-*d*) spectrum of **1c**.

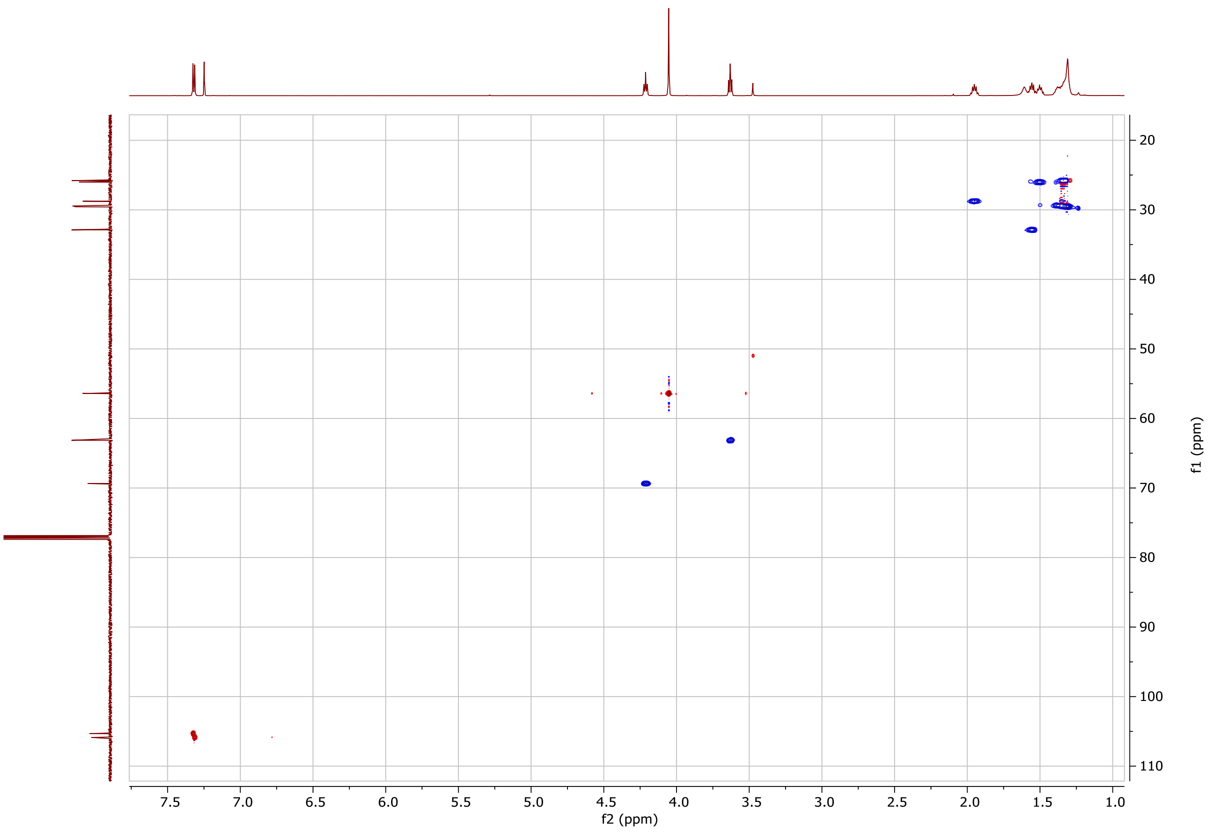

g-HSQC NMR (Chloroform-*d*) spectrum of **1c**.

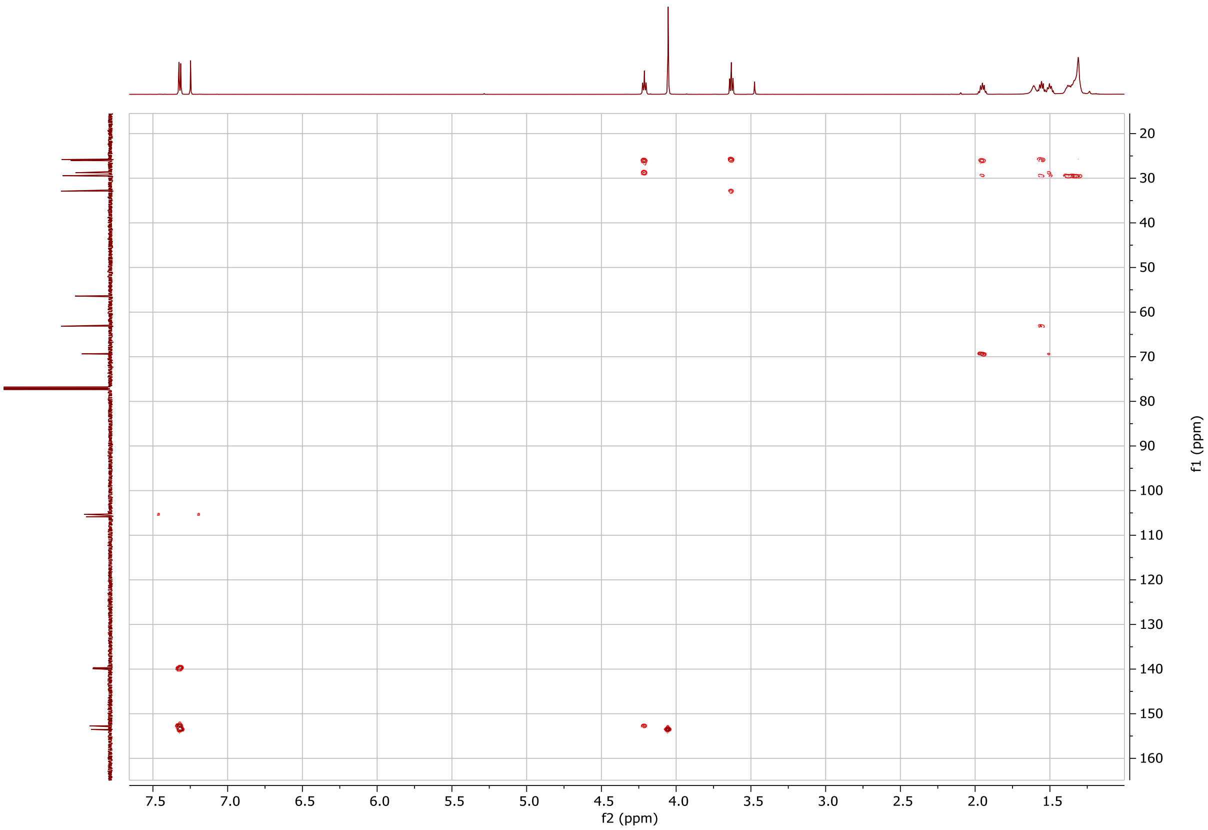

g-HMBC NMR (Chloroform-*d*) spectrum of **1c**.

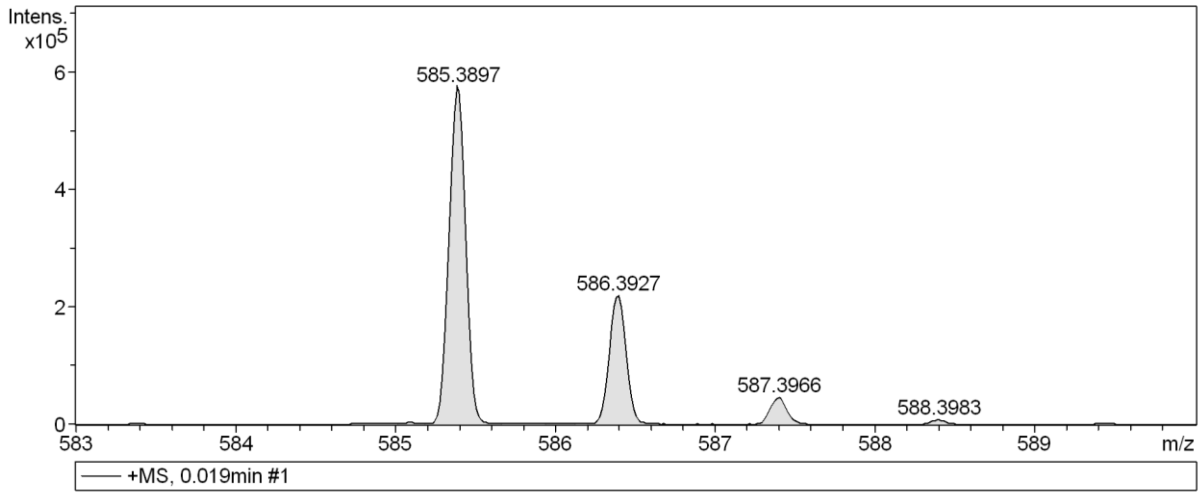

HRMS (ESI+) spectrum of **1c**.

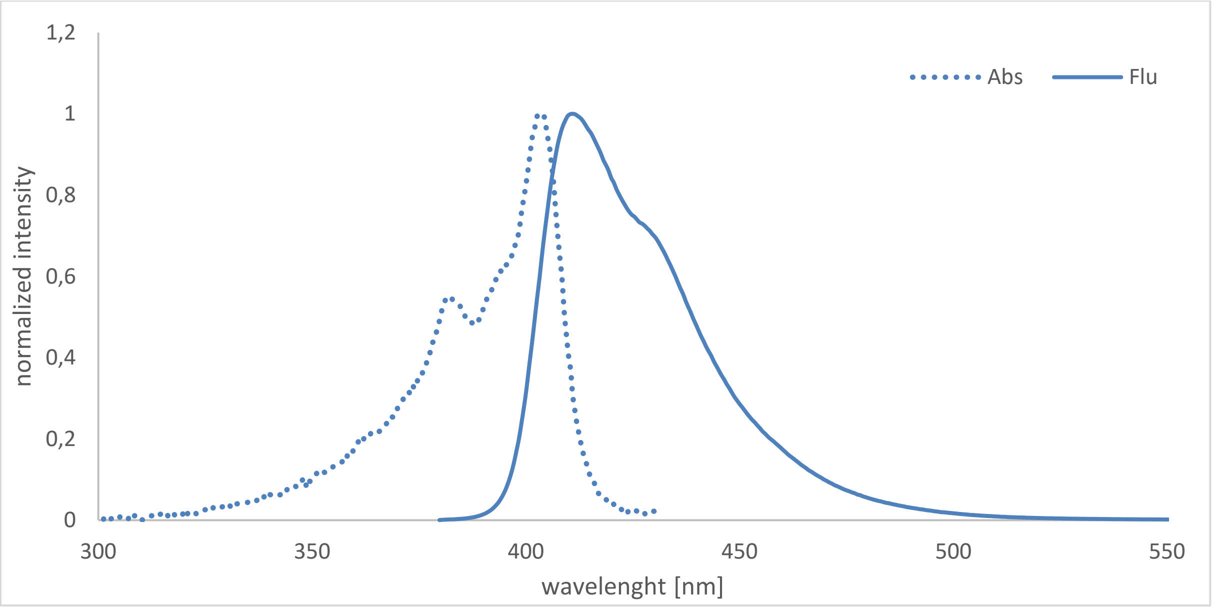

The absorbance and fluorescence (λ_{EX} = 375 nm) spectrum of **1c** in acetonitrile. The compound has Φ = 0.12 in acetonitrile solution.

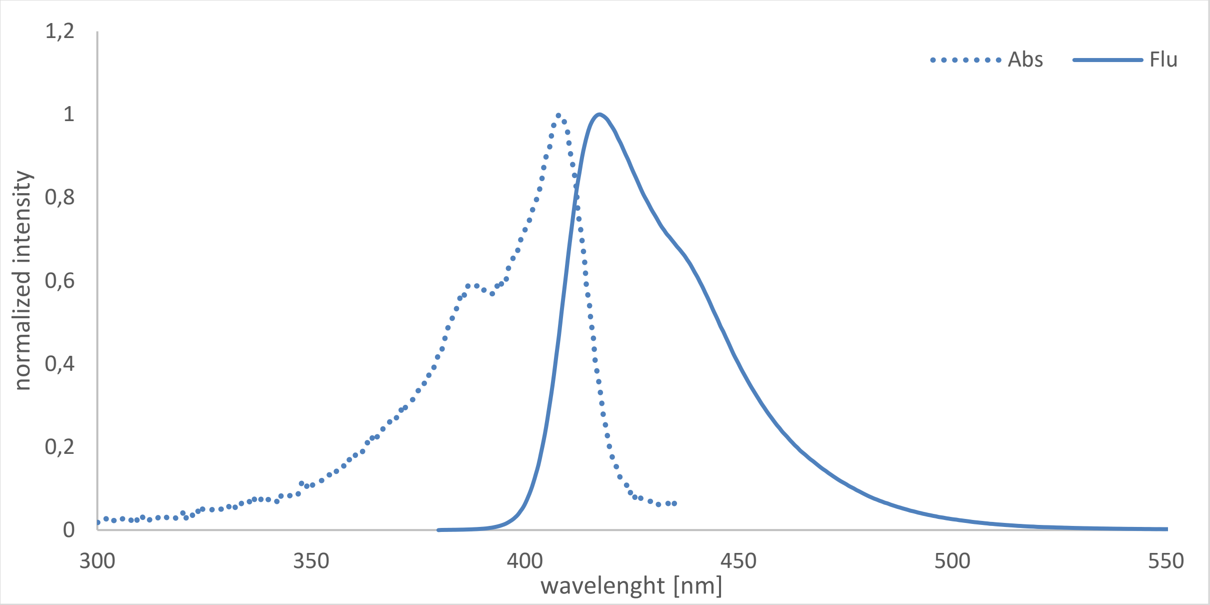

The absorbance and fluorescence (λ_{EX} = 375 nm) spectrum of **1c** in chloroform. The compound has Φ = 0.49 in chloroform solution.

10-((3-methoxy-7-(trifluoromethyl)phenazin-2-yl)oxy)decan-1-ol (1d)

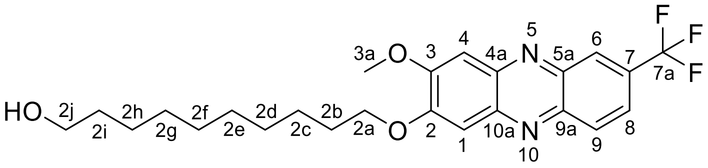

The compound was synthesised from **3d**. Received pale yellow solid. M.p. = 131.6 – 133.7 °C. Yield = 150 mg (83 %).

^{1}H NMR (400 MHz, Chloroform-*d*) δ: 8.47 (d, *J* = 1.9 Hz, 1H, H_6), 8.26 (d, *J* = 9.0 Hz, 1H, H_9), 7.89 (dd, *J* = 9.0, 2.0 Hz, 1H, H_8), 7.41 – 7.37 (m, 2H, $H_{1,4}$), 4.27 (t, *J* = 6.8 Hz, 2H, H_{2a}), 4.12 (s, 3H, H_{3a}), 3.65 (t, *J* = 6.6 Hz, 2H, H_{2j}), 2.03 – 1.94 (m, 2H, H_{2b}), 1.61 – 1.48 (m, 4H, $H_{2c,2i}$), 1.43 – 1.30 (m, 10H, $H_{2d,2e,2f,2g,2h}$).

^{13}C{^{1}H} NMR (101 MHz, Chloroform-*d*) δ: 155.8 (C_2), 155.5 (C_3), 145.0 (C_{5a}), 143.0 (C_{9a}), 140.5 ($C_{4a,10a}$), 132.1 – 131.0 (m, C_7), 130.2 – 130.1 (m, C_9), 127.4 – 127.1 (m, C_8), 124.4 (C_6), 130.1 – 120.6 (m, C_{7a}), 105.5 (C_1), 105.2 (C_4), 69.9 (C_{2a}), 63.2 (C_{2j}), 56.8 (C_{3a}), 32.9 (C_{2i}), 29.63 (C_{2b}), 29.55 (C_{2d}), 29.51 (C_{2e}), 29.4 (C_{2f}), 28.7 (C_{2g}), 26.1 (C_{2c}), 25.9 (C_{2h}).

^{19}F NMR (282 MHz, Chloroform-*d*) δ: -62.59 (s, 3F, F_{CF3}). [relative chemical shift – no reference was used]

HRMS (ESI) *m/z* Calculated for $C_{24}H_{29}F_3N_2O_3Na$ $[M+Na]^+$: 473.2028, found: 473.2022.

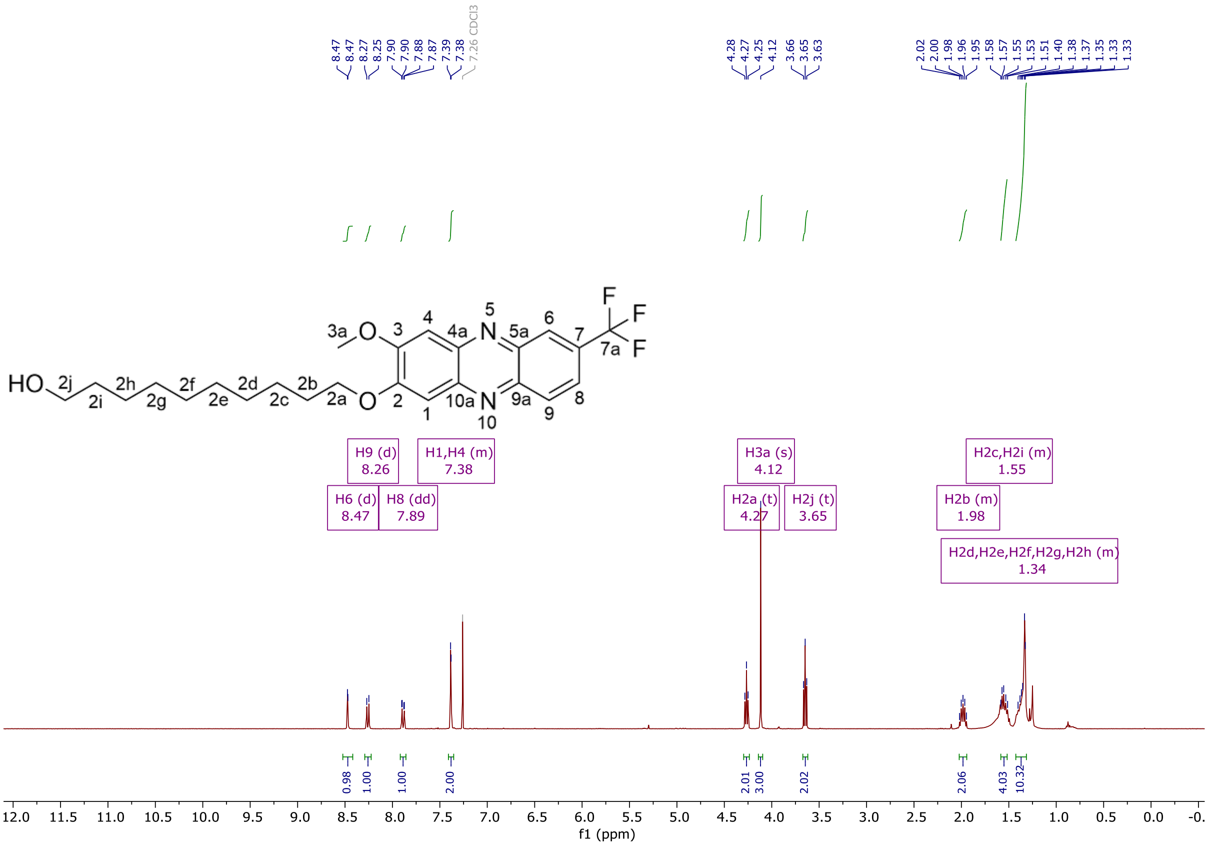

^{1}H NMR (400 MHz, Chloroform-*d*) spectrum of **1d**.

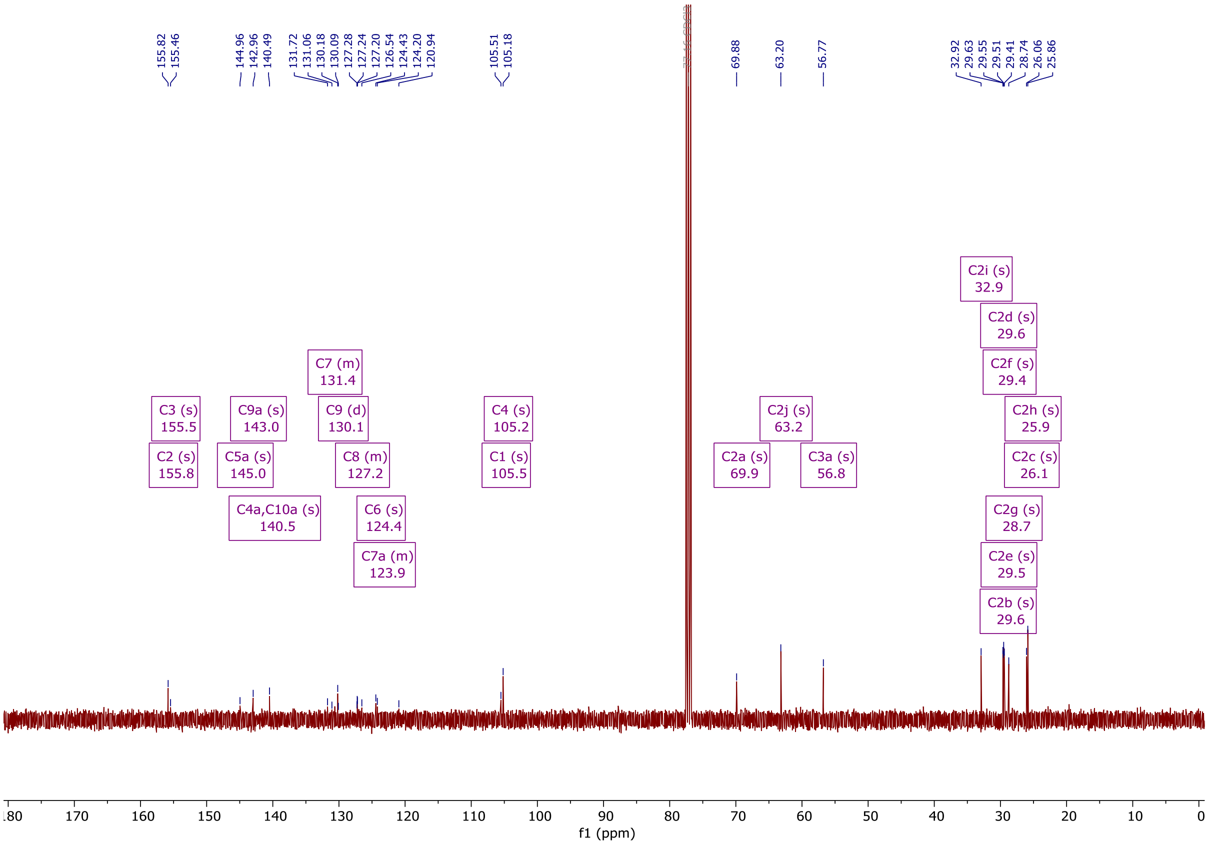

^{13}C{^{1}H} NMR (101 MHz, Chloroform-*d*) spectrum of **1d**.

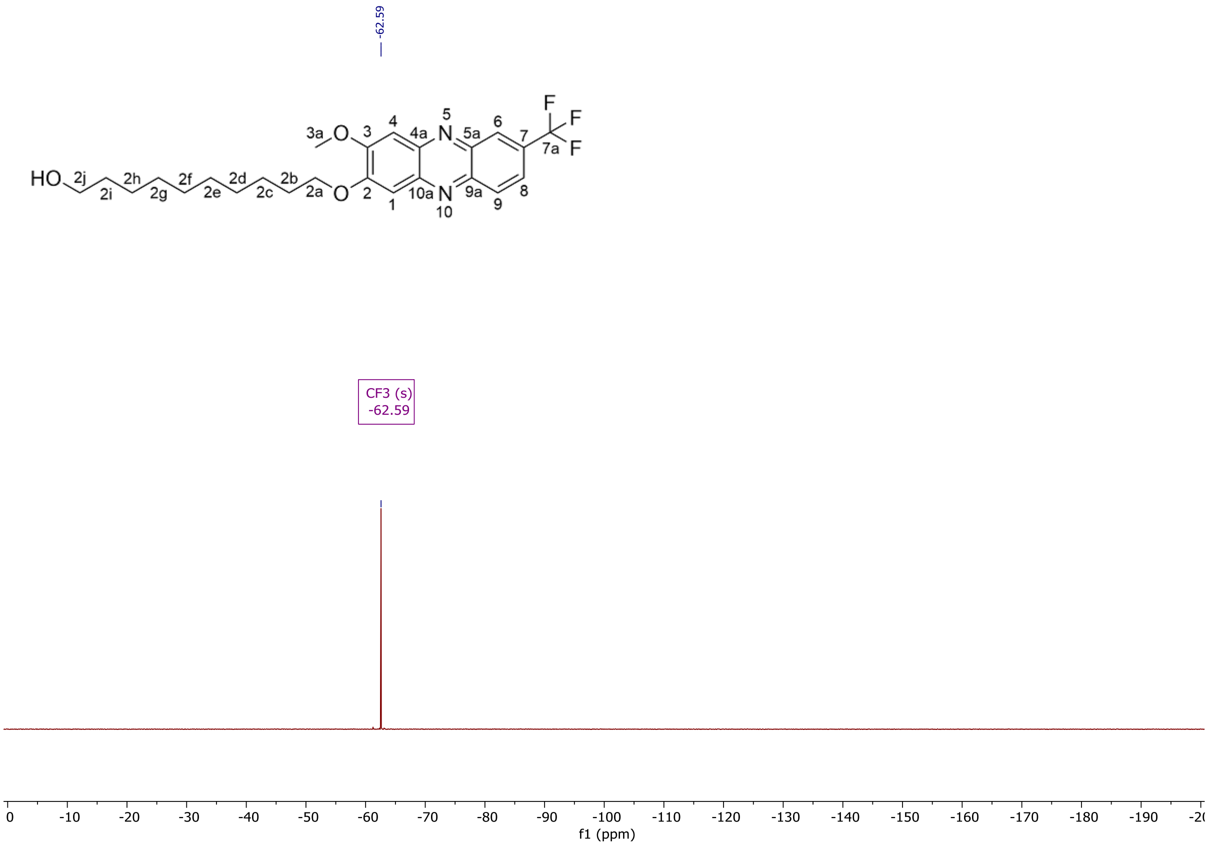

^{19}F NMR (282 MHz, Chloroform-*d*) spectrum of **1d**.

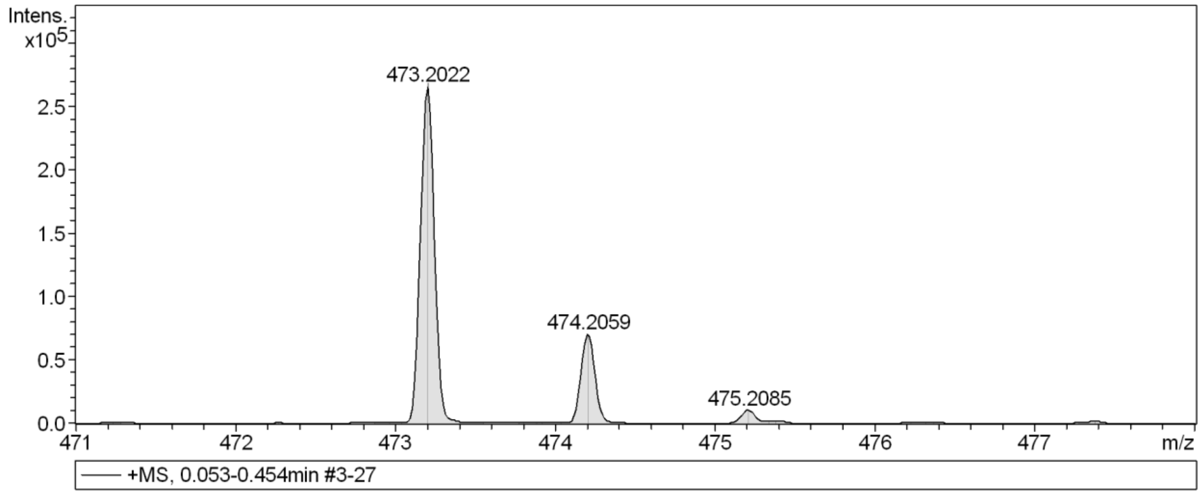

HRMS (ESI+) spectrum of **1d**.

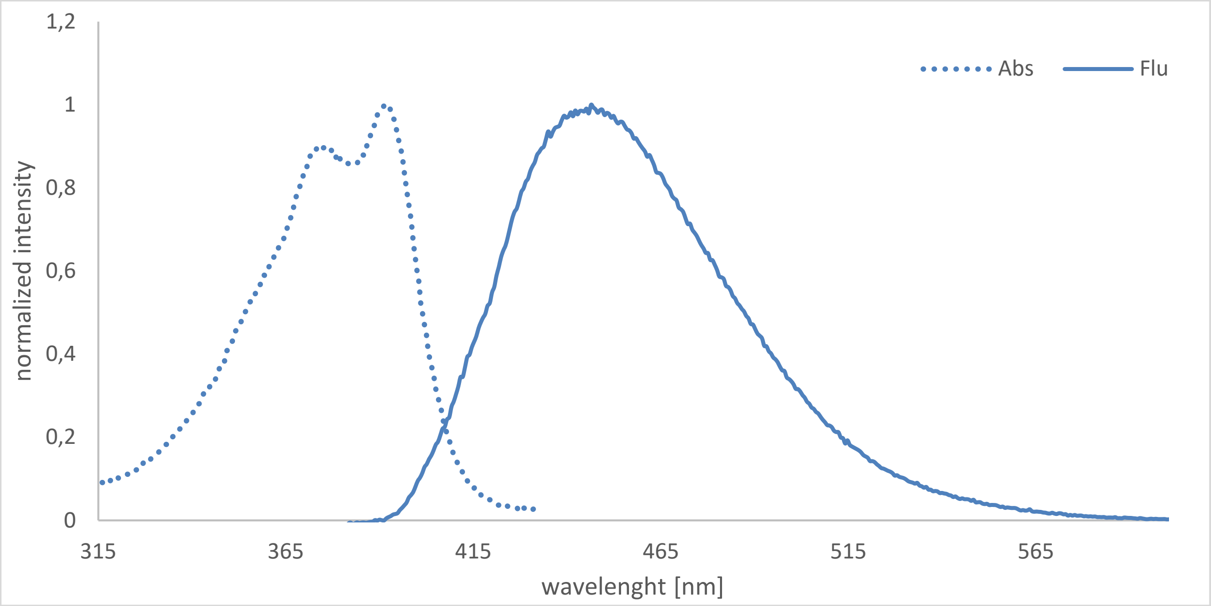

The absorbance and fluorescence (λ_{EX} = 375 nm) spectrum of **1d** in acetonitrile. The compound has Φ = 0.002 in acetonitrile solution.

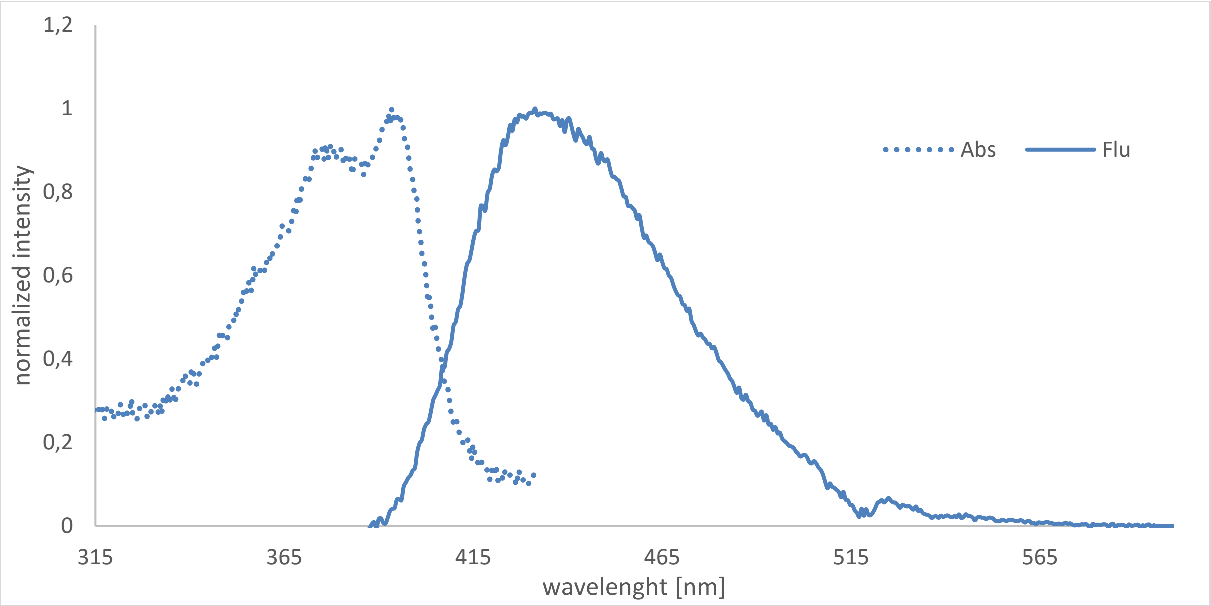

The absorbance and fluorescence (λ_{EX} = 375 nm) spectrum of **1d** in chloroform. The compound has Φ = 0.003 in chloroform solution.

10-((8-(*tert*-butyl)-3-methoxyphenazin-2-yl)oxy)decan-1-ol (1e)

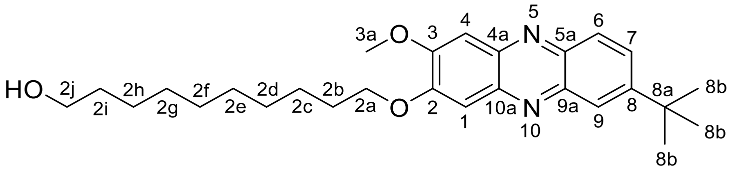

The compound was synthesised from **3e**. Received pale yellow solid. M.p. = 86.7 – 88.5 °C. Yield = 151 mg (86 %).

^{1}H NMR (400 MHz, Chloroform-*d*) δ: 8.10 – 8.02 (m, 2H, $H_{6,9}$), 7.84 (dd, *J* = 9.0, 2.3 Hz, 1H, H_7), 7.37 (s, 1H, H_1), 7.34 (s, 1H, H_4), 4.22 (t, *J* = 6.8 Hz, 2H, H_{2a}), 4.06 (s, 3H, H_{3a}), 3.63 (t, *J* = 6.6 Hz, 2H, H_{2j}), 2.02 – 1.88 (m, 2H, H_{2b}), 1.60 – 1.45 (m, 4H, $H_{2c,2i}$), 1.46 (s, 9H, H_{8b}), 1.41 – 1.25 (m, 10H, $H_{2d,2e,2f,2g,2h}$).

^{13}C{^{1}H} NMR (101 MHz, Chloroform-*d*) δ: 154.4 (C_2), 153.8 (C_3), 152.3 (C_8), 141.8 (C_{9a}), 141.7 (C_{5a}), 141.2 (C_{4a}), 140.4 (C_{10a}), 128.5 (C_6), 128.0 (C_7), 123.3 (C_9), 105.5 (C_4), 105.0 (C_1), 69.3 (C_{2a}), 62.6 (C_{2j}), 56.3 (C_{3a}), 35.2 (C_{8a}), 32.8 (C_{2i}), 30.9 (C_{8b}), 29.5 (C_{2b}), 29.39 (C_{2d}), 29.37 (C_{2e}), 29.3 (C_{2f}), 28.6 (C_{2g}), 25.9 (C_{2c}), 25.8 (C_{2h}).

HRMS (ESI) *m/z* Calculated for $C_{27}H_{39}N_2O_3$ $[M+H]^+$: 439.2955, found: 439.2952.

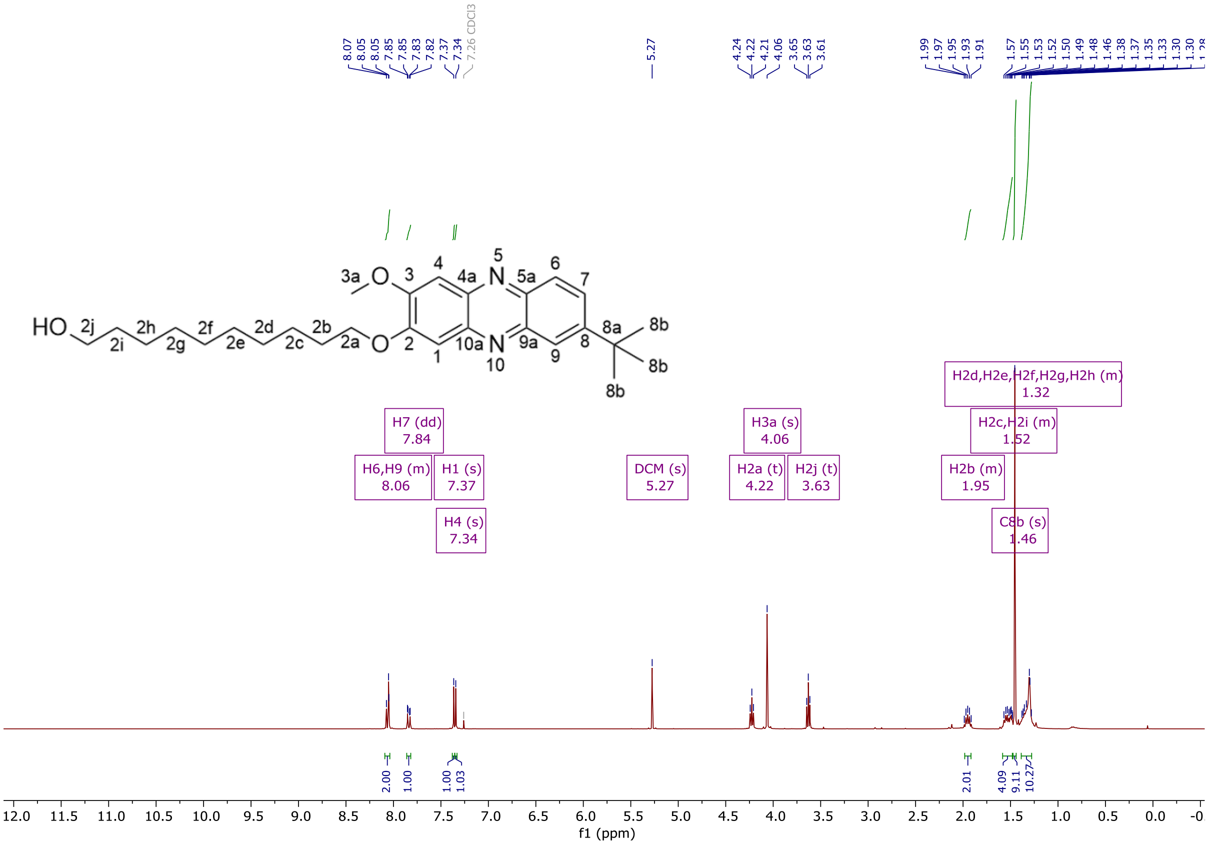

^{1}H NMR (400 MHz, Chloroform-*d*) spectrum of **1e**.

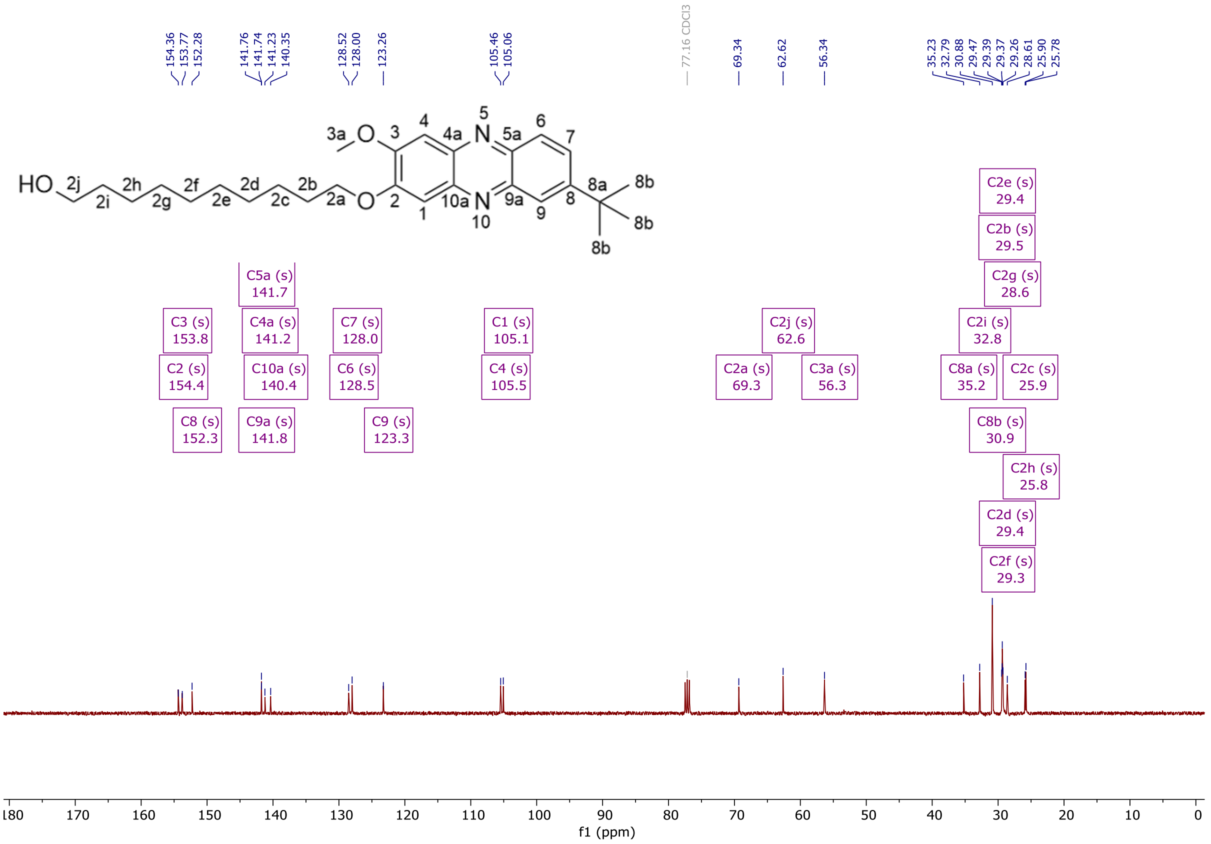

$^{13}C\{^{1}H\}$ NMR (101 MHz, Chloroform-*d*) spectrum of **1e**.

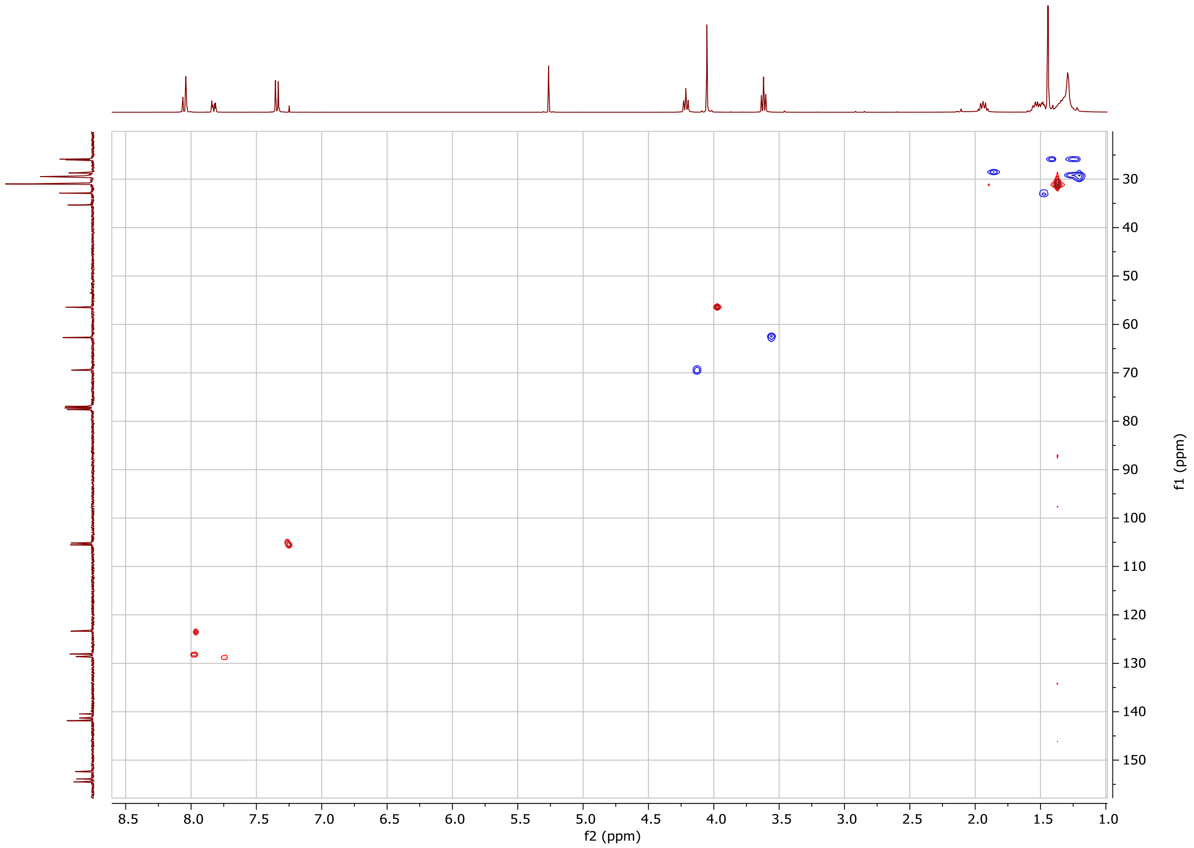

g-HSQC NMR (101 MHz, Chloroform-*d*) spectrum of **1e**.

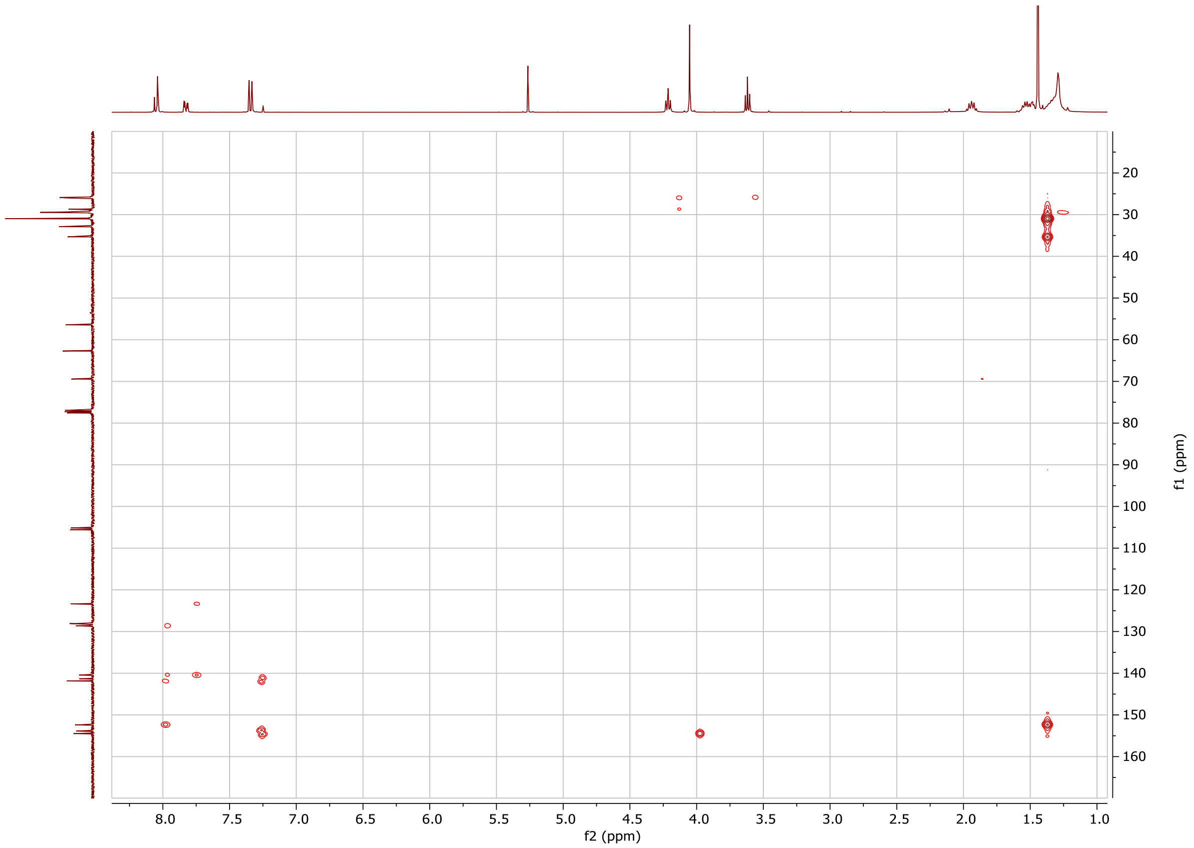

g-HMBC NMR (101 MHz, Chloroform-*d*) spectrum of **1e**.

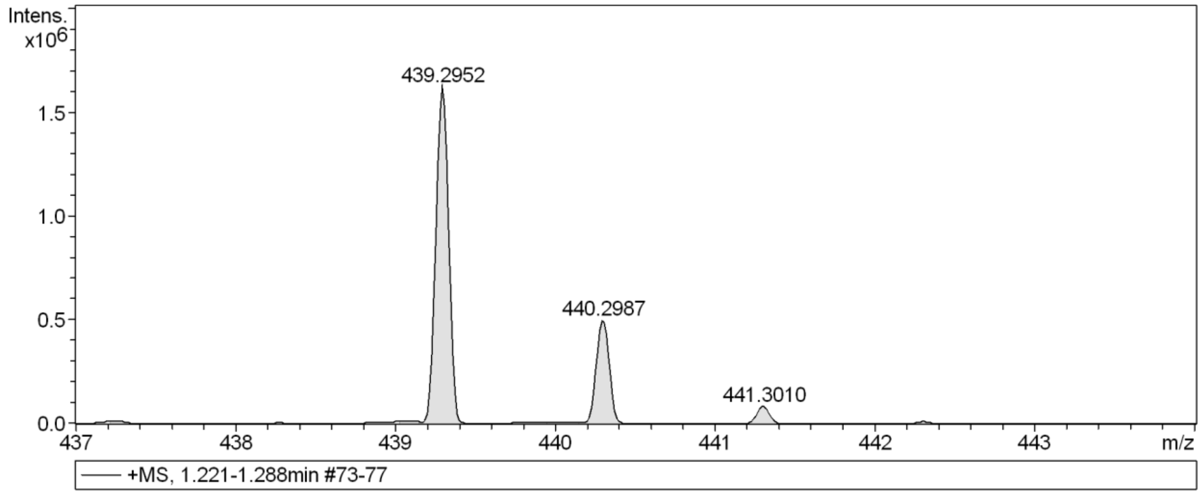

HRMS (ESI+) spectrum of **1e**.

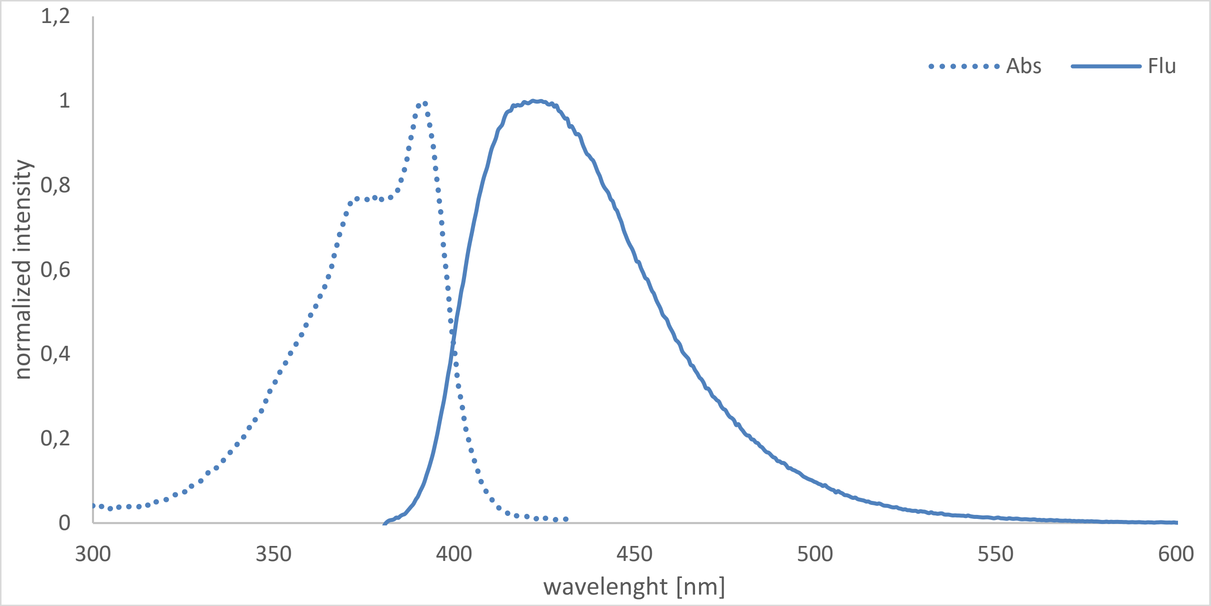

The absorbance and fluorescence (λ_{EX} = 375 nm) spectrum of **1e** in acetonitrile. The compound has Φ = 0.003 in acetonitrile solution.

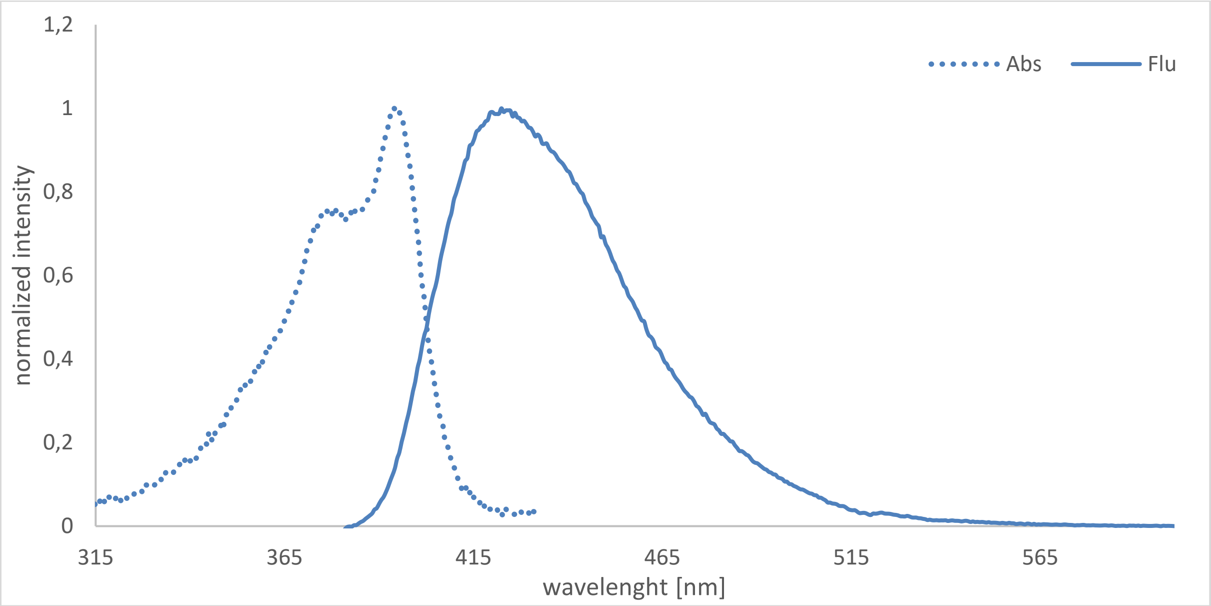

The absorbance and fluorescence (λ_{EX} = 375 nm) spectrum of **1e** in chloroform. The compound has Φ = 0.008 in chloroform solution.

Exchange of chloride counter ion for tetrafluoroborate in phenazinium salts (2a-2f).

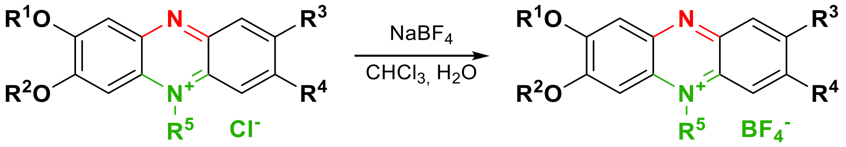

In the round-bottom flask (250 mL of volume), phenazinium salt (0.30 mmol) was dissolved in chloroform (50 mL), and a solution of sodium tetrafluoroborate (330 mg, 10 eq.) in distilled water (50 mL) was added. The mixture was stirred in a closed flask for 24 h, and then the phases were separated. The organic phase was dried over anhydrous magnesium sulphate and concentrated under reduced pressure on a rotary evaporator. Residual phenazinium chloride was removed by short column chromatography (silica gel, DCM / MeOH: 0.5-3%).

For the gram-scale synthesis (**2b**), only the sodium tetrafluoroborate excess was scaled proportionally, and the rest of the protocol remained the same.

2-ethoxy-8-(hexyloxy)-7-isobutoxy-3-methoxy-5-methylphenazin-5-ium chloride (2a Cl$^-$)

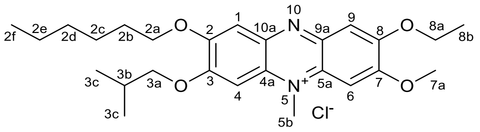

The compound was synthesised from **4a**. Received orange solid. M.p. = 172.0 – 179.2 °C. Yield = 168 mg (88 %)

^{1}H NMR (400 MHz, Chloroform-*d*) δ: 8.08 (s, 1H, H_4), 7.77 (s, 1H, H_6), 7.40 (s, 1H, H_1), 7.39 (s, 1H, H_9), 5.19 (s, 3H, H_{5b}), 4.42 (s, 3H, H_{7a}), 4.30 (q, J = 7.1 Hz, 4H, $H_{3a,8a}$), 4.20 (t, J = 6.3 Hz, 2H, H_{2a}), 2.32 – 2.22 (m, 1H, H_{3b}), 1.99 – 1.86 (m, 2H, H_{2b}), 1.58 (t, J = 6.9 Hz, 3H, H_{8b}), 1.52 (d, J = 15.1 Hz, 2H, H_{2c}), 1.42 – 1.30 (m, 4H, $H_{2d,2e}$), 1.12 (d, J = 6.7 Hz, 6H, H_{3c}), 0.89 (t, J = 6.9 Hz, 3H, H_{2f}).

^{13}C{^{1}H} NMR (101 MHz, Chloroform-*d*) δ: 159.60 (C_3), 159.55 (C_7), 153.7 (C_8), 153.1 (C_2), 140.9 (C_{9a}), 140.7 (C_{10a}), 130.2 (C_{5a}), 130.1 (C_{4a}), 107.7 (C_1), 107.6 (C_9), 97.8 (C_4), 97.1 (C_6), 77.4 (C_{3a}), 69.8 (C_{2a}), 65.6 (C_{8a}), 59.4 (C_{7a}), 41.0 (C_{5a}), 31.5 (C_{5b}), 29.8 (C_{2d}), 28.7 (C_{2b}), 28.3 (C_{3b}), 25.7 (C_{2c}), 22.6 (C_{2e}), 19.2 (C_{3c}), 14.4 (C_{8b}), 14.0 (C_{2f}).

HRMS (ESI) *m/z* Calculated for $C_{26}H_{37}N_2O_4$ [M]$^+$: 441.2748, found: 441.2747.

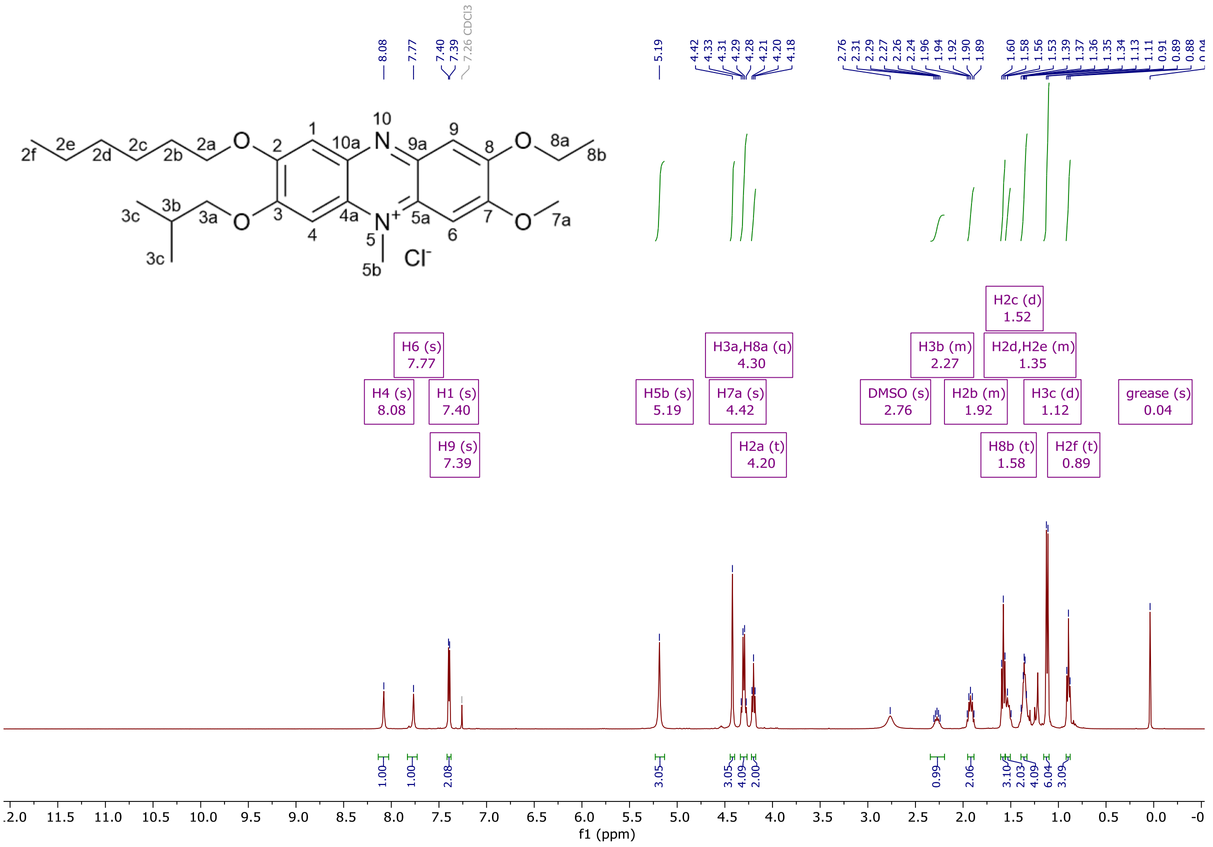

^{1}H NMR (400 MHz, Chloroform-*d*) spectrum of **2a** chloride.

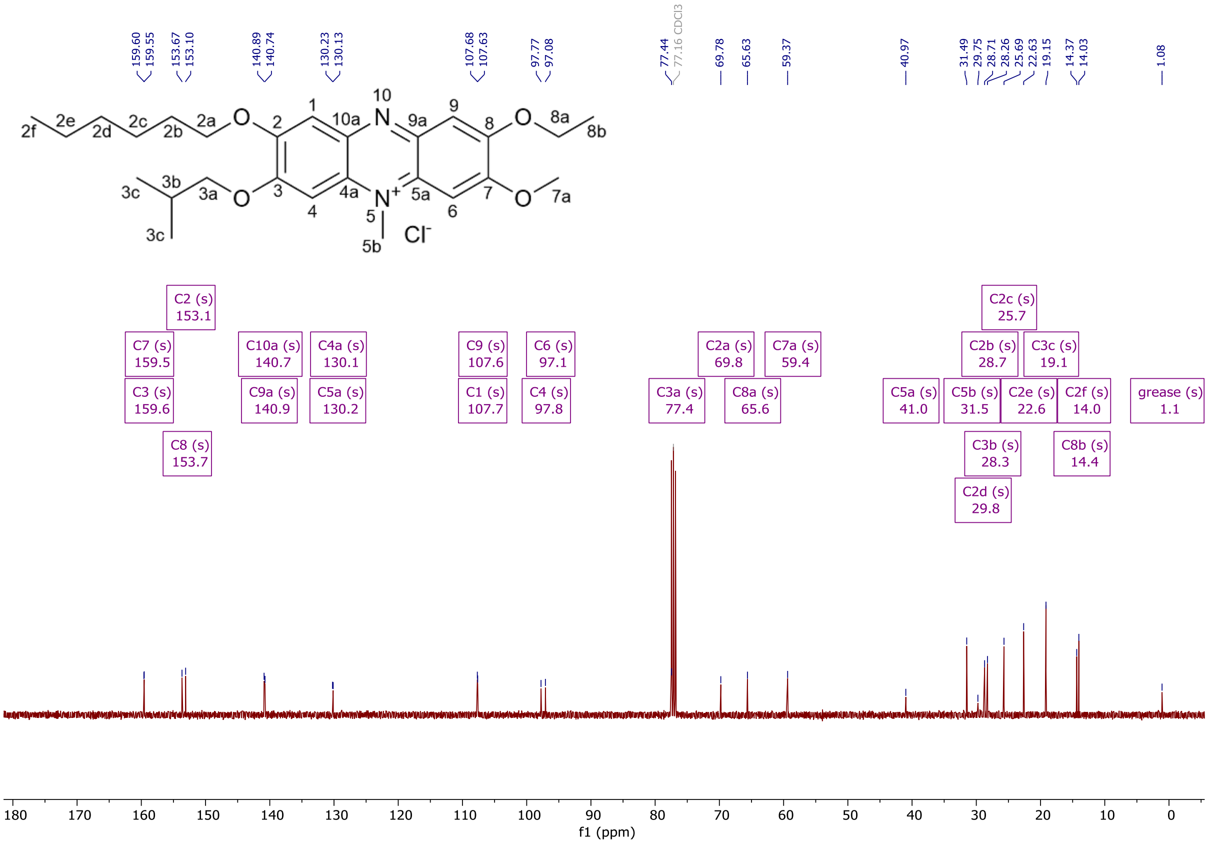

^{13}C{^{1}H} NMR (101 MHz, Chloroform-*d*) spectrum of **2a** chloride.

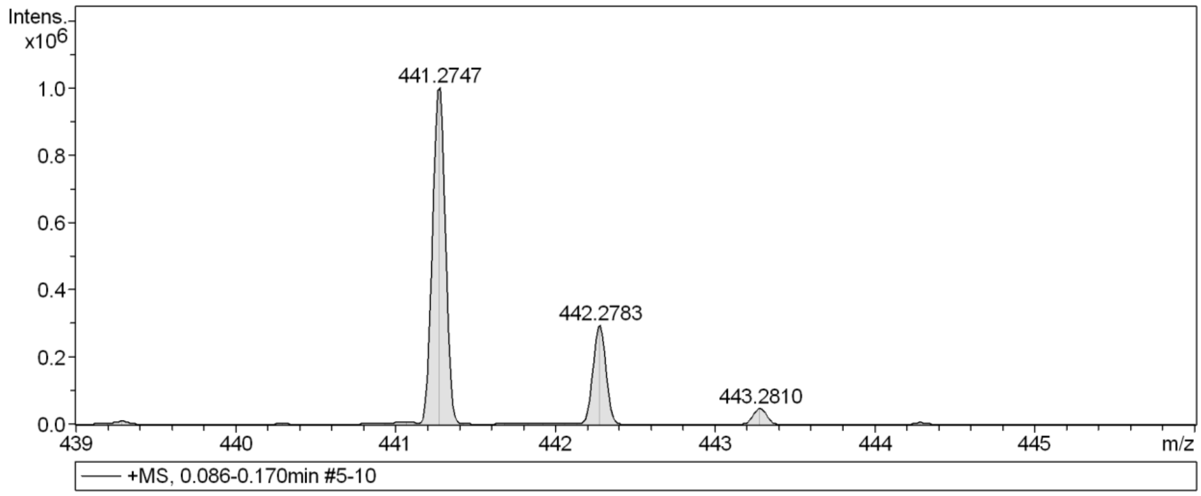

HRMS (ESI+) spectrum of **2a**.

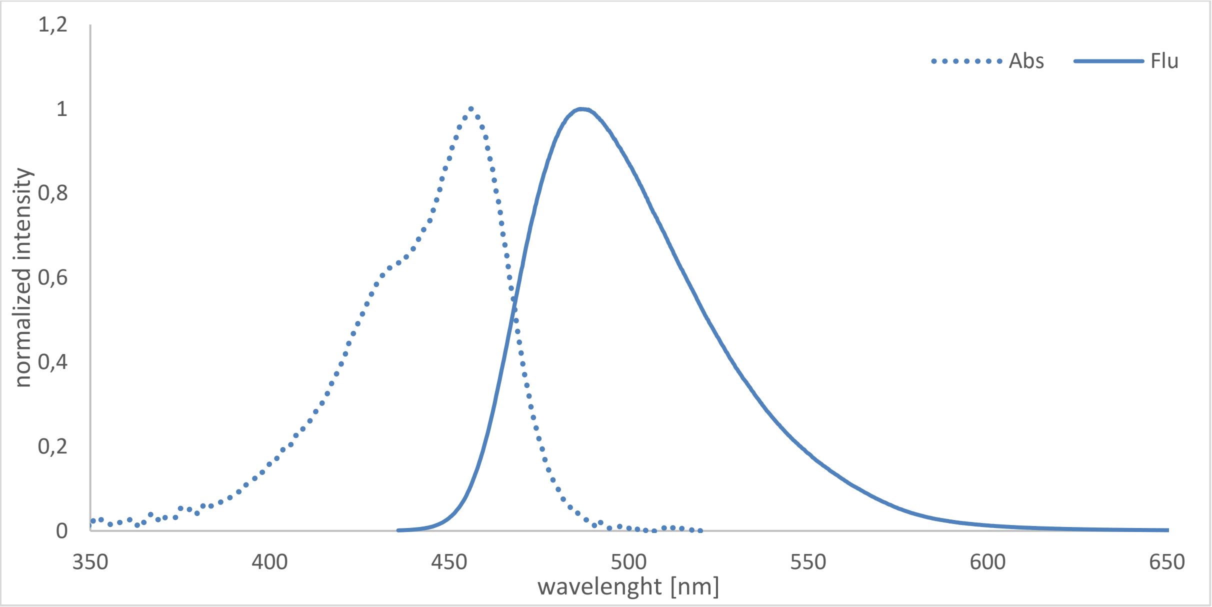

The absorbance and fluorescence (λ_{EX} = 430 nm) spectrum of **2a chloride** in acetonitrile. The compound has Φ = 0.42 in acetonitrile solution.

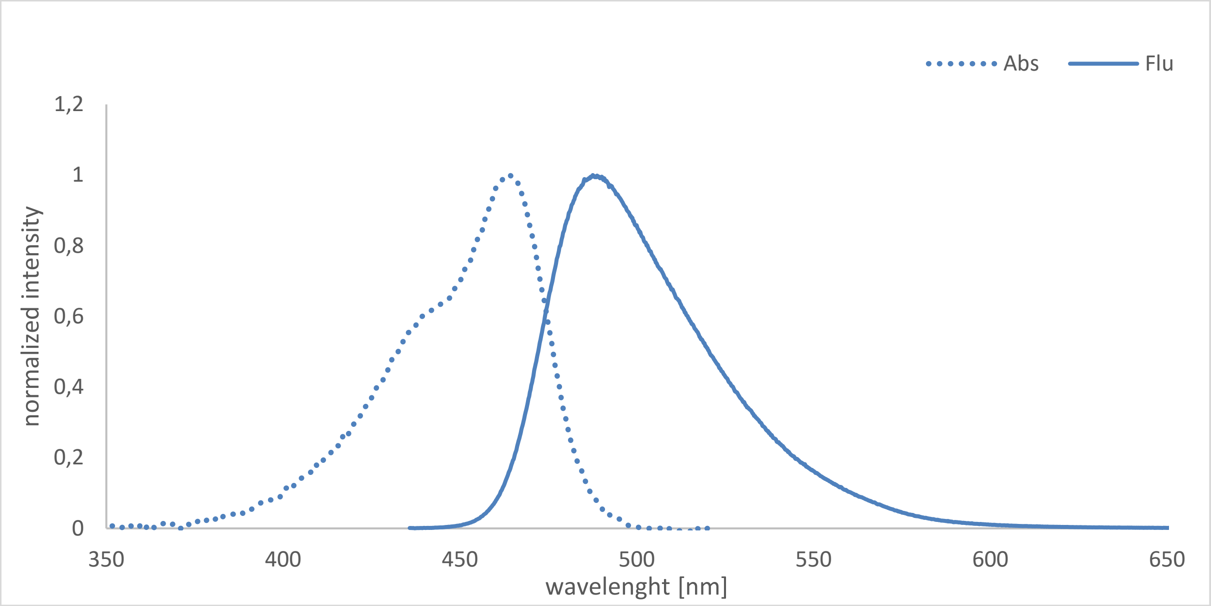

The absorbance and fluorescence (λ_{EX} = 430 nm) spectrum of **2a chloride** in chloroform. The compound has Φ = 0.06 in chloroform solution.

2-ethoxy-8-(hexyloxy)-7-isobutoxy-3-methoxy-5-methylphenazin-5-ium tetrafluoroborate (2a BF_4^-)

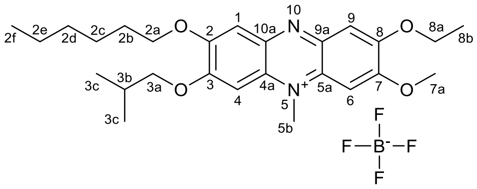

The compound was synthesised from **4a**. Received orange solid. M.p. = 225.0 – 228.0 °C. Yield = 149 mg (94 %, anion exchange of Cl^- to BF_4^-). The total yield of cyclisation and anion exchange is 83 %.

^{1}H NMR (400 MHz, Chloroform-*d*) δ: 7.75 (s, 1H, H_6), 7.58 (s, 1H, H_4), 7.46 (s, 1H, H_9), 7.44 (s, 1H, H_1), 4.83 (s, 3H, H_{5b}), 4.38 – 4.30 (m, 5H, $H_{7a,8a}$), 4.26 – 4.19 (m, 4H, $H_{2a,3a}$), 2.36 – 2.22 (m, 1H, H_{3b}), 2.02 – 1.89 (m, 2H, H_{2b}), 1.61 (t, *J* = 7.0 Hz, 3H, H_{8b}), 1.61 – 1.49 (m, 2H, H_{2c}), 1.45 – 1.33 (m, 4H, $H_{2d,2e}$), 1.14 (d, *J* = 6.7 Hz, 6H, H_{3c}), 0.92 (t, *J* = 7.1 Hz, 2H, H_{2f}).

^{13}C{^{1}H} NMR (101 MHz, Chloroform-*d*) δ: 159.8 (C_3), 159.7 (C_7), 153.8 (C_8), 153.1 (C_2), 141.1 (C_{9a}), 140.8 (C_{10a}), 130.3 (C_{5a}), 130.0 (C_{4a}), 107.9 (C_1), 107.7 (C_9), 96.6 (C_4), 96.4 (C_6), 69.9 (C_{3a}), 65.7 (C_{2a}), 58.5 (C_{8a}), 39.0 (C_{7a}), 31.6 (C_{5b}), 28.8 (C_{2d}), 28.3 (C_{2b}), 25.8 (C_{3b}), 22.7 (C_{2c}), 19.1 (C_{3c}), 14.4 (C_{8b}), 14.1 (C_{2f}).

HRMS (ESI) *m/z* Calculated for $C_{26}H_{37}N_2O_4$ [M]$^+$: 441.2748, found: 441.2744.

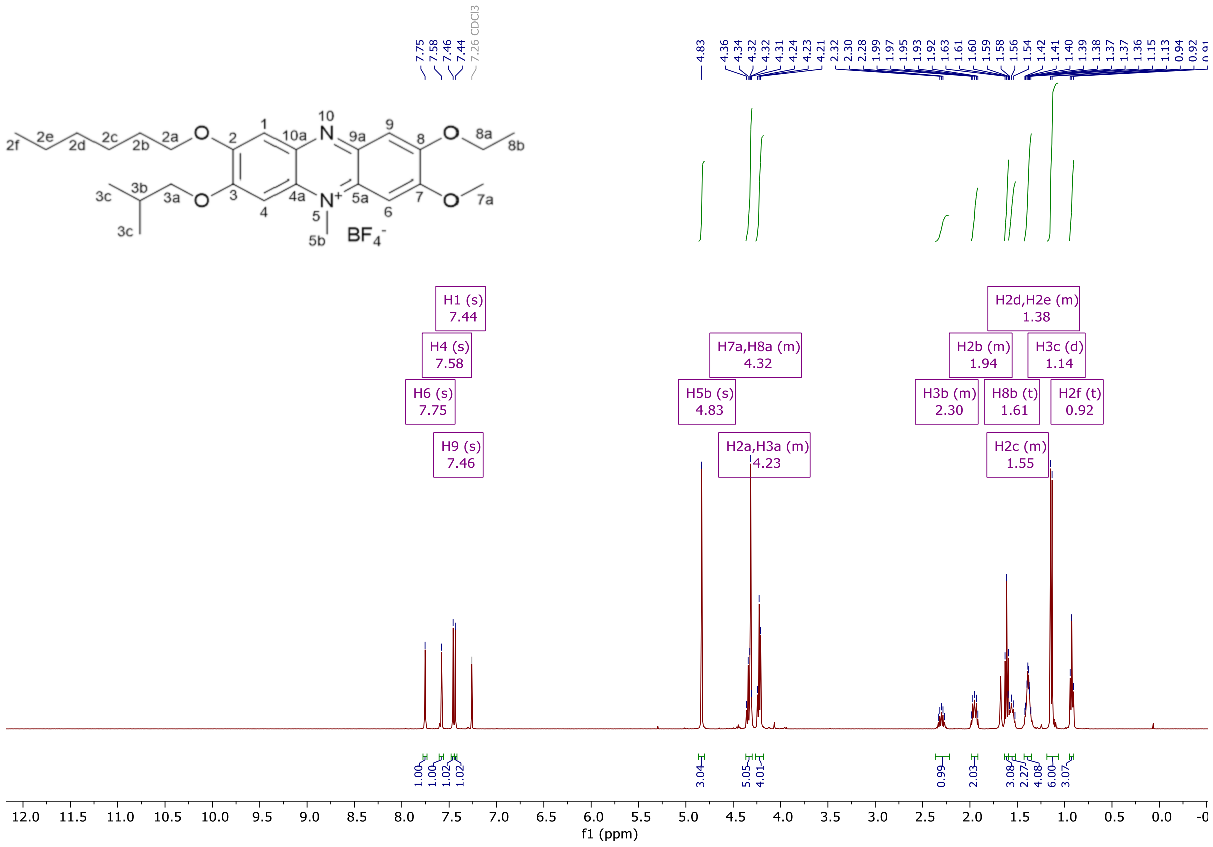

^{1}H NMR (400 MHz, Chloroform-*d*) spectrum of **2a** tetrafluoroborate.

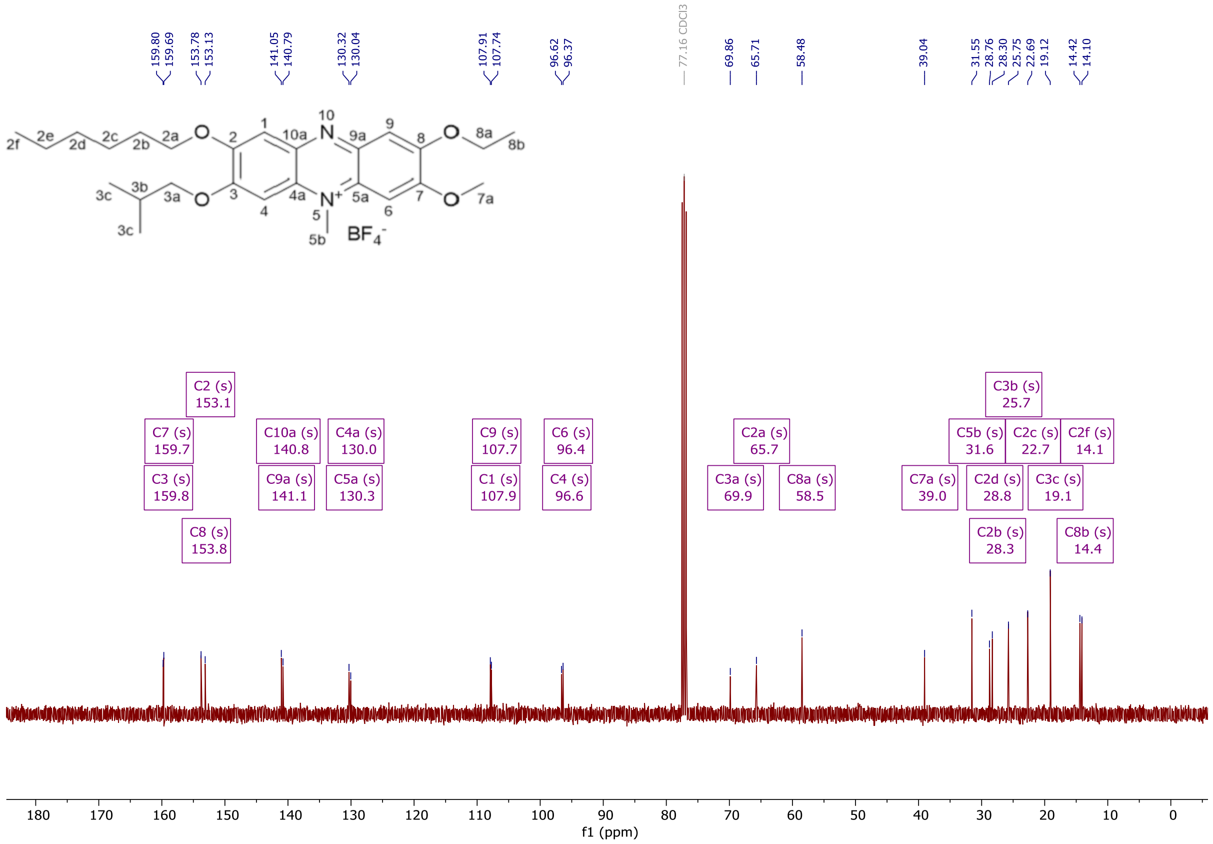

^{13}C{^{1}H} NMR (101 MHz, Chloroform-*d*) spectrum of **2a** tetrafluoroborate.

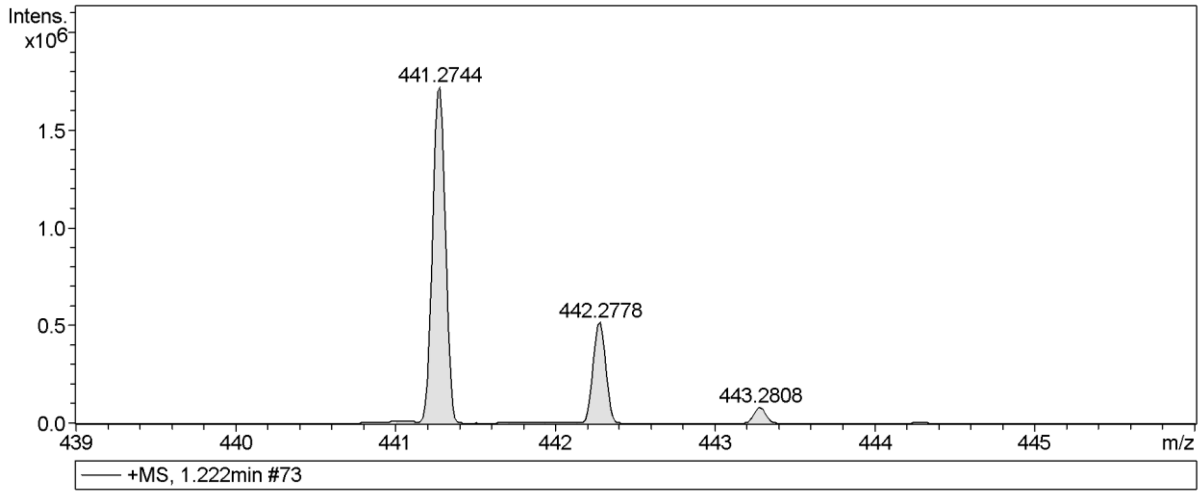

HRMS (ESI+) spectrum of **2a** (tetrafluoroborate).

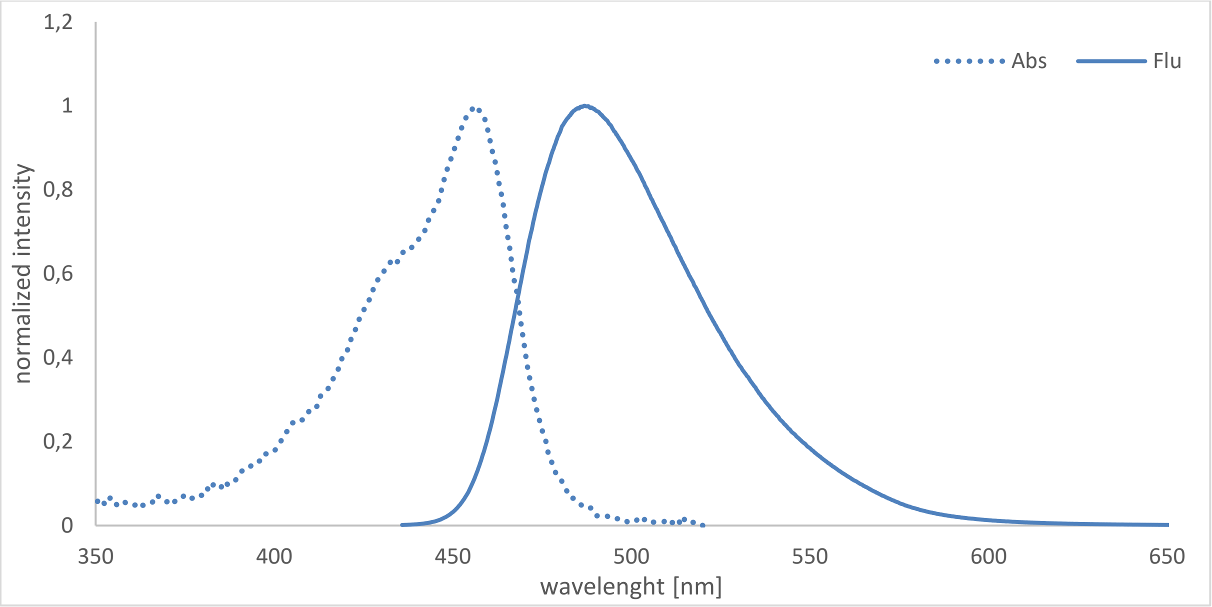

The absorbance and fluorescence (λ_{EX} = 430 nm) spectrum of **2a tetrafluoroborate** in acetonitrile. The compound has Φ = 0.53 in acetonitrile solution.

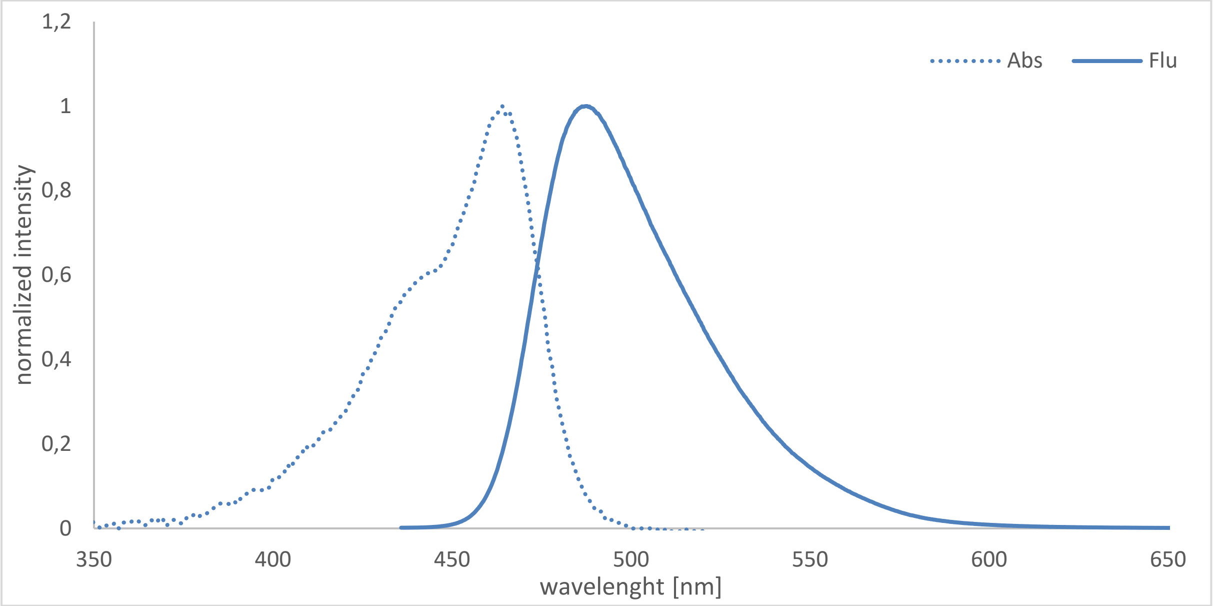

The absorbance and fluorescence (λ_{EX} = 430 nm) spectrum of **2a tetrafluoroborate** in chloroform. The compound has Φ = 0.46 in chloroform solution.

2,3-bis(hexyloxy)-7,8-dimethoxy-5-methylphenazin-5-ium chloride (2b Cl^-)

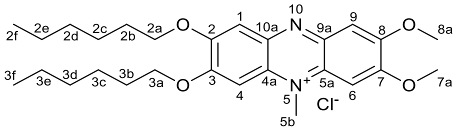

The compound was synthesised from **4b**. Received orange solid. M.p. = 184.2 – 190.0 °C. Yield = 167 mg (85 %). Yield = 804 mg (87 %, gram scale reaction performed on 1.00 g of substrate).

^{1}H NMR (300 MHz, DMSO-*d*$_6$) δ: 7.78 (s, 1H, H_4), 7.75 (s, 1H, H_6), 7.64 (s, 1H, H_1), 7.62 (s, 1H, H_9), 4.74 (s, 3H, H_{5b}), 4.45 (t, *J* = 6.3 Hz, 2H, H_{3a}), 4.30 (t, *J* = 6.2 Hz, 2H, H_{2a}), 4.23 (s, 3H, H_{7a}), 4.09 (s, 3H, H_{8a}), 1.95 – 1.81 (m, 4H, $H_{2b,3b}$), 1.58 – 1.47 (m, 4H, $H_{2c,3c}$), 1.40 – 1.32 (m, 8H, $H_{2d,2e,3d,3e}$), 0.95 – 0.86 (m, 6H, $H_{2f,3f}$).

^{13}C{^{1}H} NMR (75 MHz, DMSO-*d*$_6$) δ: 158.1 (C_3), 158.0 (C_7), 153.0 (C_2), 152.6 (C_8), 140.6 (C_{9a}), 140.3 (C_{10a}), 129.9 (C_{5a}), 129.7 (C_{4a}), 107.6 (C_9), 107.3 (C_1), 97.4 (C_6), 97.1 (C_4), 70.5 (C_{2a}), 69.4 (C_{3a}), 57.9 (C_{8a}), 56.8 (C_{7a}), 38.9 (C_{5b}), 30.9 (C_{3d}), 30.9 (C_{2d}), 28.2 (C_{3b}), 28.1 (C_{2b}), 25.2 (C_{3c}), 25.1 (C_{2c}), 22.1 ($C_{2e,3e}$), 13.8 ($C_{2f,3f}$).

^{1}H NMR (400 MHz, Chloroform-*d*) δ: 8.05 (s, 1H, H_4), 7.69 (s, 1H, H_6), 7.51 (s, 1H, H_1), 7.48 (s, 1H, H_9), 5.14 (s, 3H, H_{5b}), 4.50 (t, *J* = 6.4 Hz, 2H, H_{3a}), 4.44 (s, 3H, H_{7a}), 4.25 (t, *J* = 6.5 Hz, 2H, H_{2a}), 4.13 (s, 3H, H_{3a}), 2.05 – 1.92 (m, 4H, $H_{2b,3b}$), 1.43 – 1.37 (m, 8H, $H_{2d,2e,2d,2e}$), 0.96 – 0.89 (m, 6H, $H_{2f,2f}$). Signals from 2c and 3c are missing – overlap with water signal – 1.56 ppm.

HRMS (ESI) *m/z* Calculated for $C_{27}H_{39}N_2O_4$ $[M]^+$: 455.2904, found: 455.2900.

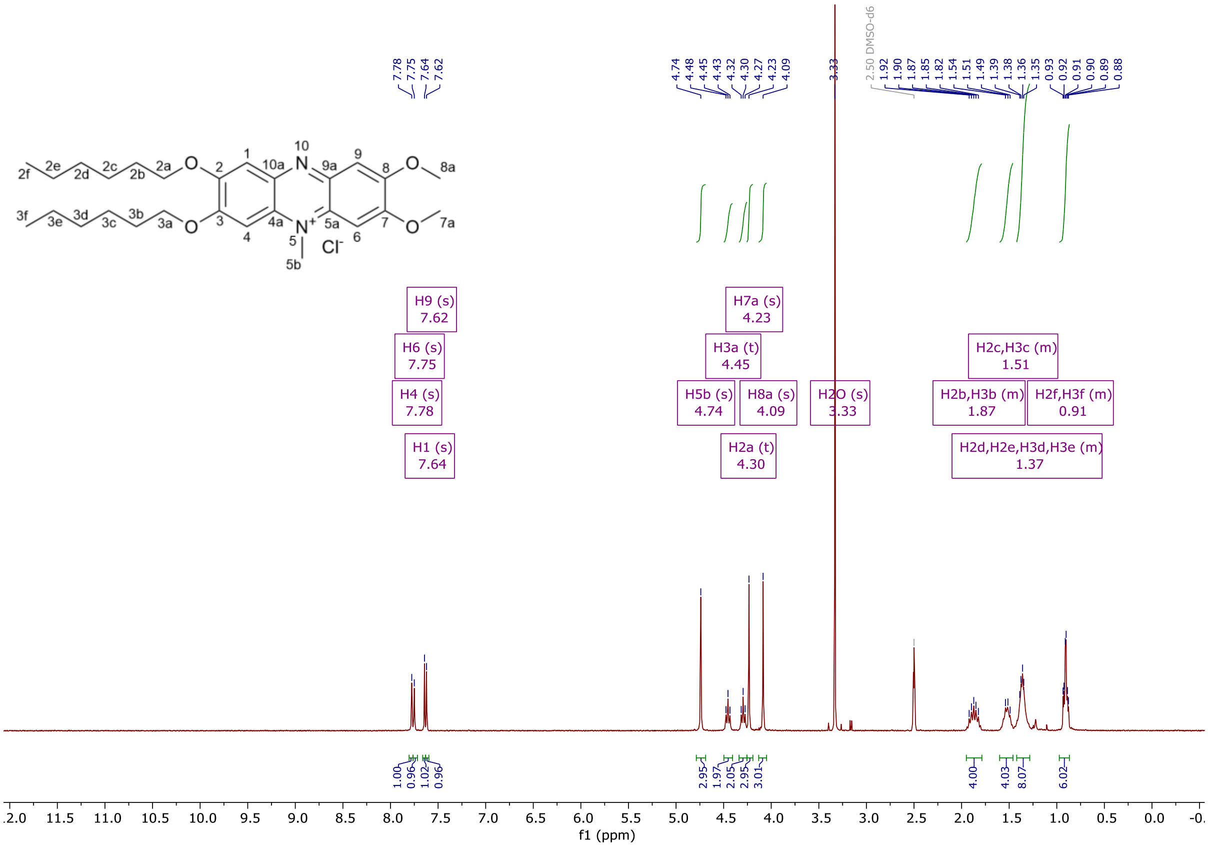

^{1}H NMR (300 MHz, DMSO-d_6) spectrum of **2b** chloride.

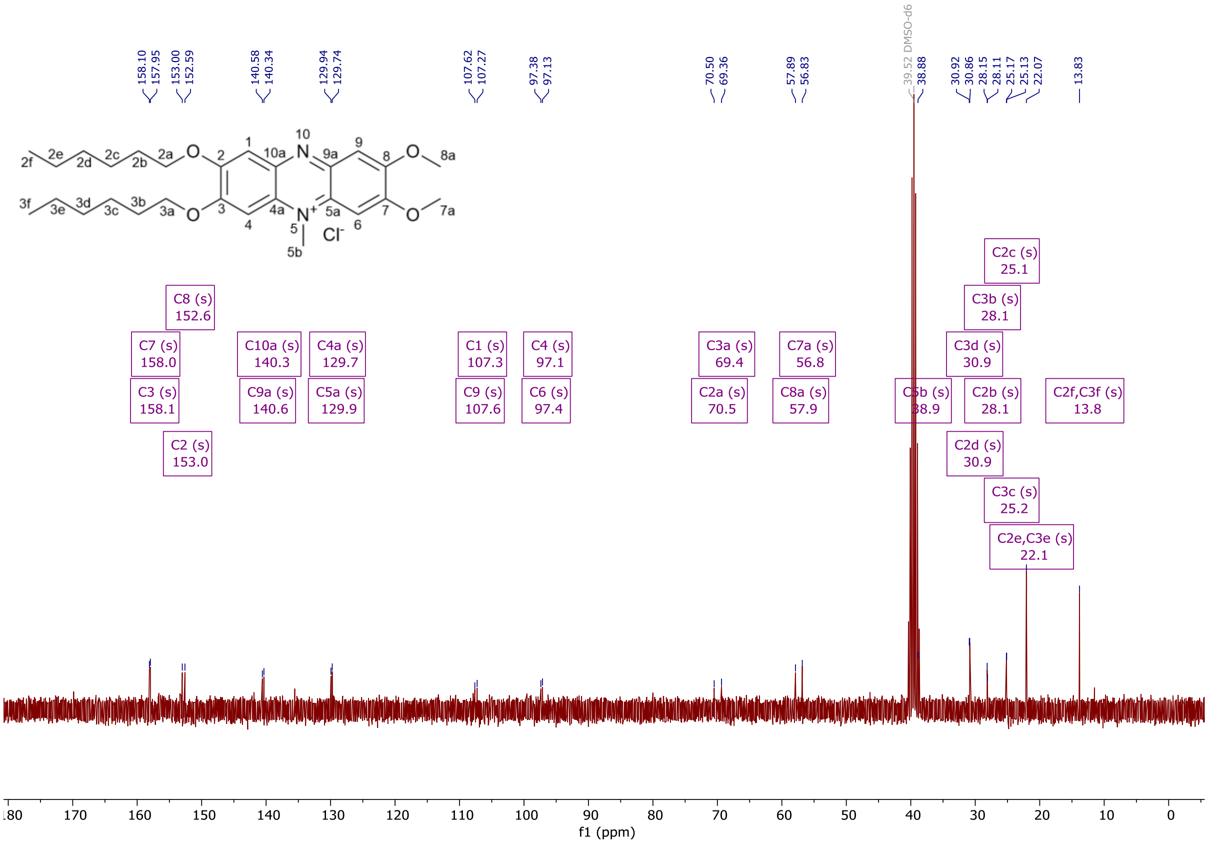

^{13}C{^{1}H} NMR (75 MHz, DMSO-d_6) spectrum of **2b** chloride.

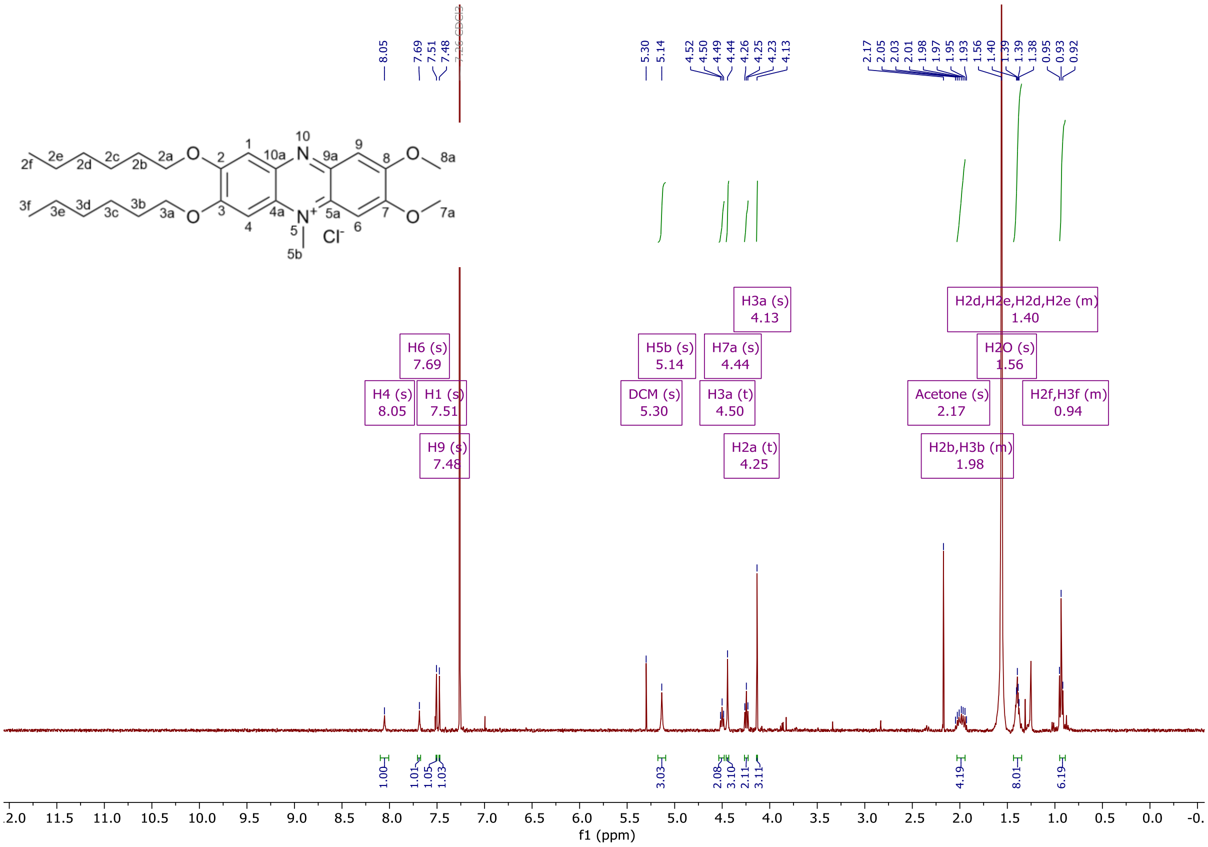

^{1}H NMR (400 MHz, Chloroform-*d*) spectrum of **2b** chloride.

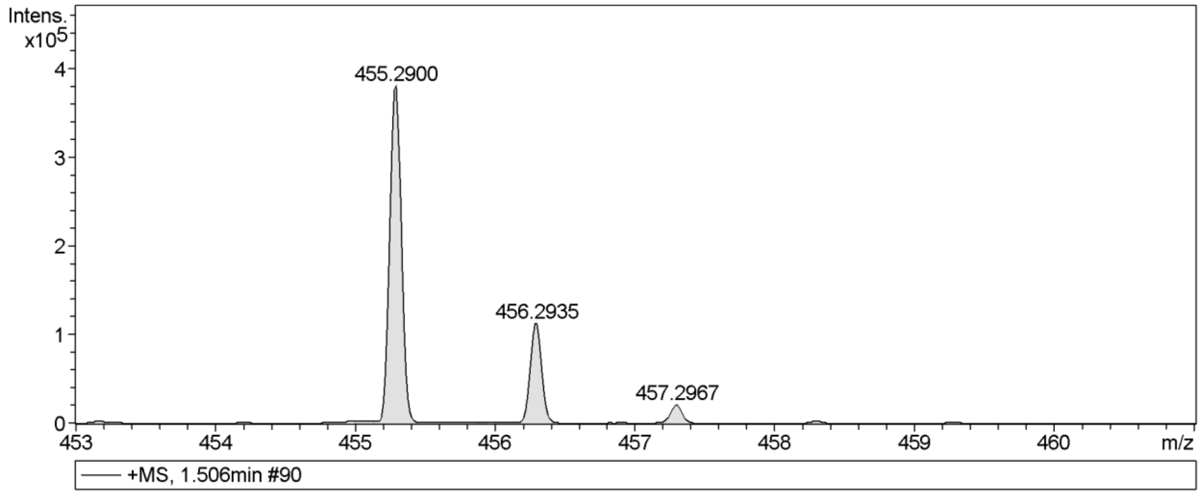

HRMS (ESI+) spectrum of **2a** (chloride).

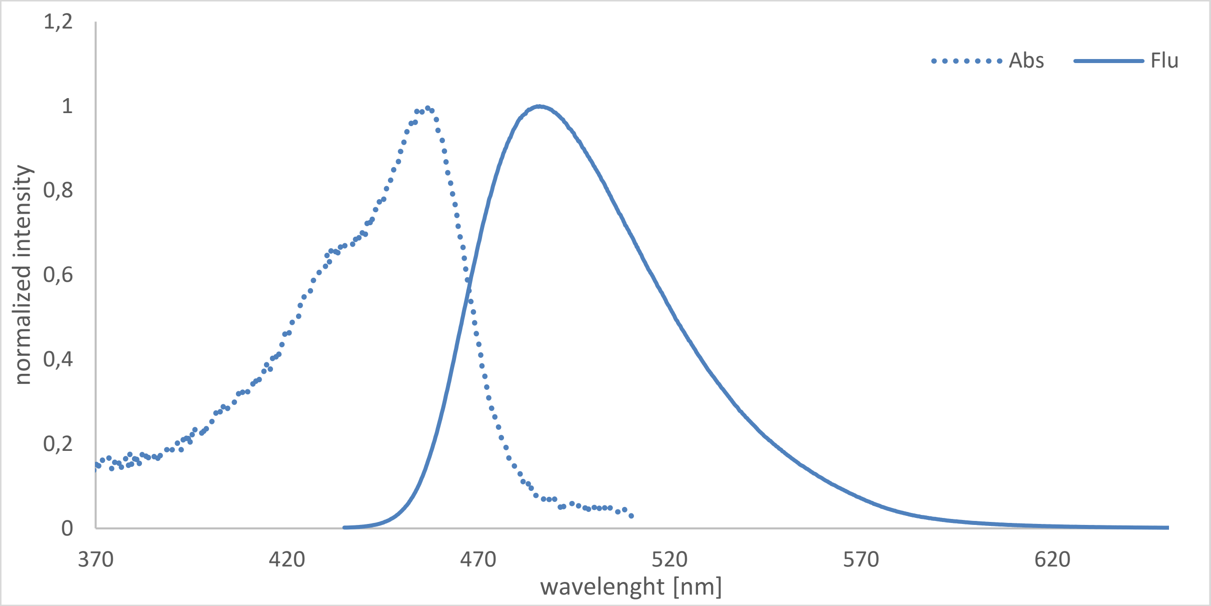

The absorbance and fluorescence (λ_{EX} = 430 nm) spectrum of **2b chloride** in acetonitrile. The compound has Φ = 0.42 in acetonitrile solution.

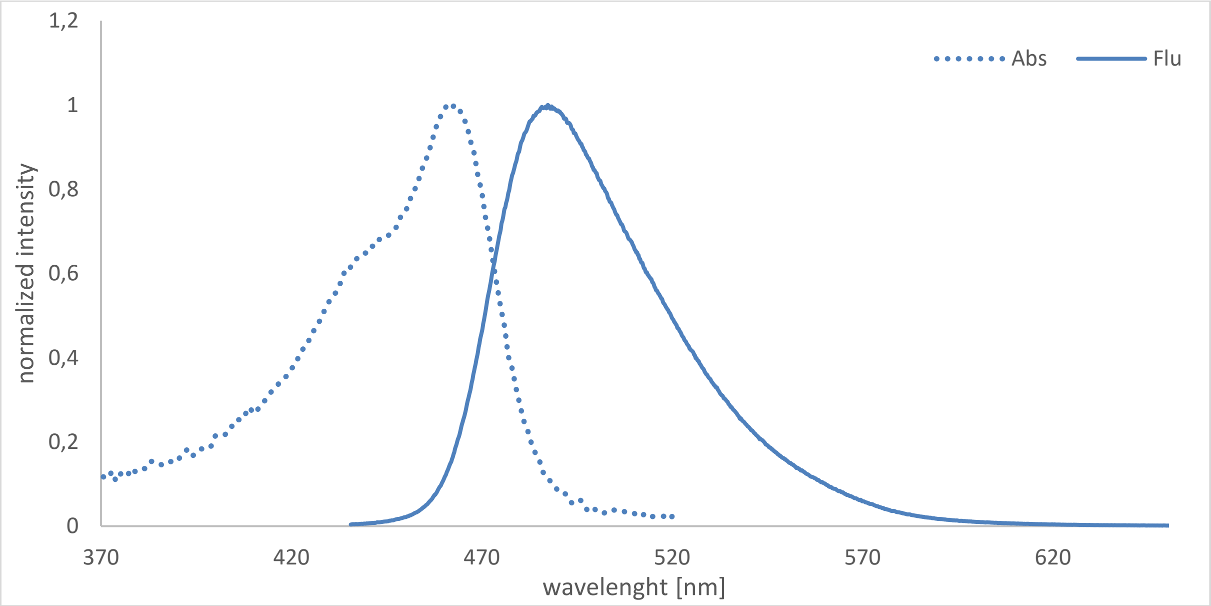

The absorbance and fluorescence (λ_{EX} = 430 nm) spectrum of **2b chloride** in chloroform. The compound has Φ = 0.06 in chloroform solution.

2,3-bis(hexyloxy)-7,8-dimethoxy-5-methylphenazin-5-ium tetrafluoroborate (2b BF_4^-)

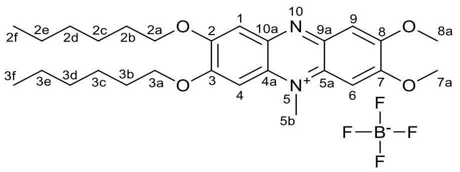

The compound was synthesised from **4b**. Received orange solid. M.p. = 222.0 – 228.5 °C. Yield = 158 mg, (97 %, anion exchange of Cl^- to BF_4^-). The total yield of cyclisation and anion exchange is 83 %.

^{1}H NMR (400 MHz, Chloroform-*d*) δ: 7.78 (s, 1H, H_4), 7.59 (s, 1H, H_6), 7.51 (s, 1H, H_1), 7.47 (s, 1H, H_9), 4.85 (s, 3H, H_{5b}), 4.45 (t, *J* = 6.3 Hz, 2H, H_{3a}), 4.34 (s, 3H, H_{7a}), 4.24 (t, *J* = 6.4 Hz, 2H, H_{2a}), 4.13 (s, 3H, H_{8a}), 2.06 – 1.90 (m, 4H, $H_{2b,3b}$), 1.64 – 1.50 (m, 4H, $H_{2c,3c}$), 1.43 – 1.35 (m, 8H, $H_{2d,2e,3d,3e}$), 0.98 – 0.88 (m, 6H, $H_{2f,3f}$).

^{13}C{^{1}H} NMR (151 MHz, Chloroform-*d*) δ: 160.0 (C_3), 159.6 (C_7), 153.88 (C_2), 153.86 (C_8), 141.2 (C_{9a}), 140.8 (C_{10a}), 130.4 (C_{5a}), 130.2 (C_{4a}), 107.9 (C_9), 107.6 (C_1), 96.7 (C_6), 96.3 (C_4), 71.7 (C_{3a}), 70.1 (C_{2a}), 58.7 (C_{7a}), 57.0 (C_{8a}), 31.6 (C_{5b}), 30.3 (C_{3d}), 29.8 (C_{2d}), 28.8 (C_{3b}), 28.7 (C_{2b}), 25.8 (C_{3c}), 25.7 (C_{2c}), 22.7 (C_{3e}), 22.7 (C_{2e}), 14.1 ($C_{2f,3f}$).

HRMS (ESI) *m/z* Calculated for $C_{27}H_{39}N_2O_4$ $[M]^+$: 455.2904, found: 455.2902.

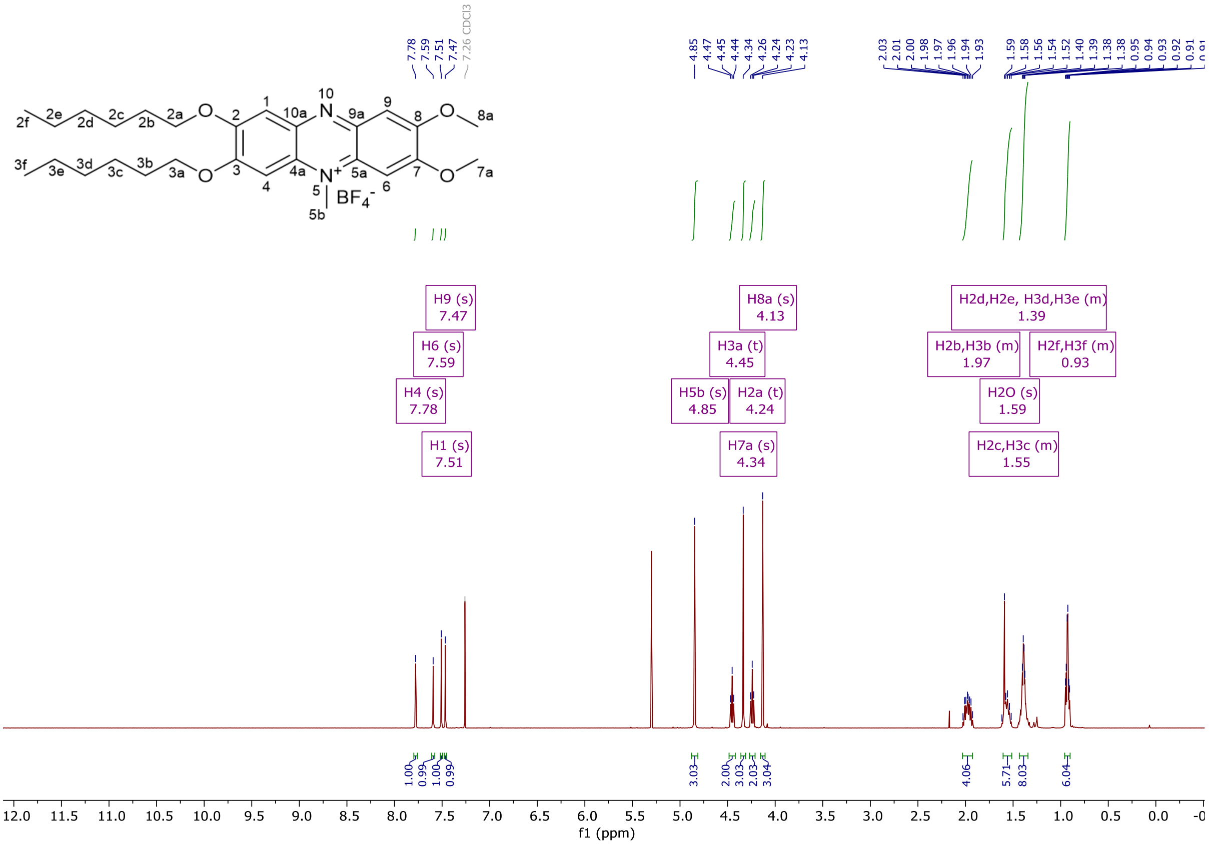

^{1}H NMR (400 MHz, Chloroform-*d*) spectrum of **2b** tetrafluoroborate.

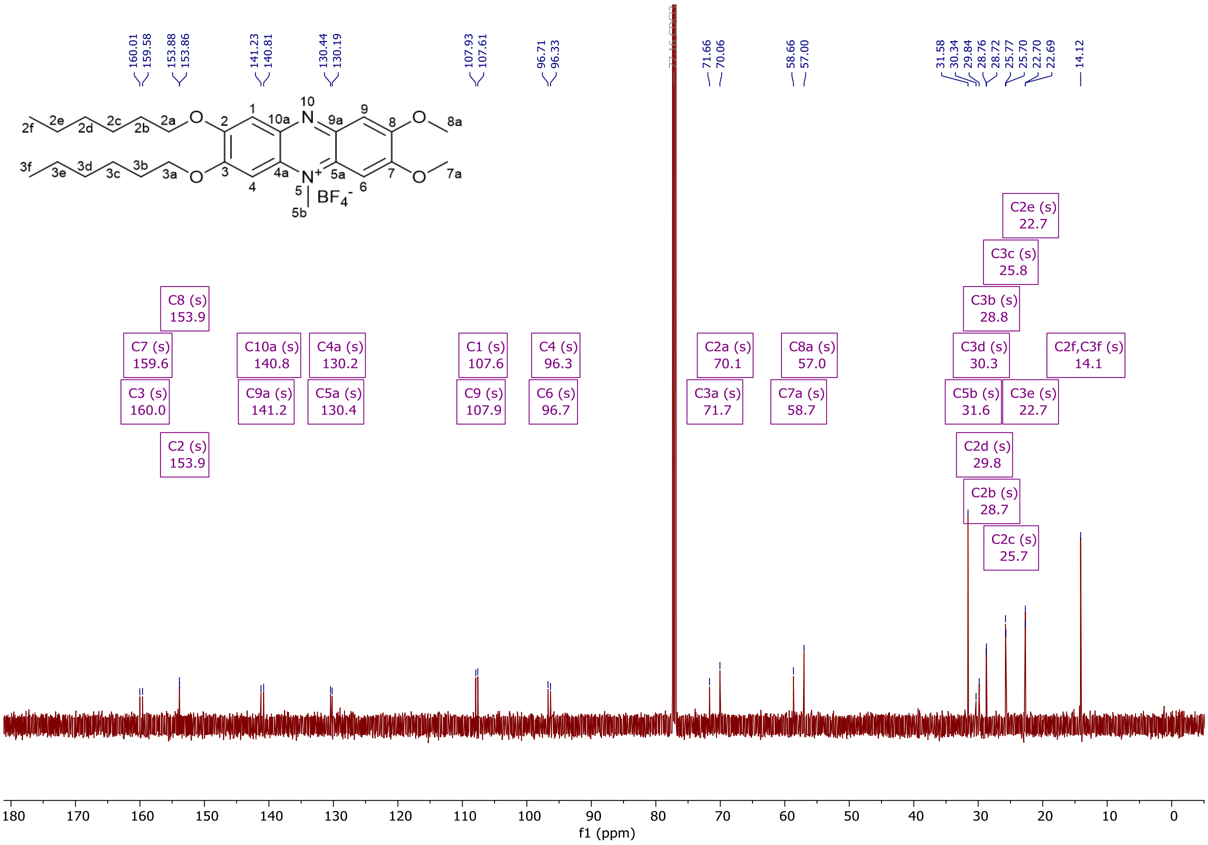

$^{13}C\{^1H\}$ NMR (151 MHz, Chloroform-*d*) spectrum of **2b** tetrafluoroborate.

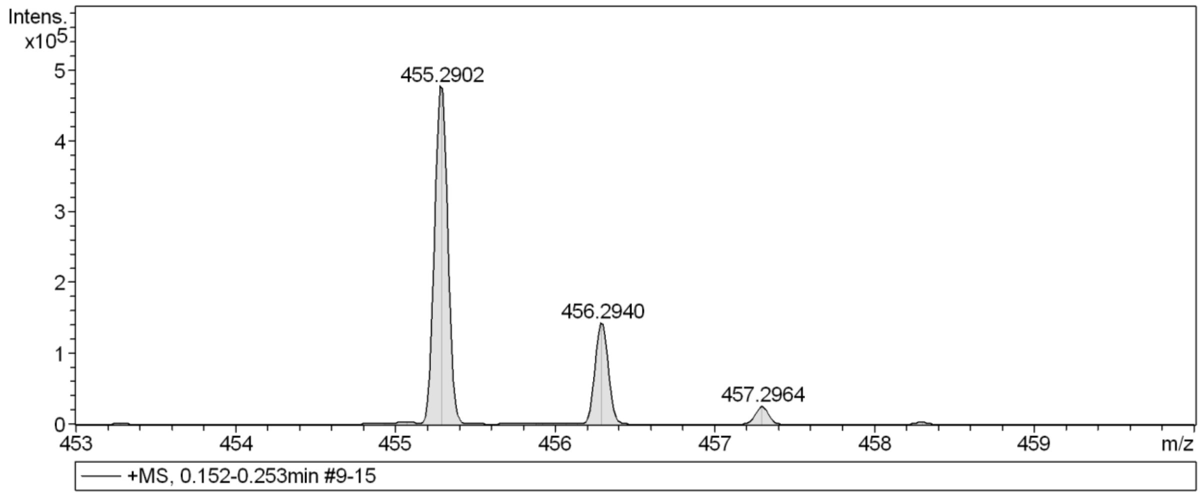

HRMS (ESI+) spectrum of **2b**.

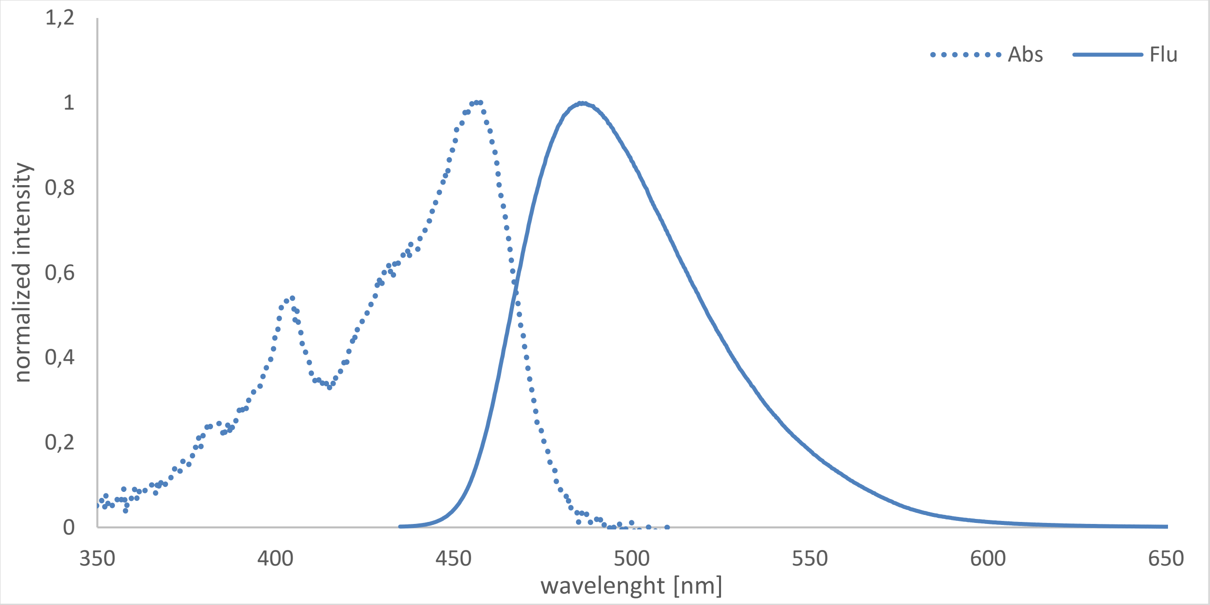

The absorbance and fluorescence (λ_{EX} = 430 nm) spectrum of **2b tetrafluoroborate** in acetonitrile. The compound has Φ = 0.53 in acetonitrile solution.

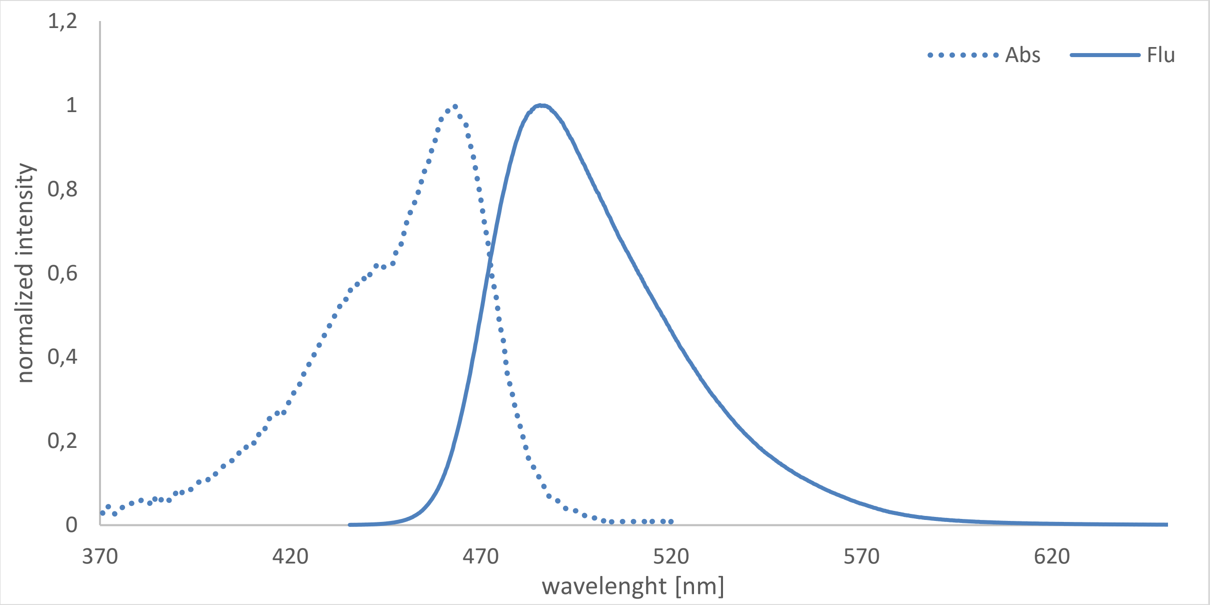

The absorbance and fluorescence (λ_{EX} = 430 nm) spectrum of **2b tetrafluoroborate** in chloroform. The compound has Φ = 0.45 in chloroform solution.

5-hexyl-2,3-bis(hexyloxy)-7,8-dimethoxyphenazin-5-ium tetrafluoroborate (2c)

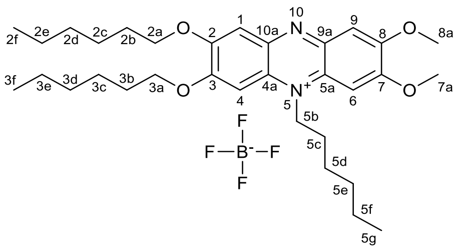

The compound was synthesised from **4c**. Received orange solid. M.p. = 215.0 – 224.5 °C. Yield = 188 mg (84 %, Cl^- salt), Yield = 177 mg (96 %, anion exchange of Cl^- to BF_4^-). The total yield of cyclisation and anion exchange is 81 %.

^{1}H NMR (400 MHz, Chloroform-*d*) δ: 8.07 (s, 1H, H_4), 7.69 (s, 1H, H_6), 7.43 (s, 1H, H_1), 7.40 (s, 1H, H_9), 5.92 (s, 2H, H_{5b}), 4.49 (t, *J* = 6.4 Hz, 2H, H_{3a}), 4.42 (s, 3H, H_{7a}), 4.20 (t, *J* = 6.4 Hz, 2H, H_{2a}), 4.08 (s, 3H, H_{8a}), 2.06 – 2.00 (m, 2H, H_{5c}), 1.93 (m, 4H, $H_{2b,3b}$), 1.65 – 1.58 (m, 2H, H_{5d}), 1.59 – 1.46 (m, 4H, $H_{2c,3c}$), 1.40 – 1.22 (m, 12H, $H_{2d,2e,3d,3e,5e,5f}$), 0.88 (t, *J* = 6.8 Hz, 6H, $H_{2f,3f}$), 0.79 (t, *J* = 7.1 Hz, 3H, H_{5g}).

^{13}C{^{1}H} NMR (101 MHz, Chloroform-*d*) δ: 159.8 (C_3), 159.5 (C_7), 153.8 (C_2), 153.7 (C_8), 141.4 (C_{9a}), 141.0 (C_{10a}), 129.5 (C_{5a}), 129.4 (C_{4a}), 108.1 (C_9), 107.8 (C_1), 96.5 (C_4), 96.2 (C_6), 71.4 (C_{2a}), 70.0 (C_{3a}), 58.4 (C_{8a}), 57.0 (C_{7a}), 50.6 (C_{5b}), 31.7 (C_{5e}), 31.59 (C_{3d}), 31.57 (C_{2d}), 29.8 (C_{5c}), 28.7 (C_{3b}), 28.7 (C_{2b}), 26.4 (C_{5d}), 25.8 (C_{3c}), 25.7 (C_{2c}), 22.69(C_{3e}), 22.67 (C_{2e}), 22.5 (C_{5f}), 14.3 (C_{5g}), 14.11 (C_{3f}), 14.05 (C_{2f}).

HRMS (ESI) *m/z* Calculated for $C_{32}H_{49}N_2O_4$ $[M]^+$: 525.3687, found: 525.3685.

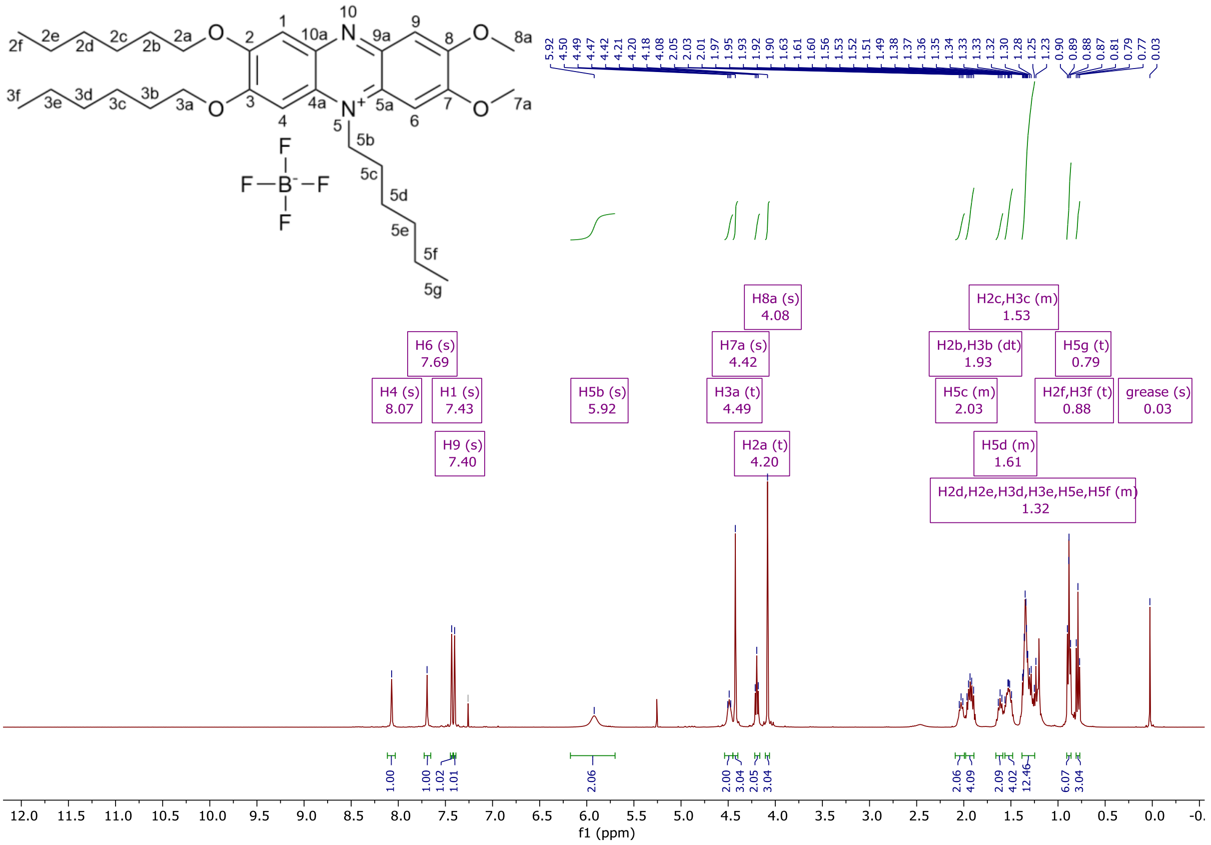

^{1}H NMR (400 MHz, Chloroform-*d*) spectrum of **2c** tetrafluoroborate.

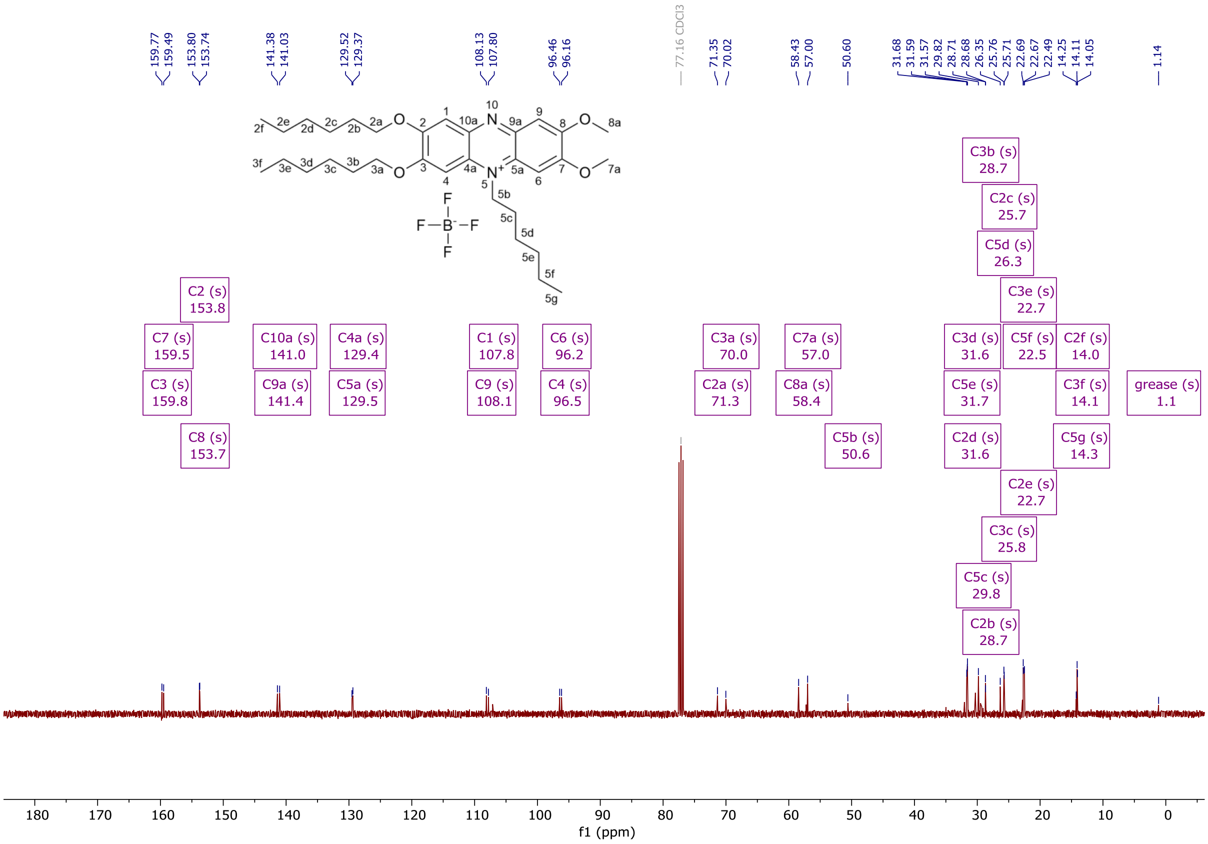

$^{13}C\{^{1}H\}$ NMR (101 MHz, Chloroform-*d*) spectrum of **2c** tetrafluoroborate.

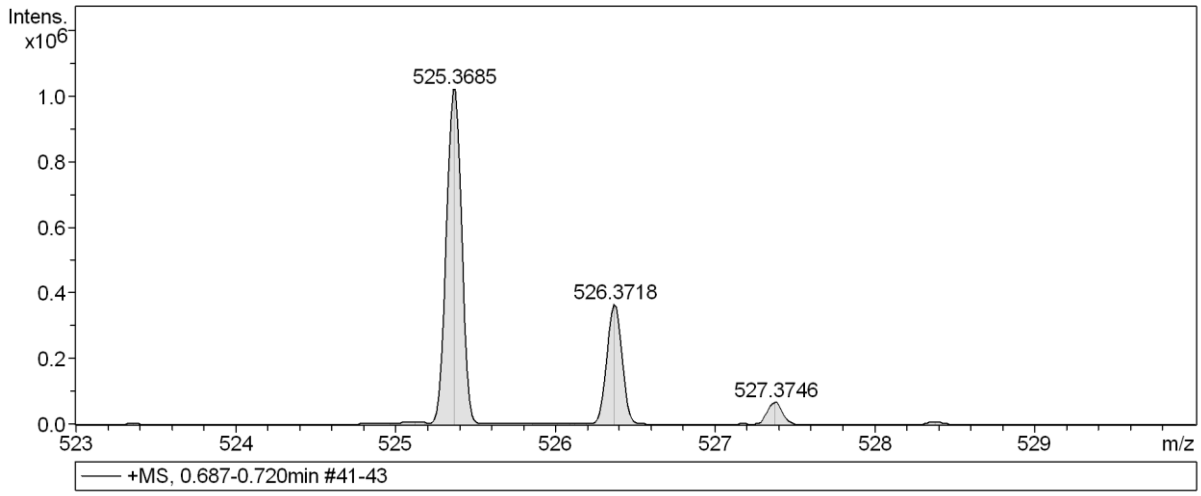

HRMS (ESI+) spectrum of **2c**.

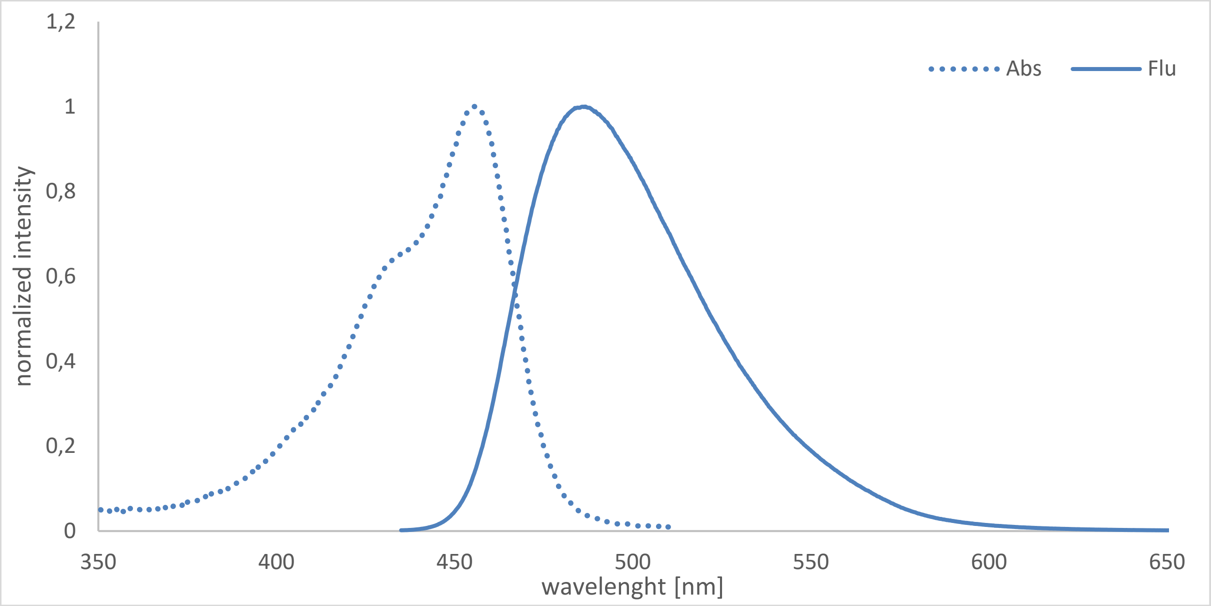

The absorbance and fluorescence (λ_{EX} = 430 nm) spectrum of **2c tetrafluoroborate** in acetonitrile. The compound has Φ = 0.26 in acetonitrile solution.

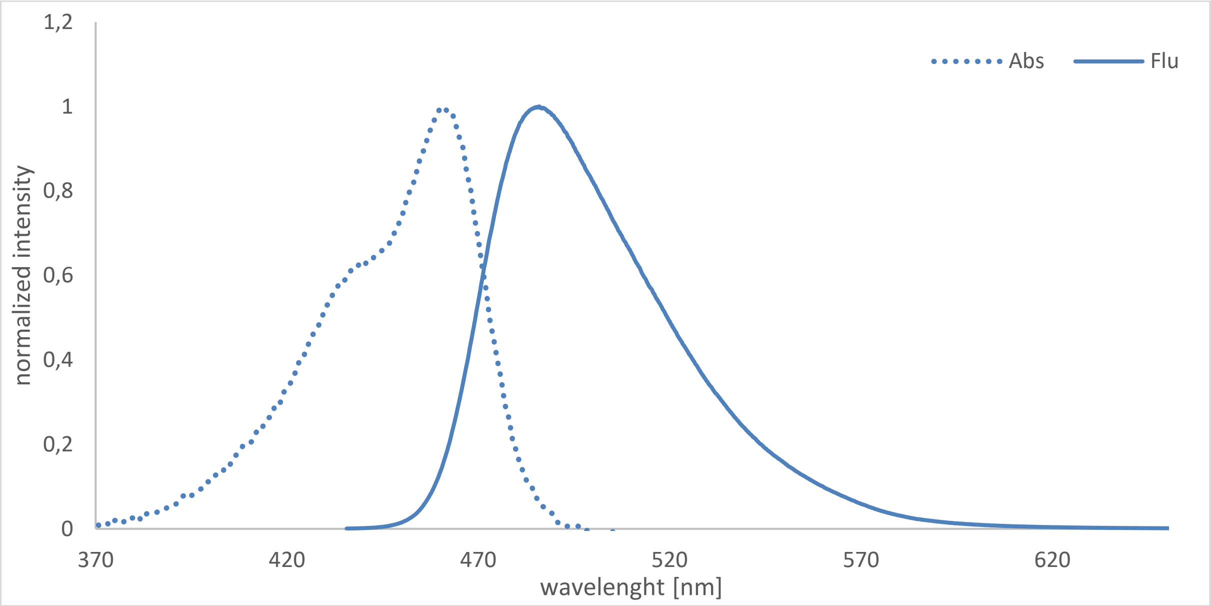

The absorbance and fluorescence (λ_{EX} = 430 nm) spectrum of **2c tetrafluoroborate** in chloroform. The compound has Φ = 0.43 in chloroform solution.

2-(hexyloxy)-3-isobutoxy-5-methyl-8-(trifluoromethyl)phenazin-5-ium tetrafluoroborate (2d)

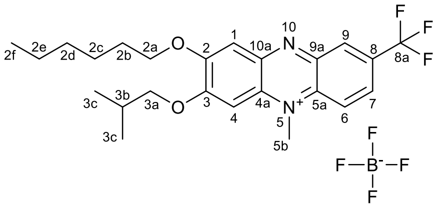

The compound was synthesised from **4d**. Received pale orange solid. M.p. = 174.4 – 177.4 °C. Yield = 149 mg (79 %, Cl^- salt), Yield = 147 mg (94%, anion exchange of Cl^- to BF_4^-). The total yield of cyclisation and anion exchange is 74 %.

^{1}H NMR (400 MHz, Chloroform-*d*) δ: 8.72 (d, *J* = 9.4 Hz, 1H, H_6), 8.67 (d, *J* = 2.1 Hz, 1H, H_9), 8.31 (dd, *J* = 9.4, 2.1 Hz, 1H, H_7), 7.82 (s, 1H, H_4), 7.51 (s, 1H, H_1), 4.94 (s, 3H, H_{5b}), 4.43 (d, *J* = 6.3 Hz, 2H, H_{3a}), 4.30 (t, *J* = 6.3 Hz, 2H, H_{2a}), 2.37 – 2.28 (m, 1H, H_{3b}), 2.05 – 1.92 (m, 2H, H_{2b}), 1.59 (d, *J* = 6.9 Hz, 2H, H_{2c}), 1.46 – 1.34 (m, 3H, $H_{2d,2e}$), 1.15 (d, *J* = 6.6 Hz, 6H, H_{3c}), 0.93 (t, *J* = 6.9 Hz, 3H, H_{2f}).

^{13}C{^{1}H} NMR (101 MHz, Chloroform-*d*) δ: 165.3 (C_3), 156.0 (C_2), 146.1 (C_{9a}), 140.6 ($C_{10a,5a}$), 135.3 (C_{4a}), 131.7 (m, C_8), 130.9 – 130.7 (m, C_7), 129.2 – 129.0 (m, C_9), 127.0 (m, C_{8a}), 119.8 (C_6), 107.6 (C_1), 96.8 (C_4), 78.6 (C_{3a}), 70.6 (C_{2a}), 39.4 (C_{5b}), 31.5 (C_{2d}), 28.6 (C_{2b}), 28.2 (C_{3b}), 25.7 (C_{2c}), 22.7 (C_{2e}), 19.0 (C_{3c}), 14.1 (C_{2f}).

^{19}F NMR (282 MHz, Chloroform-*d*) δ: -65.96 (s, 3F, F_{CF3}), -152.81 – -156.30 (m, 4F, F_{BF4-}). [relative chemical shift – no reference was used]

^{11}B NMR (128 MHz, Chloroform-*d*) δ: -2.01 (s, B_{BF4-}). [relative chemical shift – no reference was used]

HRMS (ESI) *m/z* Calculated for $C_{24}H_{30}F_3N_2O_2$ $[M]^+$: 435.2254, found: 435.2254.

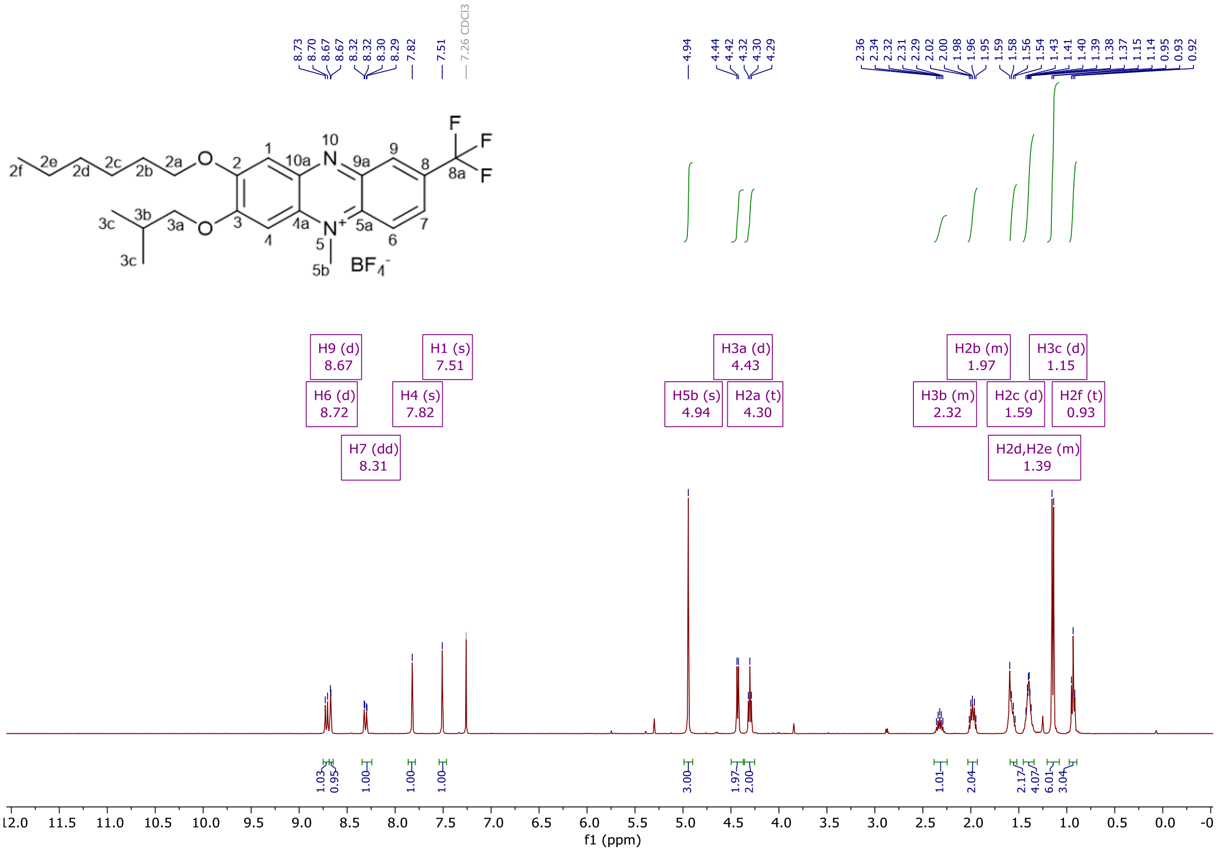

^{1}H NMR (400 MHz, Chloroform-*d*) spectrum of **2d** tetrafluoroborate.

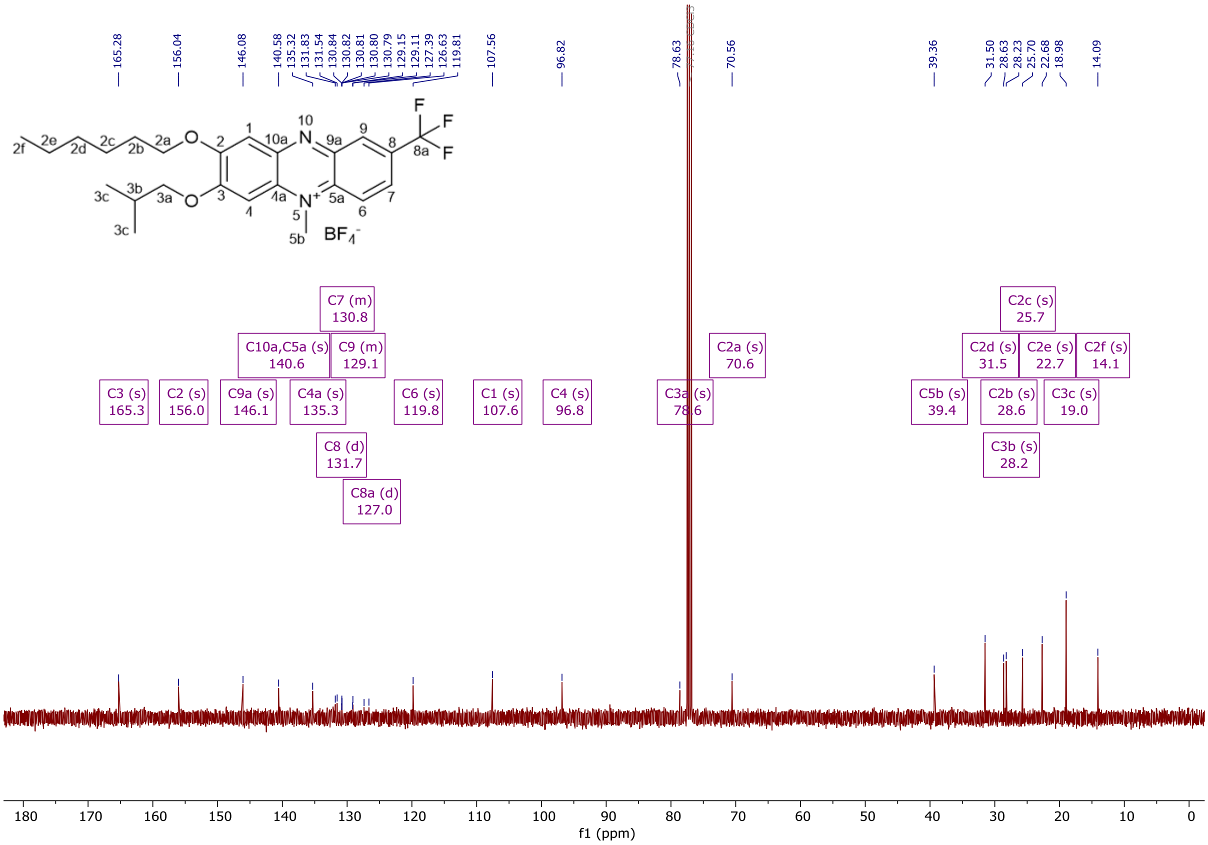

^{13}C{^{1}H} NMR (101 MHz, Chloroform-*d*) spectrum of **2d** tetrafluoroborate.

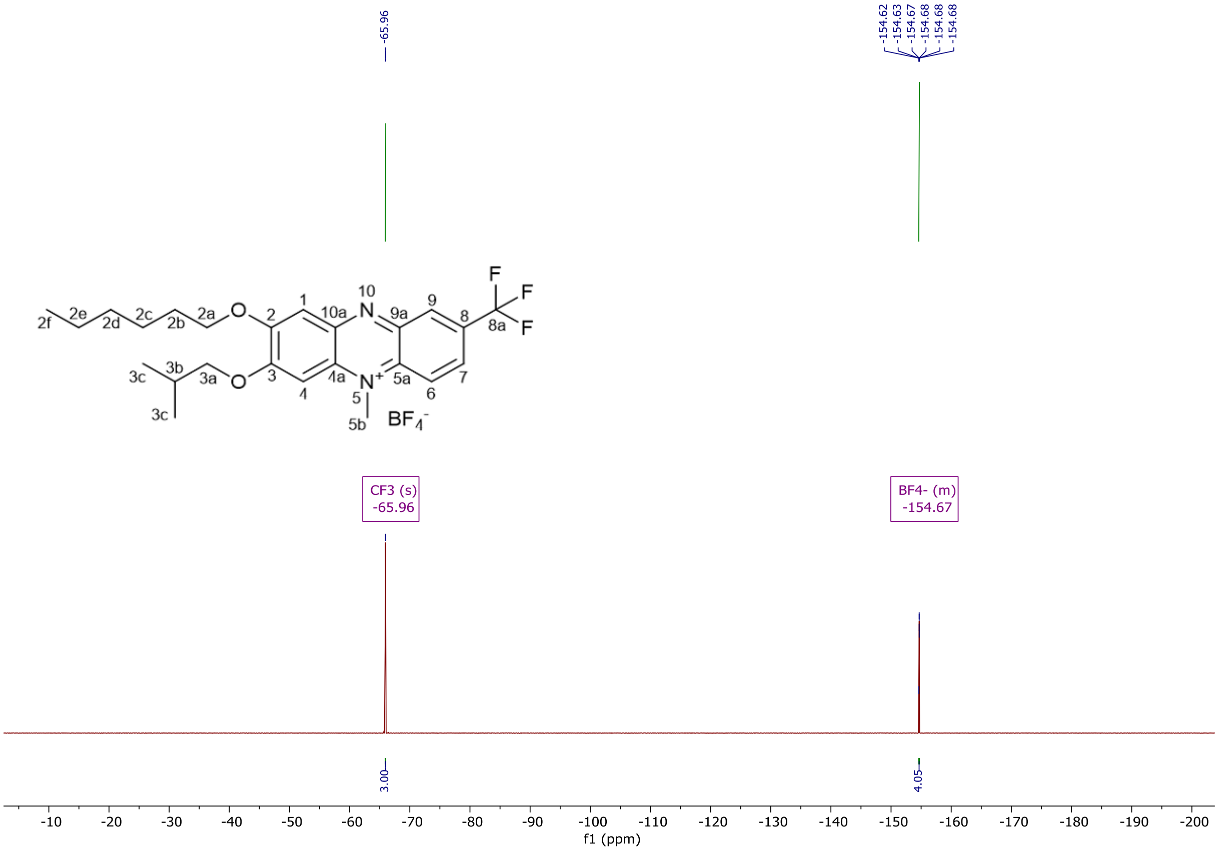

^{19}F NMR (282 MHz, Chloroform-*d*) spectrum of **2d** tetrafluoroborate.

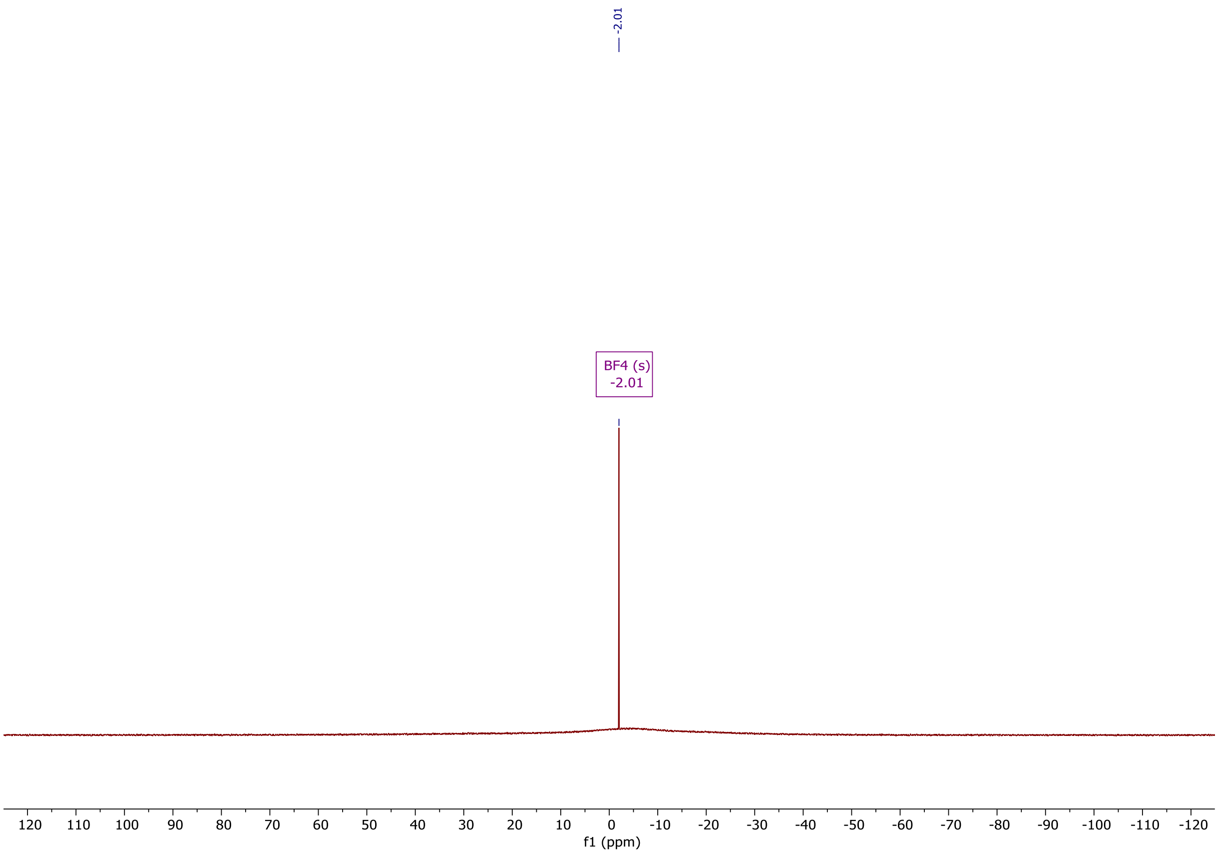

^{11}B NMR (128 MHz, Chloroform-*d*) spectrum of **2d** tetrafluoroborate.

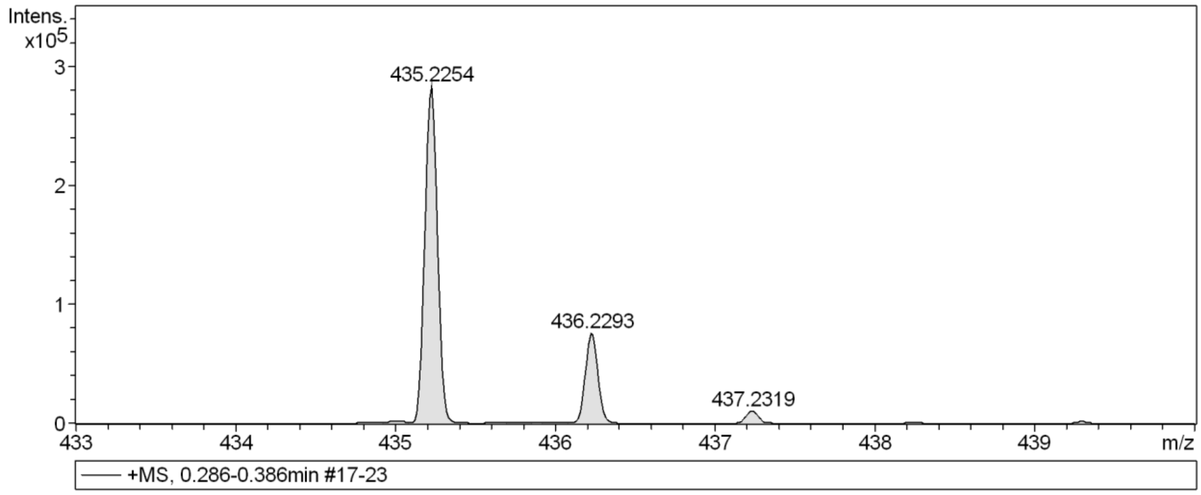

HRMS (ESI+) spectrum of **2d**.

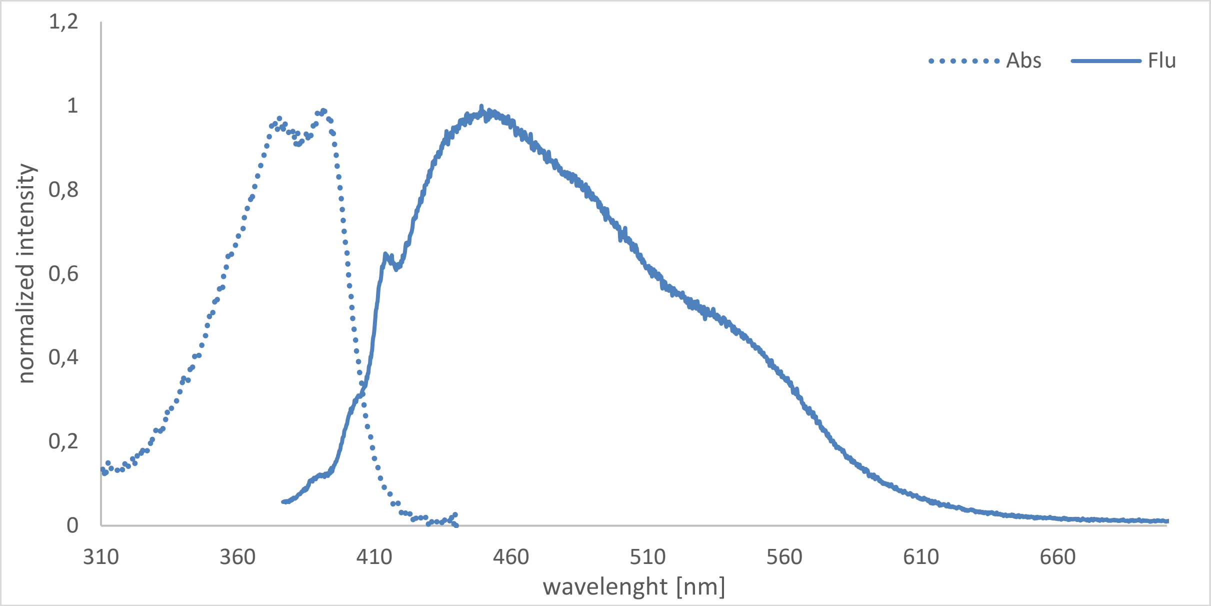

The absorbance and fluorescence (λ_{EX} = 370 nm) spectrum of **2d tetrafluoroborate** in acetonitrile. The compound has Φ = 0.008 in acetonitrile solution.

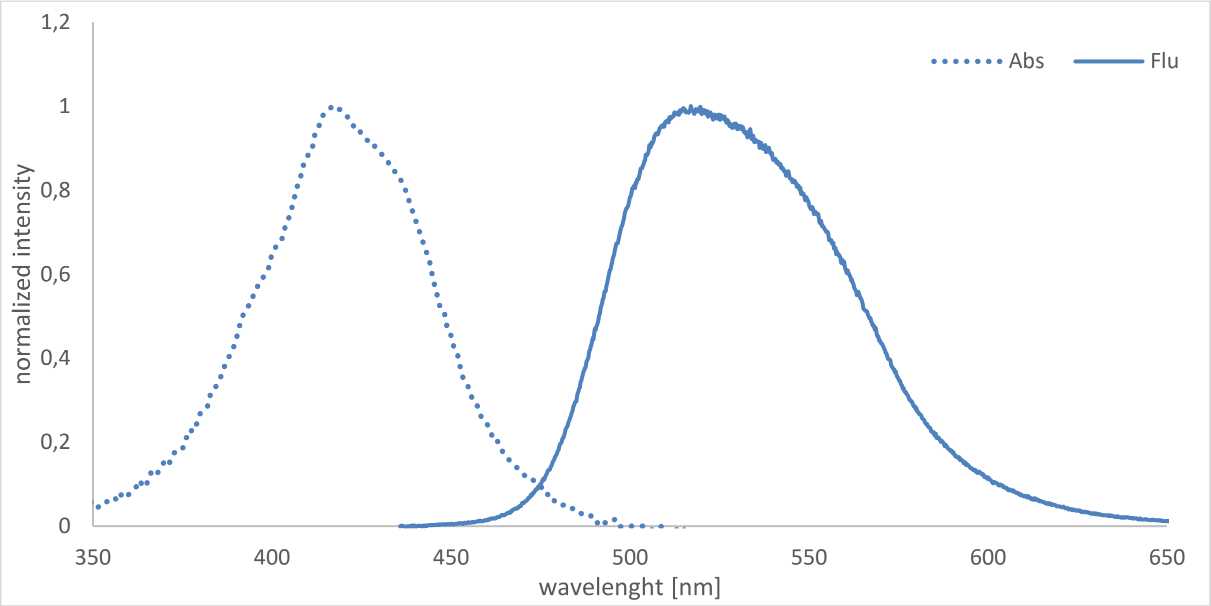

The absorbance and fluorescence (λ_{EX} = 430 nm) spectrum of **2d tetrafluoroborate** in chloroform. The compound has Φ = 0.013 in chloroform solution.

7-(*tert*-butyl)-2-(hexyloxy)-3-isobutoxy-5-methylphenazin-5-ium tetrafluoroborate (2e)

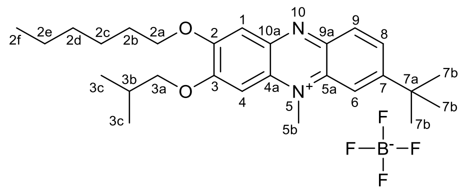

The compound was synthesised from **4e**. Received orange solid. M.p. = 104.4 – 106.8 °C. Yield = 159 mg (87 %, Cl^- salt), Yield = 174 mg (96 %, anion exchange of Cl^- to BF_4^-). The total yield of cyclisation and anion exchange is 84 %.

^{1}H NMR (400 MHz, Chloroform-*d*) δ: 8.34 (d, *J* = 9.0 Hz, 1H, H_9), 8.20 (d, *J* = 1.8 Hz, 1H, H_6), 8.10 (dd, *J* = 9.0, 1.8 Hz, 1H, H_8), 7.85 (s, 1H, H_1), 7.50 (s, 1H, H_4), 4.90 (s, 3H, H_{5b}), 4.41 (d, *J* = 6.2 Hz, 2H, H_{3a}), 4.27 (t, *J* = 6.3 Hz, 2H, H_{2a}), 2.39 – 2.27 (m, 1H, H_{3b}), 2.04 – 1.91 (m, 2H, H_{2b}), 1.59 – 1.55 (m, 2H, H_{2c}), 1.53 (s, 9H, H_{7b}), 1.45 – 1.33 (m, 4H, $H_{2d,H2e}$), 1.16 (d, *J* = 6.7 Hz, 6H, H_{3c}), 0.98 – 0.88 (m, 3H, H_{2f}).

^{13}C{^{1}H} NMR (101 MHz, Chloroform-*d*) δ: 163.3 (C_3), 161.2 (C_7), 155.0 (C_2), 144.0 (C_{9a}), 140.8 (C_{5a}), 133.7 (C_{10a}), 131.1 (C_8), 130.5 (C_{4a}), 129.7 (C_9), 112.6 (C_6), 107.6 (C_1), 96.7 (C_4), 78.0 (C_{3a}), 70.2 (C_{2a}), 38.8 (C_{5b}), 37.0 (C_{7a}), 31.5 (C_{2d}), 30.9 (C_{7b}), 28.7 (C_{2b}), 28.3 (C_{3b}), 25.7 (C_{2c}), 22.7 (C_{2e}), 19.0 (C_{3c}), 14.1 (C_{2f}).

HRMS (ESI) *m/z* Calculated for $C_{27}H_{39}N_2O_2$ $[M]^+$: 423.3006, found: 423.3003.

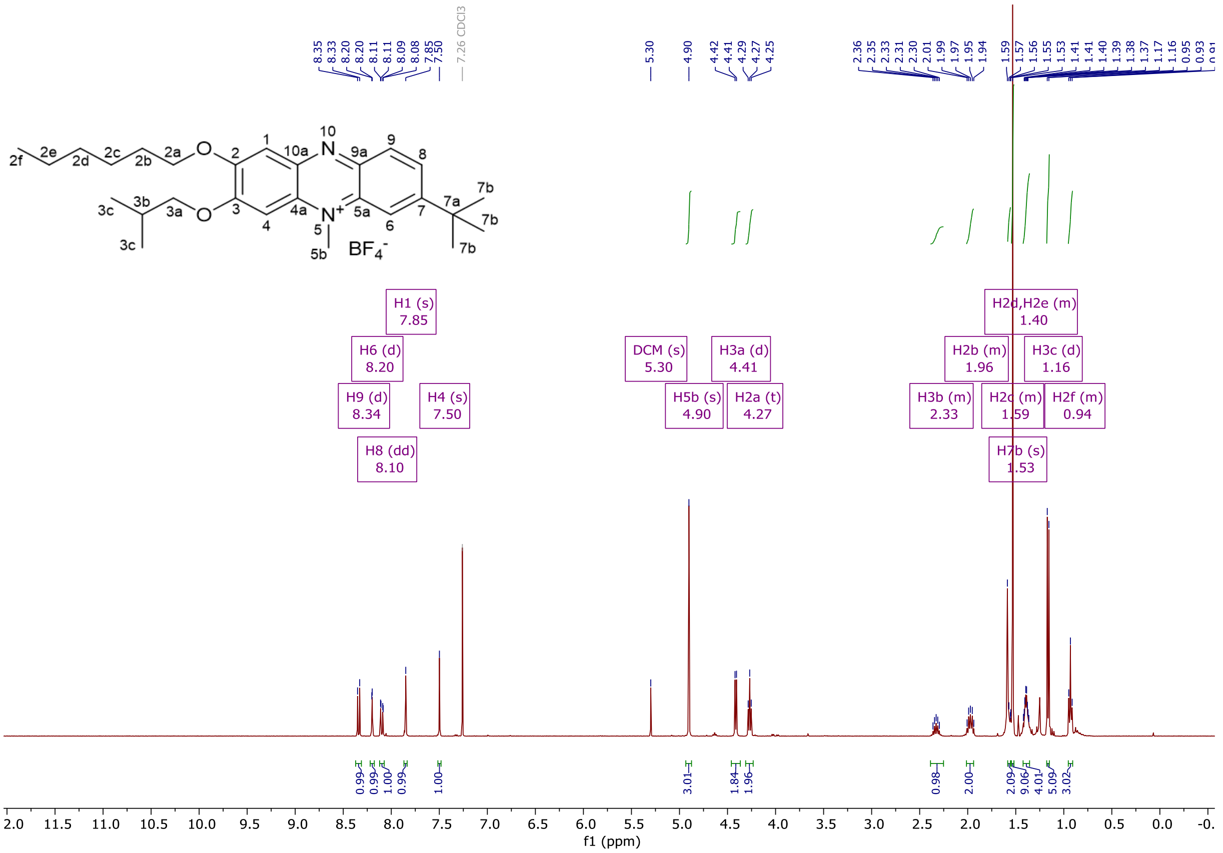

^{1}H NMR (400 MHz, Chloroform-*d*) spectrum of **2e** tetrafluoroborate.

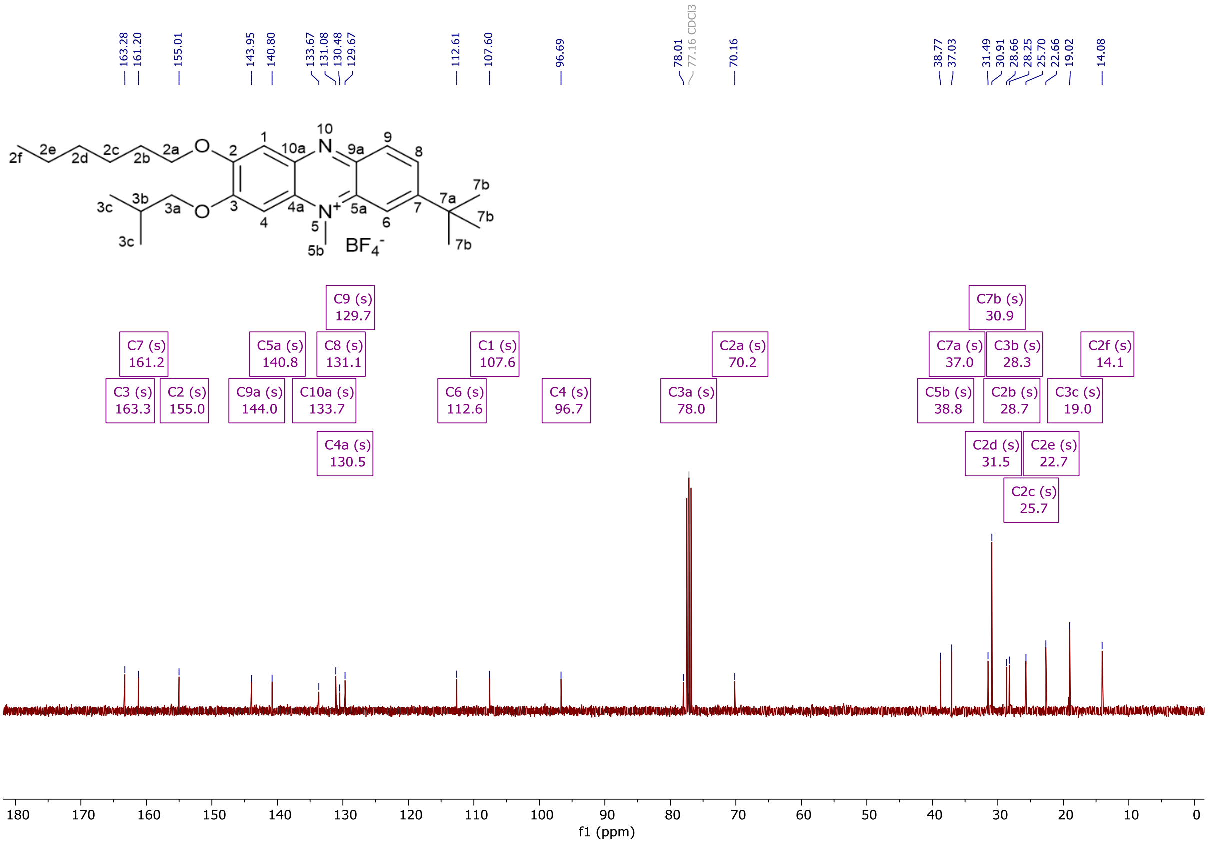

$^{13}C\{^{1}H\}$ NMR (101 MHz, Chloroform-*d*) spectrum of **2e** tetrafluoroborate.

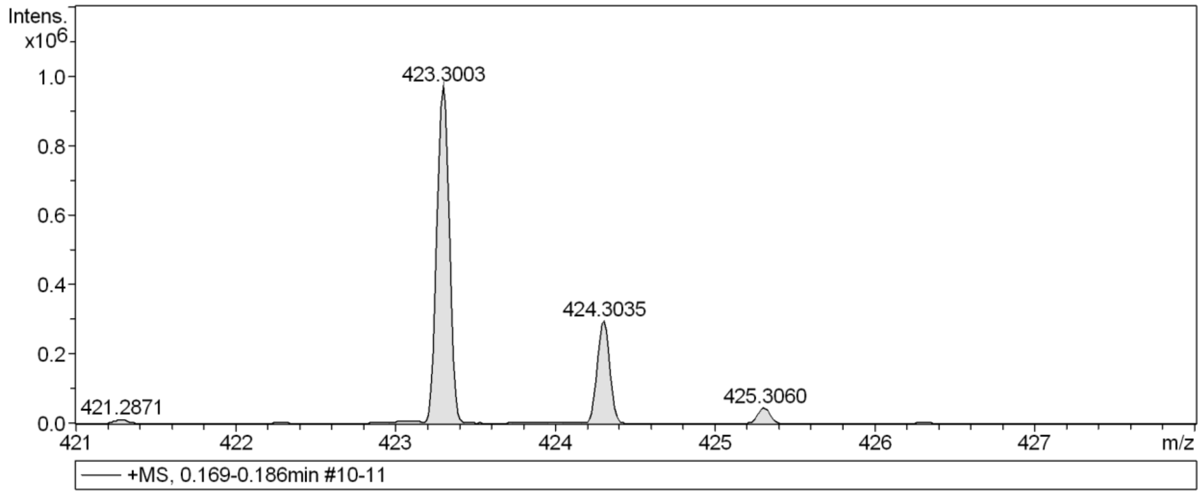

HRMS (ESI+) spectrum of **2e**.

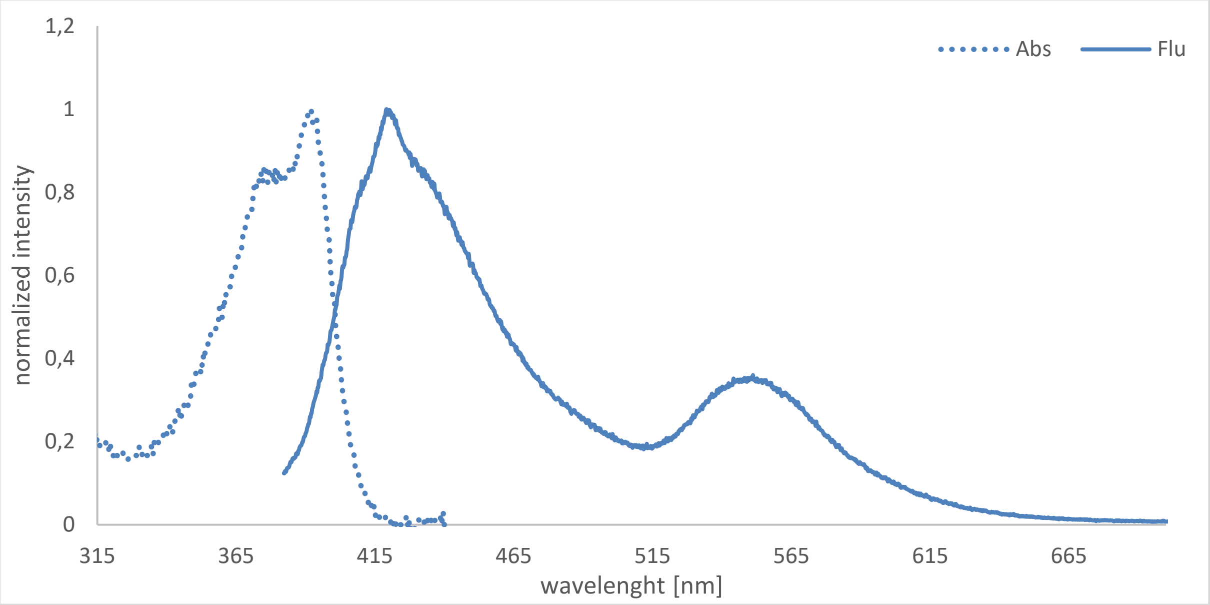

The absorbance and fluorescence (λ_{EX} = 375 nm) spectrum of **2e tetrafluoroborate** in acetonitrile. The compound has Φ = 0.011 in acetonitrile solution.

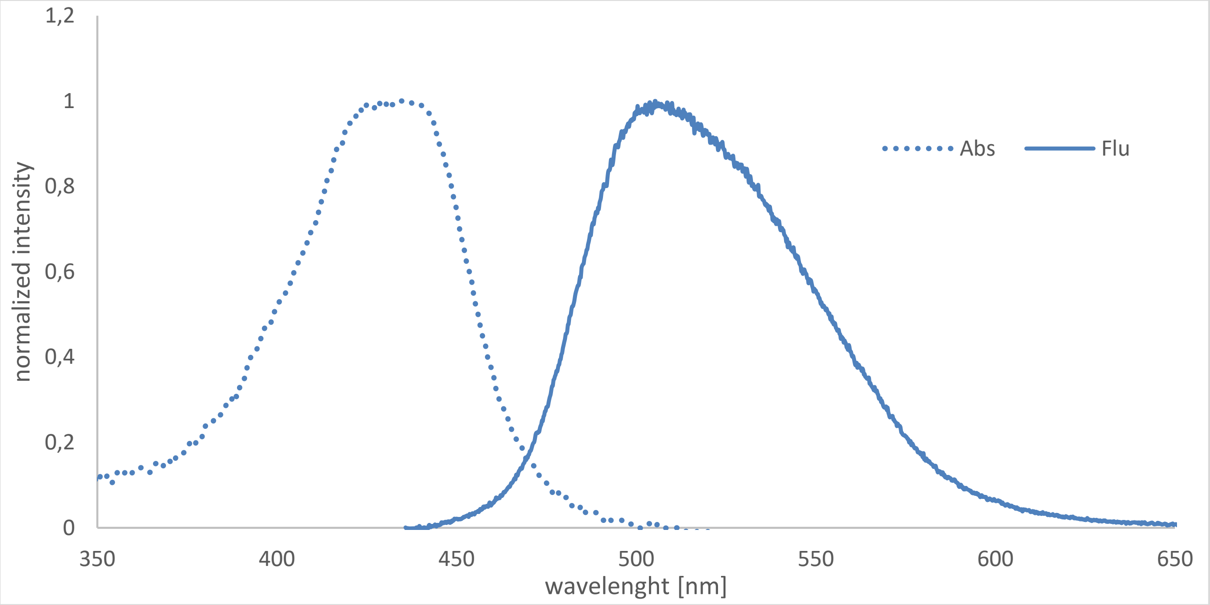

The absorbance and fluorescence (λ_{EX} = 430 nm) spectrum of **2e tetrafluoroborate** in chloroform. The compound has Φ = 0.004 in chloroform solution.

2,3-bis(hexyloxy)-5-methylphenazin-5-ium tetrafluoroborate (2f)

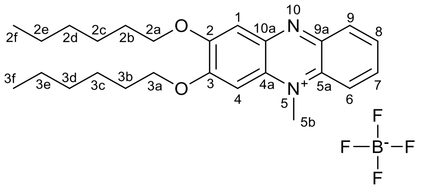

The compound was synthesised from **4f**. Received orange solid. M.p. = 198.6 – 205.5 °C. Yield = 133 mg (77 %, Cl^- salt), Yield = 133 mg, (92 %, anion exchange of Cl^- to BF_4^-). The total yield of cyclisation and anion exchange is 71 %.

^{1}H NMR (400 MHz, Chloroform-*d*) δ: 8.52 (dd, *J* = 9.1, 0.7 Hz, 1H, H_6), 8.38 (dd, *J* = 8.5, 1.4 Hz, 1H, H_9), 8.19 (ddd, *J* = 8.9, 6.9, 1.5 Hz, 1H, H_8), 7.99 (ddd, *J* = 8.3, 6.9, 1.0 Hz, 1H, H_7), 7.80 (s, 1H, H_1), 7.49 (s, 1H, H_4), 4.91 (s, 3H, H_{5b}), 4.61 (t, *J* = 6.2 Hz, 2H, H_{3a}), 4.27 (t, *J* = 6.4 Hz, 2H, H_{2a}), 2.05 – 1.90 (m, 4H, $H_{2b,3b}$), 1.64 – 1.49 (m, 4H, $H_{2c,3c}$), 1.46 – 1.31 (m, 8H, $H_{2e,2e,3d,3e}$), 0.98 – 0.86 (m, 6H, $H_{2f,3f}$).

^{13}C{^{1}H} NMR (101 MHz, Chloroform-*d*) δ: 163.7 (C_3), 155.3 (C_2), 144.7 (C_{9a}), 141.9 (C_{5a}), 136.1 (C_{10a}), 134.0 (C_{4a}), 131.4 (C_8), 130.5 (C_7), 130.4 (C_9), 118.0 (C_6), 107.5 (C_1), 96.6 (C_4), 72.8 (C_{3a}), 70.3 (C_{2a}), 38.9 (C_{5b}), 31.6 (C_{3d}), 31.5 (C_{2d}), 28.7 (C_{3b}), 28.6 (C_{2b}), 25.71 (C_{3c}), 25.66 (C_{2c}), 22.7 (C_{3e}), 22.6 (C_{2e}), 14.1 ($C_{2f,3f}$).

HRMS (ESI) *m/z* Calculated for $C_{25}H_{35}N_2O_2$ $[M]^+$: 395.2693, found: 395.2695.

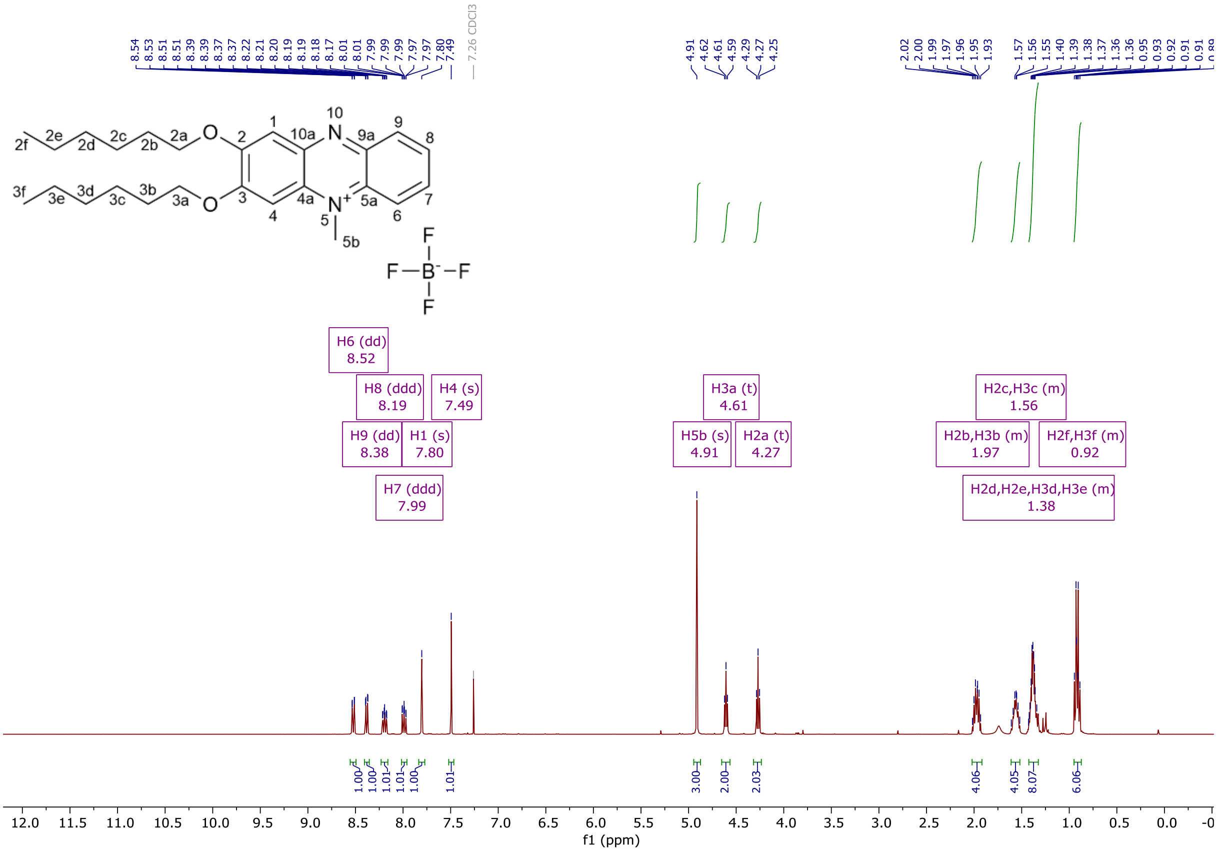

^{1}H NMR (400 MHz, Chloroform-*d*) spectrum of **2f** tetrafluoroborate.

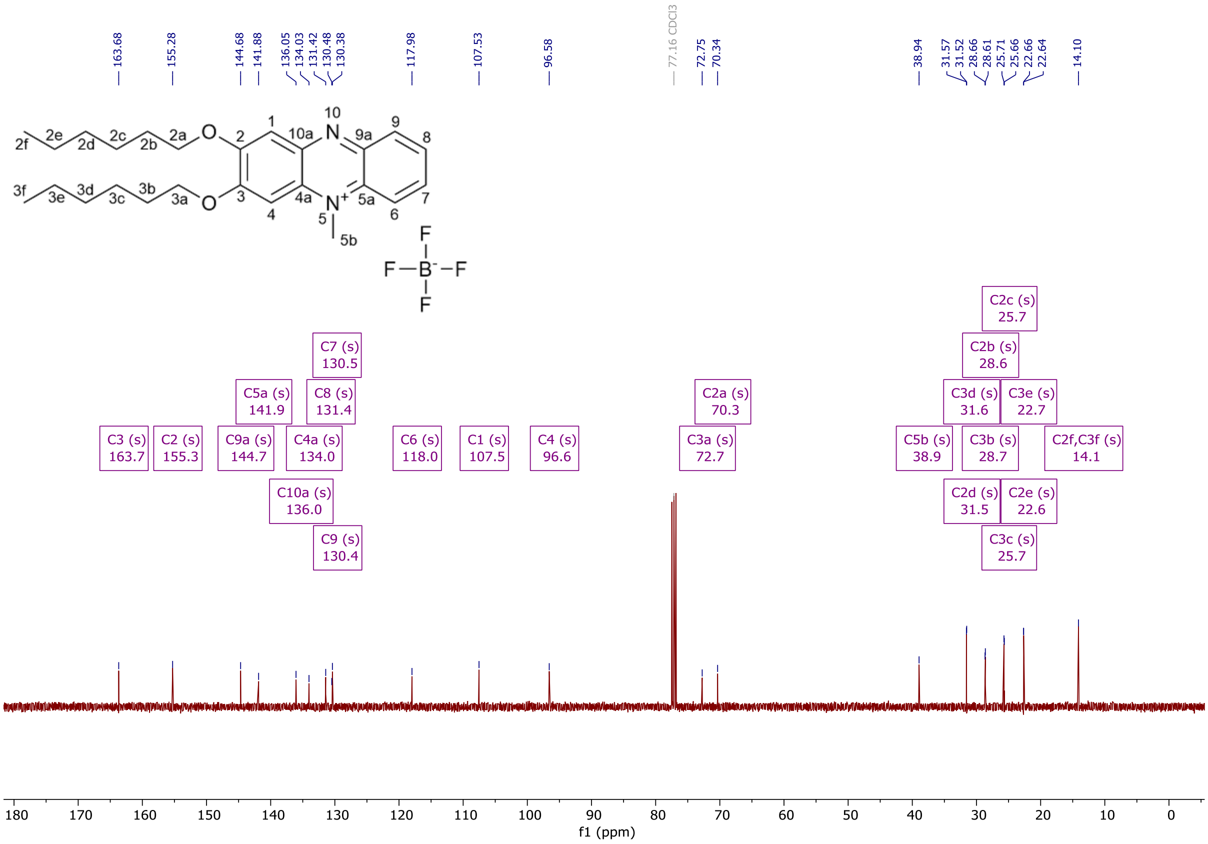

$^{13}C\{^1H\}$ NMR (101 MHz, Chloroform-*d*) spectrum of **2f** tetrafluoroborate.

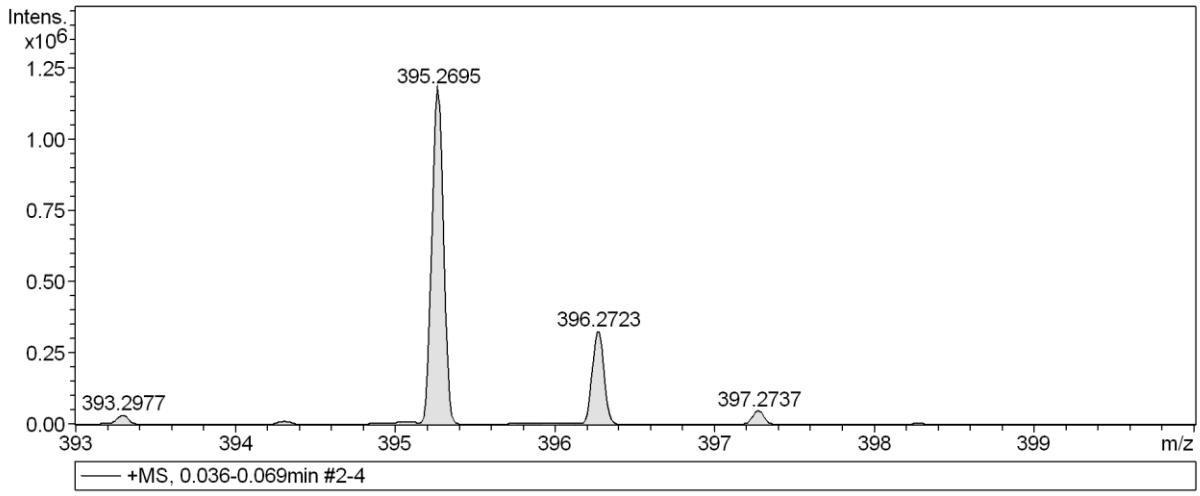

HRMS (ESI+) spectrum of **2f**.

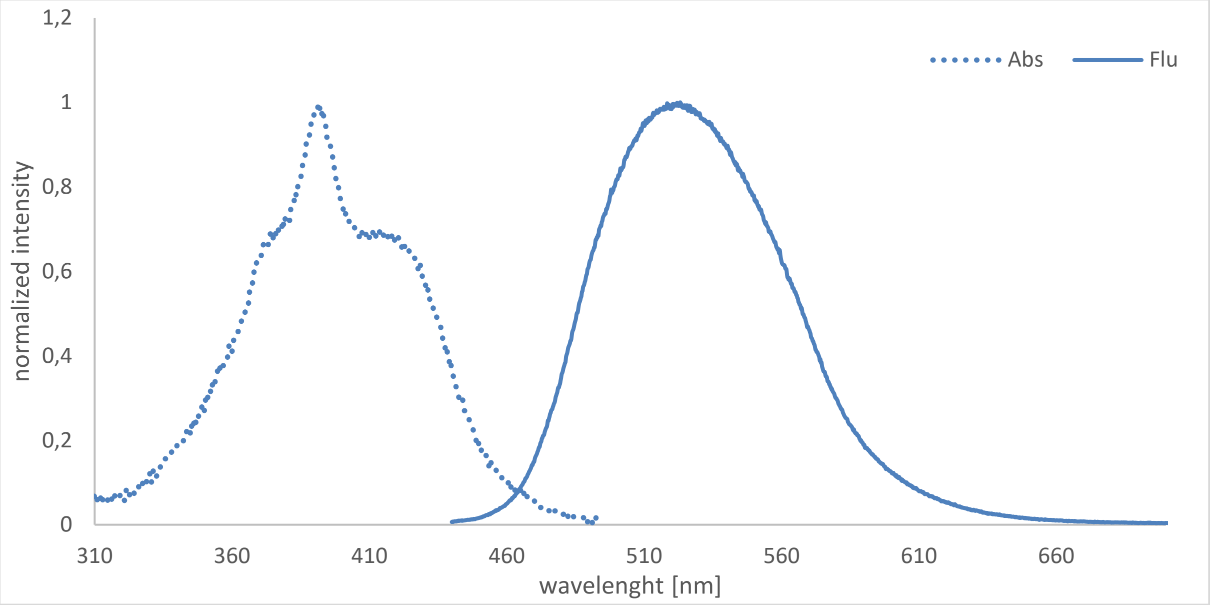

The absorbance and fluorescence (λ_{EX} = 430 nm) spectrum of **2f tetrafluoroborate** in acetonitrile. The compound has Φ = 0.025 in acetonitrile solution.

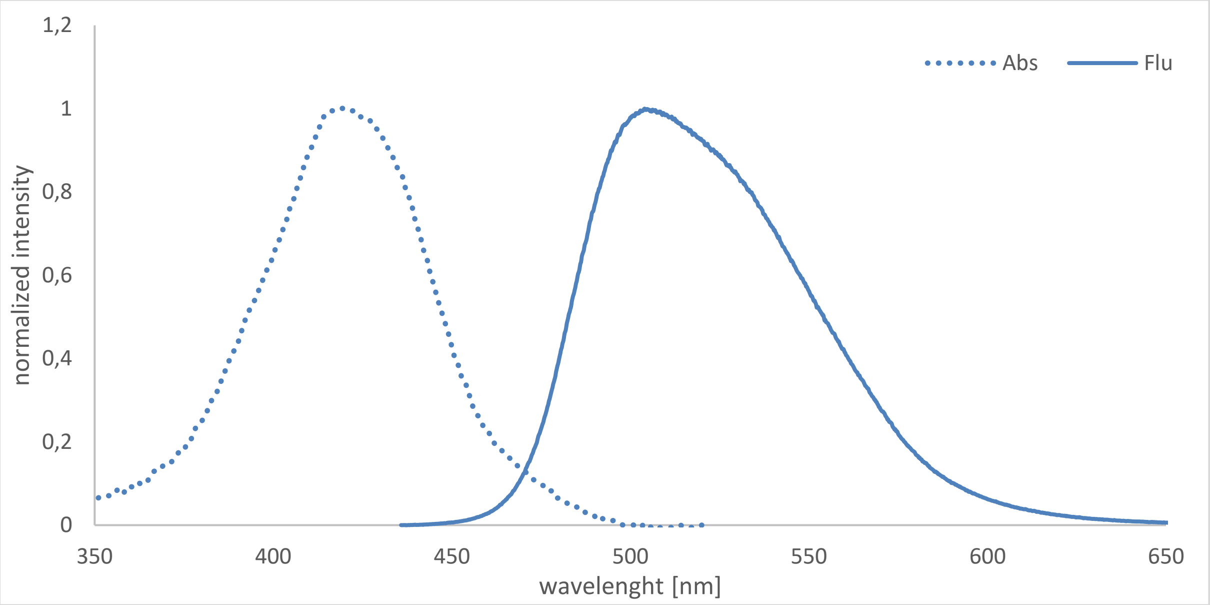

The absorbance and fluorescence (λ_{EX} = 430 nm) spectrum of **2f tetrafluoroborate** in chloroform. The compound has Φ = 0.034 in chloroform solution.

Buchwald Hartwig coupling of 2-nitroanilines with 1-bromo-2-nitrobenzene derivatives (3a-3e).

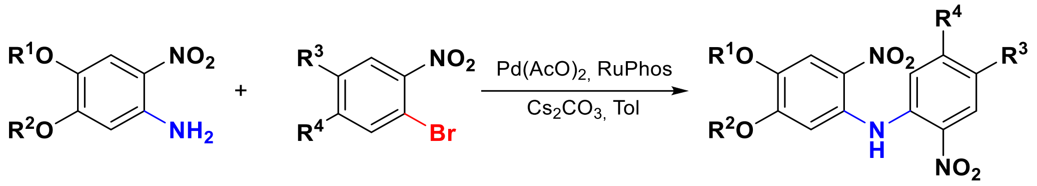

R^1,R^2 = Alkyl, R^3 = -OAlkyl, $-CF_3$, -H, R^4 = -OAlkyl, -H, -*t*Bu

In the vial (20 mL of volume) 2-nitroaniline (1 mmol) and 1-bromo-2nitrobenzene derivative (1.05 eq. 1.05 mmol) were placed. Palladium(II) acetate (11 mg, 0.05 eq.), RuPhos (35 mg, 0.075 eq.), and cesium carbonate (391 mg, 1.2 eq.) were then added. The vial was closed with a septum and flushed with argon through a needle three times. The dry toluene was added through a syringe (4 mL) and the mixture was bubbled with argon for 3 minutes. The septum was replaced with a seal cap and heated for 90 minutes (**3a-3c**) / 24 h (**3d**,**3e**) at 90 °C (**3a-3c**) / 110 °C (**3d**,**3e**) on an aluminium heating block. The mixture was then cooled to room temperature, diluted with DCM (50 mL) and extracted with water (100 mL), then brine (50 mL). The organic phase was collected, and dried over anhydrous magnesium sulphate and the solvent was removed under reduced pressure on a rotary evaporator. The crude product was purified by gradient elution column chromatography (silica gel, DCM / MeOH: 0 – 2.5%).

Using a small excess (1.05 eq.) of the 1-bromo-2-nitrobenzene derivative is beneficial for product purification as it separates well during chromatography. The unreacted 2-nitroanilines are usually inseparable from the product.

The reaction on a gram scale was performed in 50 mL vial using 20 mL of toluene. All reagents were scaled proportionally, and the reaction mixture was diluted with 100 mL of DCM instead of 50 mL. The rest of the protocol remains the same.

4-ethoxy-*N*-(4-(hexyloxy)-5-isobutoxy-2-nitrophenyl)-5-methoxy-2-nitroaniline (3a)

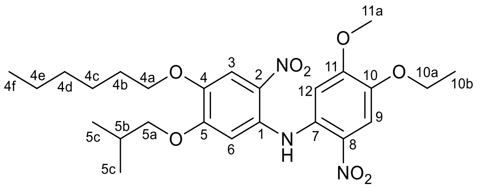

The compound was synthesised from 4-(hexyloxy)-5-isobutoxy-2-nitroaniline obtained as described in literature[4] and **5d**. Received orange solid. M.p. = 112.8 – 117.5 °C Yield = 497 mg (98 %). Yield = 1.56 g (96 %, gram scale synthesis was performed on 1.00 g of substrate – 2-nitroaniline derivative)

^{1}H NMR (300 MHz, Chloroform-*d*) δ: 11.11 (s, 1H, H_{NH}), 7.54 (s, 2H, $H_{3,9}$), 6.95 (s, 1H, H_6), 6.92 (s, 1H, H_{12}), 4.05 (q, J = 7.0 Hz, 2H, H_{10a}), 3.95 (t, J = 6.4 Hz, 2H, H_{4a}), 3.83 (s, 3H, H_{11a}), 3.68 (d, J = 6.5 Hz, 2H, H_{5a}), 2.16 – 2.05 (m, 1H, H_{5b}), 1.85 – 1.70 (m, 2H, H_{4b}), 1.47 – 1.40 (m, 5H, $H_{4c,10b}$), 1.37 – 1.25 (m, 4H, $H_{4d,4e}$), 0.98 (d, J = 6.7 Hz, 6H, H_{5c}), 0.90 – 0.80 (m, 3H, H_{4f}).

^{13}C{^{1}H} NMR (75 MHz, Chloroform-*d*) δ: 155.5 (C_{11}), 155.3 (C_5), 143.7 (C_4), 143.0 (C_{10}), 133.7 (C_7), 133.5 (C_1), 130.3 (C_8), 130.2 (C_2), 109.5 (C_3), 108.7 (C_9), 102.2 (C_6), 101.2 (C_{12}), 75.6 (C_{5a}), 69.5 (C_{4a}), 64.9 (C_{10a}), 56.4 (C_{11a}), 31.4 (C_{4d}), 28.9 (C_{4b}), 28.1 (C_{5b}), 25.6 (C_{4c}), 22.5 (C_{4e}), 19.0 (C_{5c}), 14.5 (C_{10b}), 13.9 (C_{4f}).

HRMS (ESI) *m/z* Calculated for $C_{25}H_{35}N_3O_8Na$ $[M+Na]^+$: 528.2322, found: 528.2312.

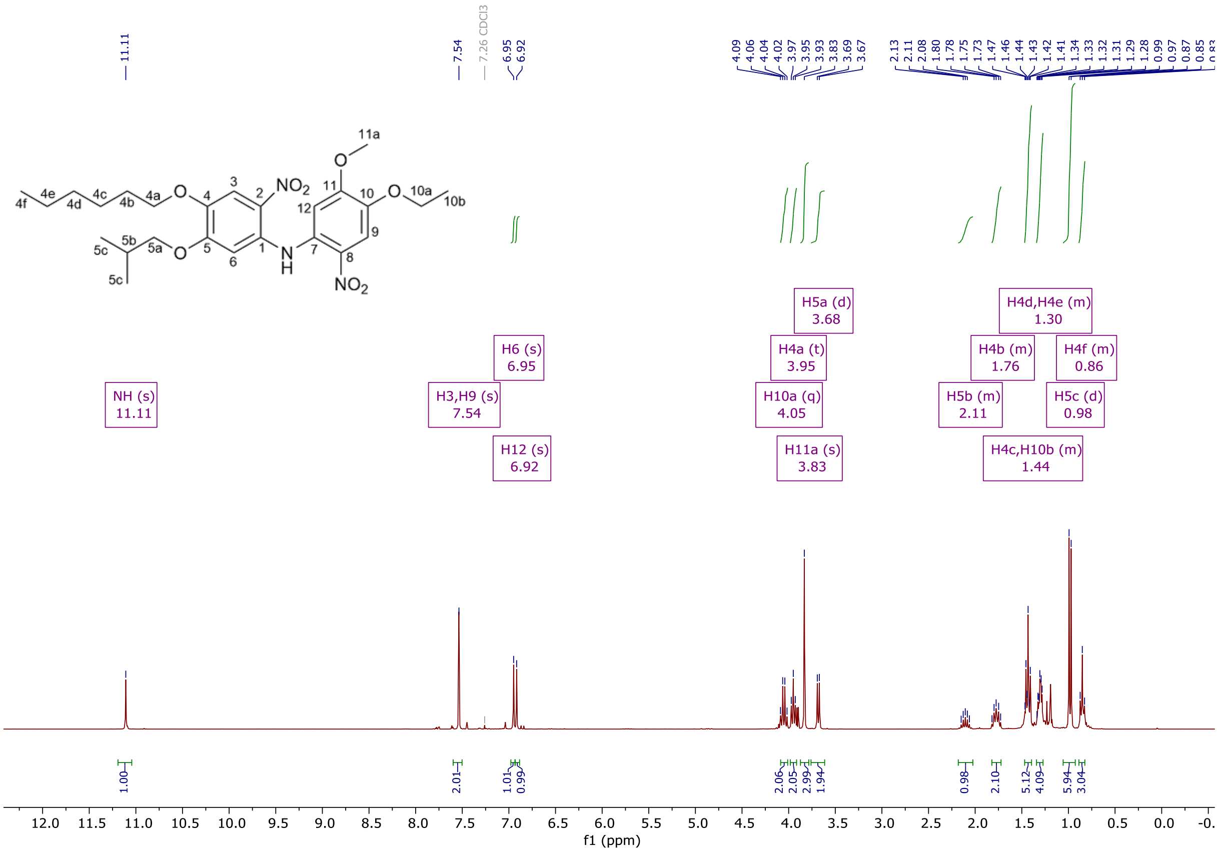

^{1}H NMR (300 MHz, Chloroform-*d*) spectrum of **3a**.

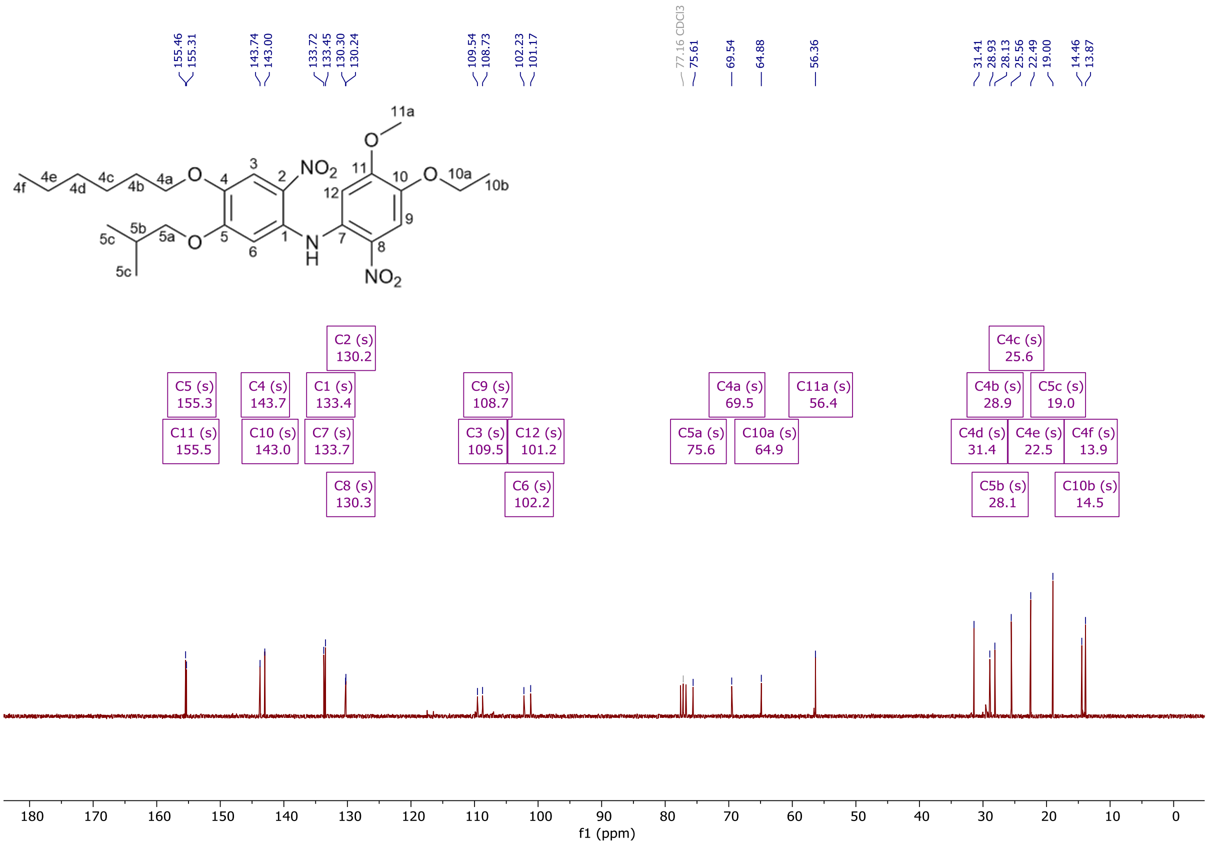

^{13}C{^{1}H} NMR (75 MHz, Chloroform-*d*) spectrum of **3a**.

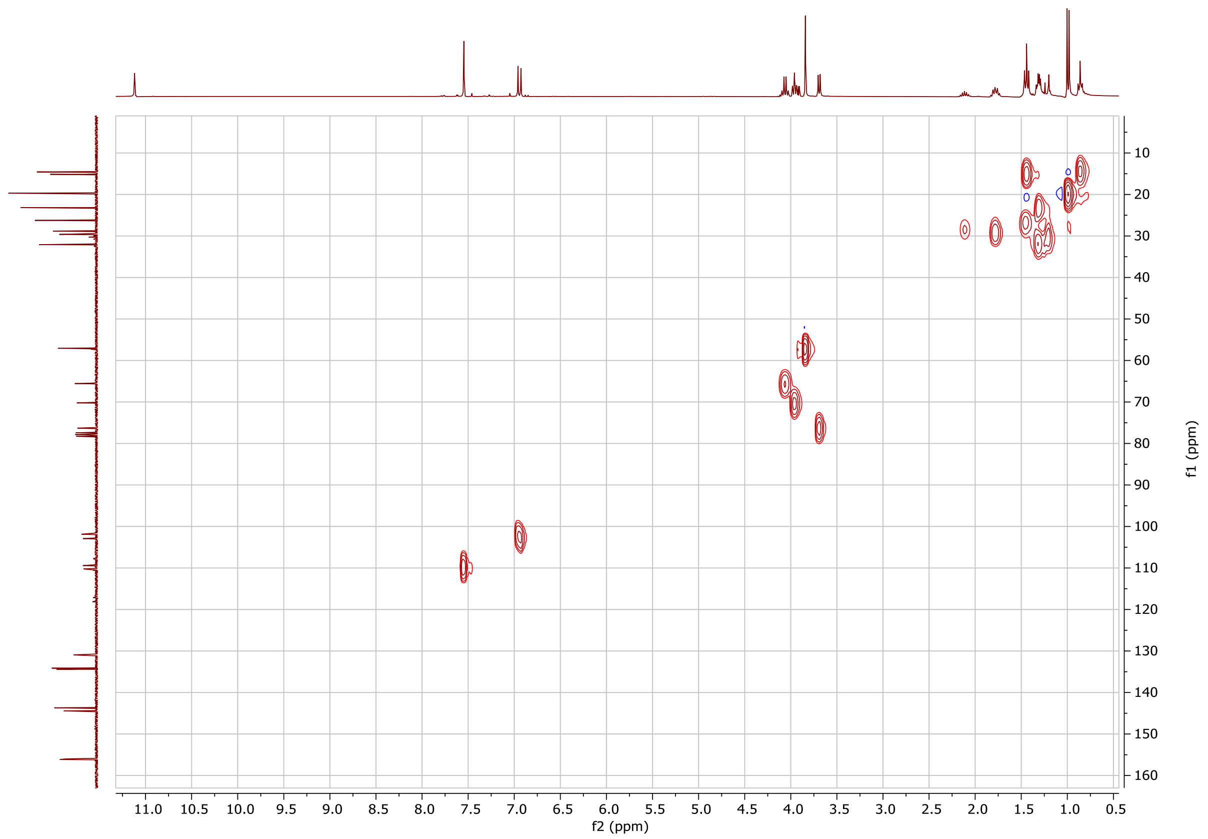

g-HSQC NMR (Chloroform-*d*) spectrum of **3a**.

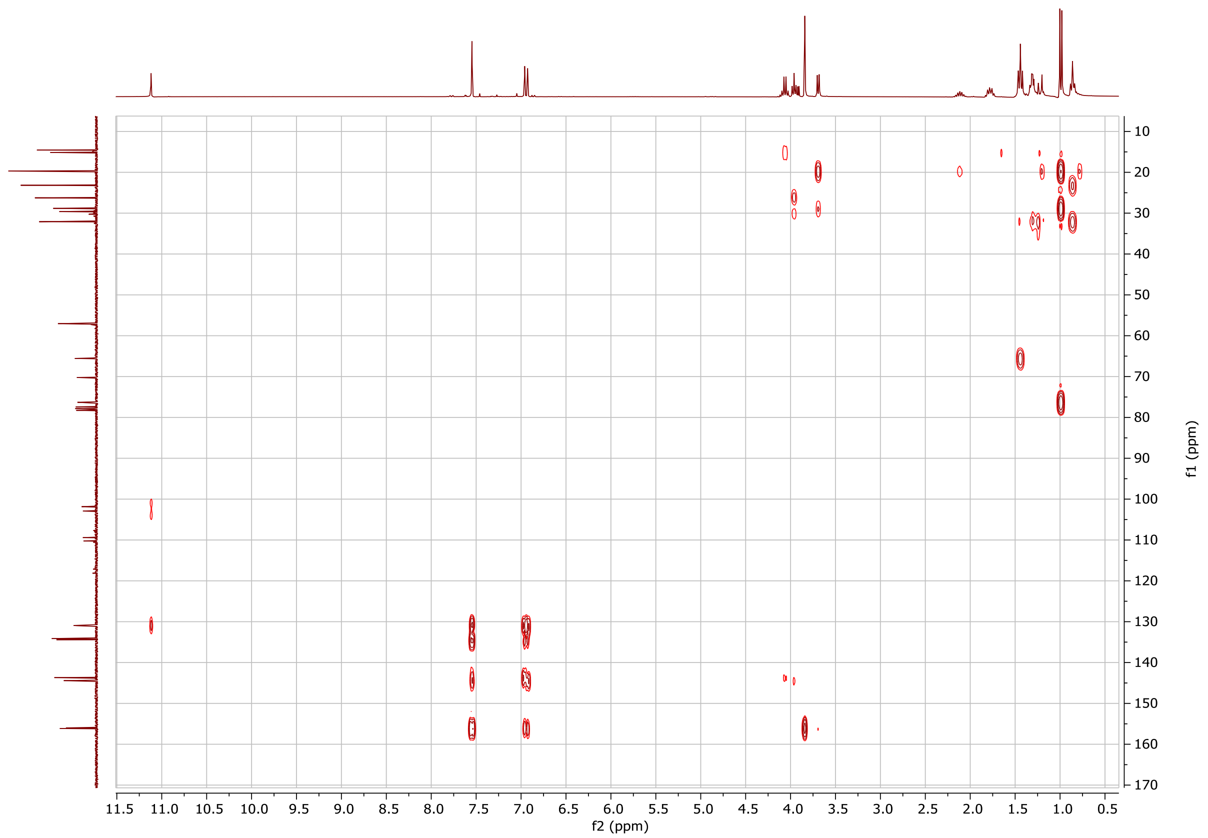

g-HMBC NMR (Chloroform-*d*) spectrum of **3a**.

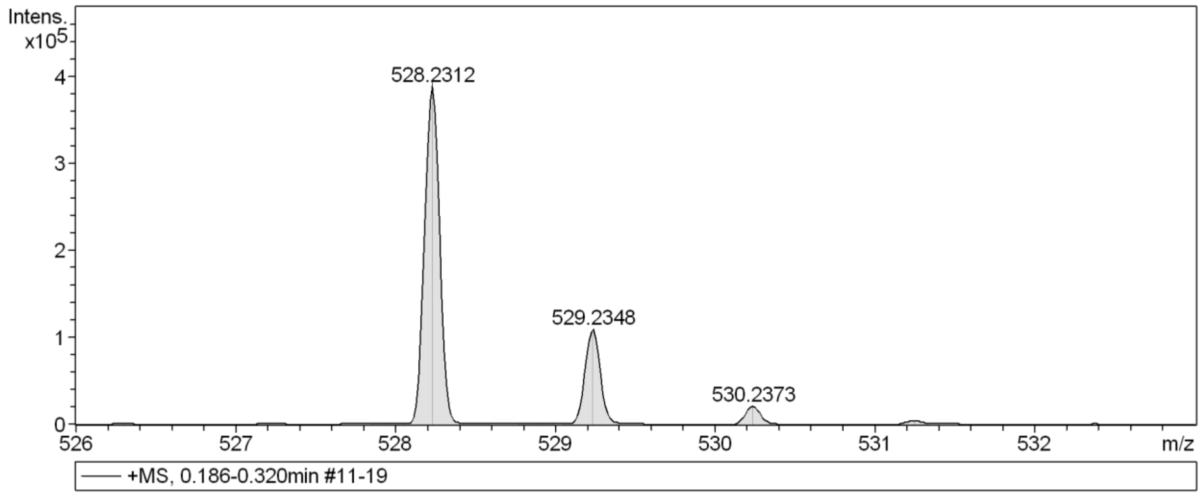

HRMS (ESI+) spectrum of **3a**.

10-(5-((4-(dodecyloxy)-5-(isopentyloxy)-2-nitrophenyl)amino)-2-methoxy-4-nitrophenoxy)decyl acetate (3b)

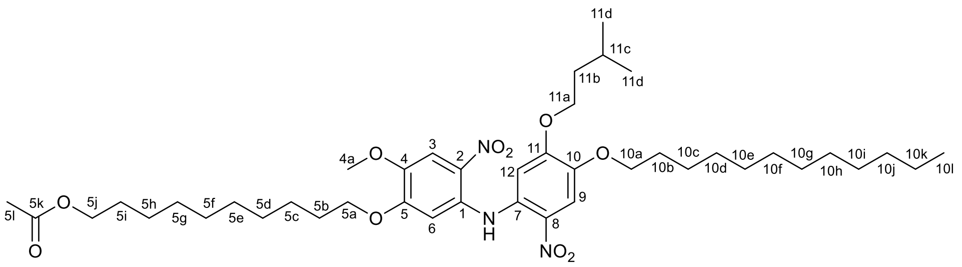

The compound was synthesised from **6c** and **5e**. Received orange solid. M.p. = 68.2 – 70.8 °C. Yield = 630 mg (81 %).

^{1}H NMR (400 MHz, Chloroform-*d*) δ: 11.14 (s, 1H, H_{NH}), 7.61 (d, *J* = 1.1 Hz, 2H, $H_{3,9}$), 6.91 (s, 2H, $H_{6,12}$), 4.06 – 3.96 (m, 4H, $H_{11a,5j}$), 3.97 – 3.90 (m, 4H, $H_{5a,10a}$), 3.88 (s, 3H, H_{4a}), 2.01 (s, 3H, H_{5l}), 1.87 – 1.74 (m, 5H, $H_{5b,10b,11c}$), 1.71 (q, *J* = 6.5 Hz, 2H, H_{11b}), 1.62 – 1.54 (m, 2H, H_{5i}), 1.50 – 1.20 (m, 30H, $H_{5c,5d,5e,5f,5g,5h,10c,10d,10e,10f,10g,10h,10i,10j,10k}$), 0.92 (d, *J* = 6.5 Hz, 6H, H_{11d}), 0.87 – 0.82 (m, 3H, H_{10l}).

^{13}C{^{1}H} NMR (101 MHz, Chloroform-*d*) δ: 171.2 (C_{5k}), 155.3 (C_5), 154.9 (C_{11}), 144.1 (C_4), 143.8 (C_{10}), 134.0 (C_1), 133.6 (C_7), 130.6 (C_8), 130.4 (C_2), 109.4 (C_9), 108.0 (C_3), 102.3 (C_{12}), 101.9 (C_6), 69.9 – 69.2 (m, $C_{5a,10a}$), 68.0 (C_{11a}), 64.6 (C_{5j}), 56.4 (C_{4a}), 37.5 (C_{11b}), 31.93 (C_{10j}), 29.71 (C_{10e}), 29.66 (C_{10d}), 29.62 (C_{5d}), 29.61 (C_{5f}), 29.41 ($C_{5e,5g}$), 29.39 (C_{10b}), 29.37 (C_{5b}), 29.3 (C_{10h}), 29.2 (C_{10i}), 29.0 (C_{10g}), 28.9 (C_{10f}), 28.6 (C_{10i}), 26.0 (C_{11b}), 26.0 – 25.8 (m, $C_{5c,10c}$), 25.1 (C_{5h}), 22.7 (C_{10k}), 22.5 (C_{11d}), 21.0 (C_{5l}), 14.1 (C_{10l}).

HRMS (ESI) *m/z* Calculated for $C_{42}H_{67}N_3O_{10}Na$ $[M+Na]^+$: 796.4724, found: 796.4718.

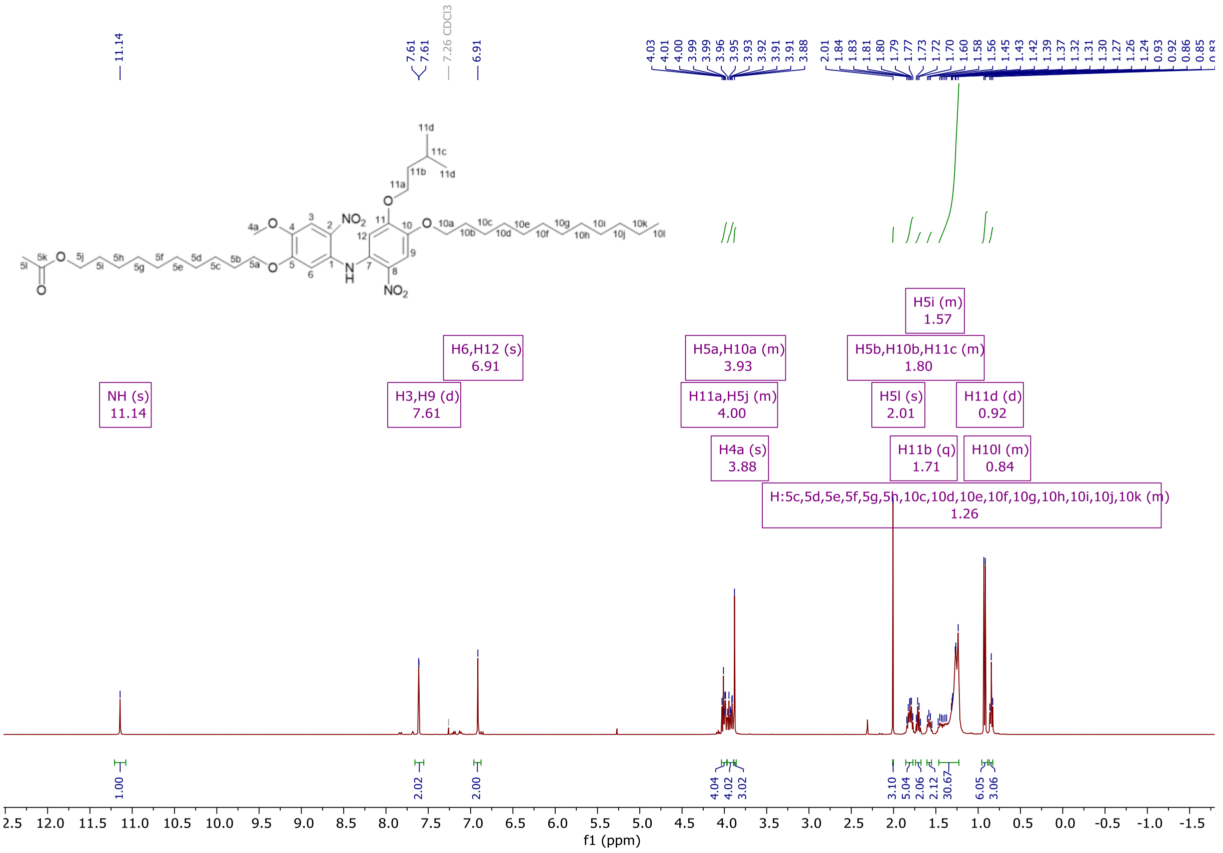

^{1}H NMR (400 MHz, Chloroform-*d*) spectrum of **3b**.

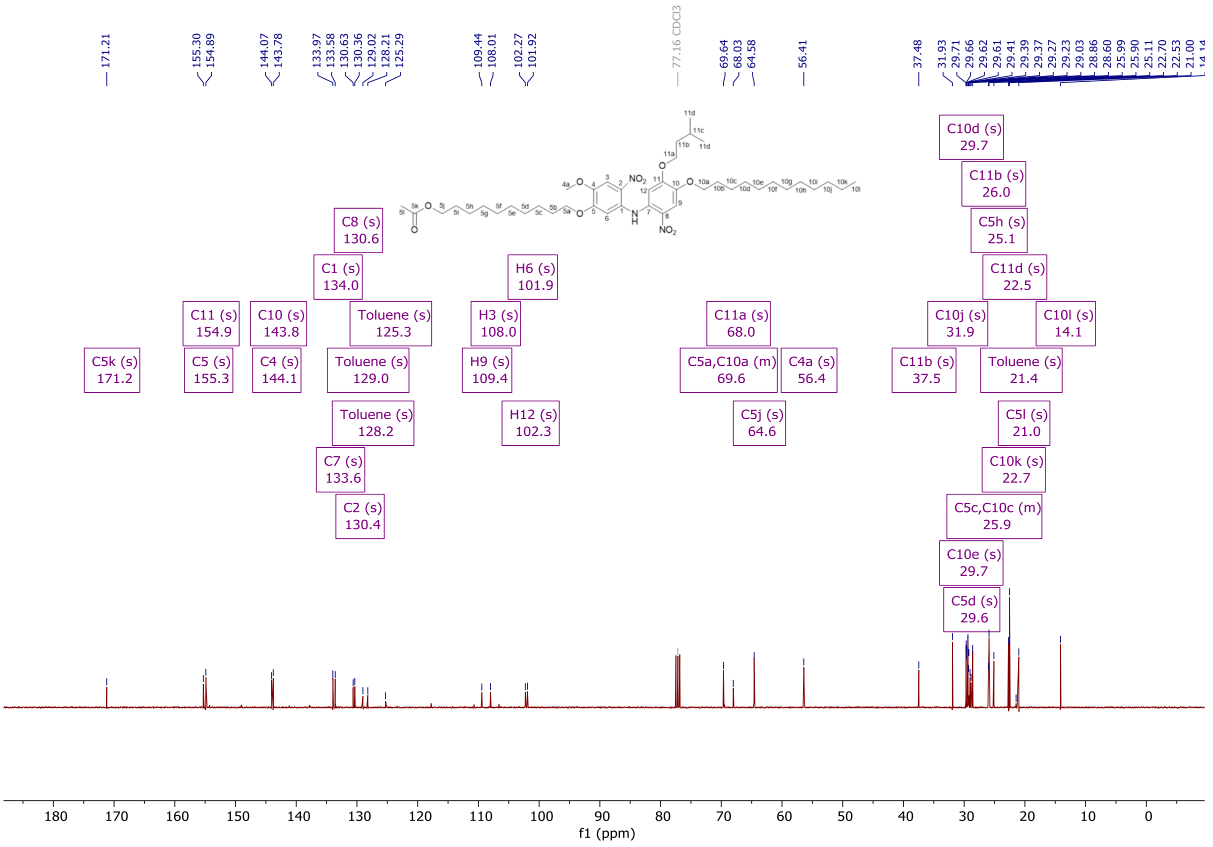

^{13}C{^{1}H} NMR (101 MHz, Chloroform-*d*) spectrum of **3b**.

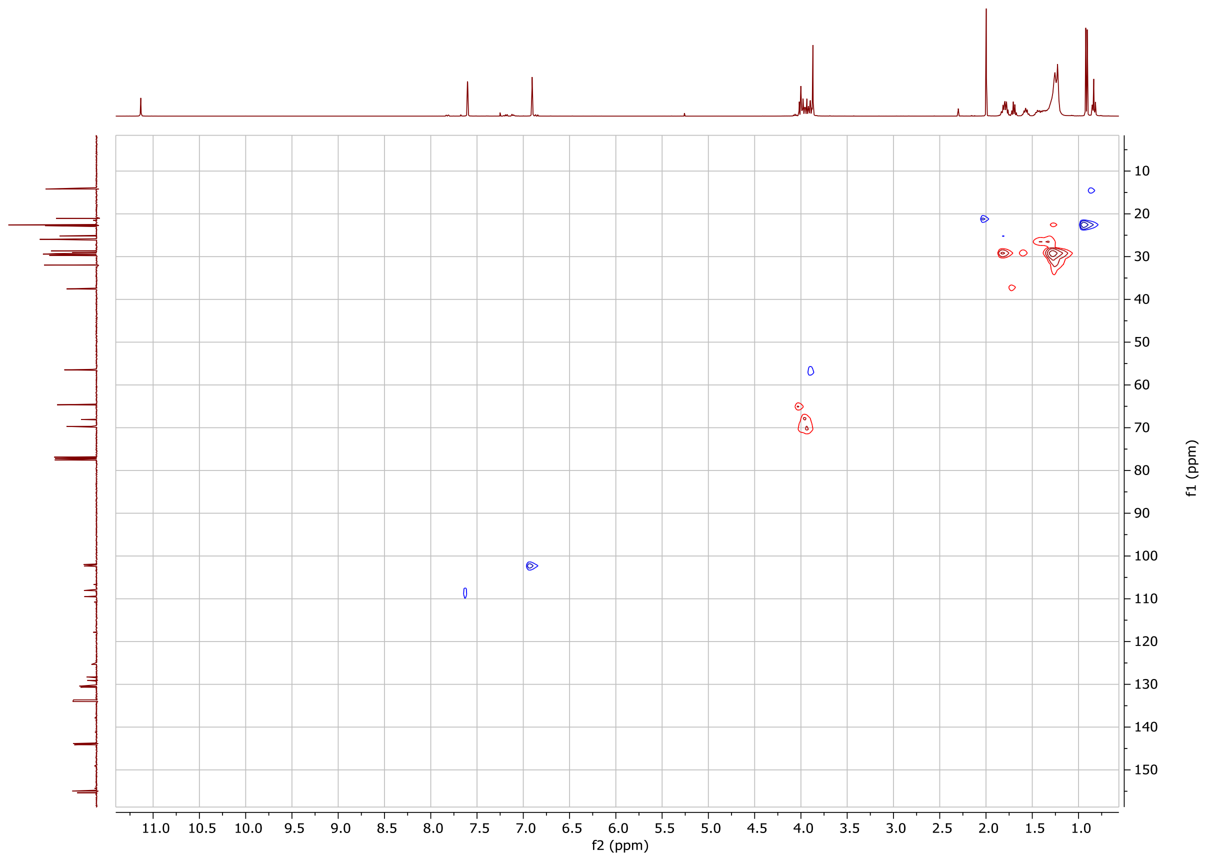

g-HSQC NMR (101 MHz, Chloroform-*d*) spectrum of **3b**.

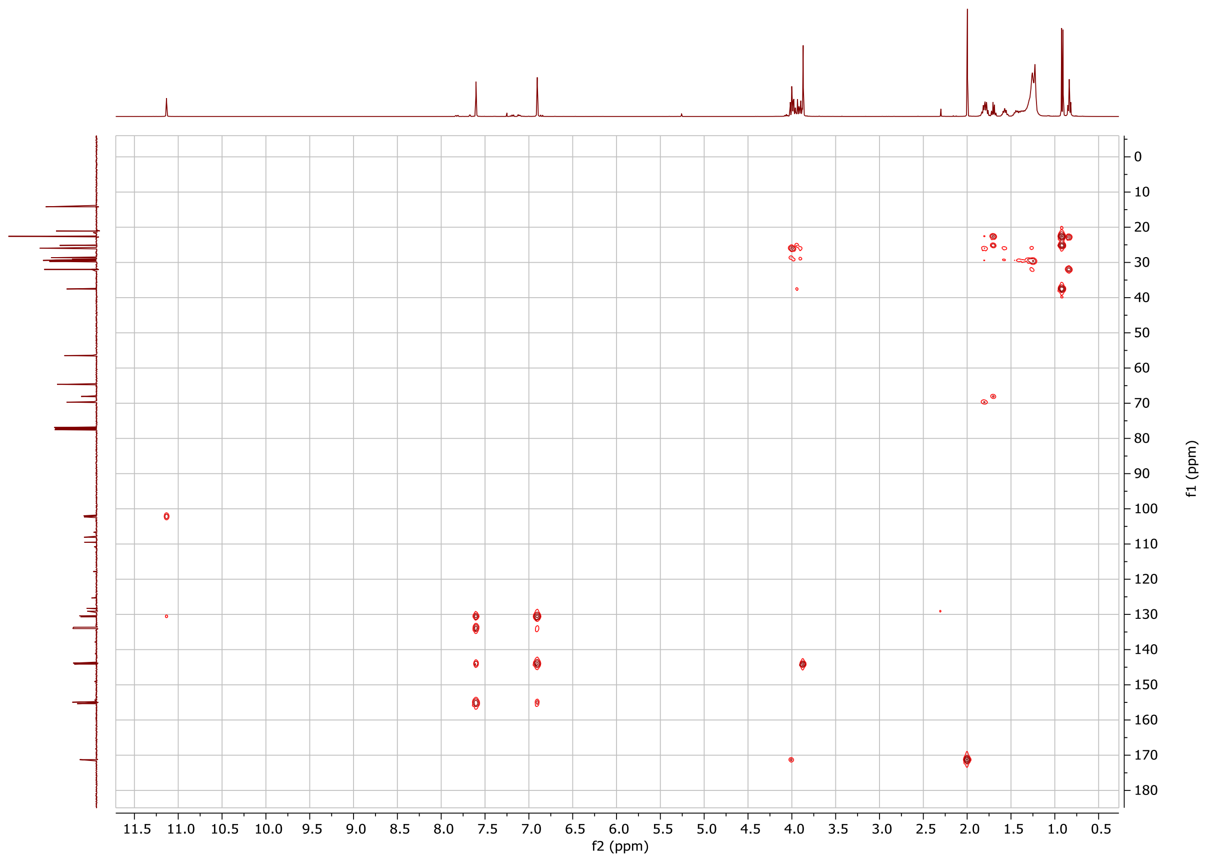

g-HMBC NMR (101 MHz, Chloroform-*d*) spectrum of **3b**.

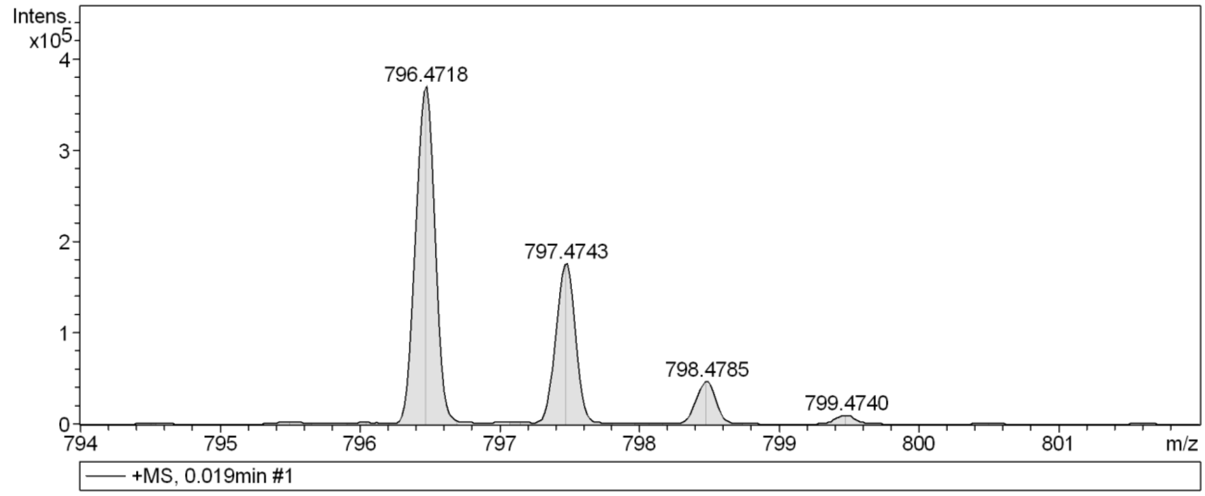

HRMS (ESI+) spectrum of **3b**.

((azanediylbis(6-methoxy-4-nitro-3,1-phenylene))bis(oxy))bis(decane-10,1-diyl) diacetate (3c)

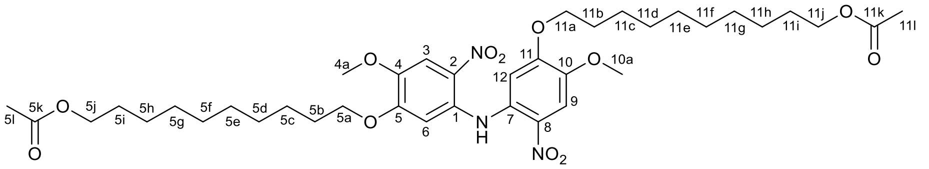

The compound was synthesised from **6b** and **5e**. Received orange solid. M.p. = 81.0 – 82.1 °C. Yield = 626 mg (84 %).

^{1}H NMR (400 MHz, Chloroform-d) δ: 11.10 (s, 1H, H_{NH}), 7.56 (s, 2H, $H_{3,9}$), 6.89 (s, 2H, $H_{6,12}$), 3.97 (t, J = 6.8 Hz, 4H, $H_{5a,11a}$), 3.89 (t, J = 6.7 Hz, 4H, $H_{5j,11j}$), 3.85 (s, 6H, $H_{4a,10a}$), 1.97 (s, 6H, $H_{5l,11l}$), 1.85 – 1.72 (m, 4H, $H_{5b,11b}$), 1.58 – 1.51 (m, 4H, $H_{5i,11i}$), 1.43 – 1.30 (m, 4H, $H_{5c,11c}$), 1.31 – 1.20 (m, 20H, $H_{5d,5e,5f,5g,5h,11d,11e,11f,11g,11h}$).

^{13}C{^{1}H} NMR (101 MHz, Chloroform-*d*) δ: 171.1 ($C_{5l,11l}$), 154.8 ($C_{5,11}$), 144.1 ($C_{4,10}$), 133.7 ($C_{1,7}$), 130.3 ($C_{2,8}$), 107.9 ($C_{3,9}$), 102.0 ($C_{6,12}$), 69.6 ($C_{5a,11a}$), 64.5 ($C_{5j,11j}$), 56.3 ($C_{4a,10a}$), 29.4 – 29.3 (m, $C_{5f,11f,5e,11e}$), 29.18 ($C_{5g,11g}$), 29.15 ($C_{5d,11d}$), 28.8 ($C_{5b,11b}$), 28.5 ($C_{5i,11i}$), 25.84 ($C_{5c,11c}$), 25.81 ($C_{5h,11h}$), 20.9 ($C_{5l,11l}$).

HRMS (ESI) *m/z* Calculated for $C_{38}H_{57}N_3O_{12}Na$ $[M+Na]^+$: 770.3840, found: 770.3834.

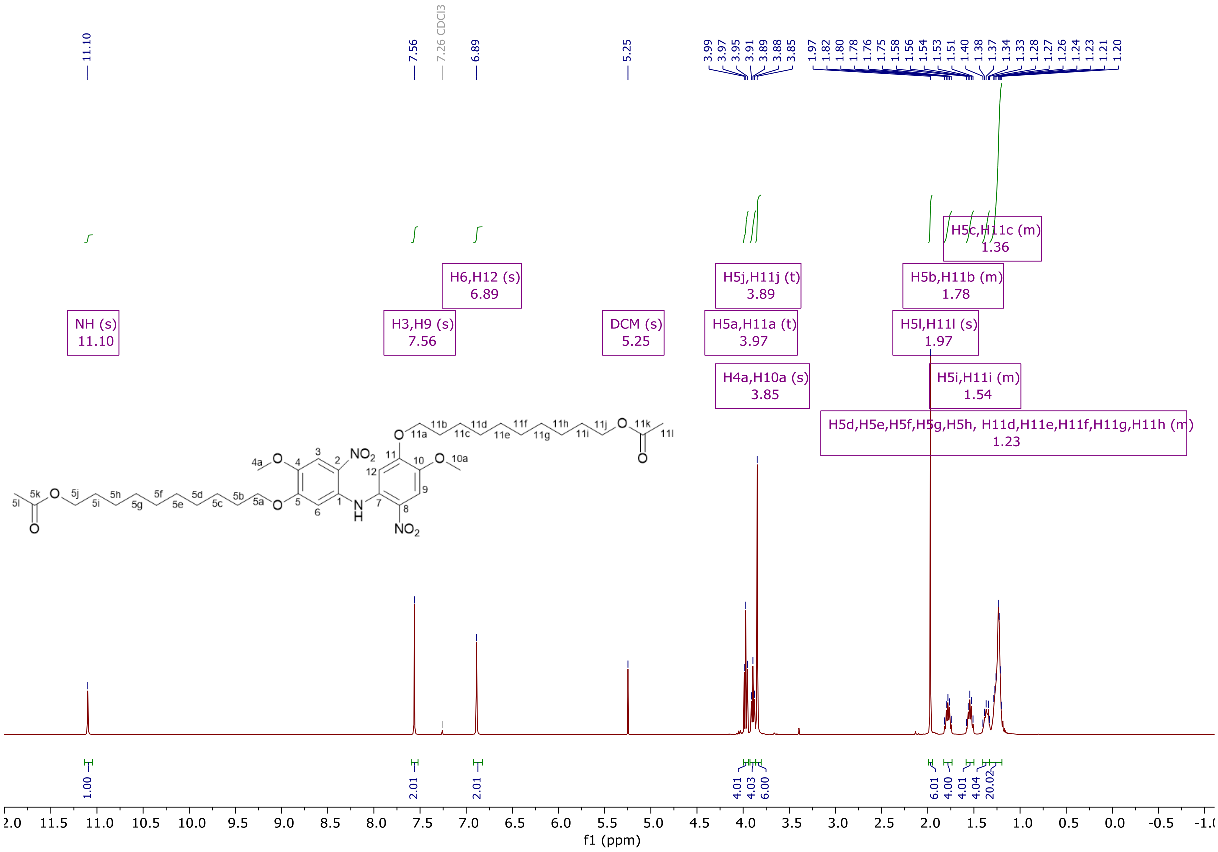

^{1}H NMR (400 MHz, Chloroform-d) spectrum of **3c**.

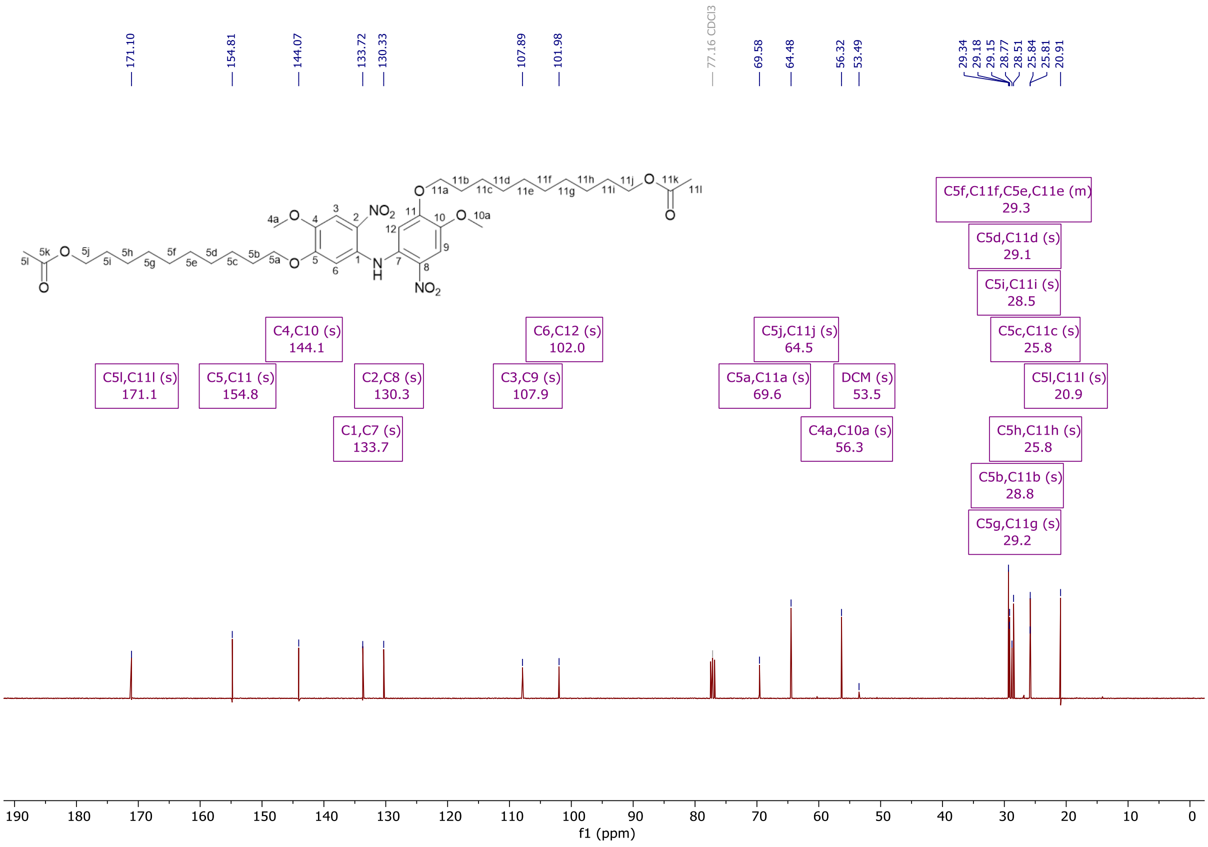

$^{13}C\{^1H\}$ NMR (101 MHz, Chloroform-*d*) spectrum of **3c**.

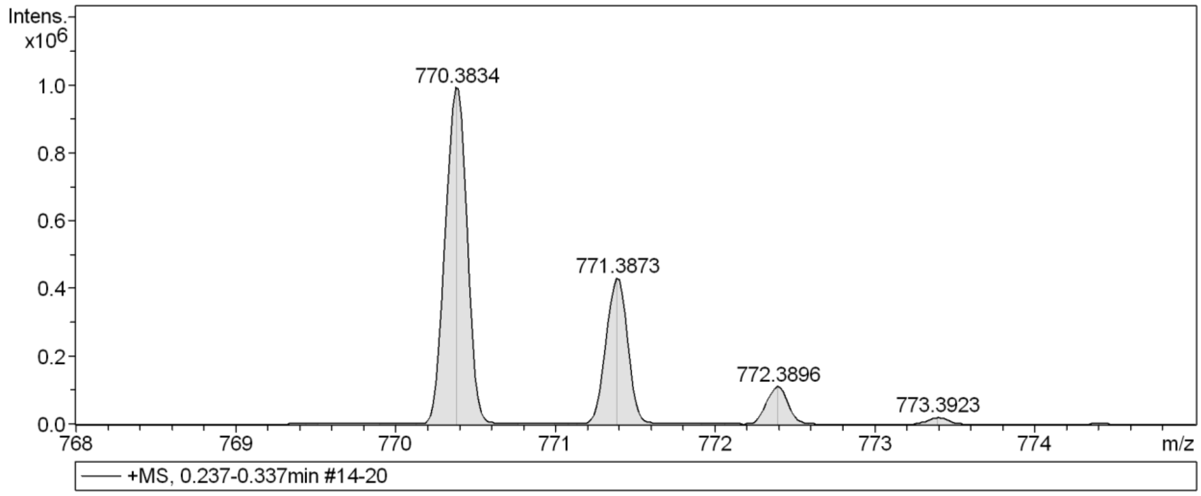

HRMS (ESI+) spectrum of **3c**.

10-(2-methoxy-4-nitro-5-((2-nitro-4-(trifluoromethyl)phenyl)amino)phenoxy)decyl acetate (3d)

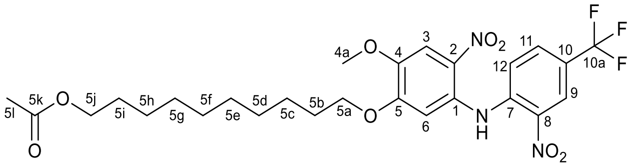

The compound was synthesised from commercially available 1-bromo-2-nitro-4-(trifluoromethyl)benzene and **6b**. Received orange solid. M.p. = 91.6 – 94.2 °C. Yield = 78 %.

^{1}H NMR (400 MHz, Chloroform-*d*) δ: 11.02 (s, 1H, H_{NH}), 8.33 (d, *J* = 2.1 Hz, 1H, H_{9}), 7.77 – 7.47 (m, 3H, $H_{3,11,12}$), 6.96 (s, 1H, H_{6}), 3.99 (t, *J* = 6.6 Hz, 2H, H_{5j}), 3.94 (t, *J* = 7.0 Hz, 2H, H_{5a}), 3.87 (s, 3H, H_{4a}), 1.94 (s, 3H, H_{5l}), 1.82 – 1.75 (m, 2H, H_{5b}), 1.57 – 1.49 (m, 2H, H_{5i}), 1.42 – 1.35 (m, 2H, H_{5c}), 1.35 – 1.16 (m, 10H, $H_{5d,H5e,H5f,H5g,H5h}$).

^{13}C{^{1}H} NMR (101 MHz, Chloroform-*d*) δ: 171.0 (C_{5k}), 154.5 (C_{5}), 145.8 (C_{4}), 141.4 (C_{7}), 135.4 (C_{1}), 132.5 (C_{8}), 131.3 (q, *J* = 3.1 Hz, C_{11}), 130.2 (C_{2}), 125.7 (q, *J* = 271.7 Hz, C_{10a}), 124.5 (q, *J* = 4.1 Hz, C_{9}), 121.7 (q, *J* = 34.5 Hz, C_{10}), 118.6 – 118.1 (m, C_{12}), 108.0 (C_{3}), 104.6 (C_{6}), 69.7 (C_{5a}), 64.4 (C_{5j}), 56.3 (C_{4a}), 29.3 ($C_{5b,5d}$), 29.2 (C_{5f}), 29.1 (C_{5e}), 28.7 (C_{5g}), 28.4 (C_{5i}), 25.73 (C_{5c}), 25.69 (C_{5h}), 20.8 (C_{5l}).

^{19}F{^{1}H} NMR (376 MHz, Chloroform-*d*) δ -62.28 (s, F_{CF3}). [relative chemical shift – no reference was used]

HRMS (ESI) *m/z* Calculated for $C_{26}H_{32}F_{3}N_{3}O_{8}Na$ $[M+Na]^{+}$: 594.2039, found: 594.2033.

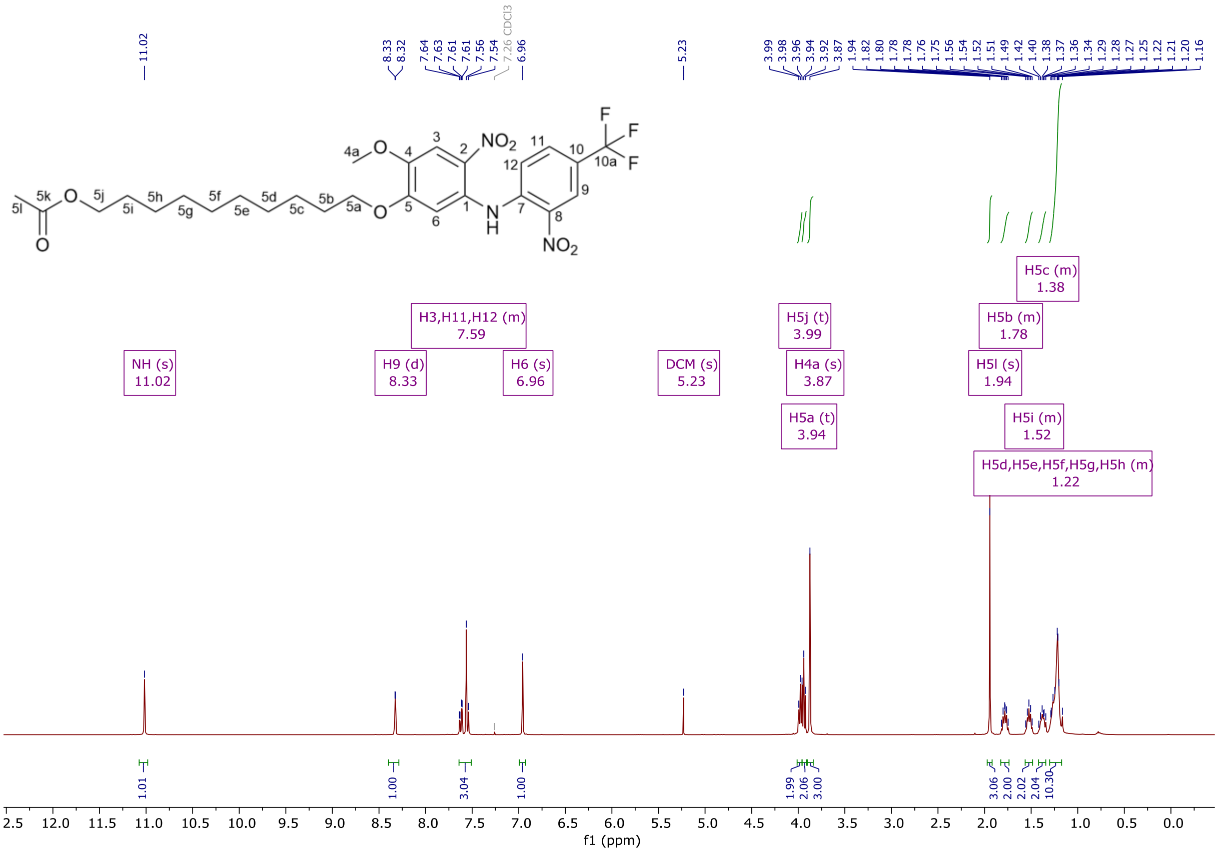

^{1}H NMR (400 MHz, Chloroform-*d*) spectrum of **3d**.

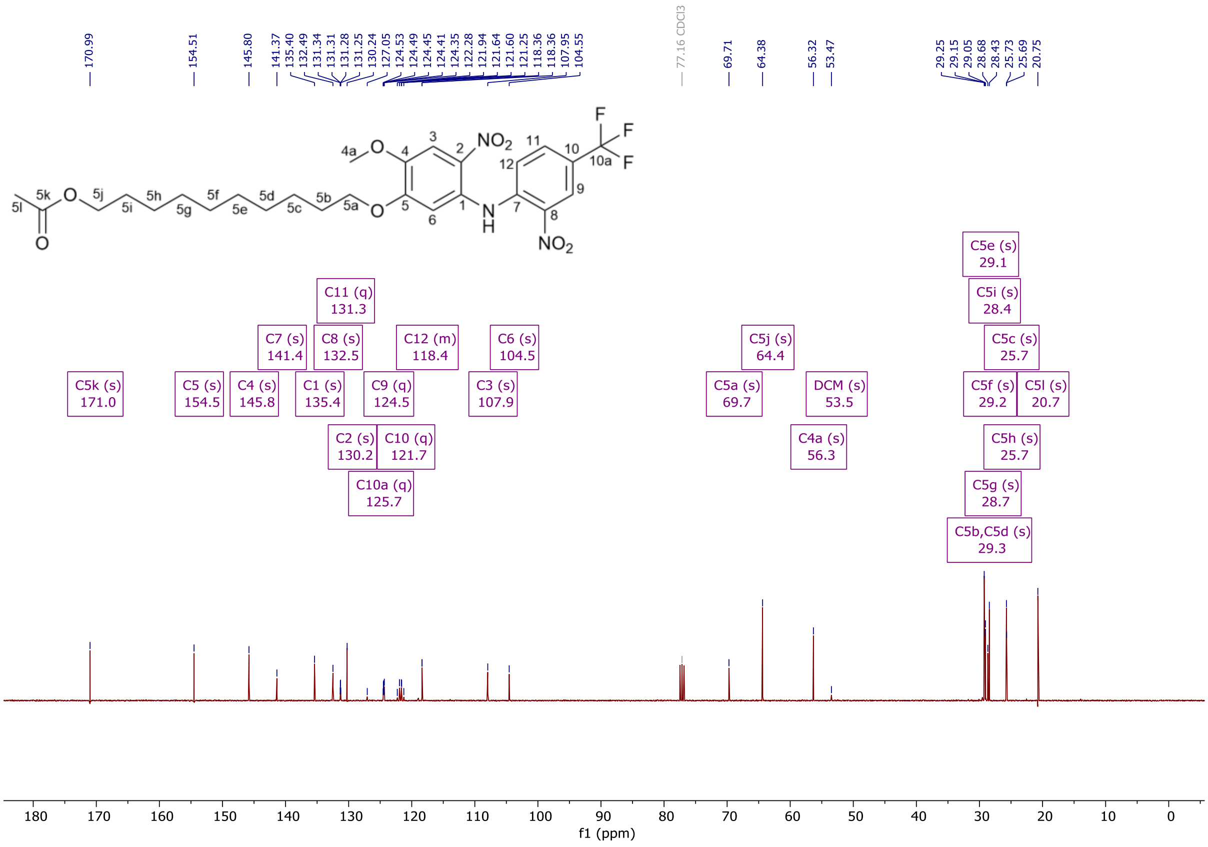

^{13}C{^{1}H} NMR (101 MHz, Chloroform-*d*) spectrum of **3d**.

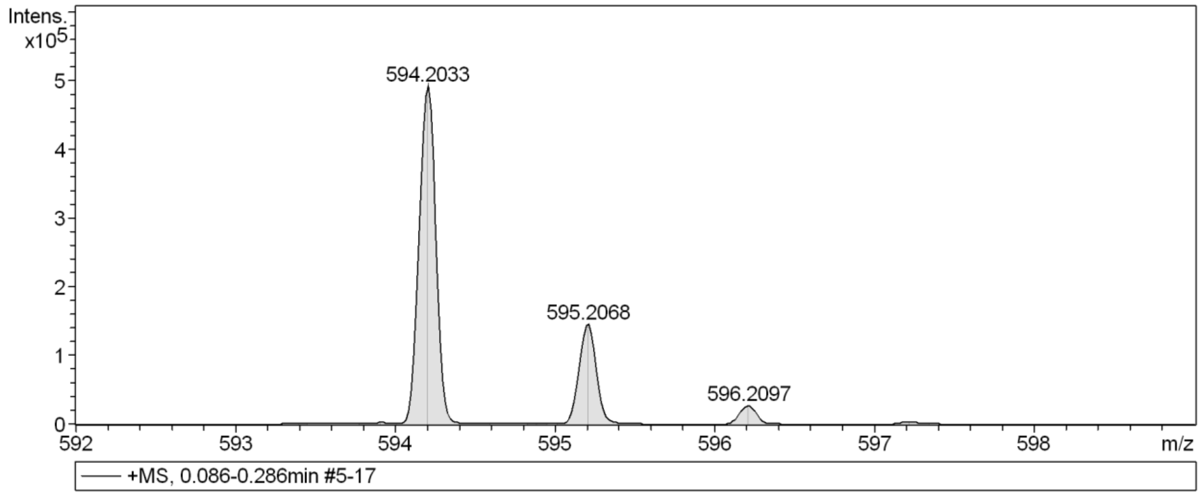

HRMS (ESI+) spectrum of **3d**.

10-(5-((5-(*tert*-butyl)-2-nitrophenyl)amino)-2-methoxy-4-nitrophenoxy)decyl acetate (3e)

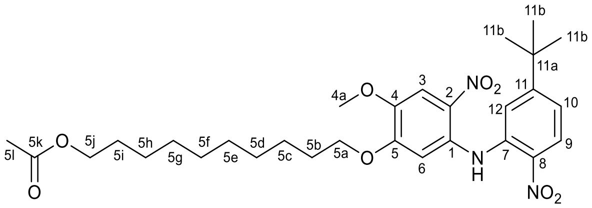

The compound was synthesised from commercially available 2-bromo-4-(*tert*-butyl)-1-nitrobenzene and **6b**. Received orange viscous oil. Yield = 553 mg (99 %).

^{1}H NMR (300 MHz, Chloroform-d) δ: 11.08 (s, 1H, H_{NH}), 7.98 (d, J = 8.9 Hz, 1H, H_9), 7.56 (s, 1H, H_3), 7.53 (d, J = 2.0 Hz, 1H, H_{12}), 7.01 (dd, J = 8.9, 2.0 Hz, 1H, H_{10}), 6.94 (s, 1H, H_6), 3.95 (t, J = 6.8 Hz, 2H, H_{5j}), 3.89 (t, J = 6.8 Hz, 2H, H_{5a}), 3.84 (s, 3H, H_{4a}), 1.95 (s, 3H, H_{5l}), 1.84 – 1.72 (m, 2H, H_{5b}), 1.58 – 1.47 (m, 2H, H_{5i}), 1.40 – 1.29 (m, 2H, H_{5c}), 1.25 (s, 9H, H_{11b}), 1.30 – 1.15 (m, 10H, $H_{5d,5e,5f,5g,5h}$).

^{13}C{^{1}H} NMR (75 MHz, Chloroform-*d*) δ: 170.9 (C_{5k}), 158.9 (C_5), 154.9 (C_{11}), 144.0 (C_4), 136.9 (C_7), 135.8 (C_8), 133.7 (C_1), 130.1 (C_2), 126.3 (C_9), 119.1 (C_{10}), 116.5 (C_{12}), 107.8 (C_3), 101.6 (C_6), 69.4 (C_{5a}), 64.3 (C_{5j}), 56.2 (C_{4a}), 35.4 (C_{11a}), 30.7 (C_{11b}), 29.2 (C_{5b}), 29.2 (C_{5f}), 29.02 (C_{5e}), 28.97 (C_{5d}), 28.6 (C_{5g}), 28.4 (C_{5i}), 25.7 (C_{5c}), 25.6 (C_{5h}), 20.8 (C_{5l}).

HRMS (ESI) *m/z* Calculated for $C_{29}H_{41}N_3O_8Na$ $[M+Na]^+$: 582.2791, found: 582.2783.

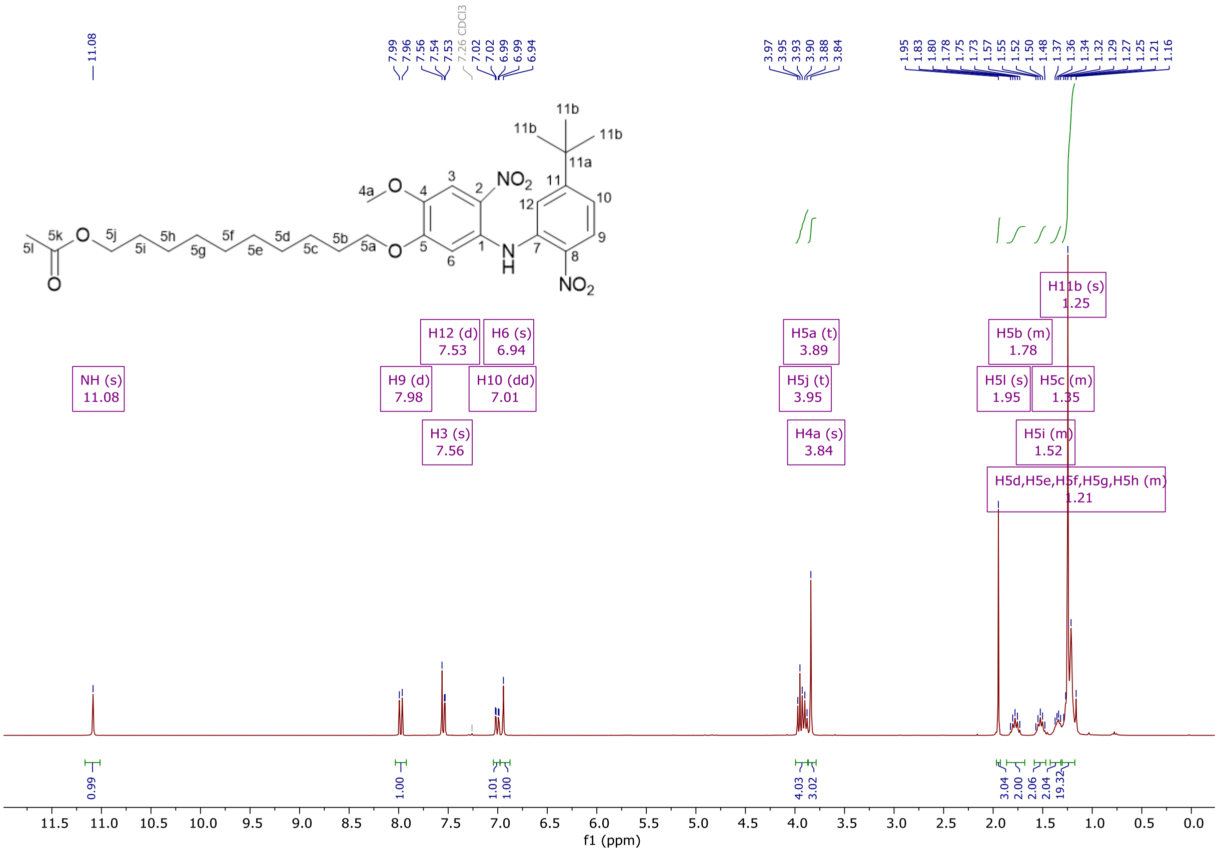

^{1}H NMR (300 MHz, Chloroform-d) spectrum of **3e**.

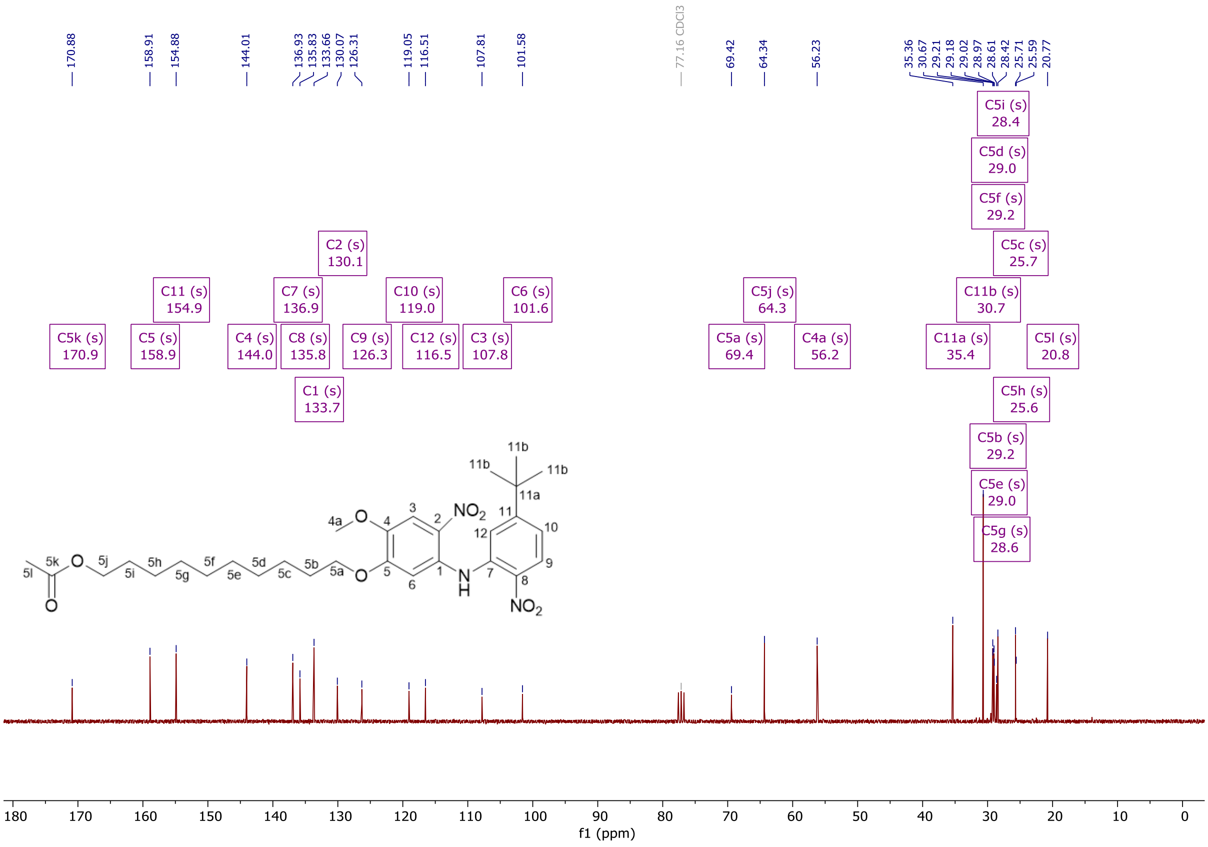

^{13}C{^{1}H} NMR (75 MHz, Chloroform-*d*) spectrum of **3e**.

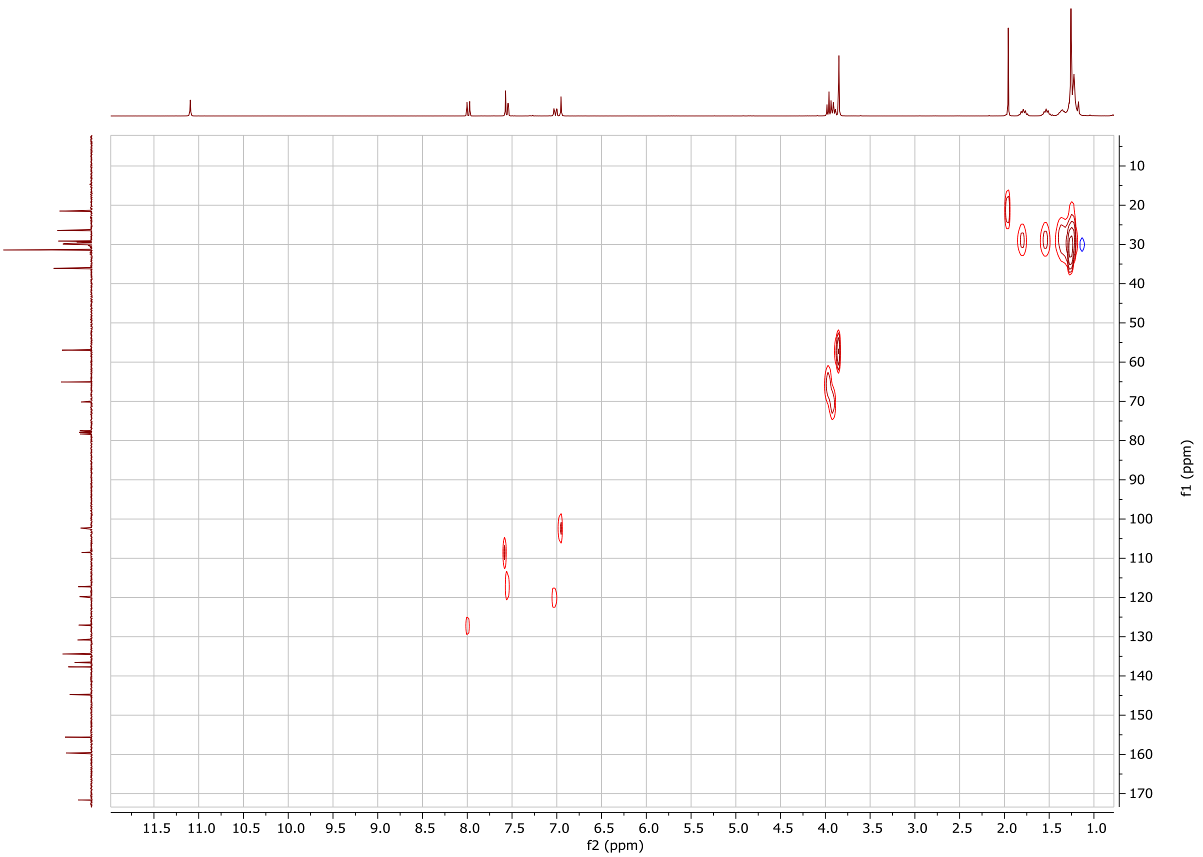

g-HSQC NMR (Chloroform-*d*) spectrum of **3e**.

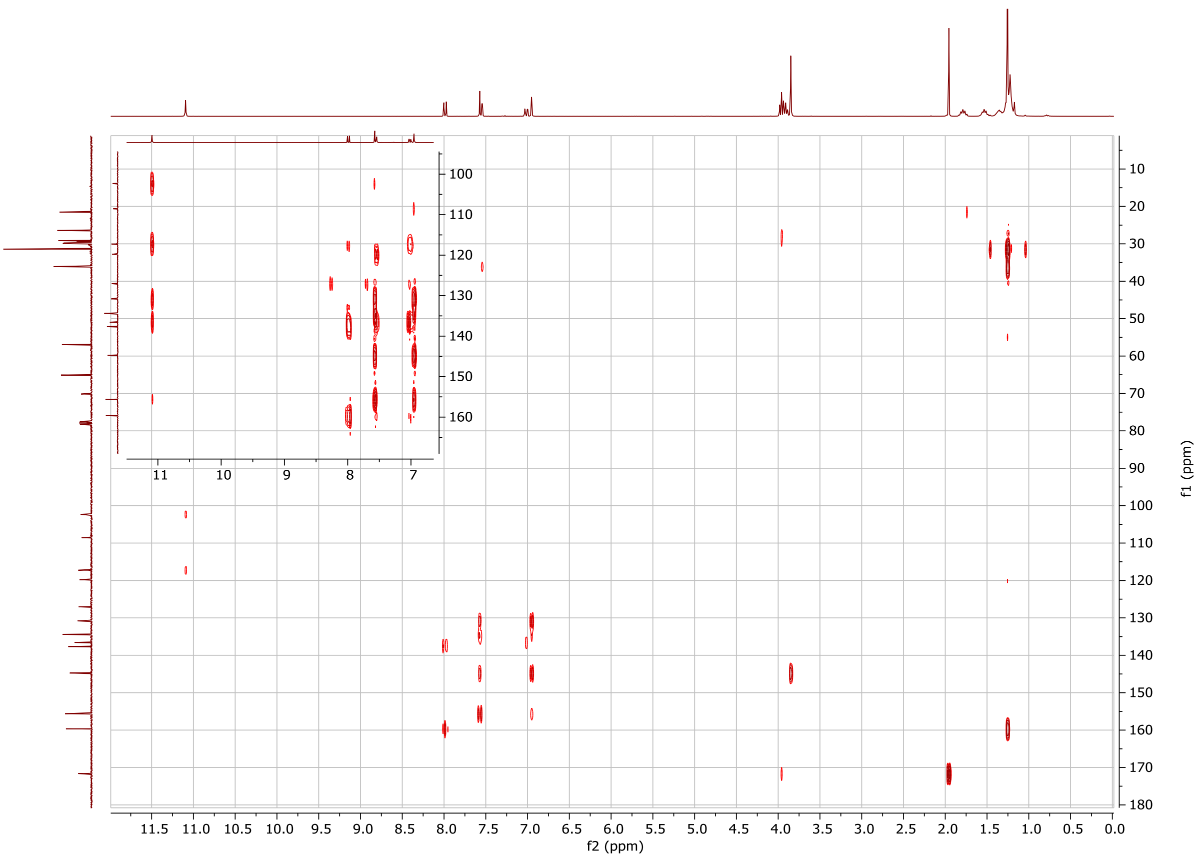

g-HMBC NMR (Chloroform-*d*) spectrum of **3e**.

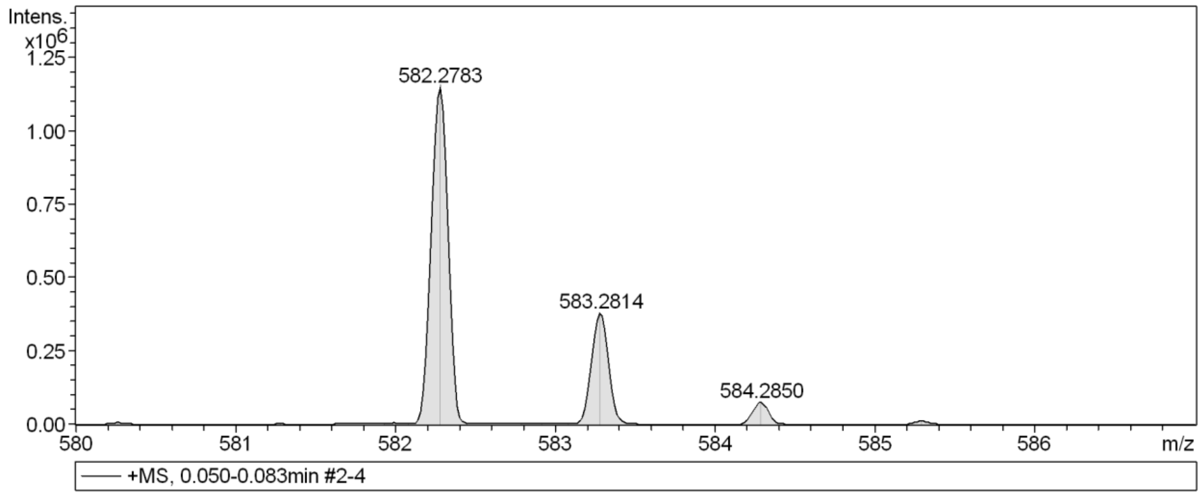

HRMS (ESI+) spectrum of **3e**.

Synthesis of 4,5-bis(hexyloxy)-2-nitro-*N*-(2-nitrophenyl)aniline (3f).

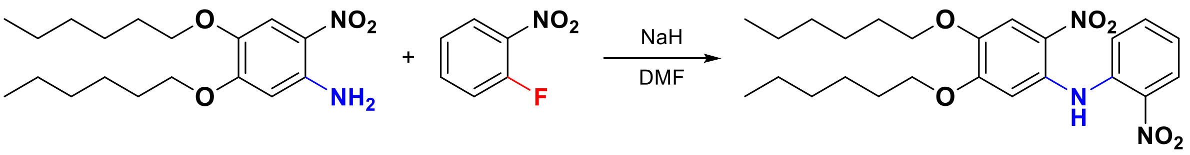

The 4,5-bis(hexyloxy)-2-nitroaniline (500 mg, 1.48 mmol) was placed in the glass vial (20 mL of volume). Dry DMF (10 mL) was added, and the mixture was stirred under argon until the substrate completely dissolved. Then sodium hydride (148 mg, 60 % in mineral oil, 2.5 eq.) was added, and the mixture was stirred under argon for 1 h. The 1-fluoro-2-nitrobenzene (167 µL, 250 mg, 1.2 eq.) was added to the mixture and stirring was continued for the next 20 h. The mixture was poured into water (100 mL) and extracted with DCM (3x 20 mL). Combined organic phases were extracted with brine (50 mL) and dried over anhydrous magnesium sulphate. After removing the solvent on a rotary evaporator, the crude product was purified by flash chromatography (silica gel, DCM).

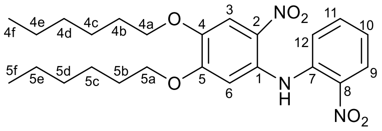

Received orange solid. M.p. = 80.0 – 81.6 °C Yield = 413 mg (61 %).

^{1}H NMR (400 MHz, Chloroform-*d*) δ: 11.04 (s, 1H, H_{NH}), 8.20 (dd, *J* = 8.4, 1.5 Hz, 1H, H_9), 7.68 (s, 1H, H_3), 7.58 – 7.44 (m, 2H, $H_{11,12}$), 7.05 (ddd, *J* = 8.4, 6.7, 1.7 Hz, 1H, H_{10}), 6.92 (s, 1H, H_6), 4.03 (t, *J* = 6.6 Hz, 2H, H_{4a}), 3.95 (t, *J* = 6.6 Hz, 2H, H_{5a}), 1.91 – 1.75 (m, 4H, $H_{4b,5b}$), 1.56 – 1.40 (m, 4H, $H_{4c,5c}$), 1.41 – 1.25 (m, 8H, $H_{4d,4e,5d,5e}$), 0.96 – 0.85 (m, 6H, $H_{4f,5f}$).

^{13}C{^{1}H} NMR (101 MHz, Chloroform-*d*) δ: 155.4 (C_5), 144.3 (C_4), 138.4 (C_7), 138.1 (C_1), 134.8 (C_{11}), 133.2 (C_8), 131.2 (C_2), 127.0 (C_9), 121.4 (C_{12}), 119.5 (C_{10}), 109.6 (C_3), 102.9 (C_6), 69.8 (C_{5a}), 69.6 (C_{4a}), 31.64 (C_{5d}), 31.58 (C_{4d}), 29.1 (C_{5b}), 28.9 C_{4b}), 25.8 (C_{5c}), 25.7 (C_{4c}), 22.72 (C_{5e}), 22.66 (C_{4e}), 14.2 (C_{5f}), 14.1 (C_{4f}).

HRMS (ESI) *m/z* Calculated for $C_{24}H_{33}N_3O_6Na$ $[M+Na]^+$: 482.2267, found: 482.2266.

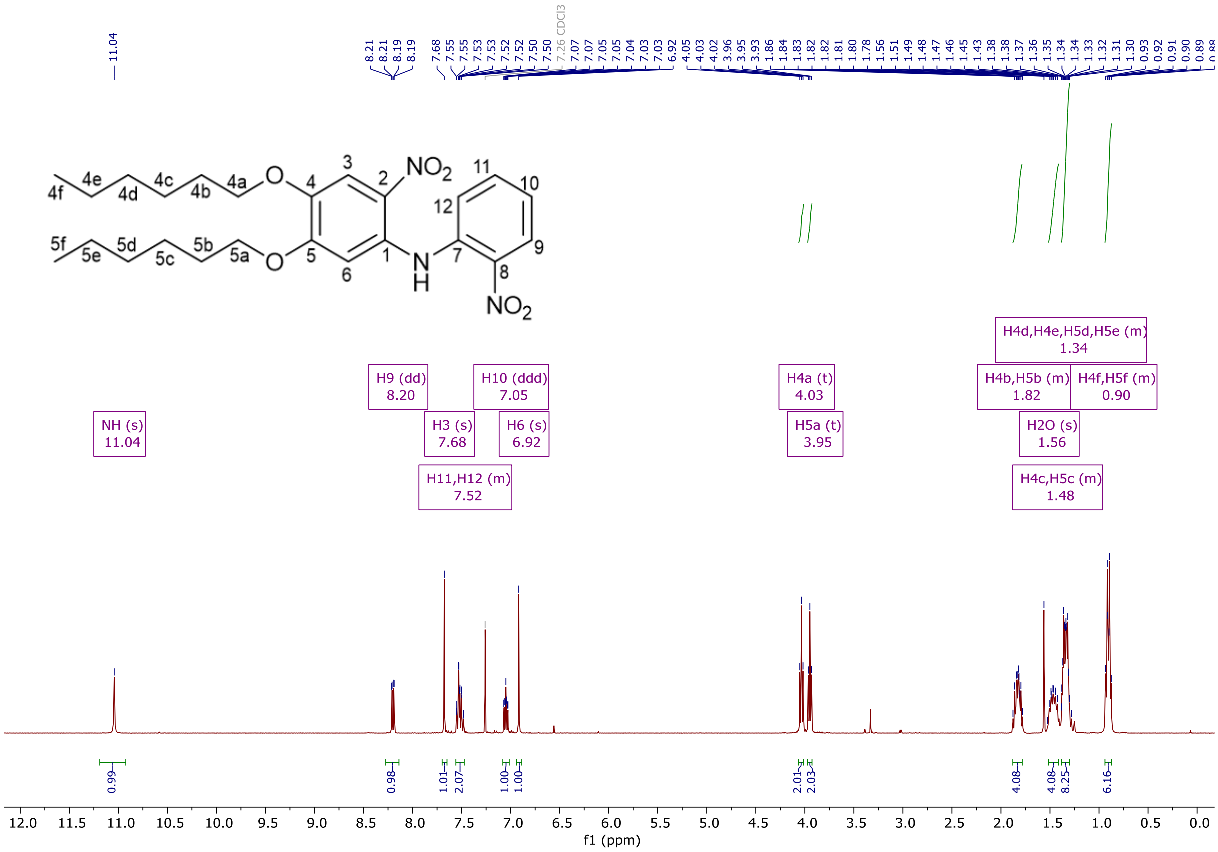

^{1}H NMR (400 MHz, Chloroform-*d*) spectrum of **3f**.

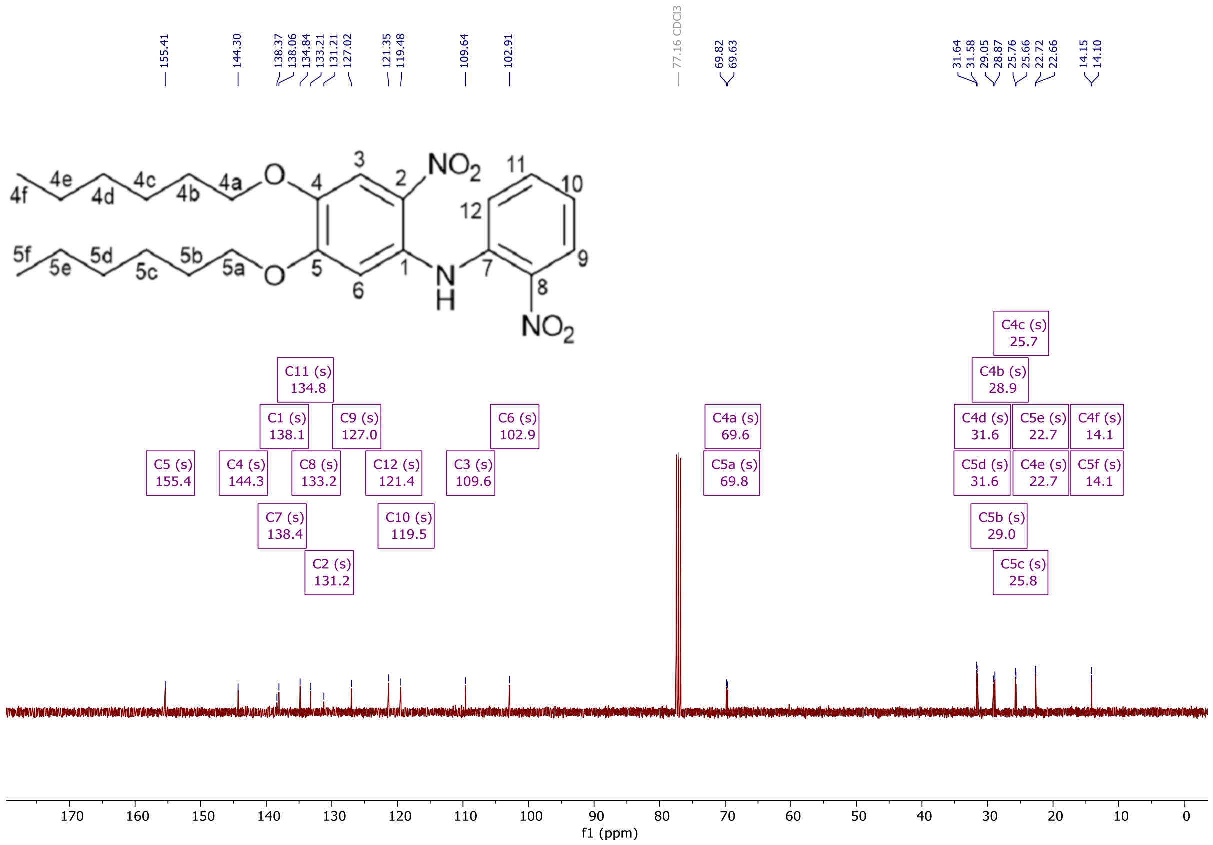

^{13}C{^{1}H} NMR (101 MHz, Chloroform-*d*) spectrum of **3f**.

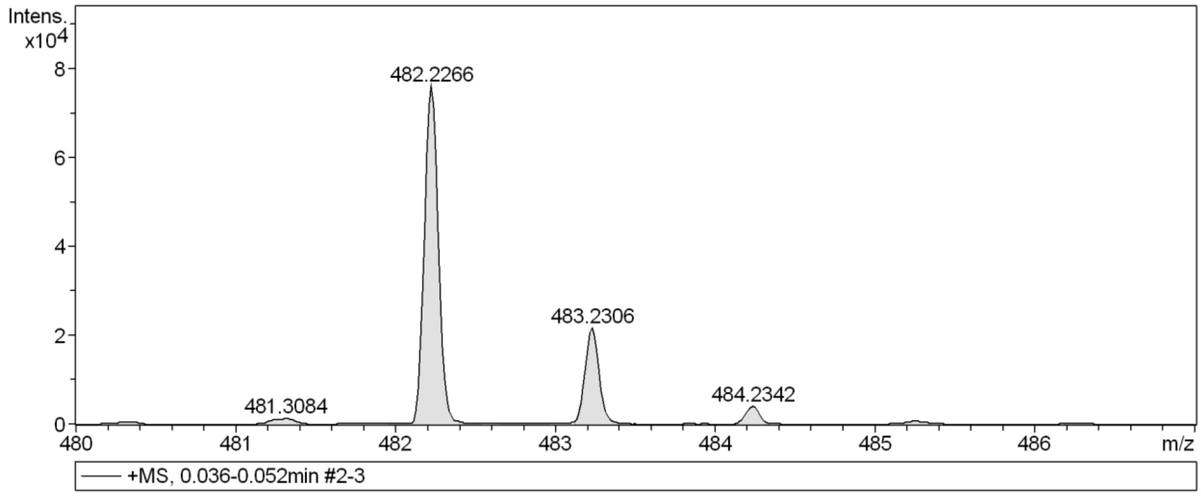

HRMS (ESI+) spectrum of **3f**.

N-alkylation of bis(2-nitrophenyl)amine derivatives (4a – 4f)

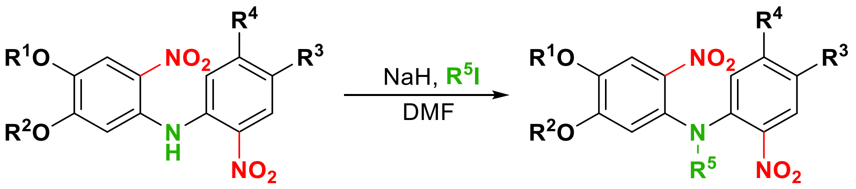

R^1,R^2 = Alkyl, R^3 = -OAlkyl, -CF_3, -H, R^4 = -OAlkyl, -H, -*t*Bu, R^5 = methyl, n-hexyl

In the glass vial (40 mL of volume), substrate – bis(2-nitrophenyl)amine derivative (0.75 mmol) was dissolved in dry DMF (20 mL). To the solution, sodium hydride was added (45 mg, 1.5 eq.) and the mixture was stirred under argon for 1 h. The methyl iodide (187 µL, 423 mg, 4 eq.) or 1-Iodohexane (440 µL, 632 mg, 4 eq. in synthesis of **4c**) was then added to the mixture. After 4 h next portion of methyl iodide (187 µL, 423 mg, 4 eq.) or 1-Iodohexane (440 µL, 632 mg, 4 eq. in synthesis of **4c**) was added and the mixture was stirred overnight under argon. The mixture was poured into the water (150 mL) and extracted with DCM (3x 30 mL). Combined organic phases were extracted with brine (50 mL) and dried over anhydrous magnesium sulphate. The solvent was removed under a vacuum on a rotary evaporator and the crude product was purified by gradient elution column chromatography (silica gel, DCM / MeOH: 0 – 1%).

For the gram scale synthesis (**4b**) reagent amounts were scaled proportionally and the volume of DMF was increased to 30 mL. The rest of the protocol remains the same.

4-ethoxy-*N*-(4-(hexyloxy)-5-isobutoxy-2-nitrophenyl)-5-methoxy-*N*-methyl-2-nitroaniline (4a)

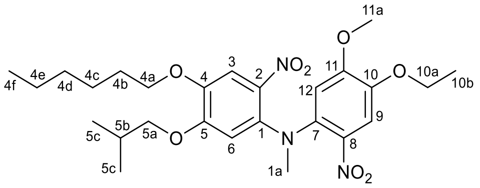

The compound synthesised from **3a**. Received red solid. M.p. = 109.4 – 112.2 °C. Yield = 345 mg (89 %).

^{1}H NMR (300 MHz, Chloroform-*d*) δ: 7.44 (s, 1H, H_3), 7.43 (s, 1H, H_{H9}), 6.56 (s, 1H, H_6), 6.56 (s, 1H, H_{12}), 4.09 (q, *J* = 7.0 Hz, 2H, H_{10a}), 3.97 (t, *J* = 6.5 Hz, 2H, H_{4a}), 3.86 (s, 3H, H_{11a}), 3.73 (d, *J* = 6.5 Hz, 2H, H_{5a}), 3.31 (s, 3H, H_{1a}), 2.19 – 2.07 (m, 1H, H_{5b}), 1.85 – 1.72 (m, 2H, H_{4b}), 1.53 – 1.39 (m, 5H, ($H_{4c,10b}$), 1.40 – 1.25 (m, 4H, $H_{4d,4e}$), 1.02 (d, *J* = 6.7 Hz, 6H, H_{5c}), 0.95 – 0.84 (m, 2H, H_{4f}).

^{13}C{^{1}H} NMR (75 MHz, Chloroform-*d*) δ: 154.4 (C_5), 154.3 (C_{11}), 144.9 (C_{10}), 144.0 (C_4), 138.7 (C_1), 138.4 (C_7), 134.9 (C_8), 134.8 (C_2), 110.8 (C_3), 110.1 (C_9), 108.3 (C_{12}), 106.8 (C_6), 75.6 (C_{5a}), 69.7 (C_{4a}), 65.1 (C_{10a}), 56.5 (C_{11a}), 42.7 (C_{1a}), 31.6 (C_{4d}), 29.2 (C_{4b}), 28.4 (C_{5b}), 25.8 (C_{4c}), 22.7 (C_{4e}), 19.2 (C_{5c}), 14.7 (C_{10b}), 14.1 (C_{4f}).

HRMS (ESI) *m/z* Calculated for $C_{26}H_{37}N_3O_8Na$ $[M+Na]^+$: 542.2478, found: 542.2471.

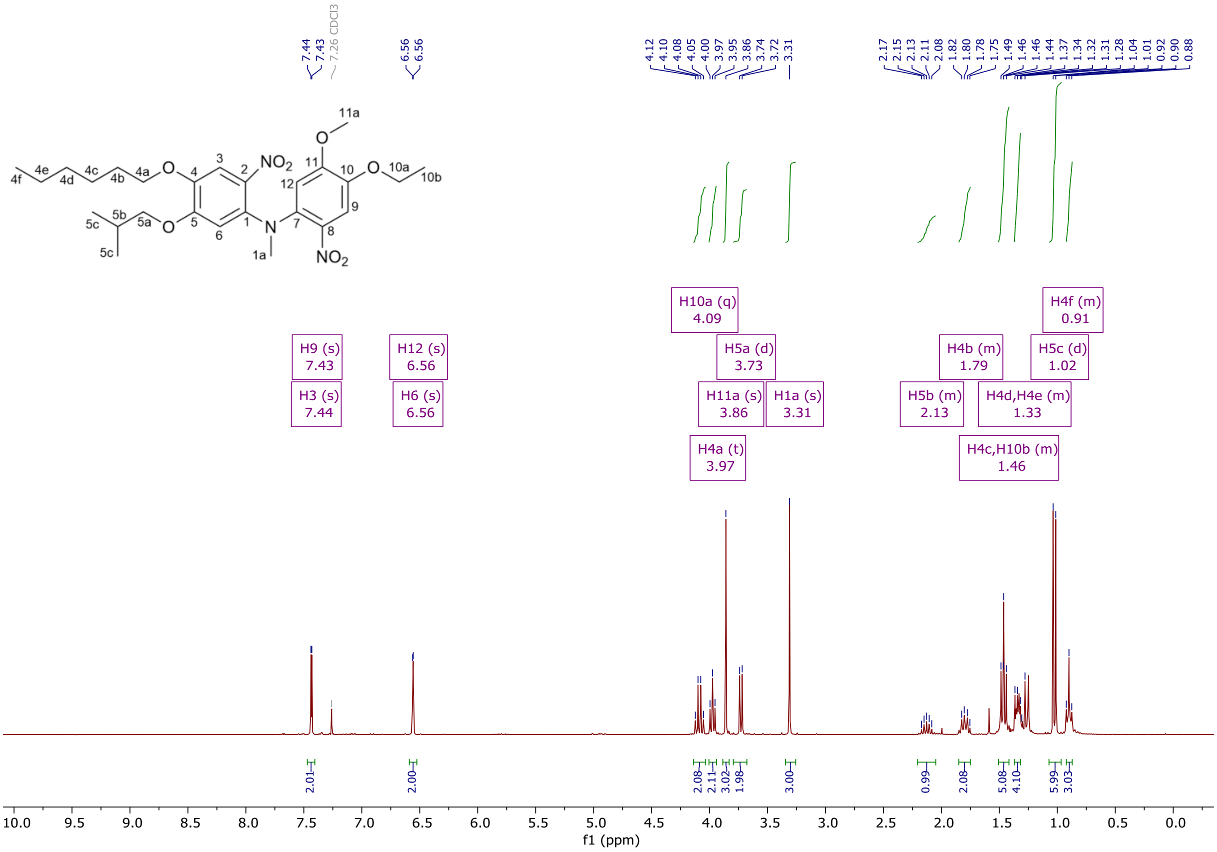

^{1}H NMR (300 MHz, Chloroform-*d*) spectrum of **4a**.

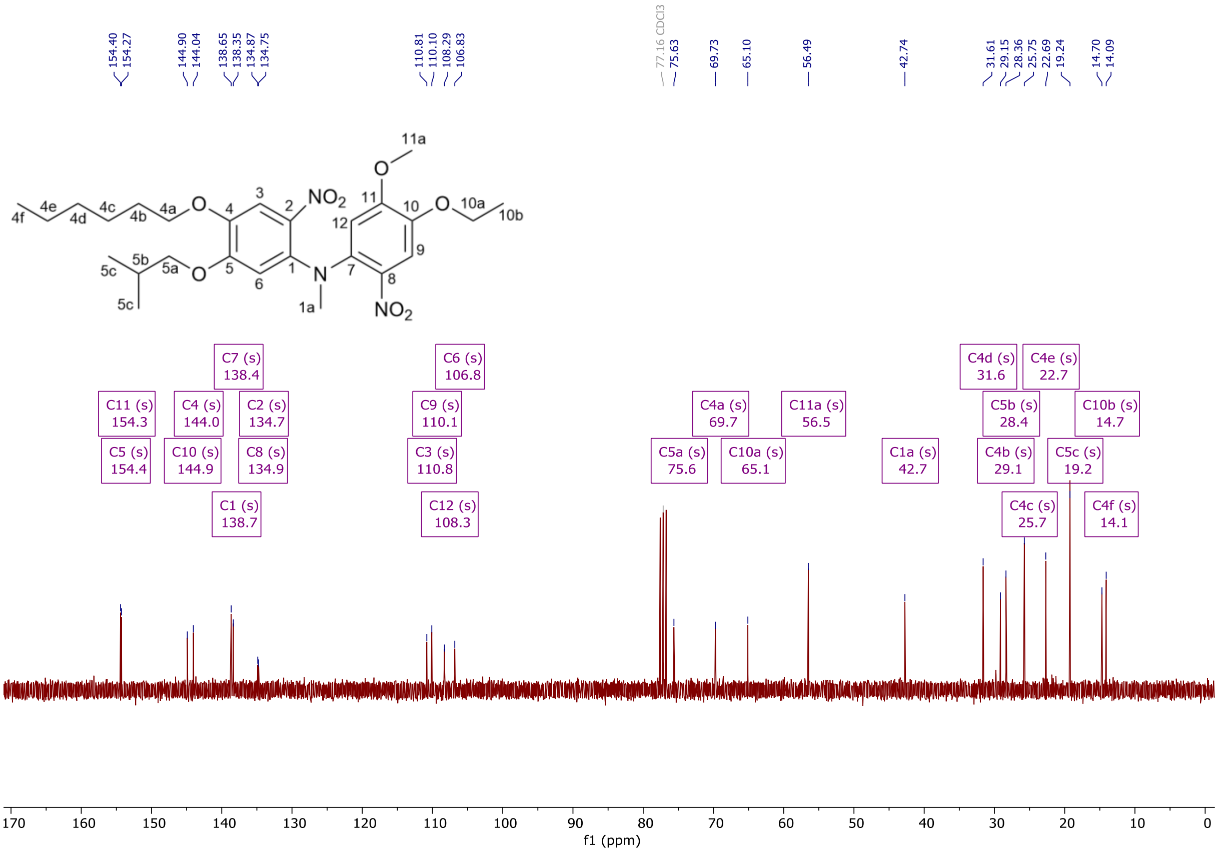

$^{13}C\{^{1}H\}$ NMR (75 MHz, Chloroform-*d*) spectrum of **4a**.

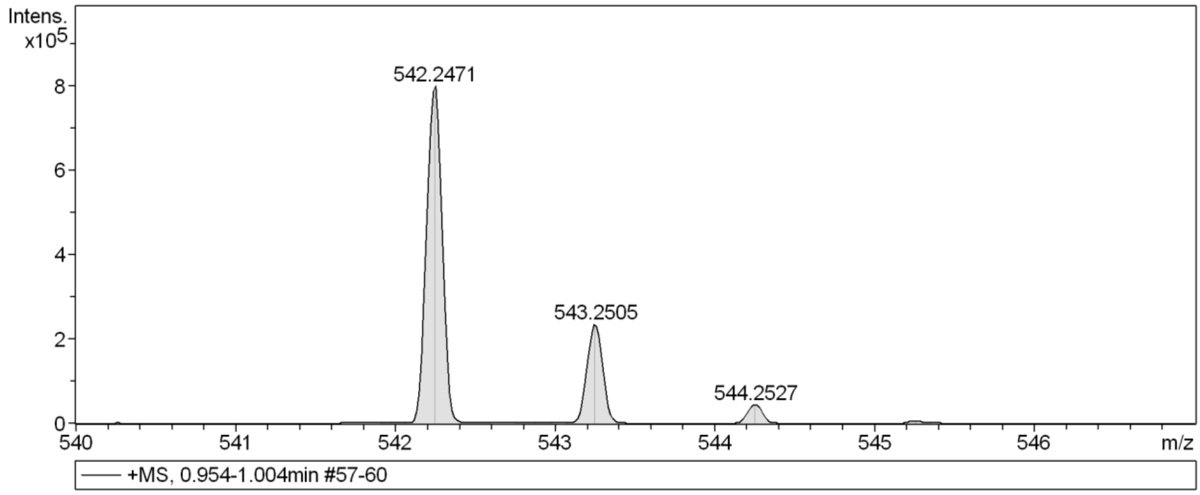

HRMS (ESI+) spectrum of **4a**.

N-(4,5-bis(hexyloxy)-2-nitrophenyl)-4,5-dimethoxy-N-methyl-2-nitroaniline (4b)

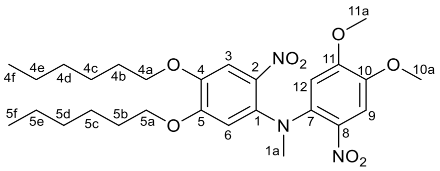

The compound synthesised from *N*-(4,5-bis(hexyloxy)-2-nitrophenyl)-4,5-dimethoxy-2-nitroaniline was obtained as described in the literature.[5] Received red solid. M.p. = 103.8 – 107.5 °C. Yield = 331 mg (83 %).

Gram scale synthesis was performed on 1.20 g of substrate. Yield = 1.10 g (89 %).

^{1}H NMR (300 MHz, Chloroform-*d*) δ: 7.35 (s, 1H, H_{3}), 7.33 (s, 1H, H_{9}), 6.53 (s, 1H, H_{6}), 6.51 (s, 1H, H_{12}), 3.95 – 3.85 (m, 4H, $H_{4a,5a}$), 3.81 – 3.74 (m, 6H, $H_{10a,11a}$), 3.23 (s, 3H, H_{1a}), 1.80 – 1.63 (m, 4H, $H_{4b,5b}$), 1.45 – 1.31 (m, 4H, $H_{4c,5c}$), 1.33 – 1.19 (m, 8H, $H_{4d,4e,5d,4e}$), 0.88 – 0.76 (m, 6H, $H_{4f,5f}$).

^{13}C{^{1}H} NMR (101 MHz, Chloroform-*d*) δ: 154.1 (C_{5}), 153.8 (C_{11}), 144.7 (C_{10}), 144.5 (C_{4}), 138.7 (C_{1}), 138.3 (C_{7}), 134.7 (C_{2}), 134.5 (C_{8}), 110.5 (C_{3}), 108.8 (C_{9}), 108.0 (C_{12}), 106.3 (C_{6}), 69.6 (C_{5a}), 69.4 (C_{4a}), 56.4 (C_{10a}), 56.3 (C_{11a}), 42.6 (C_{1a}), 31.48 (C_{5d}), 31.45 (C_{4d}), 29.0 (C_{5b}), 28.8 (C_{4b}), 25.6 (C_{5c}), 25.5 (C_{4c}), 22.6 (C_{5e}), 22.5 (C_{4e}), 13.98 (C_{5f}), 13.95 (C_{4f}).

HRMS (ESI) *m/z* Calculated for $C_{27}H_{39}N_{3}O_{8}Na$ $[M+Na]^{+}$: 556.2635, found: 556.2625.

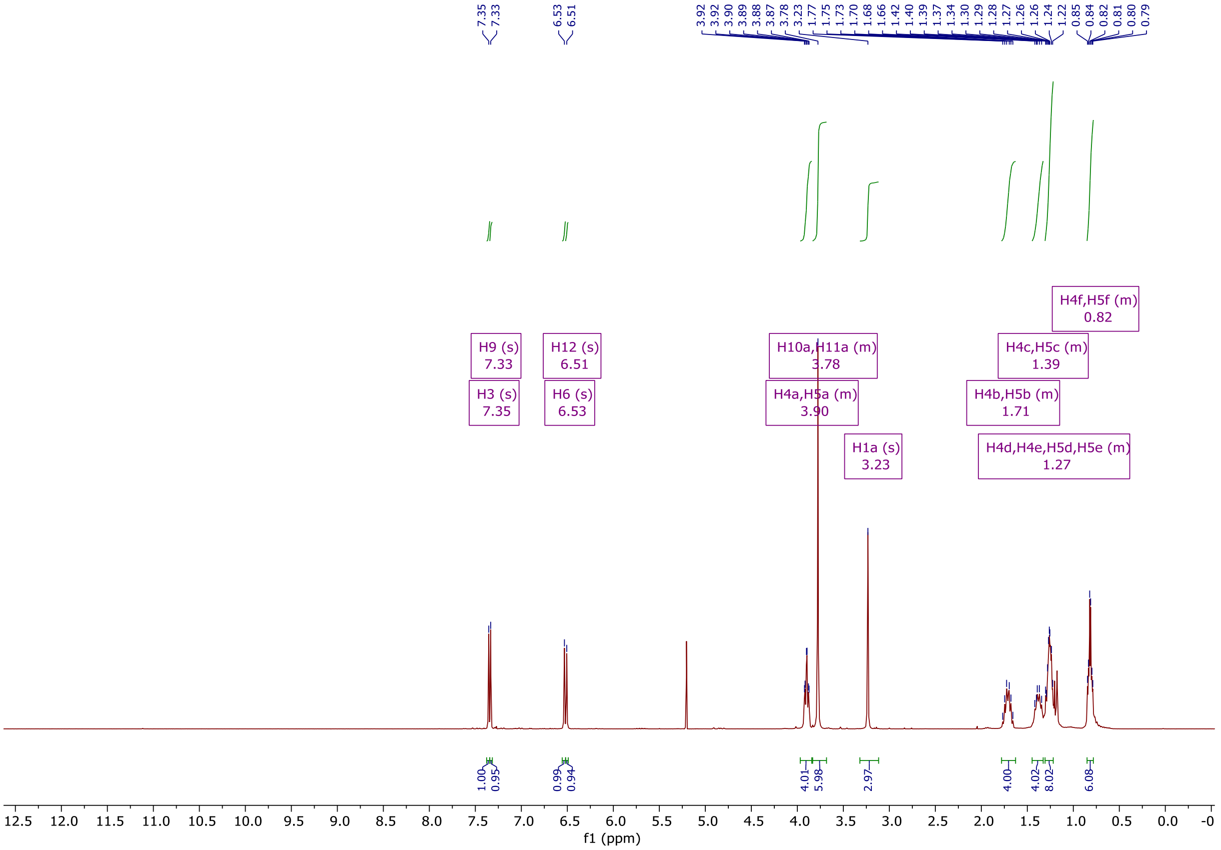

^{1}H NMR (300 MHz, Chloroform-*d*) spectrum of **4b**.

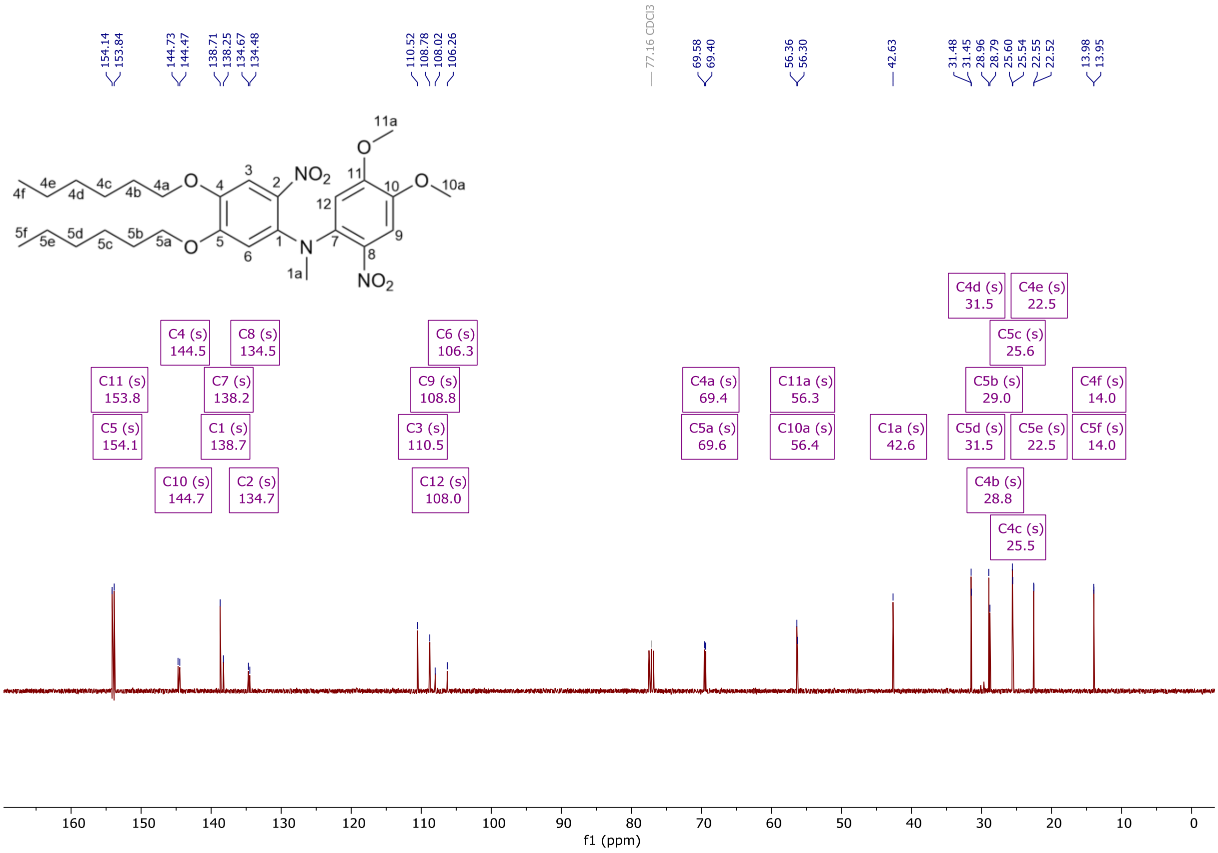

$^{13}C\{^{1}H\}$ NMR (101 MHz, Chloroform-*d*) spectrum of **4b**.

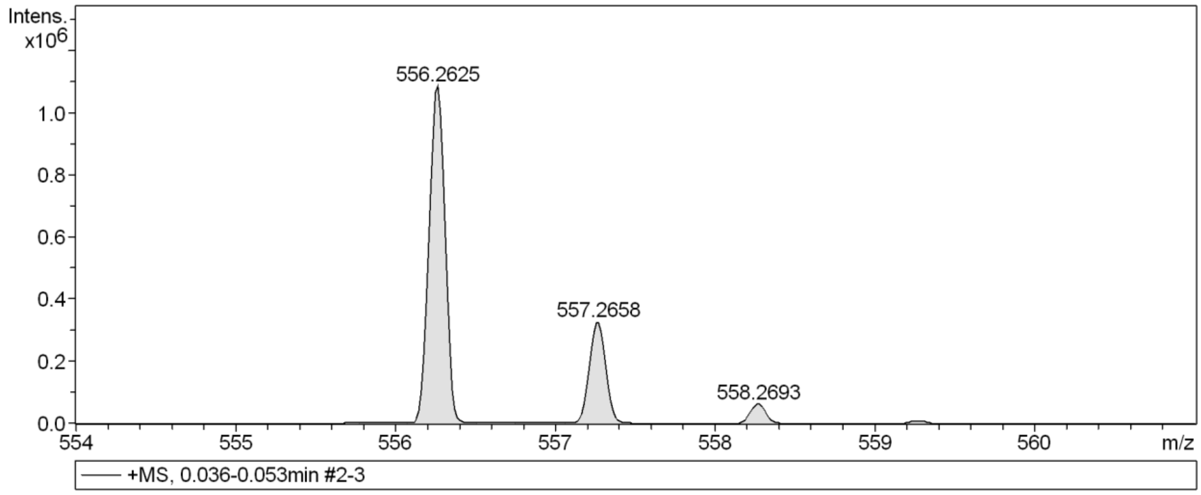

HRMS (ESI+) spectrum of **4b**.

_N_-(4,5-bis(hexyloxy)-2-nitrophenyl)-_N_-hexyl-4,5-dimethoxy-2-nitroaniline (4c)

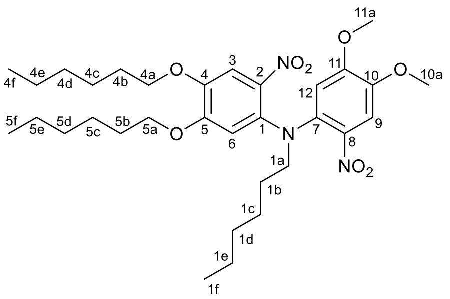

Compound synthesised from _N_-(4,5-bis(hexyloxy)-2-nitrophenyl)-4,5-dimethoxy-2-nitroaniline obtained as described in the literature.[5] Received orange solid. M.p. = 58.6 – 60.1 °C. Yield = 218 mg (48 %).

^{1}H NMR (400 MHz, Chloroform-_d_) δ 7.35 (s, 1H, H_{3}), 7.35 (s, 1H, H_{9}), 6.63 (s, 1H, H_{6}), 6.63 (s, 1H, H_{12}), 4.01 (t, _J_ = 6.6 Hz, 2H, H_{4a}), 3.96 (t, _J_ = 6.6 Hz, 2H, H_{5a}), 3.89 (s, 3H, H_{10a}), 3.87 (s, 3H, H_{11a}), 3.71 – 3.64 (m, 2H, H_{1a}), 1.89 – 1.73 (m, 4H, $H_{4b,5b}$), 1.76 – 1.65 (m, 2H, H_{1b}), 1.49 – 1.43 (m, 4H, $H_{4c,5c}$), 1.39 – 1.25 (m, 14H, $H_{4d,4e,5d,5e,1c,1d,1e}$), 0.95 – 0.82 (m, 9H, $H_{4f,5f,1f}$).

^{13}C{^{1}H} NMR (101 MHz, Chloroform-_d_) δ: 153.8 (C_{11}), 153.6 (C_{5}), 144.8 (C_{10}), 144.6 (C_{4}), 137.7 (C_{7}), 137.2 (C_{1}), 135.5 (C_{2}), 135.4 (C_{8}), 110.4 (C_{3}), 109.4 (C_{9}), 108.7 (C_{6}), 107.8 (C_{12}), 69.6 (C_{5a}), 69.5 (C_{4a}), 56.42 (C_{10a}), 56.39 (C_{11a}), 55.0 (C_{1a}), 31.61 ($C_{4d,5d}$), 31.59 (C_{1d}), 29.1 (C_{5b}), 28.9 (C_{4b}), 27.8 (C_{1b}), 26.8 (C_{1c}), 25.72 (C_{5c}), 25.70 (C_{4c}), 22.68 ($C_{4e,5e}$), 22.67 (C_{1e}), 14.10 (C_{1f}), 14.08 ($C_{4f,5f}$).

HRMS (ESI) _m/z_ Calculated for $C_{32}H_{49}N_{3}O_{8}Na$ $[M+Na]^{+}$: 626.3417, found: 626.3413.

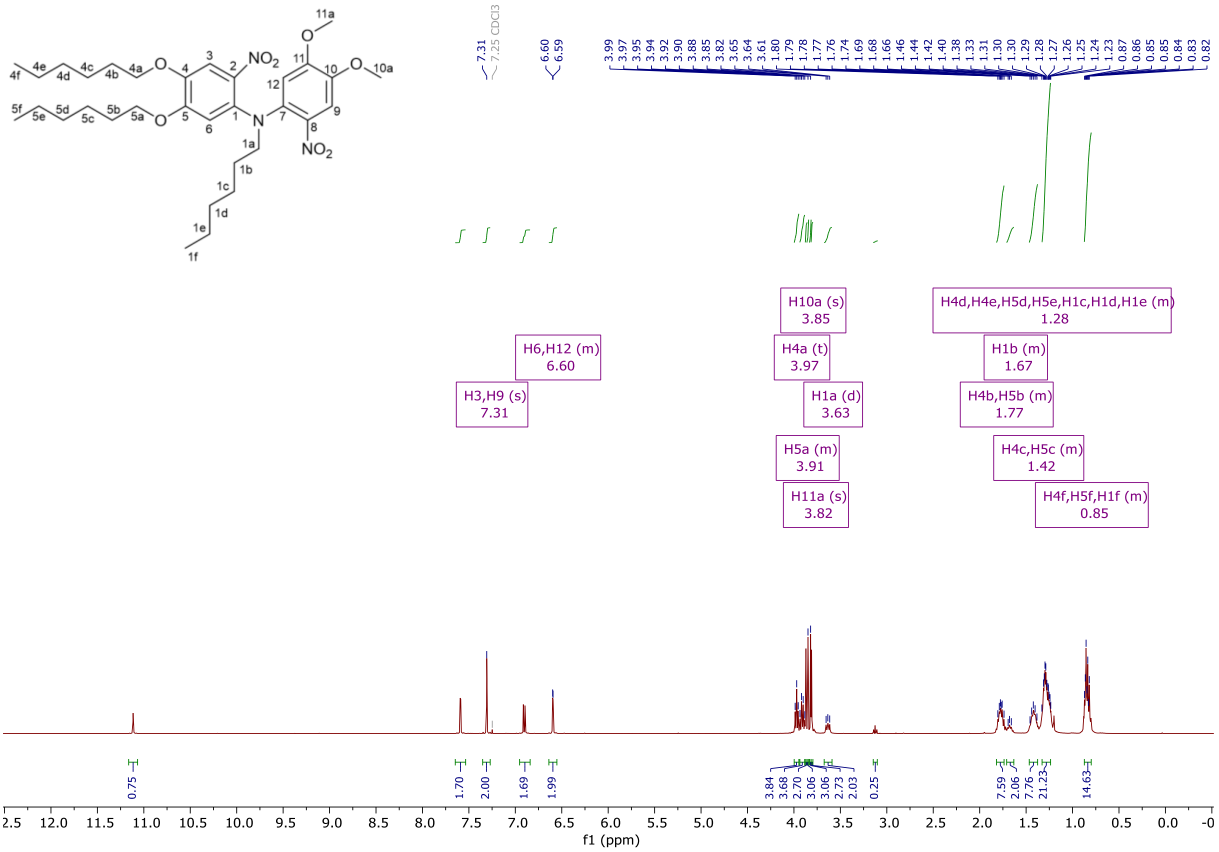

^{1}H NMR (400 MHz, Chloroform-_d_) spectrum of **4c**.

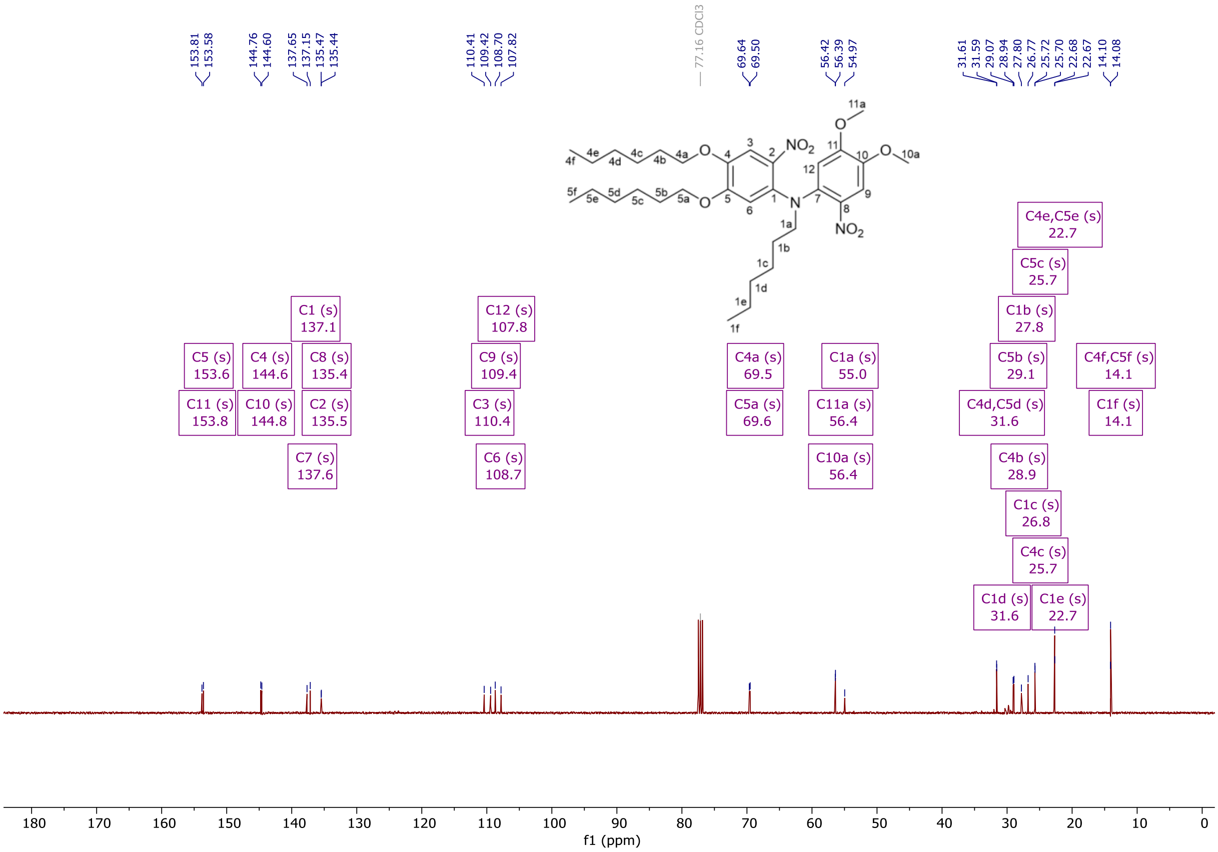

$^{13}C\{^{1}H\}$ NMR (101 MHz, Chloroform-*d*) spectrum of **4c**.

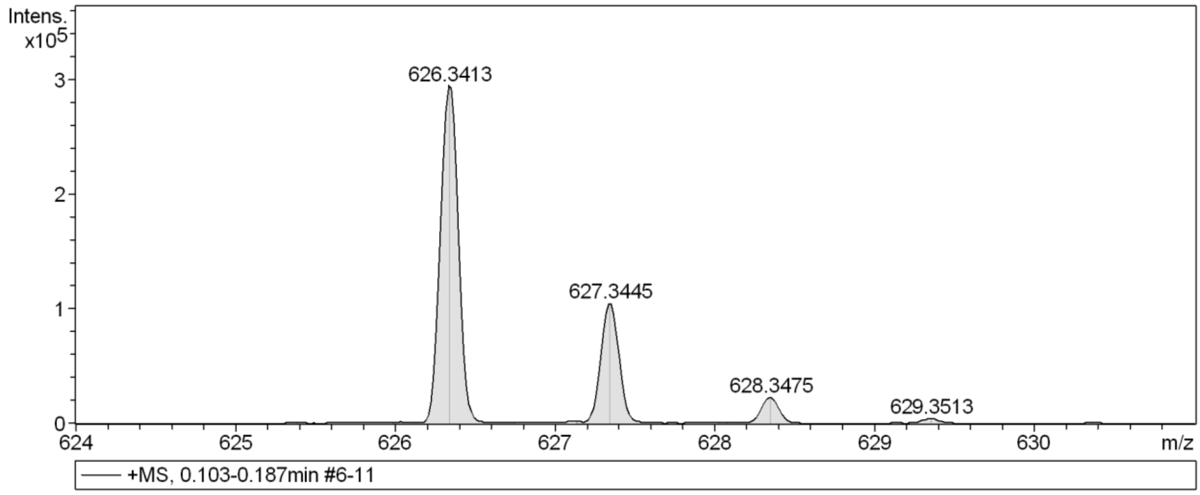

HRMS (ESI+) spectrum of **4c**.

4-(hexyloxy)-5-isobutoxy-*N*-methyl-2-nitro-*N*-(2-nitro-4-(trifluoromethyl)phenyl)aniline (4d)

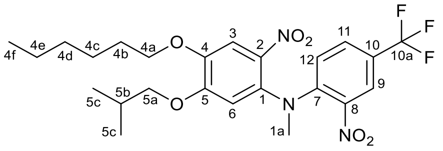

The compound was synthesised from 4-(hexyloxy)-5-isobutoxy-2-nitro-*N*-(2-nitro-4-(trifluoromethyl)phenyl)aniline obtained as described in the literature.[5]. Received yellow solid. M.p. = 73.0 – 75.2 °C. Yield = 318 mg (83 %).

^{1}H NMR (300 MHz, Chloroform-*d*) δ: 7.85 (d, *J* = 1.4 Hz, 1H, H_9), 7.64 (dd, *J* = 9.2, 1.9 Hz, 1H, H_{11}), 7.58 (s, 1H, H_3), 7.13 (d, *J* = 8.9 Hz, 1H, H_{12}), 6.57 (s, 1H, H_6), 4.04 (t, *J* = 6.4 Hz, 2H, H_{4a}), 3.73 (d, *J* = 6.5 Hz, 2H, H_{5a}), 3.34 (s, 3H, H_{1a}), 2.21 – 2.06 (m, 1H, H_{5b}), 1.92 – 1.76 (m, 2H, H_{4b}), 1.56 – 1.44 (m, 2H, H_{4c}), 1.42 – 1.30 (m, 4H, $H_{4d,4e}$), 1.03 (d, *J* = 6.7 Hz, 6H, H_{5c}), 0.96 – 0.86 (m, 3H, H_{4f}).

^{13}C{^{1}H} NMR (75 MHz, Chloroform-*d*) δ: 154.8 (C_5), 147.9 (C_4), 144.4 (C_7), 139.0 (C_8), 137.2 (C_1), 135.1 (C_2), 129.7 – 129.5 (m, C_{11}), 129.4 – 129.3 (m, C_{10}), 125.2, 121.6, 121.1 (C_{10a}), 124.2 – 124.0 (m, C_{12}), 120.0 (C_9), 112.0 (C_3), 110.1 (C_6), 75.9 (C_{5a}), 69.7 (C_{4a}), 43.0 (C_{1a}), 31.6 (C_{4d}), 29.0 (C_{4b}), 28.3 (C_{5b}), 25.7 (C_{4c}), 22.7 (C_{4e}), 19.2 (C_{5c}), 14.1 (C_{4f}).

^{19}F NMR (282 MHz, Chloroform-*d*) δ: -65.96 (F_{CF3}). [relative chemical shift – no reference]

HRMS (ESI) *m/z* Calculated for $C_{24}H_{30}F_3N_3O_6Na$ $[M+Na]^+$: 536.1984, found: 536.1977.

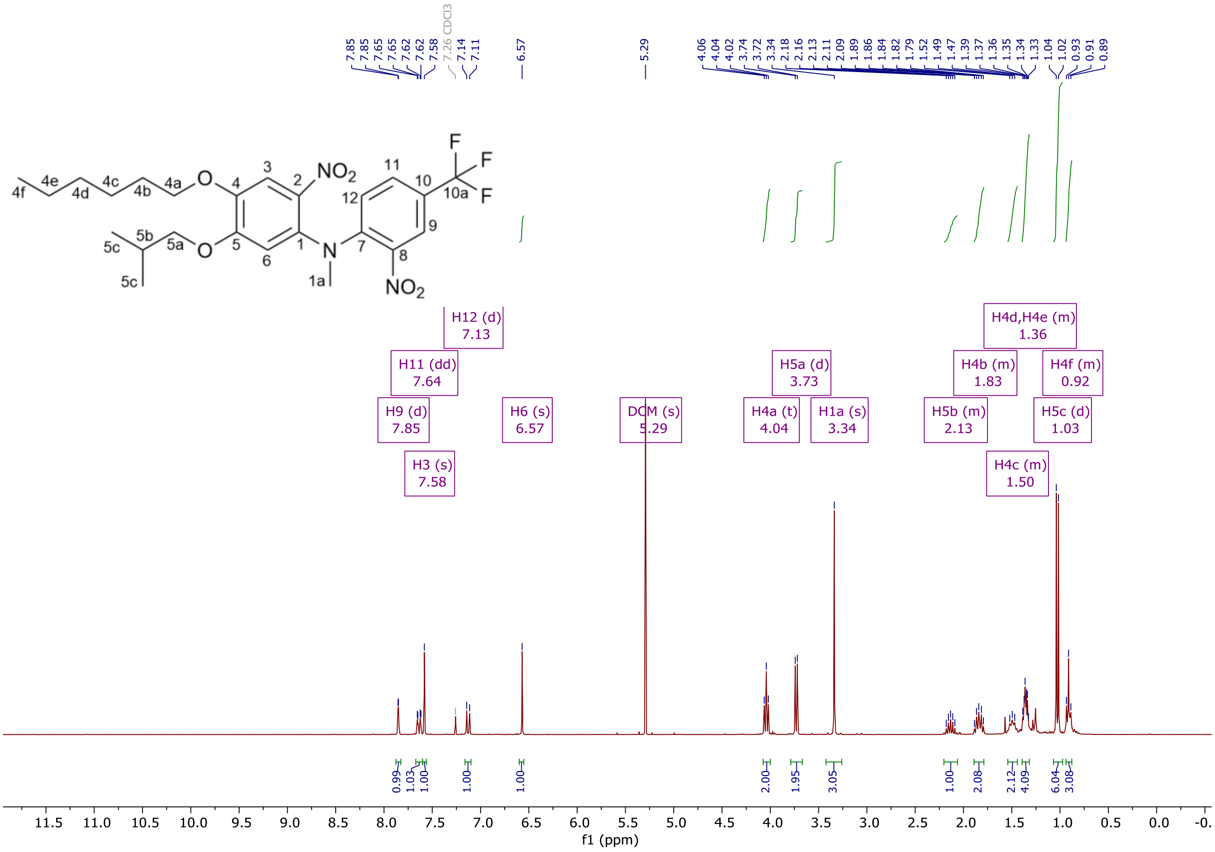

^{1}H NMR (300 MHz, Chloroform-*d*) spectrum of **4d**.

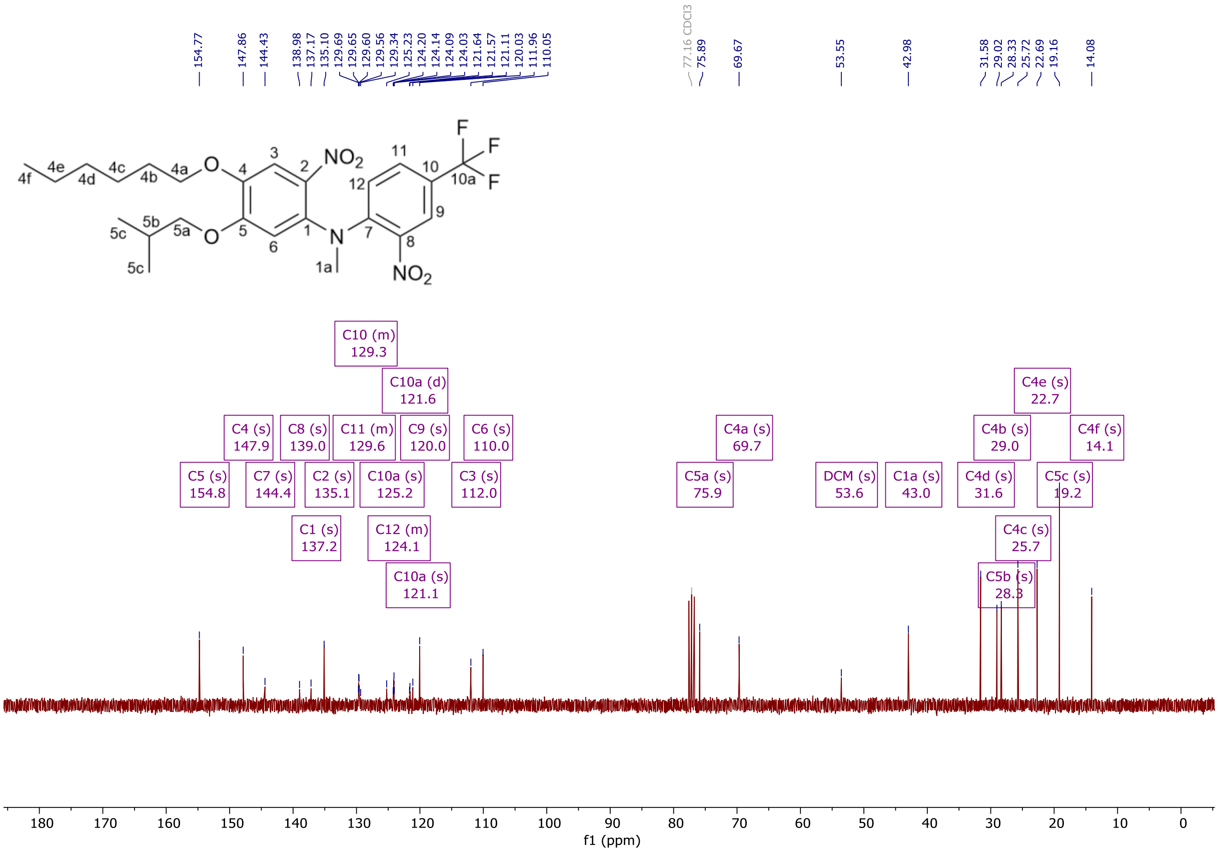

$^{13}C\{^{1}H\}$ NMR (75 MHz, Chloroform-*d*) spectrum of **4d**.

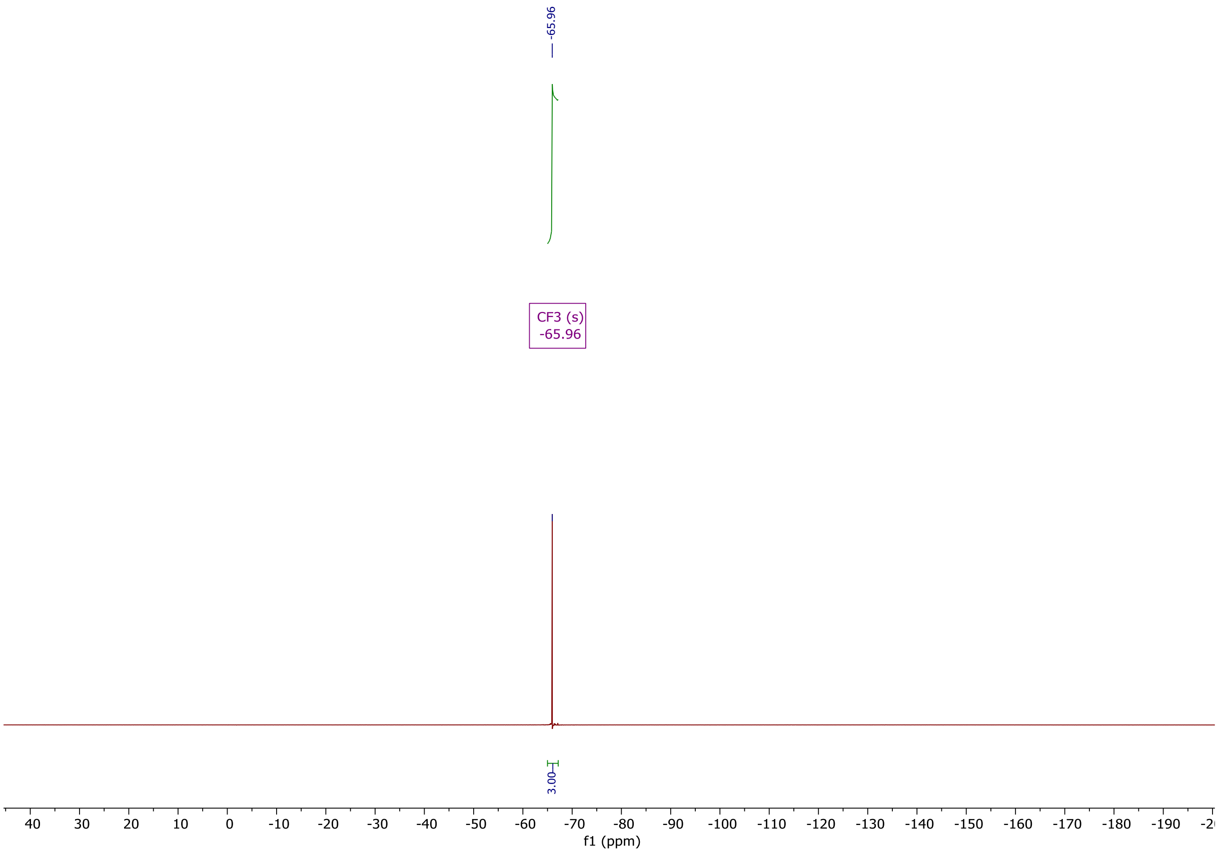

^{19}F NMR (282 MHz, Chloroform-*d*) spectrum of **4d**.

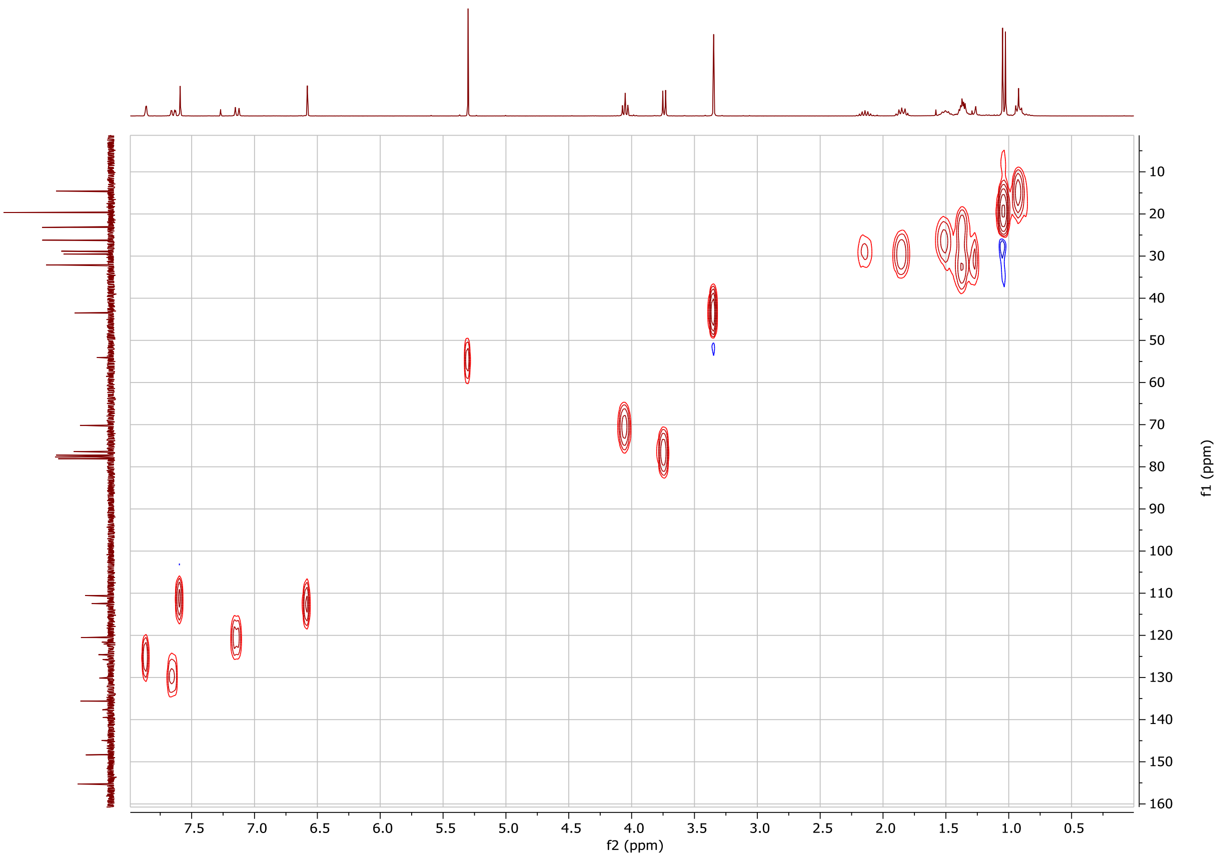

g-HSQC NMR (Chloroform-*d*) spectrum of **4d**.

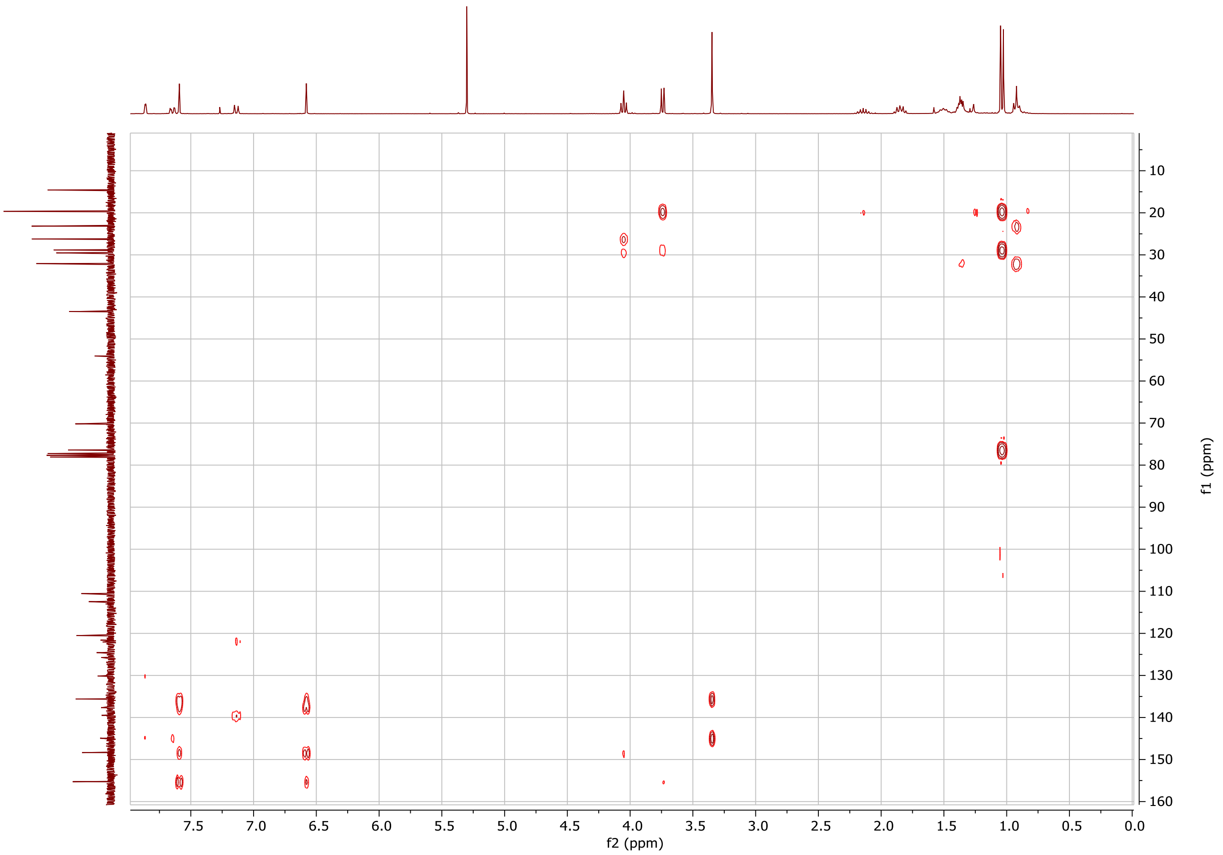

g-HMBC NMR (Chloroform-*d*) spectrum of **4d**.

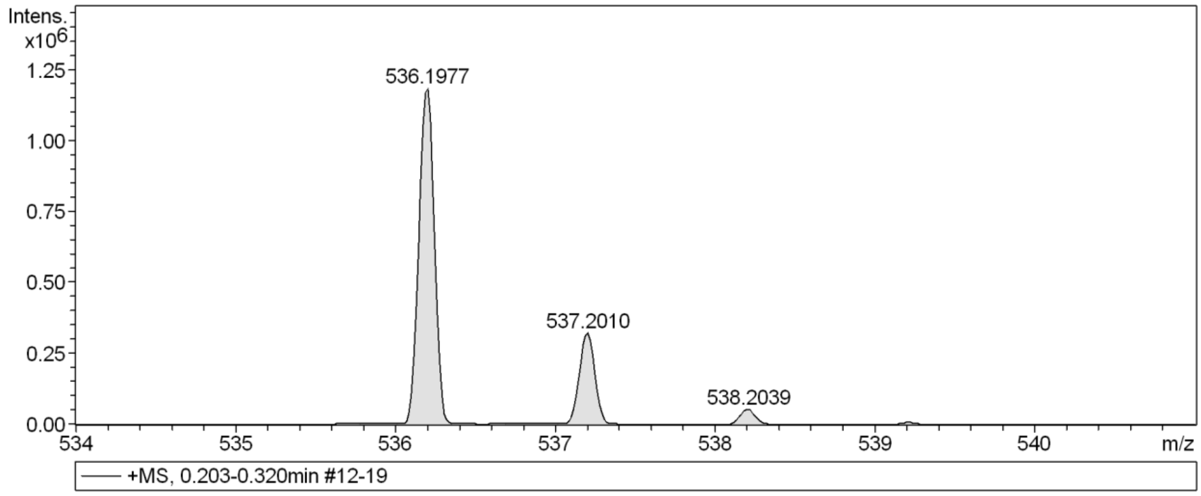

HRMS (ESI+) spectrum of **4d**.

***N*-(5-(*tert*-butyl)-2-nitrophenyl)-4-(hexyloxy)-5-isobutoxy-*N*-methyl-2-nitroaniline (4e)**

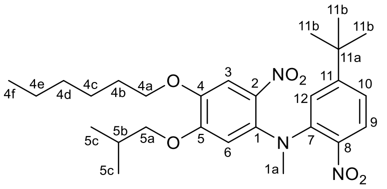

The compound was synthesised from *N*-(5-(*tert*-butyl)-2-nitrophenyl)-4-(hexyloxy)-5-isobutoxy-2-nitroaniline obtained as described in the literature.[5]. Received yellow solid. M.p. = 127.5 – 129.0 °C. Yield = 282 mg (75 %).

^{1}H NMR (300 MHz, Chloroform-*d*) δ: 7.66 (d, *J* = 8.6 Hz, 1H, H_9), 7.49 (s, 1H, H_3), 7.11 (d, *J* = 1.9 Hz, 1H, H_{12}), 7.03 (dd, *J* = 8.6, 1.9 Hz, 1H, H_{10}), 6.57 (s, 1H, H_6), 4.00 (t, *J* = 6.5 Hz, 2H, H_{4a}), 3.73 (d, *J* = 6.5 Hz, 2H, H_{5a}), 3.34 (s, 3H, H_{1a}), 2.20 – 2.02 (m, 1H, H_{5b}), 1.89 – 1.74 (m, 2H, H_{4b}), 1.55 – 1.41 (m, 2H, H_{4c}), 1.41 – 1.30 (m, 4H, $H_{4d,4e}$), 1.31 (s, 9H, H_{11b}), 1.02 (d, *J* = 6.7 Hz, 6H, H_{5c}), 0.96 – 0.85 (m, 3H, H_{4f}).

^{13}C{^{1}H} NMR (75 MHz, Chloroform-*d*) δ: 157.7 (C_{11}), 154.4 (C_5), 145.9 (C_4), 142.1 (C_7), 139.3 (C_8), 137.6 (C_1), 136.0 (C_2), 126.4 (C_9), 119.1 ($C_{10,12}$), 110.6 (C_3), 109.8 (C_6), 75.7 (C_{5a}), 69.7 (C_{4a}), 42.6 (C_{1a}), 35.4 (C_{11a}), 31.6 (C_{4d}), 31.1 (C_{11b}), 29.1 (C_{4b}), 28.3 (C_{5b}), 25.8 (C_{4c}), 22.7 (C_{4e}), 19.2 (C_{5c}), 14.1 (C_{4f}).

HRMS (ESI) *m/z* Calculated for $C_{27}H_{39}N_3O_6Na$ $[M+Na]^+$: 524.2737, found: 524.2728.

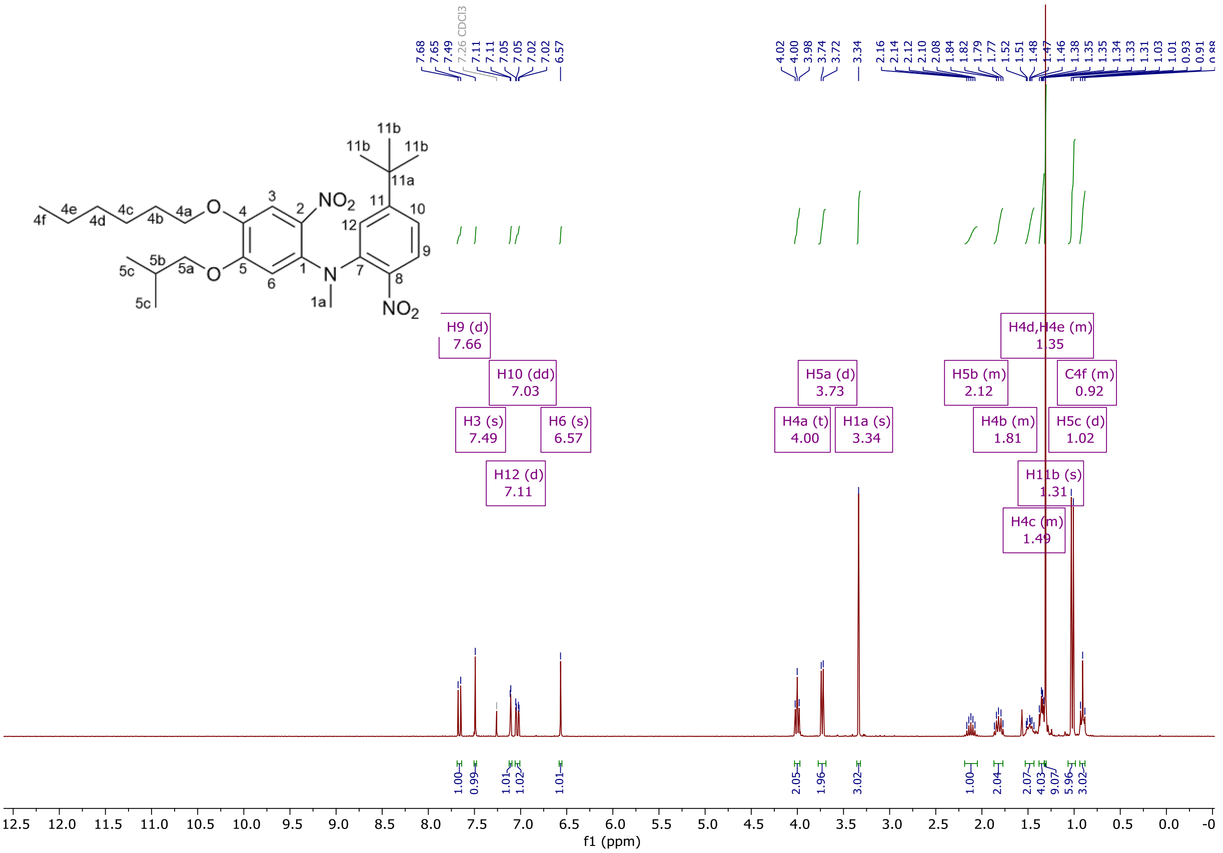

^{1}H NMR (300 MHz, Chloroform-*d*) spectrum of **4e**.

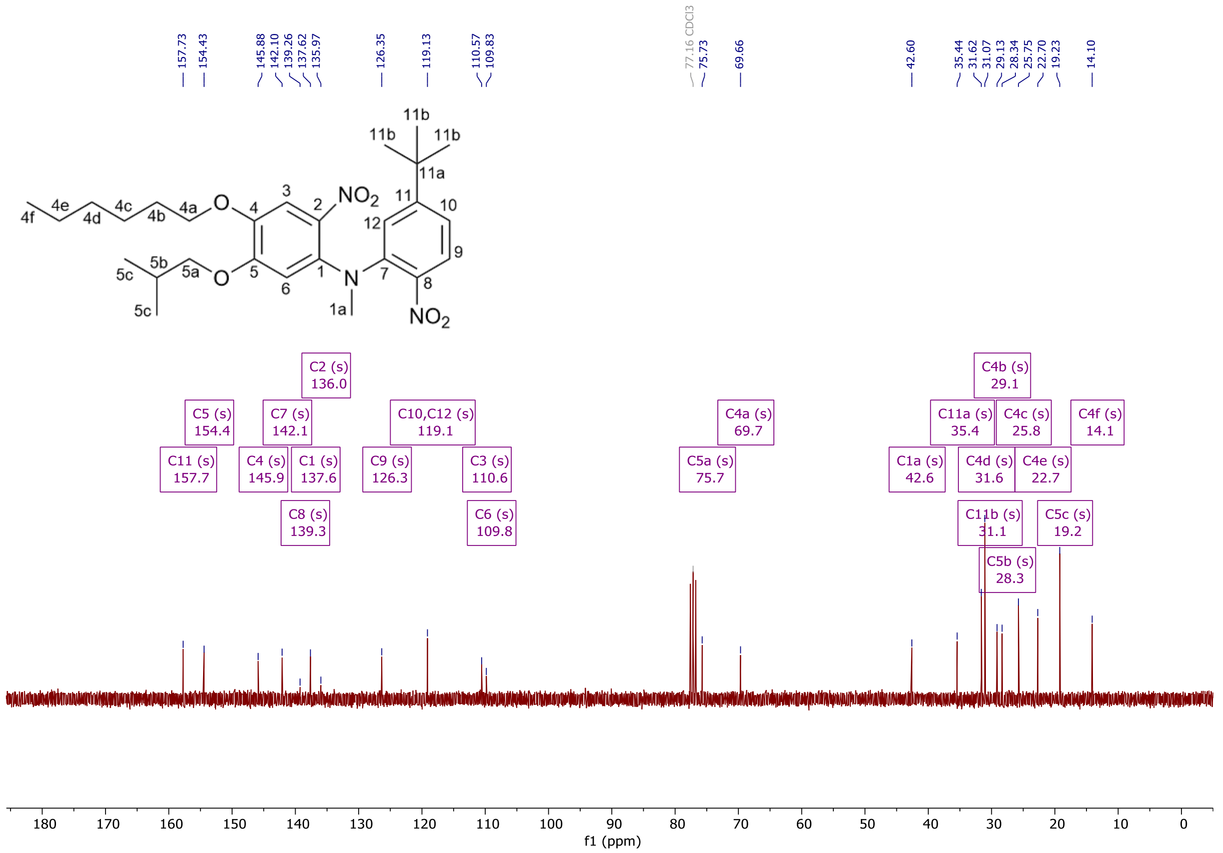

^{13}C{^{1}H} NMR (75 MHz, Chloroform-*d*) spectrum of **4e**.

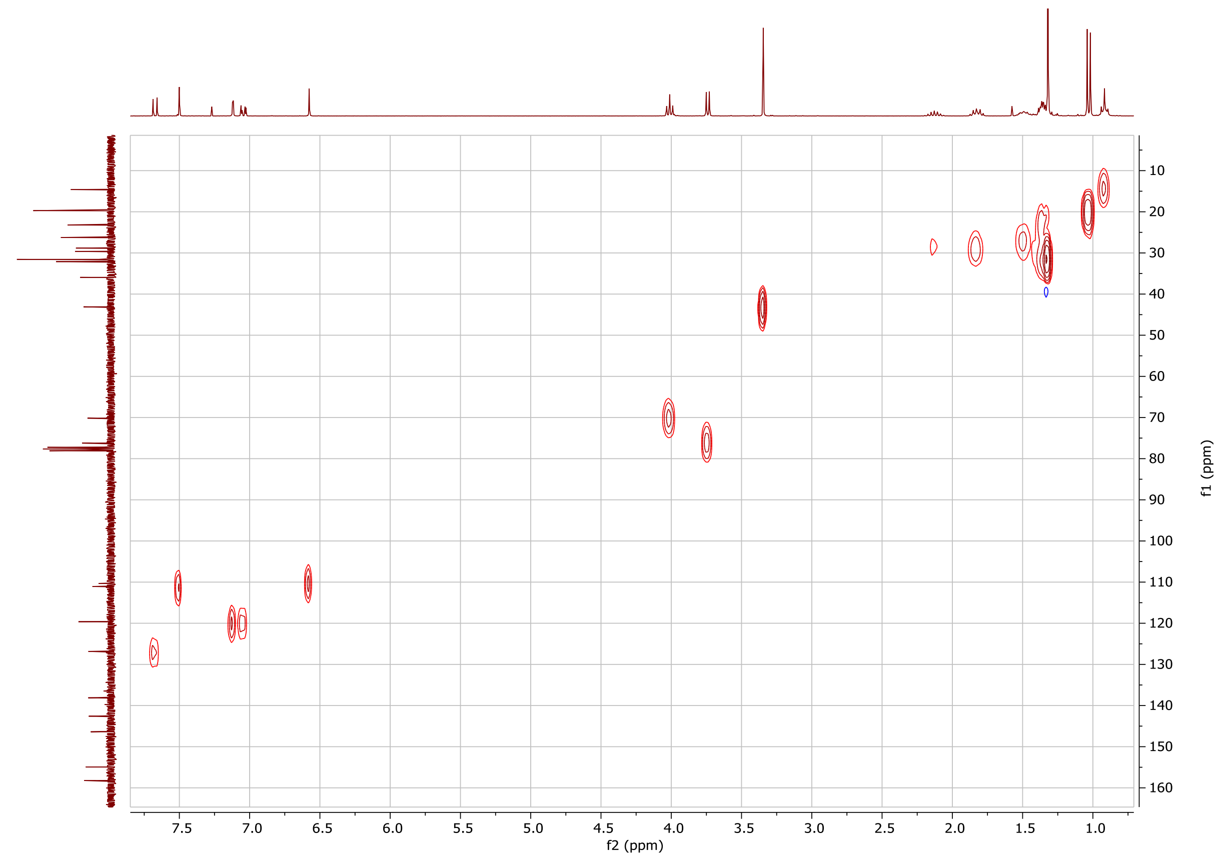

g-HSQC NMR (75 MHz, Chloroform-*d*) spectrum of **4e**.

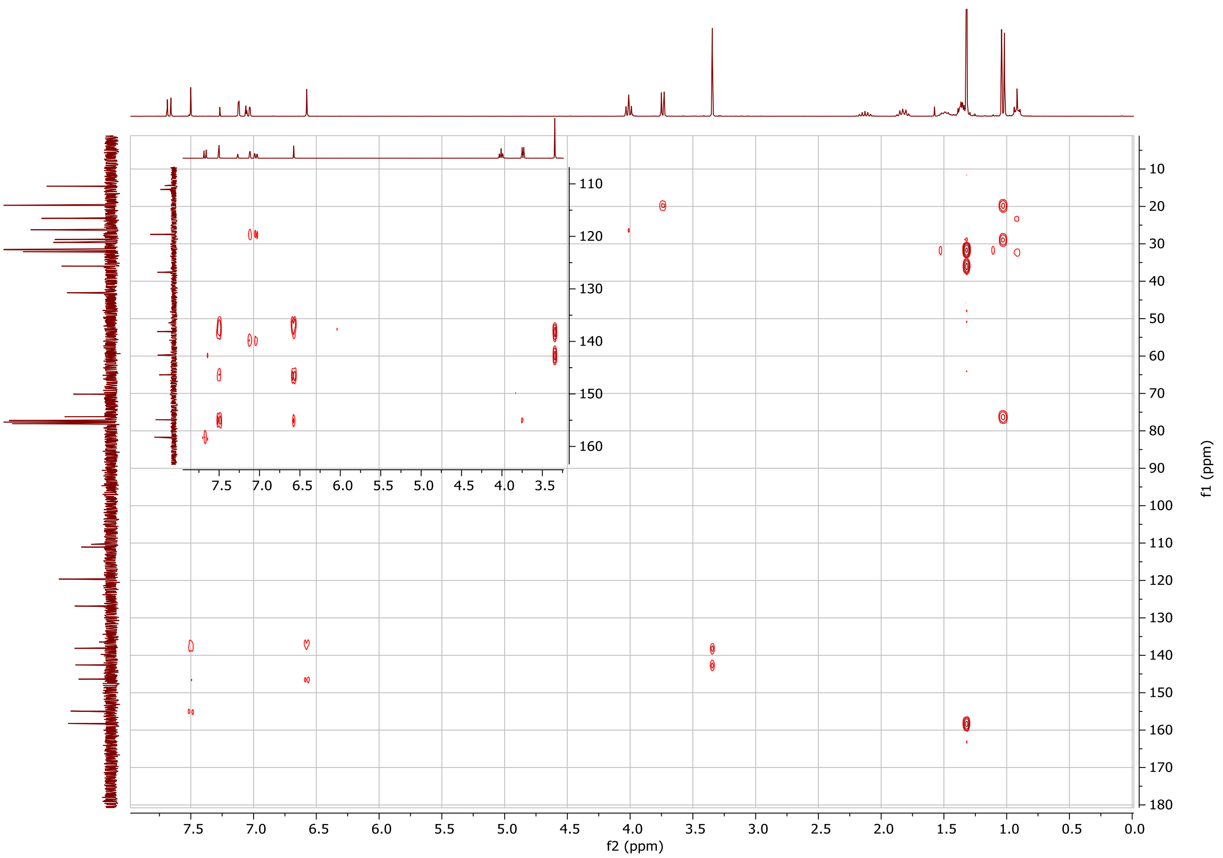

g-HMBC NMR (75 MHz, Chloroform-*d*) spectrum of **4e**.

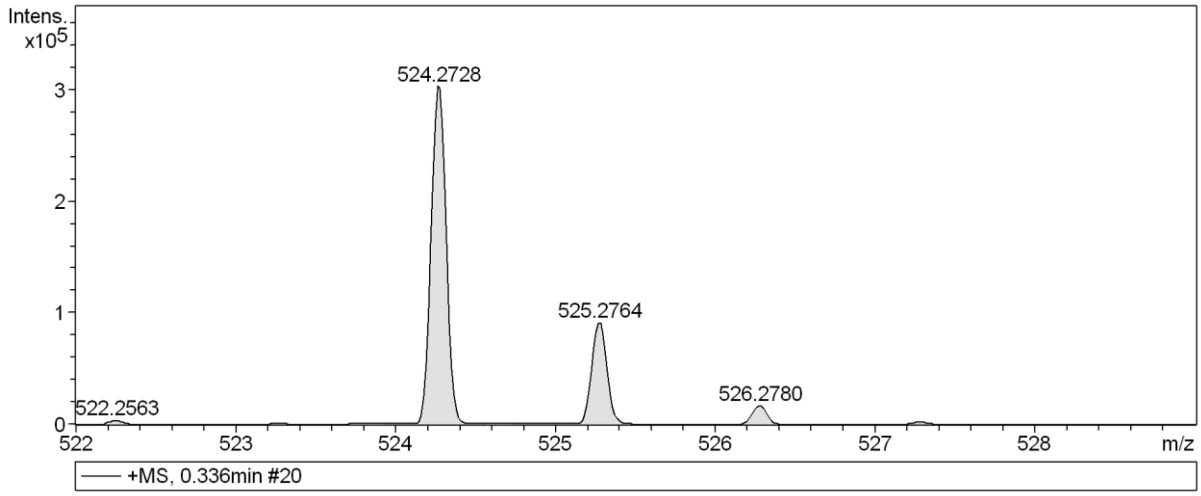

HRMS (ESI+) spectrum of **4e**.

4,5-bis(hexyloxy)-*N*-methyl-2-nitro-*N*-(2-nitrophenyl)aniline (4f)

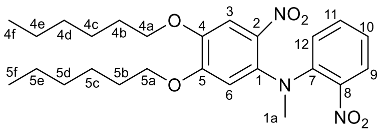

The compound was synthesised from **3f**. Received yellow solid. M.p. = 47.5 – 48.2 °C. Yield = 281 mg (79 %).

^{1}H NMR (300 MHz, Chloroform-*d*) δ: 7.63 (dd, *J* = 8.1, 1.7 Hz, 1H, H_9), 7.51 (s, 1H, H_3), 7.46 (ddd, *J* = 8.7, 7.2, 1.2 Hz, 1H, H_{11}), 7.15 (dd, *J* = 8.7, 1.2 Hz, 1H, H_{12}), 6.97 (td, *J* = 8.1, 7.2, 1.2 Hz, 1H, H_{10}), 6.56 (s, 1H, H_6), 4.04 – 3.91 (m, 4H, $H_{4a,5a}$), 3.32 (s, 3H, H_{1a}), 1.89 – 1.70 (m, 4H, $H_{4b,5b}$), 1.52 – 1.39 (m, 4H, $H_{4c,5c}$), 1.40 – 1.26 (m, 8H, $H_{4d,4e,5d,5e}$), 0.96 – 0.83 (m, 6H, $H_{4f,5f}$).

^{13}C{^{1}H} NMR (75 MHz, Chloroform-*d*) δ: 154.3 (C_5), 146.4 (C_4), 142.1 (C_7), 141.2 (C_8), 137.2 (C_1), 136.5 (C_2), 133.4 (C_{11}), 126.3 (C_9), 121.4 (C_{12}), 121.0 (C_{10}), 110.7 (C_3), 110.2 (C_6), 69.64 (C_{5a}), 69.58 (C_{4a}), 42.5 (C_{1a}), 31.6 (C_{5d}), 31.5 (C_{4d}), 29.0 (C_{5b}), 28.8 (C_{4b}), 25.7 (C_{5c}), 25.6 (C_{4c}), 22.64 (C_{5e}), 22.60 (C_{4e}), 14.1 (C_{5f}), 14.0 (C_{4f}).

HRMS (ESI) *m/z* Calculated for $C_{25}H_{35}N_3O_6Na$ $[M+Na]^+$: 496.2424, found: 496.2415.

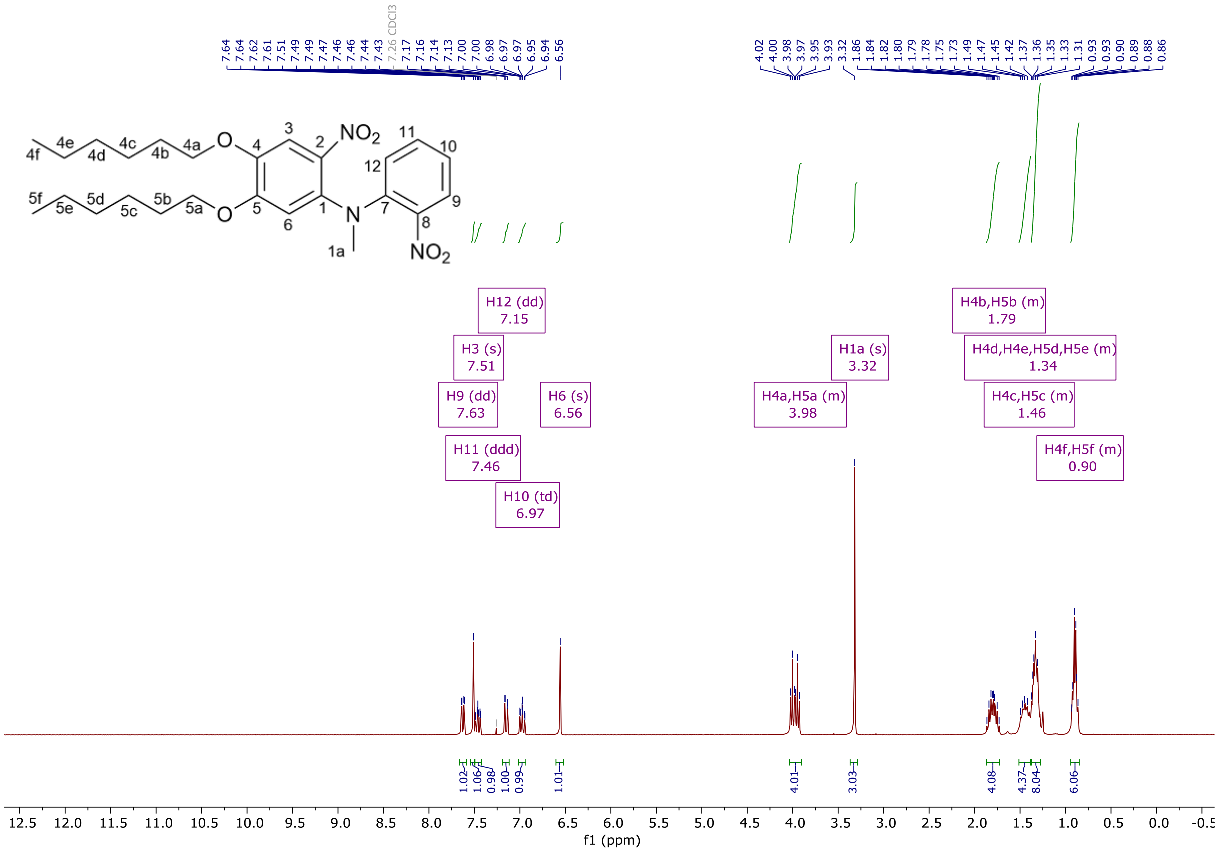

^{1}H NMR (300 MHz, Chloroform-*d*) spectrum of **4f**.

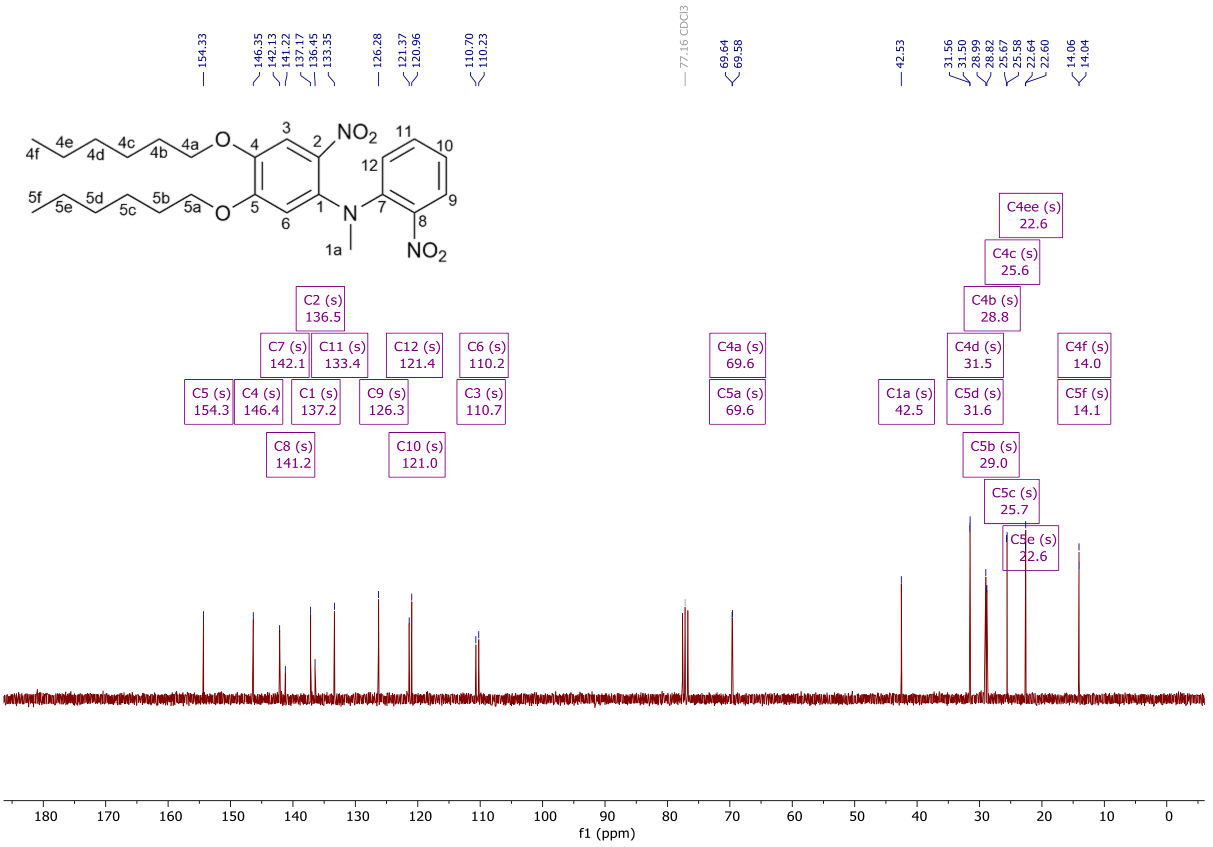

$^{13}C\{^{1}H\}$ NMR (75 MHz, Chloroform-*d*) spectrum of **4f**.

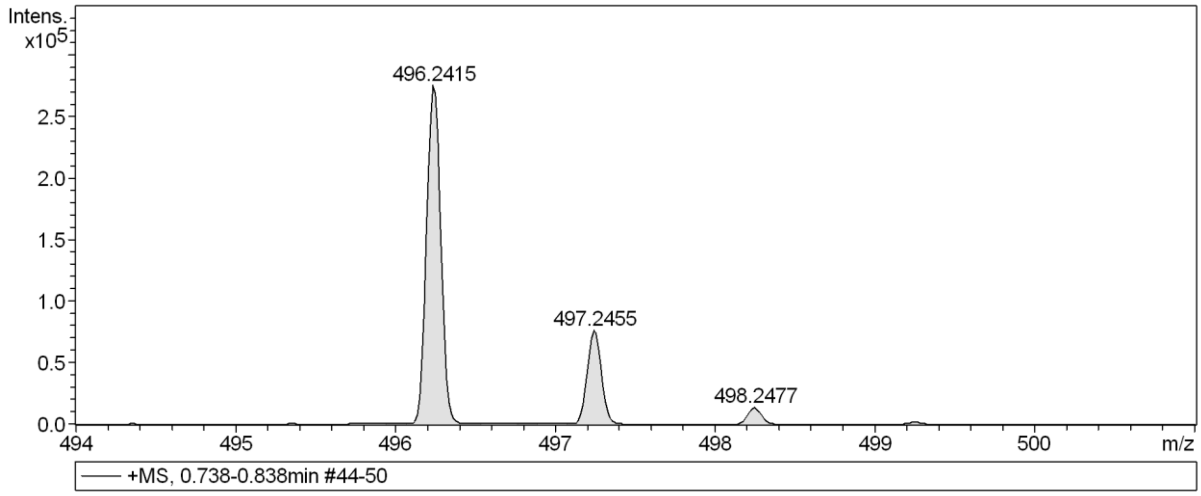

HRMS (ESI+) spectrum of **4f**.

Synthesis of 1-bromo-4,5-dialkoxy-2-nitrobenzene derivatives (5a-5e).

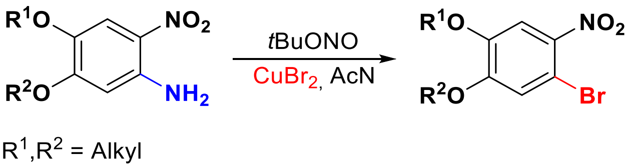

In the round-bottom flask (100 mL of volume), copper(II) bromide (923 mg, 1.5 eq.) and dry acetonitrile (20 mL) were placed. The mixture was heated to 60 °C in an oil bath, and tert-butyl nitrite (723 µL, 625 mg, 2.2 eq.) was added. The solution of 2-nitroaniline (2.75 mmol) in 30 – 50 mL (depending on substrate solubility) of hot, dry acetonitrile was added dropwise keeping the reaction temperature at 60 °C. After 90 minutes, the reaction mixture was cooled to room temperature and poured onto 100 mL of hydrochloric acid (2N), then extracted with DCM (3x 30 mL). Combined organic phases were extracted with brine (50 mL) and dried over anhydrous magnesium sulphate. The solvent was removed under reduced pressure on a rotary evaporator. The crude product was purified on the gradient column chromatography (silica gel, hexane / DCM: 20 – 100 %).

1-bromo-4,5-bis(hexyloxy)-2-nitrobenzene (5a)

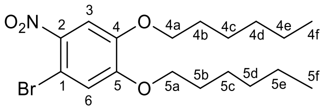

Compound synthesised form 4,5-bis(hexyloxy)-2-nitroaniline. Received yellow solid. M.p. = 38.5 – 42.0 °C. Yield = 938 mg (85 %).

^{1}H NMR (400 MHz, Chloroform-*d*) δ: 7.54 (s, 1H, H_3), 7.08 (s, 1H, H_6), 4.05 (t, *J* = 5.9 Hz, 2H, H_{4a}), 4.02 (t, *J* = 5.9 Hz, 2H, H_{5a}), 1.89 – 1.79 (m, 4H, $H_{4b,5b}$), 1.54 – 1.40 (m, 4H, $H_{4c,5c}$), 1.41 – 1.28 (m, 8H, $H_{4d,4e,5d,5e}$), 0.95 – 0.86 (m, 6H, $H_{4f,5f}$).

^{13}C{^{1}H} NMR (101 MHz, Chloroform-*d*) δ: 153.15 (C_5), 148.13 (C_4), 141.52 (C_2), 117.68 (C_6), 110.58 (C_3), 107.35 (C_1), 69.87 (C_{5a}), 69.77 (C_{4a}), 31.58 (C_{5d}), 31.55 (C_{4d}), 28.93 (C_{5b}), 28.89 (C_{4b}), 25.68 (C_{5c}), 25.64 (C_{4c}), 22.68 (C_{5e}), 22.67 (C_{4e}), 14.11 ($C_{4f,5f}$).

HRMS (ESI) *m/z* Calculated for $C_{18}H_{28}BrNO_4Na$ $[M+Na]^+$: 424.1099, found: 424.1097.

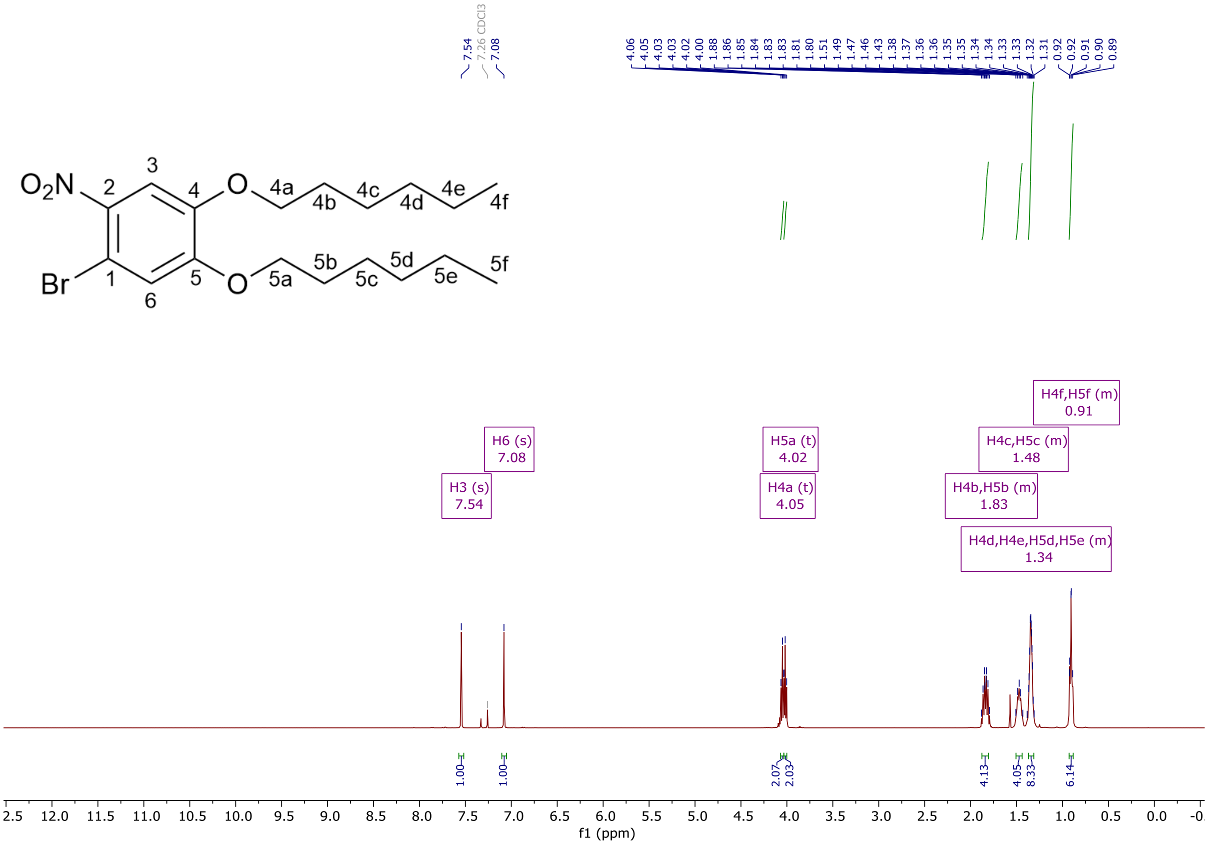

^{1}H NMR (400 MHz, Chloroform-*d*) spectrum of **5a**.

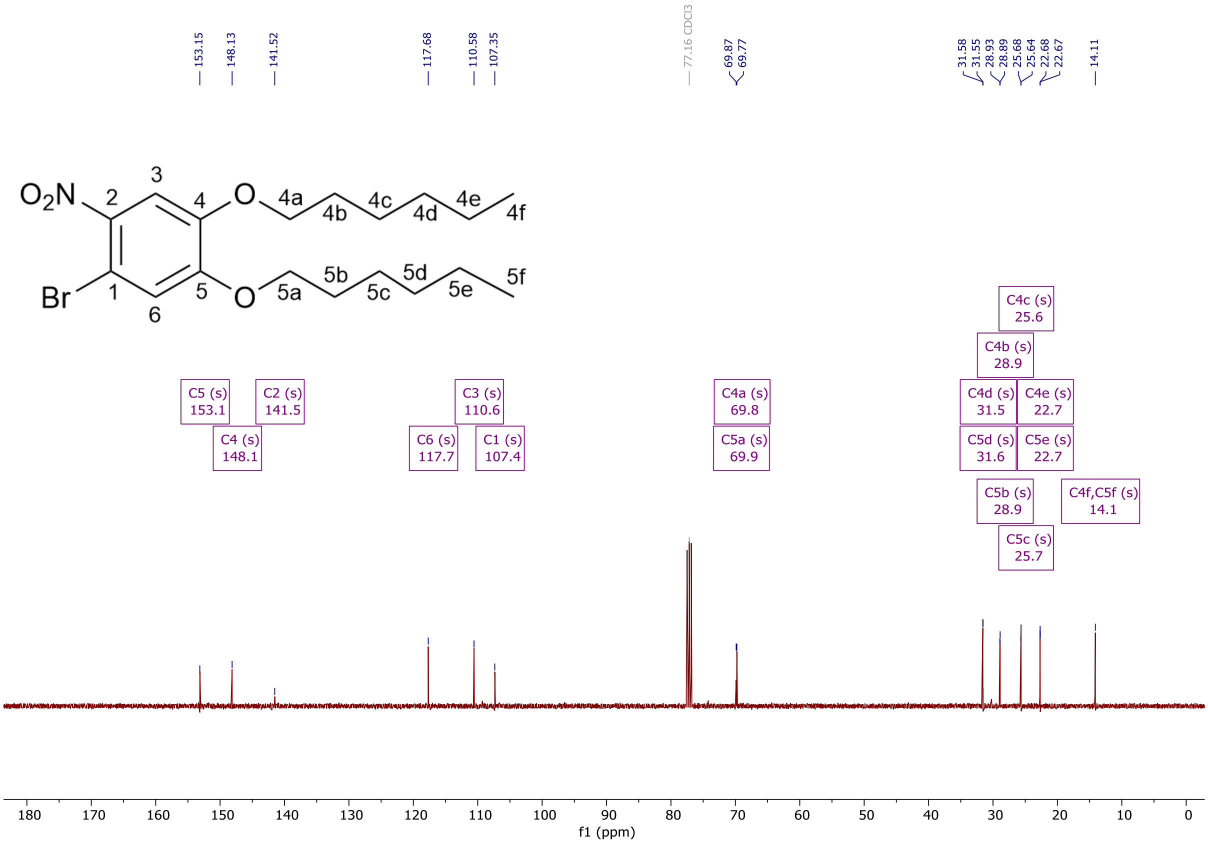

$^{13}C\{^{1}H\}$ NMR (101 MHz, Chloroform-*d*) spectrum of **5a**.

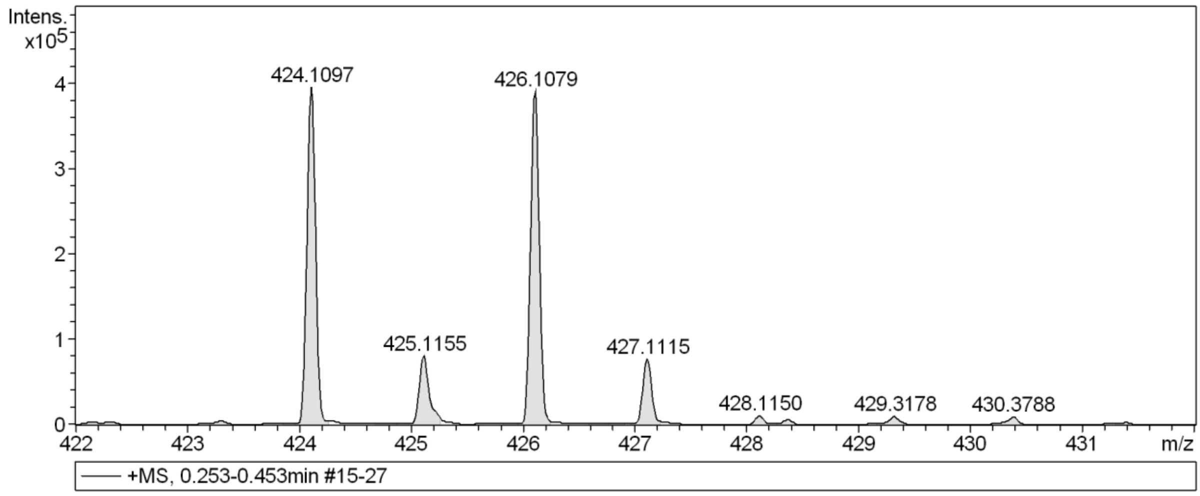

HRMS (ESI+) spectrum of **5a**.

1-bromo-4-(hexyloxy)-5-isobutoxy-2-nitrobenzene (5b)

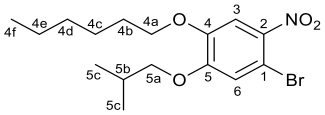

Compound synthesised form 4-(hexyloxy)-5-isobutoxy-2-nitroaniline obtained as described in the literature.[4] Received pale yellow solid. M.p. = 53.0 – 55.0 °C. Yield = 898 mg (87 %).

^{1}H NMR (400 MHz, Chloroform-*d*) δ: 7.54 (s, 1H, H_{3}), 7.07 (s, 1H, H_{6}), 4.02 (t, *J* = 6.5 Hz, 2H, H_{4a}), 3.81 (d, *J* = 6.6 Hz, 2H, H_{5a}), 2.23 – 2.11 (m, 1H, H_{5b}), 1.89 – 1.76 (m, 2H, H_{4b}), 1.54 – 1.41 (m, 2H, H_{4c}), 1.41 – 1.29 (m, 4H, $H_{4d,4e}$), 1.05 (d, *J* = 6.7 Hz, 6H, H_{5c}), 0.95 – 0.85 (m, 3H, H_{4f}).

^{13}C{^{1}H} NMR (101 MHz, Chloroform-*d*) δ: 153.3 (C_{5}), 148.2 (C_{4}), 141.5 (C_{2}), 117.8 (C_{6}), 110.6 (C_{3}), 107.4 (C_{1}), 76.0 (C_{5a}), 69.7 (C_{4a}), 31.6 (C_{4d}), 29.0 (C_{4b}), 28.3 (C_{5b}), 25.7 (C_{4c}), 22.7 (C_{4e}), 19.2 (C_{5c}), 14.1 (C_{4f}).

HRMS (ESI) *m/z* Calculated for $C_{16}H_{24}BrNO_4Na$ $[M+Na]^+$: 396.0786, found: 396.0778.

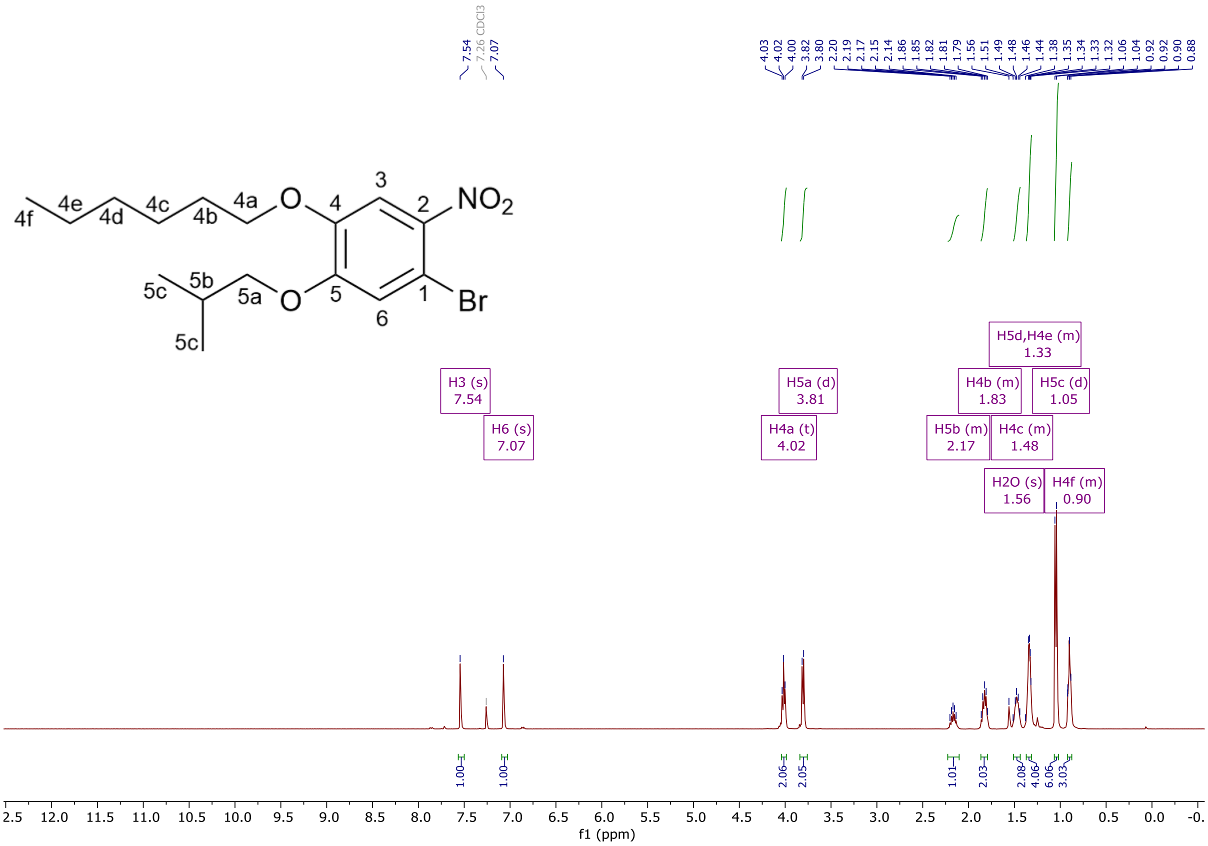

^{1}H NMR (400 MHz, Chloroform-*d*) spectrum of **5b**.

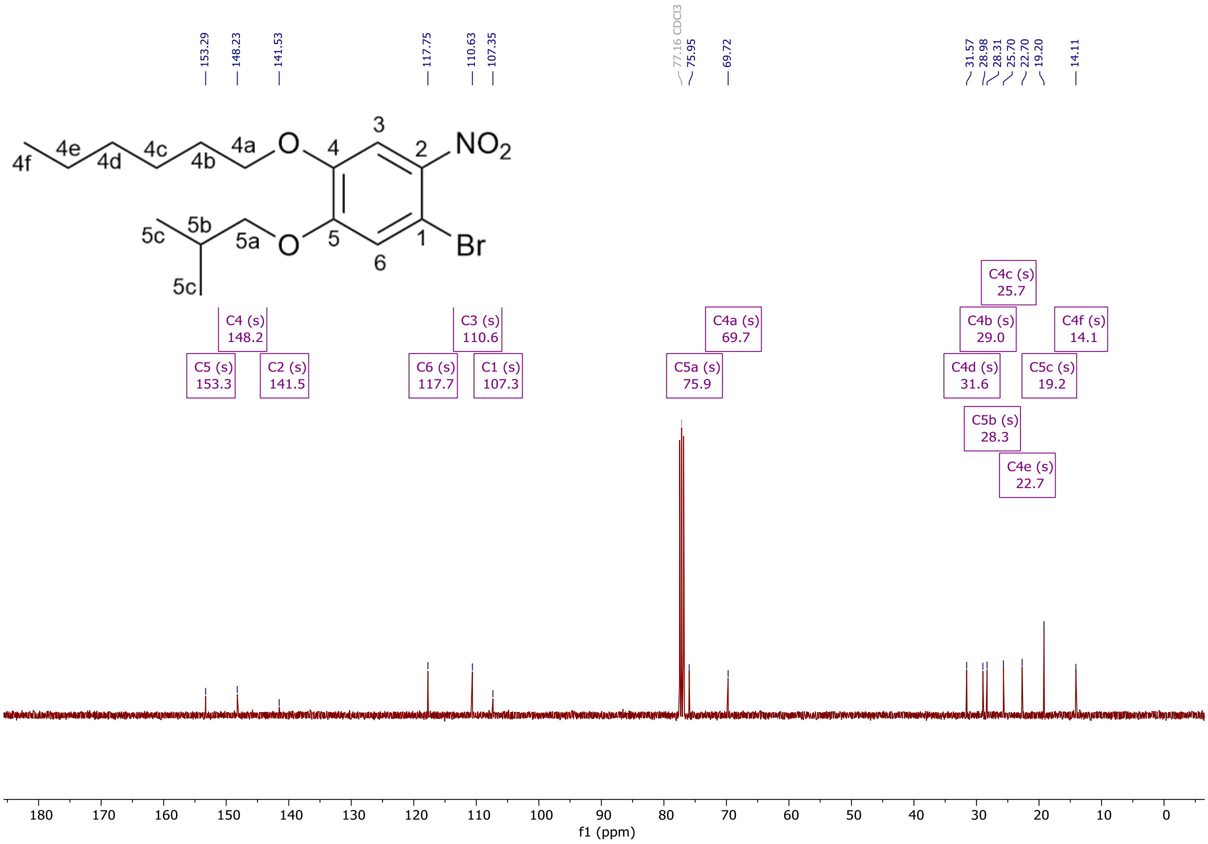

$^{13}C\{^{1}H\}$ NMR (101 MHz, Chloroform-*d*) spectrum of **5b**.

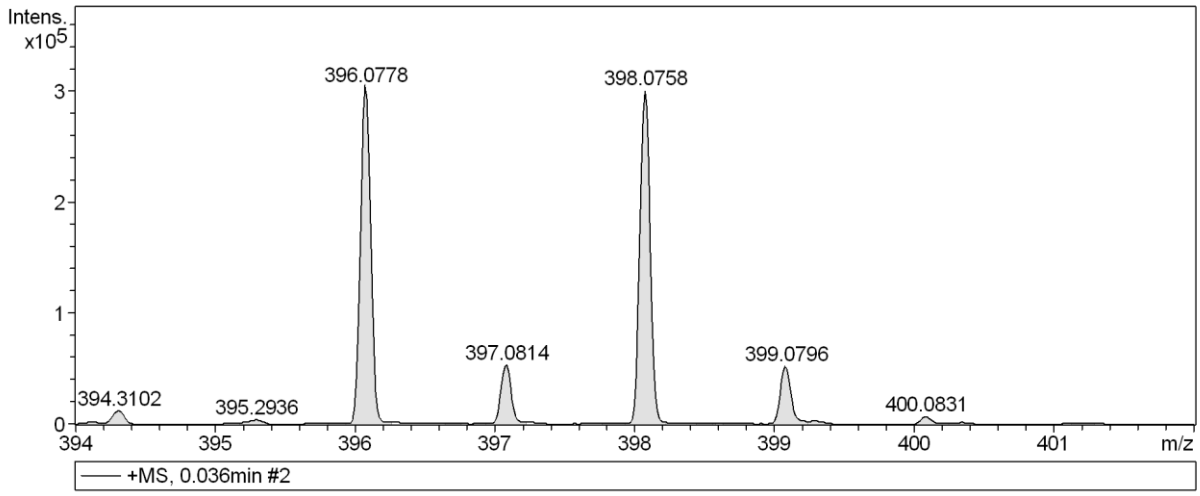

HRMS (ESI+) spectrum of **5b**.

1-bromo-4-ethoxy-5-isobutoxy-2-nitrobenzene (5c)

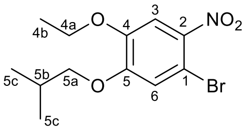

Compound synthesised form 4-ethoxy-5-isobutoxy-2-nitroaniline obtained as described in the literature.[4] Received pale yellow solid. M.p. = 83.6 – 86.0 °C. Yield = 785 mg (90 %).

^{1}H NMR (400 MHz, Chloroform-*d*) δ: 7.55 (s, 1H, H_3), 7.08 (s, 1H, H_6), 4.11 (q, *J* = 7.0 Hz, 2H, H_{4a}), 3.81 (d, *J* = 6.7 Hz, 2H, H_{5a}), 2.26 – 2.10 (m, 1H, H_{5b}), 1.46 (t, *J* = 7.0 Hz, 3H, H_{4b}), 1.05 (d, *J* = 6.7 Hz, 6H, H_{5c}).

^{13}C{^{1}H} NMR (101 MHz, Chloroform-*d*) δ: 153.3 (C_5), 148.0 (C_4), 141.5 (C_2), 117.8 (C_6), 110.8 (C_3), 107.5 (C_1), 76.0 (C_{5a}), 65.4 (C_{4a}), 28.3 (C_{5b}), 19.2 (C_{5c}), 14.6 (C_{4b}).

HRMS (ESI) *m/z* Calculated for $C_{12}H_{16}BrNO_4Na$ $[M+Na]^+$: 340.0160, found: 340.0161.

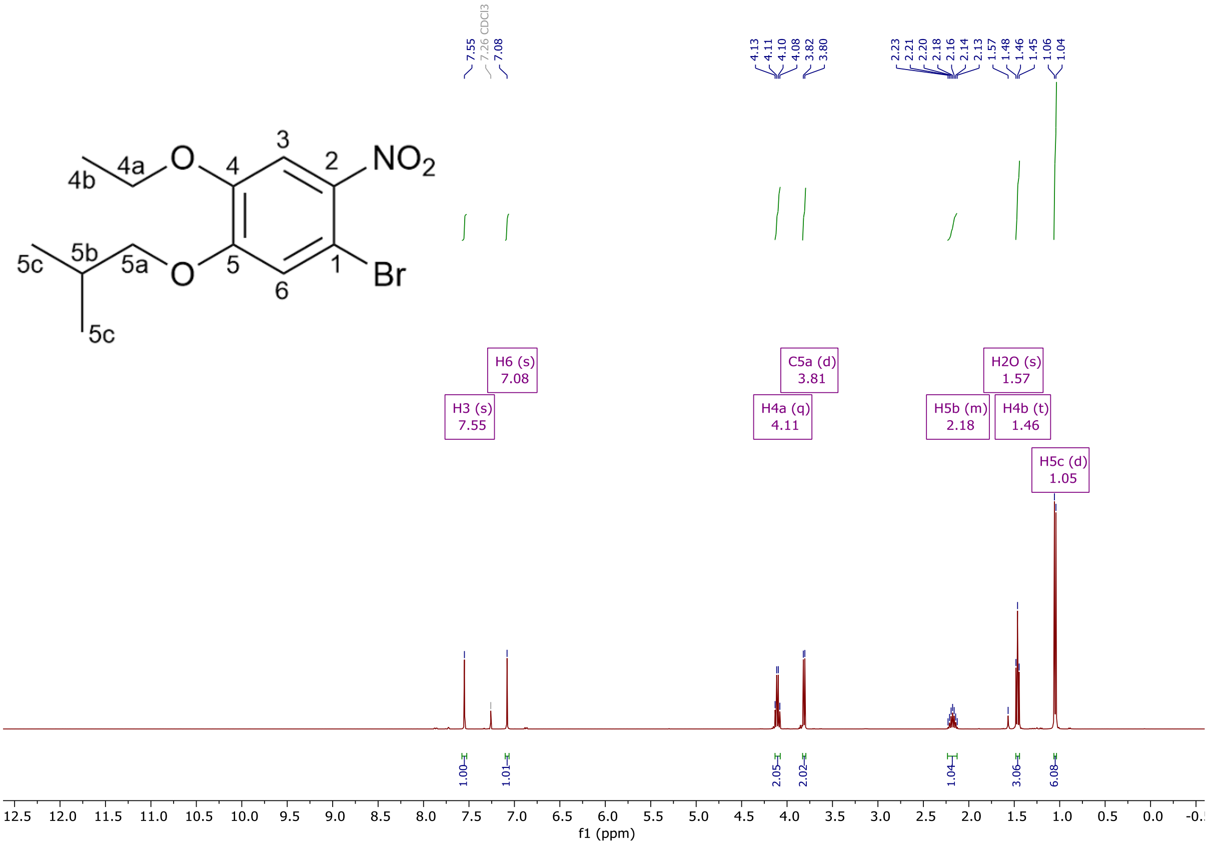

^{1}H NMR (400 MHz, Chloroform-*d*) spectrum of **5c**.

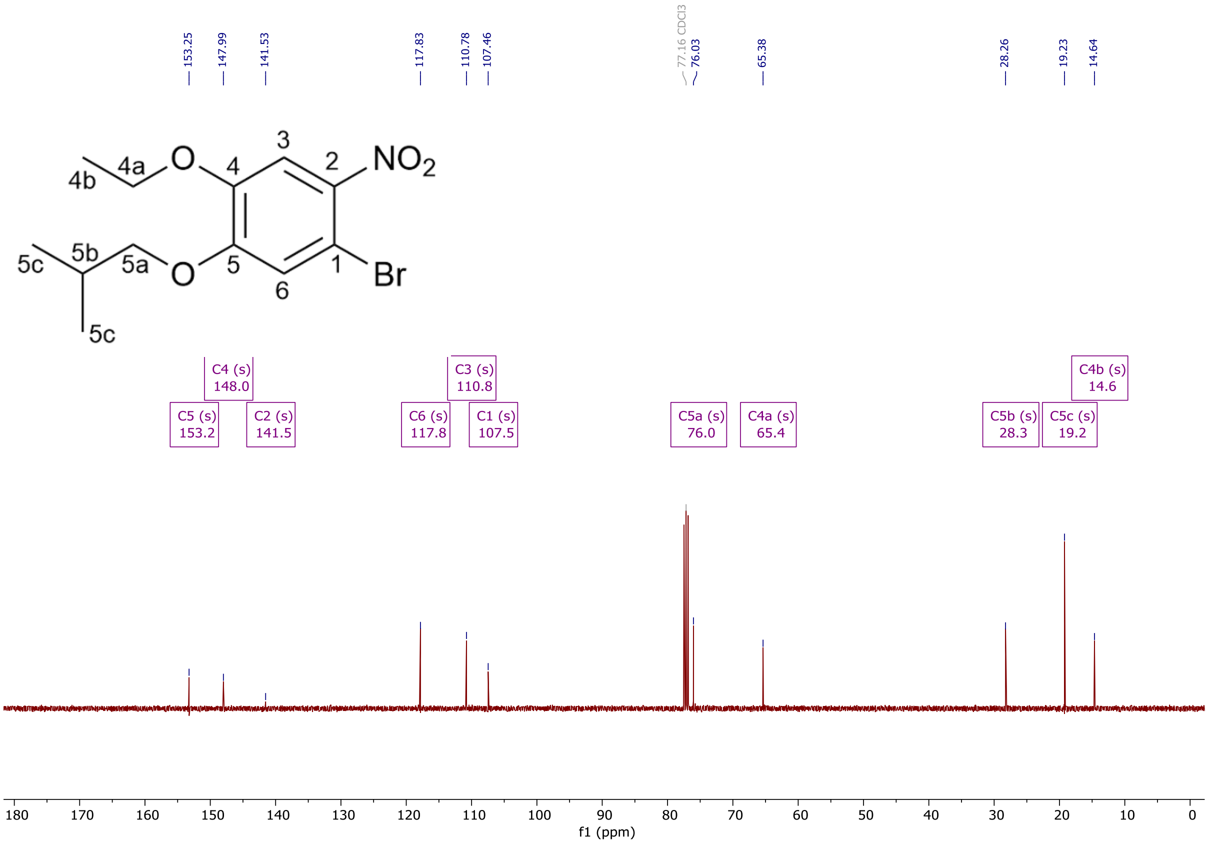

^{13}C{^{1}H} NMR (101 MHz, Chloroform-*d*) spectrum of **5c**.

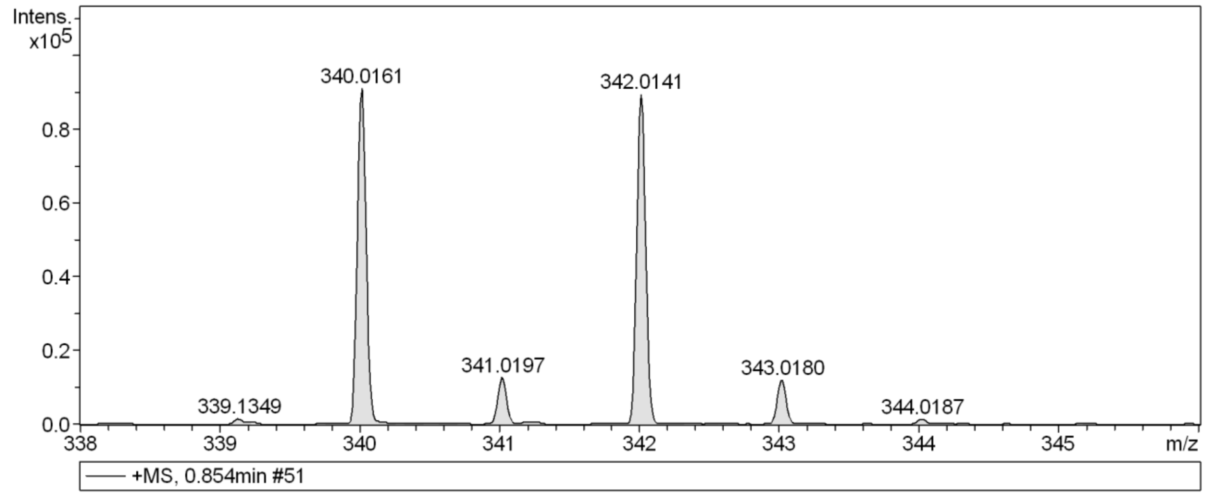

HRMS (ESI+) spectrum of **5c**.

1-bromo-4-ethoxy-5-methoxy-2-nitrobenzene (5d)

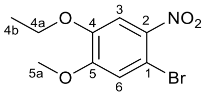

Compound synthesised form 4-ethoxy-5-methoxy-2-nitroaniline obtained as described in the literature.[4] Received pale yellow solid. M.p. = 128.4 – 131.7 °C. Yield = 633 mg (83 %).

^{1}H NMR (300 MHz, Chloroform-*d*) δ: 7.50 (s, 1H, H_3), 7.07 (s, 1H, H_6), 4.10 (q, *J* = 7.0 Hz, 2H, H_{4a}), 3.92 (s, 3H, H_{5a}), 1.46 (t, *J* = 7.0 Hz, 3H, H_{4b}).

$^{13}C\{^{1}H\}$ NMR (75 MHz, Chloroform-*d*) δ: 153.1 (C_5), 147.6 (C_4), 141.8 – 141.5 (m, C_2), 116.6 (C_6), 109.9 (C_3), 107.2 (C_1), 65.2 (C_{4a}), 56.7 (C_{5a}), 14.5 (C_{4b}).

HRMS (ESI) *m/z* Calculated for $C_9H_{10}BrNO_4Na$ $[M+Na]^+$: 297.9691, found: 297.9688.

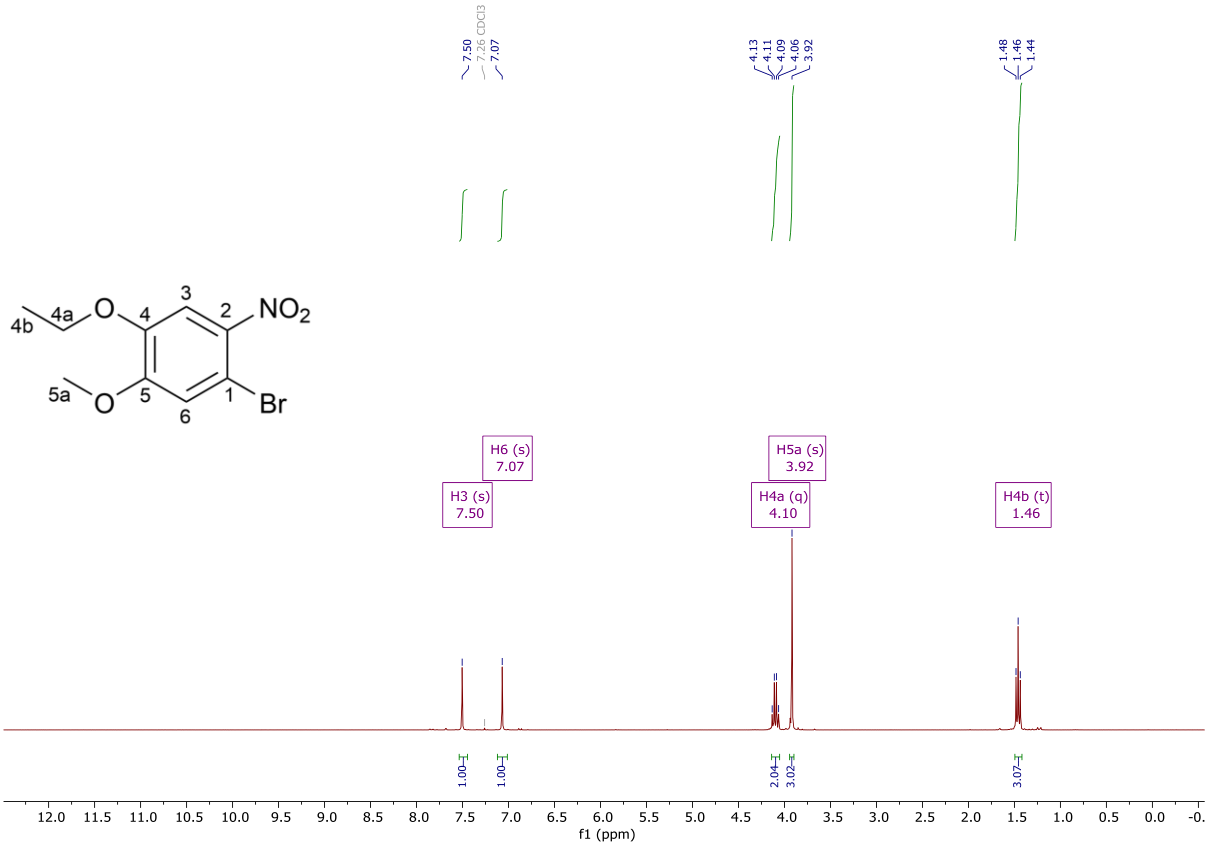

^{1}H NMR (300 MHz, Chloroform-*d*) spectrum of **5d**.

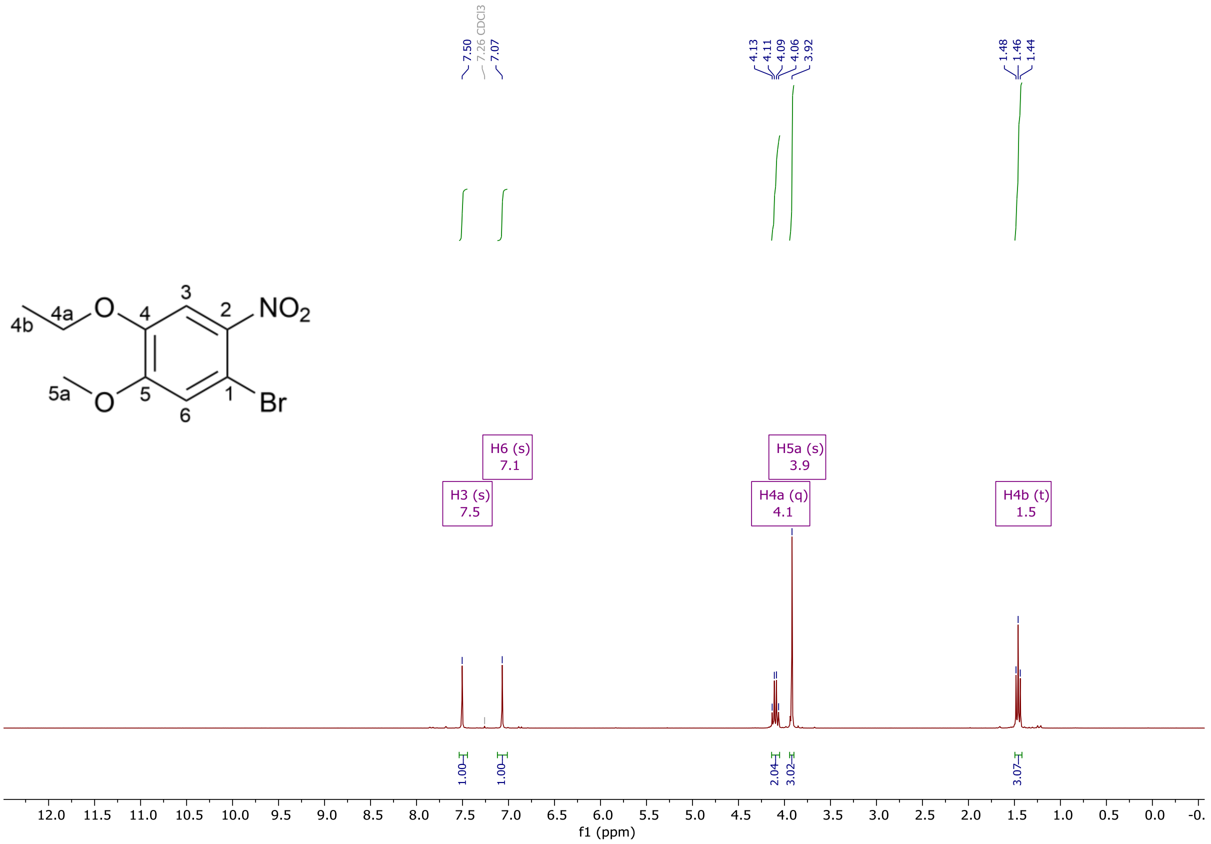

$^{13}C\{^{1}H\}$ NMR (75 MHz, Chloroform-*d*) spectrum of **5d**.

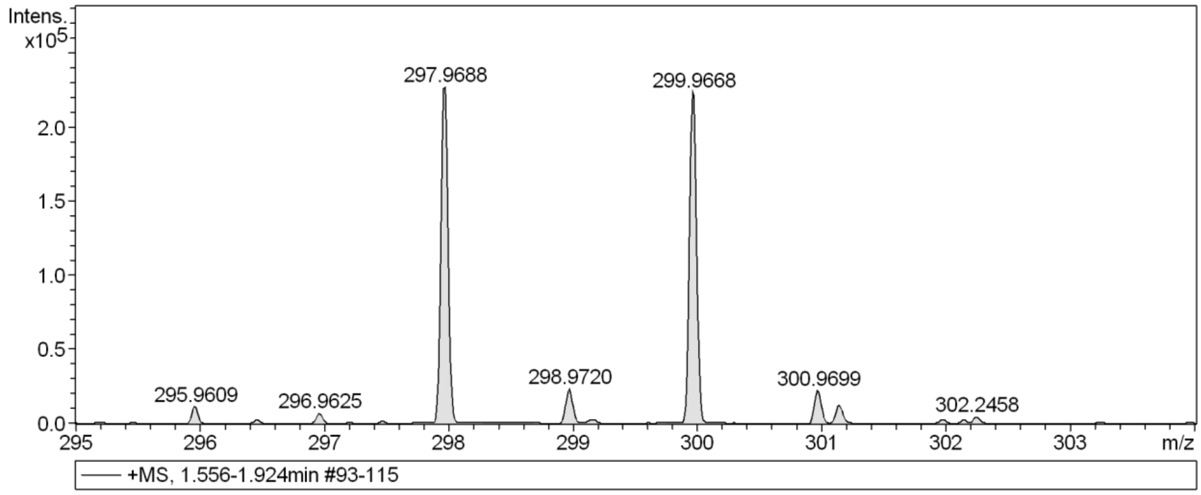

HRMS (ESI+) spectrum of **5d**.

10-(5-bromo-2-methoxy-4-nitrophenoxy)decyl acetate (5e)

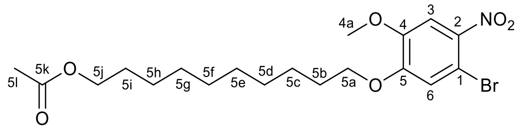

Compound synthesised form 10-(5-amino-2-methoxy-4-nitrophenoxy)decyl acetate (**6b**). Received pale yellow solid, m.p. = 55.7-57.6 °C, Yield = 918 mg (75 %).

^{1}H NMR (400 MHz, Chloroform-*d*) δ 7.53 (s, 1H, H_3), 7.07 (s, 1H, H_6), 4.06 – 4.00 (m, 4H, $H_{5a,5j}$), 3.89 (s, 3H, H_{4a}), 2.01 (s, 3H, H_{5l}), 1.90 – 1.77 (m, 2H, H_{5b}), 1.59 (m, 2H, H_{5i}), 1.50 – 1.37 (m, 2H, H_{5c}), 1.38 – 1.24 (m, 10H, $H_{5d,5e,5f,5g,5h}$).

^{13}C{^{1}H} NMR (101 MHz, Chloroform-*d*) δ: 171.3 (C_{5k}), 152.6 (C_5), 148.5 (C_4), 141.4 (C_2), 117.4 (C_6), 109.2 (C_3), 107.5 (C_1), 69.9 (C_{5a}), 64.7 (C_{5j}), 56.5 (C_{4a}), 29.44 (C_{5b}), 29.43 (C_{5d}), 29.28 (C_{5f}), 29.25 (C_{5e}), 28.8 (C_{5g}), 28.6 (C_{5i}), 25.9 (C_{5c}), 25.8 (C_{5h}), 21.1 (C_{5l}).

HRMS (ESI) *m/z* Calculated for $C_{19}H_{28}BrNO_6Na$ $[M+Na]^+$: 468.0998, found: 468.0988.

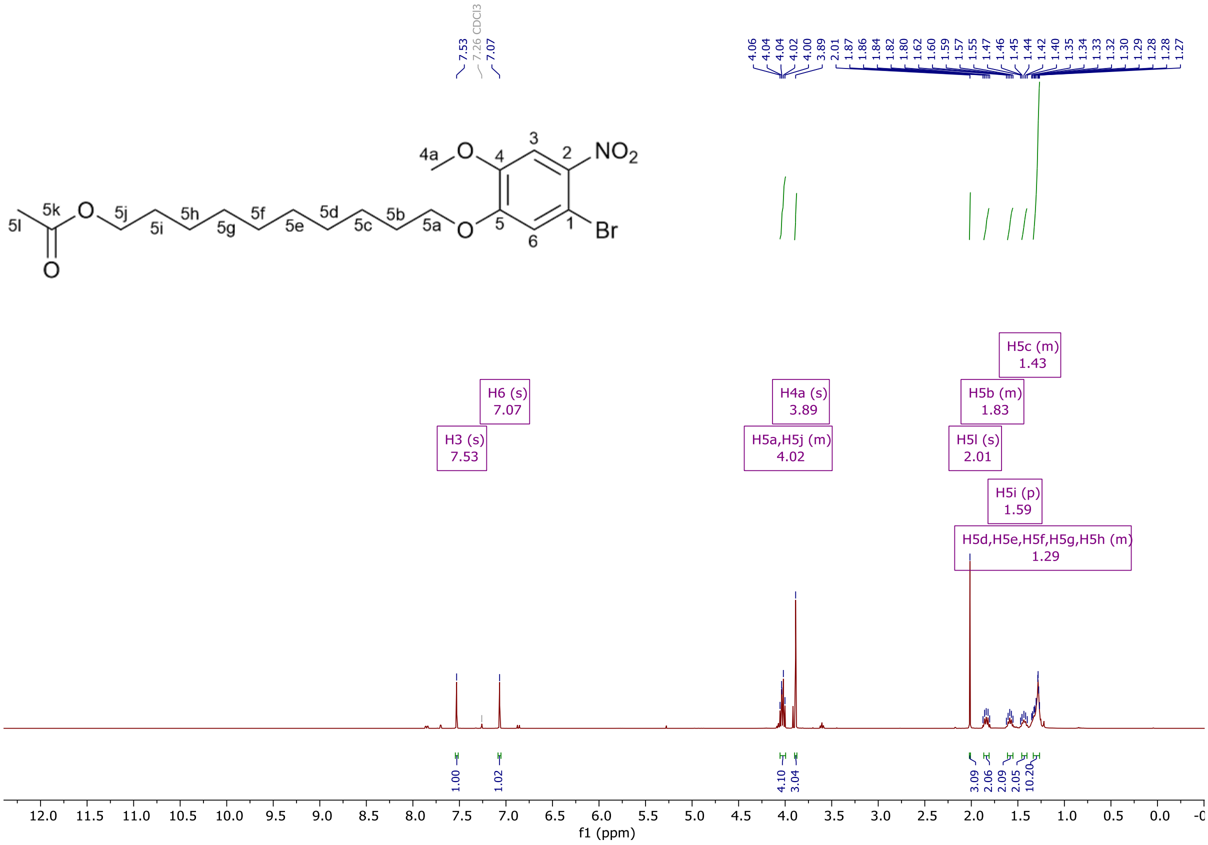

^{1}H NMR (400 MHz, Chloroform-*d*) spectrum of **5e**.

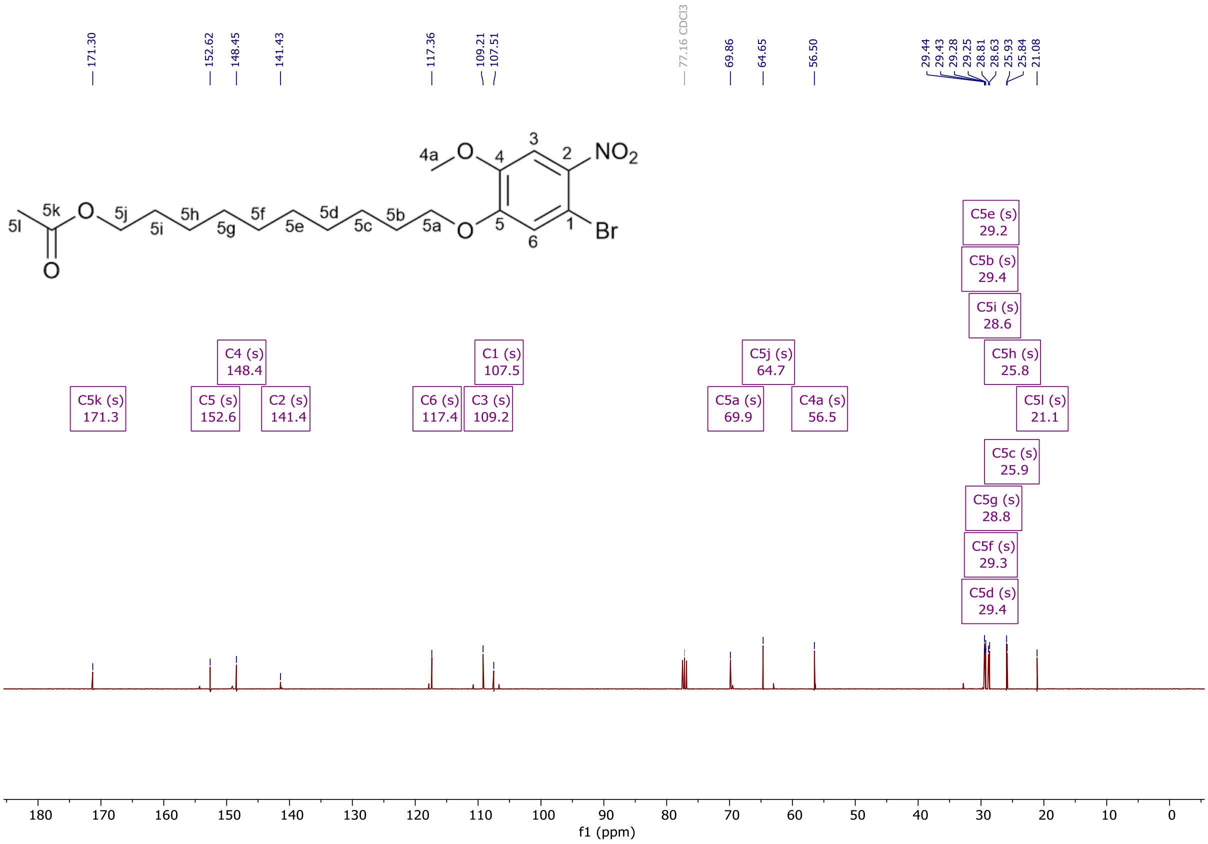

$^{13}C\{^{1}H\}$ NMR (101 MHz, Chloroform-*d*) spectrum of **5e**.

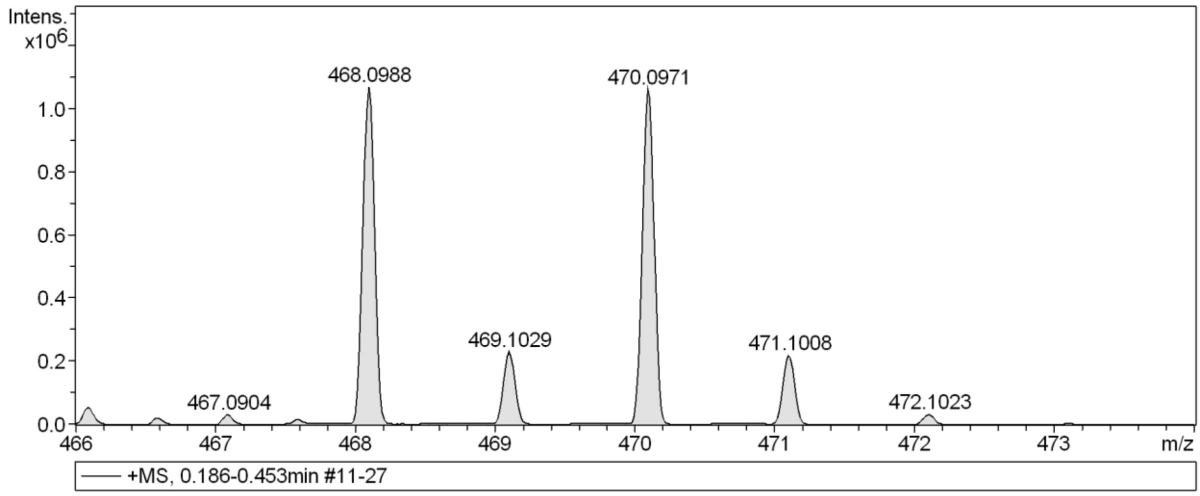

HRMS (ESI+) spectrum of **5e**.

Synthesis of 10-(5-amino-2-methoxy-4-nitrophenoxy)decan-1-ol (6a).

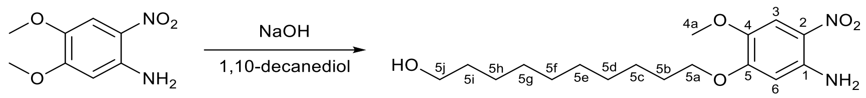

In the round-bottom flask (250 mL of volume) 4,5-dimethoxy-2-nitroaniline (1.00 g, 5.05 mmol), 1,10-decanediol (89.00 g, 100 eq.) and freshly ground sodium hydroxide (2.02 g. 10 eq.) were placed. The mixture was then heated to 90 °C in an oil bath for 24 h. To the hot mixture, acetic acid (3.5 mL, 12 eq.) was added, and the hot mixture was poured into hot chloroform (750 mL). After cooling to room temperature crystalized decanediol was removed by filtration and the filtrate was concentrated to around 100 mL. After cooling to room temperature, the next portion of decanediol was removed by filtration and filtrate was concentrated one more time to the volume of 40 mL. After removing the last portion of decanediol the filtrate was loaded to a short flash-column and purified with gradient elution (silica gel, DCM / MeOH: 1 – 2%).

The recovered 1,10-decanediol can be reused in the same reaction at least three times after drying on air, and then under vacuum. The column chromatography does not allow the removal of residual decanediol which is present in the product at ~1:1 ratio. The reaction yield and exact amount of decanediol can be determined by ^{1}H NMR. An analytical sample of **6a** was obtained by removing decanediol by sublimation under vacuum at 110 °C. The product (**6b**) can be easily purified after the reaction on the substrate (**6a**) containing decanediol, therefore using it as the mixture for the next step is beneficial.

Received orange solid, m.p. = 102.5 – 103.5 °C, yield = 1.39 g (81 %, NMR).

^{1}H NMR (600 MHz, Chloroform-*d*) δ: 7.50 (s, 1H, H_3), 6.21 (s, 2H, H_{NH2}), 6.15 (s, 1H, H_6), 3.99 (t, *J* = 6.8 Hz, 2H, H_{5a}), 3.82 (s, 3H, H_{4a}), 3.63 (t, *J* = 6.6 Hz, 2H, H_{5j}), 3.49 – 3.44 (m, 1H, H_{OH}), 1.87 – 1.82 (m, 2H, H_{5b}), 1.60 – 1.50 (m, 2H, H_{5i}), 1.49 – 1.38 (m, 2H, H_{5c}), 1.38 – 1.25 (m, 10H, $H_{5d,5e,5f,5g,5h}$).

^{13}C{^{1}H} NMR (151 MHz, Chloroform-*d*) δ: 156.7 (C_5), 142.9 (C_4), 141.9 (C_1), 124.4 (C_2), 106.8 (C_3), 99.7 (C_6), 69.5 (C_{5a}), 63.1 (C_{5j}), 56.4 (C_{4a}), 32.8 (C_{5i}), 29.6 (C_{5b}), 29.47 (C_{5d}), 29.45 (C_{5e}), 29.3 (C_{5f}), 28.8 (C_{5g}), 25.9 (C_{5h}), 25.8 (C_{5c}).

HRMS (ESI) *m/z* Calculated for $C_{17}H_{28}N_2O_5Na$ $[M+Na]^+$: 363.1896, found: 363.1885.

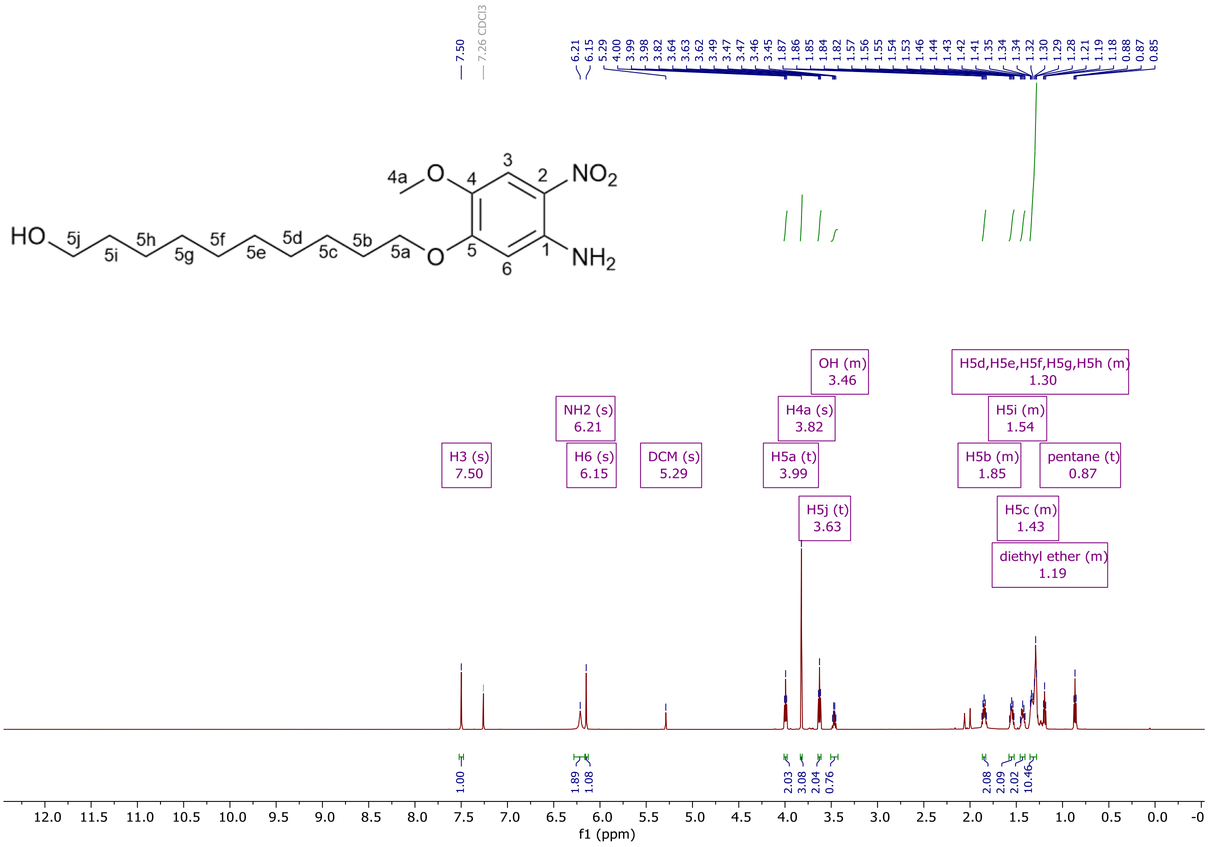

^{1}H NMR (600 MHz, Chloroform-*d*) spectrum of **6a**.

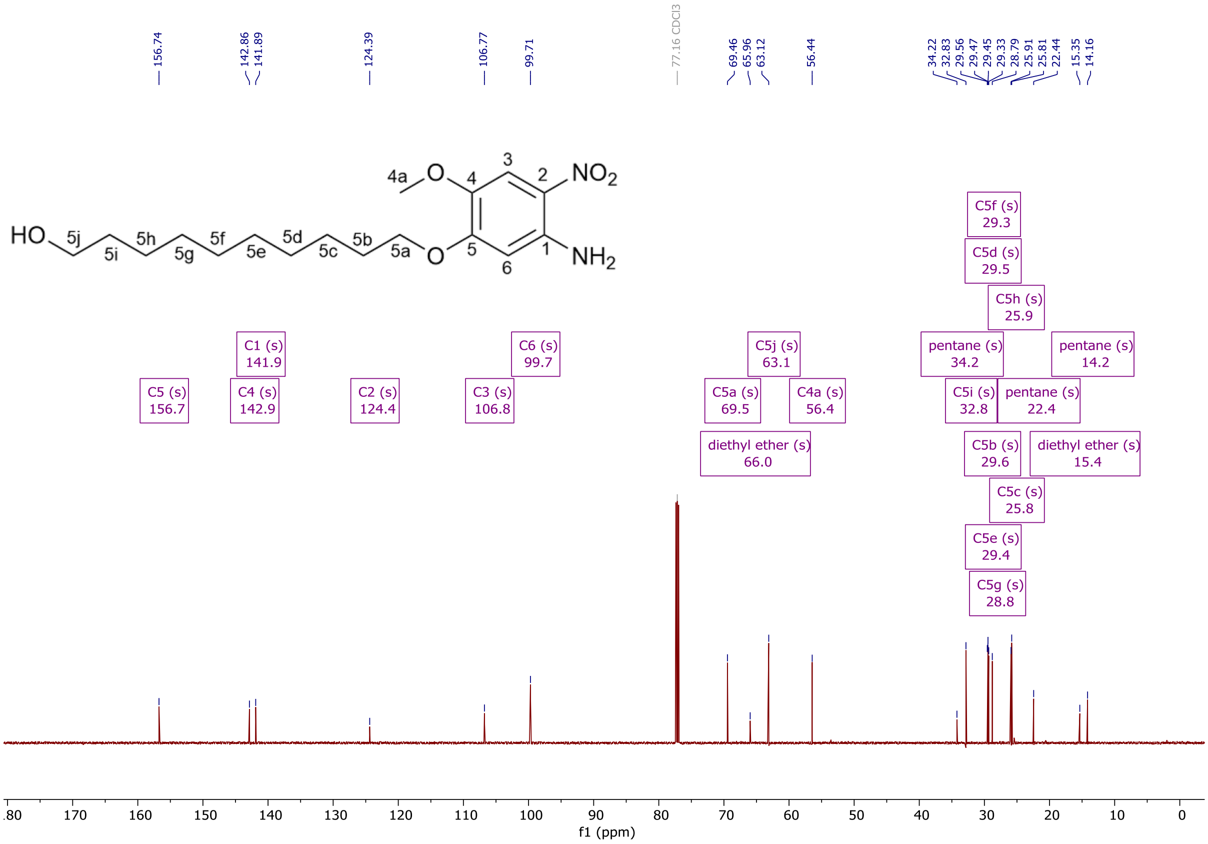

^{13}C{^{1}H} NMR (151 MHz, Chloroform-*d*) spectrum of **6a**.

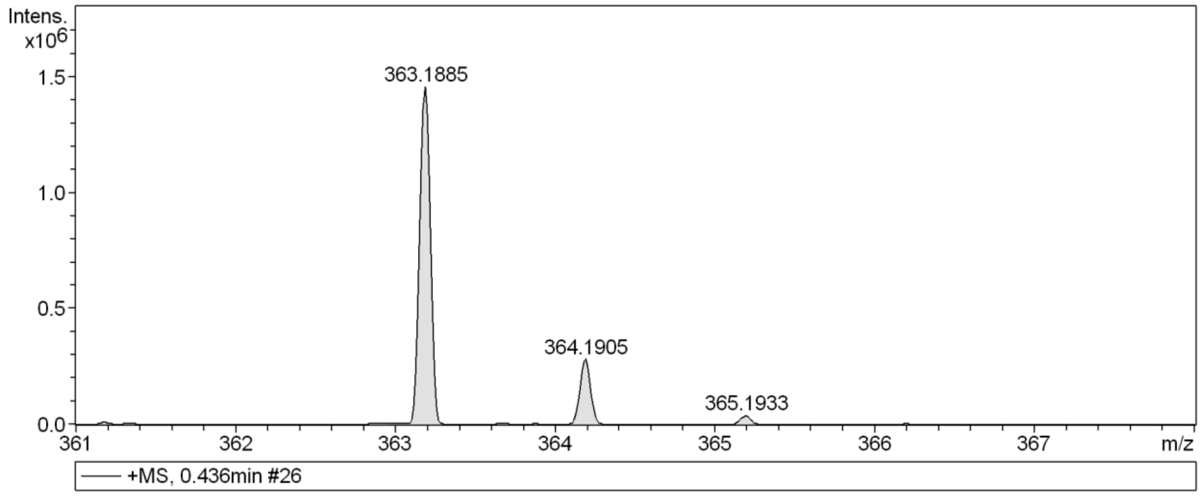

HRMS (ESI+) spectrum of **6a**.

Synthesis of 10-(5-amino-2-methoxy-4-nitrophenoxy)decyl acetate (6b).

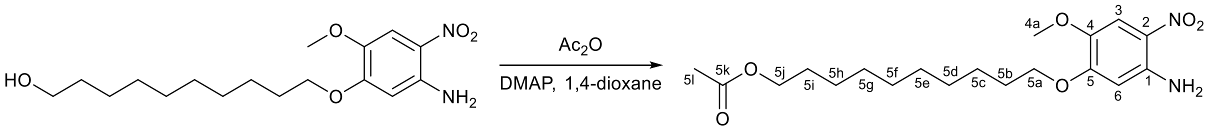

In the round-bottom flask (250 mL of volume) the mixture containing **6a** (865 mg, 2.54 mmol) and decanediol (483 mg, 1.09 eq. – determined by NMR) was placed. The anhydrous 1,4-dioxane (100 mL) was added to dissolve the mixture, then DMAP (3 mg, 0.01 eq.) and acetic anhydride (878 µL, 3.66 eq. – 1.15 eq. regarding each hydroxyl group) were added and the mixture was sired under argon for 2 h in 80 °C in an oil bath. Then the reaction mixture was cooled to room temperature, poured into water (200 mL) and extracted with DCM (3x 50 mL). Combined organic phases were dried over anhydrous magnesium sulphate, and the solvent was removed on a rotary evaporator. The crude product was purified by column chromatography (silica gel, DCM).

Received orange solid, m.p. = 101.0-102.7 °C, yield = 866 mg (89 %).

^{1}H NMR (400 MHz, Chloroform-*d*) δ: 7.49 (s, 1H, H_3), 6.24 (s, 2H, H_{NH2}), 6.16 (s, 1H, H_6), 4.03 (t, *J* = 6.8 Hz, 2H, H_{5j}), 3.98 (t, *J* = 6.8 Hz, 2H, H_{5a}), 3.81 (s, 3H, H_{4a}), 2.03 (s, 3H, H_{5l}), 1.90 – 1.77 (m, 2H, H_{5b}), 1.64 – 1.55 (m, 2H, H_{5i}), 1.49 – 1.36 (m, 2H, H_{5c}), 1.38 – 1.23 (m, 10H, $H_{5d,5e,5f,5g,5h}$).

^{13}C{^{1}H} NMR (101 MHz, Chloroform-*d*) δ: 171.4 (C_{5k}), 156.7 (C_5), 142.9 (C_4), 141.8 (C_1), 124.3 (C_2), 106.6 (C_3), 99.7 (C_6), 69.4 (C_{5a}), 64.7 (C_{5j}), 56.4 (C_{4a}), 29.44 (C_{5b}), 29.43 (C_{5d}), 29.3 (C_{5f}), 29.2 (C_{5e}), 28.8 (C_{5g}), 28.6 (C_{5i}), 25.9 (C_{5c}), 25.9 (C_{5h}), 21.1 (C_{5l}).

HRMS (ESI) *m/z* Calculated for $C_{19}H_{30}N_2O_6Na$ $[M+Na]^+$: 405.2002, found: 405.1995.

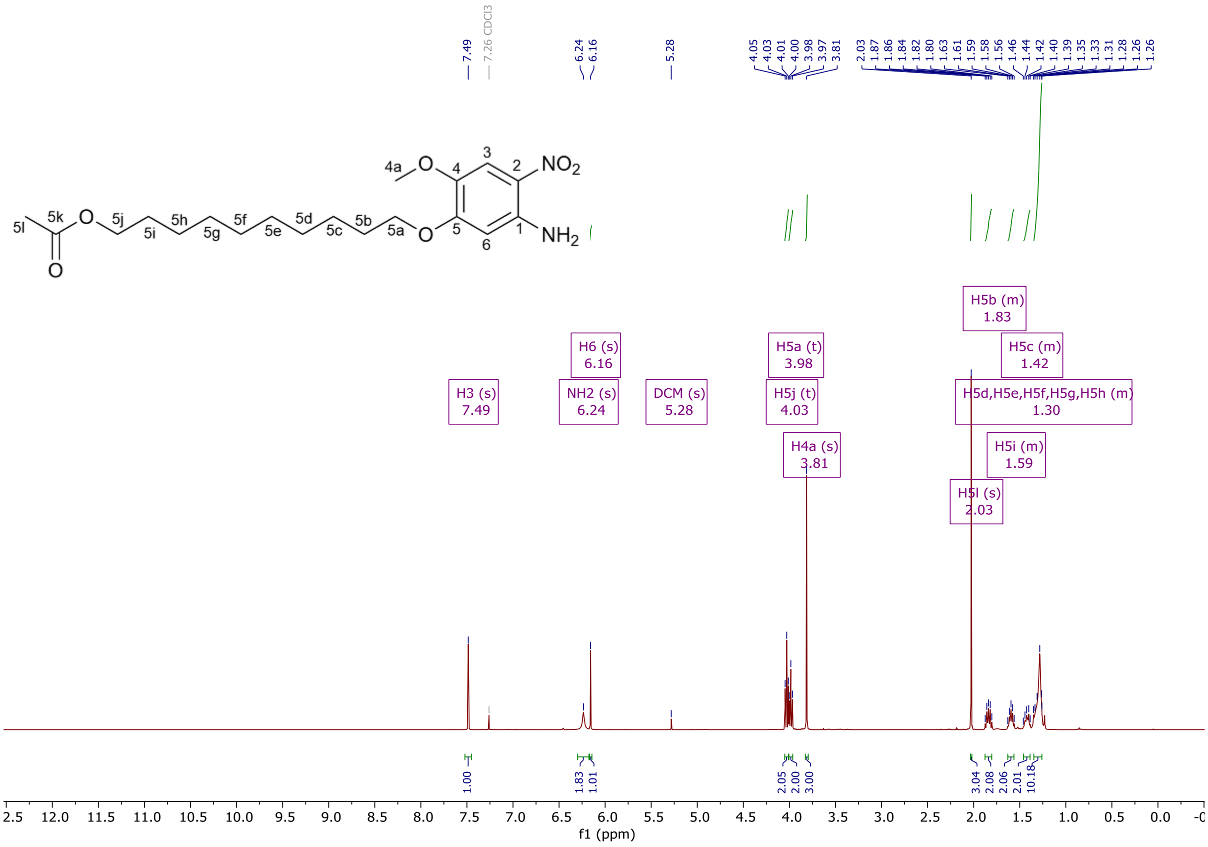

^{1}H NMR (400 MHz, Chloroform-*d*) spectrum of **6b**.

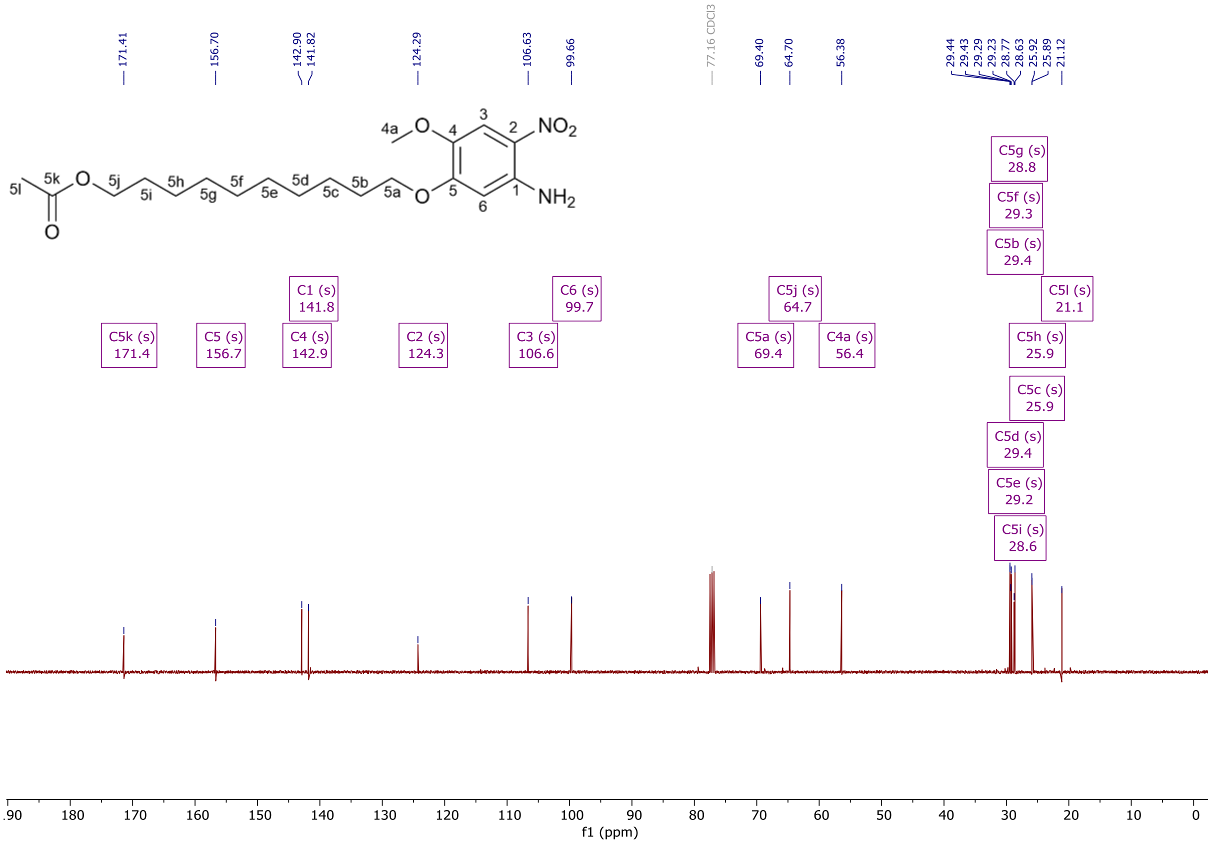

$^{13}C\{^{1}H\}$ NMR (101 MHz, Chloroform-*d*) spectrum of **6b**.

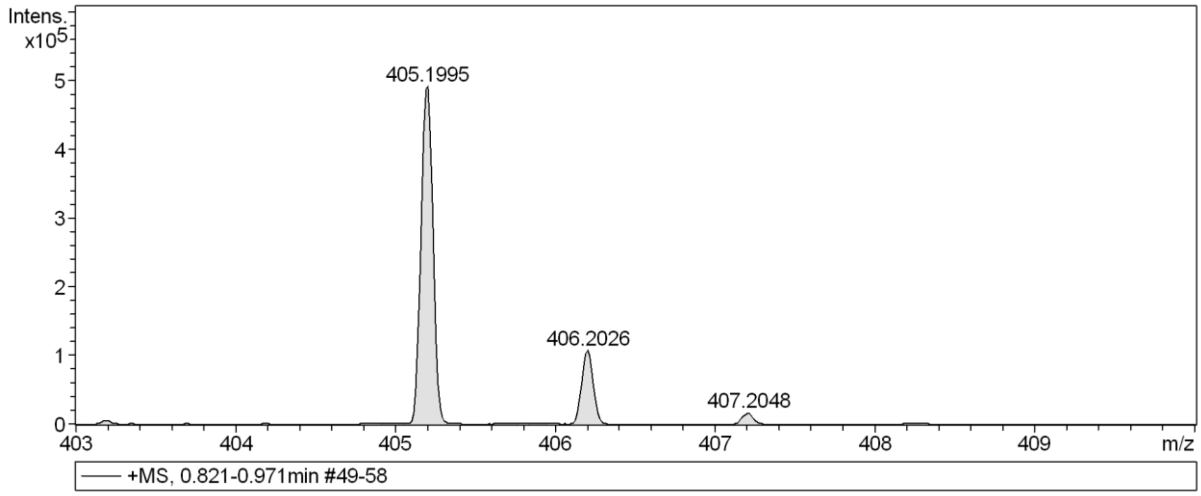

HRMS (ESI+) spectrum of **6b**.

Synthesis of 4-(dodecyloxy)-5-(isopentyloxy)-2-nitroaniline (6c).

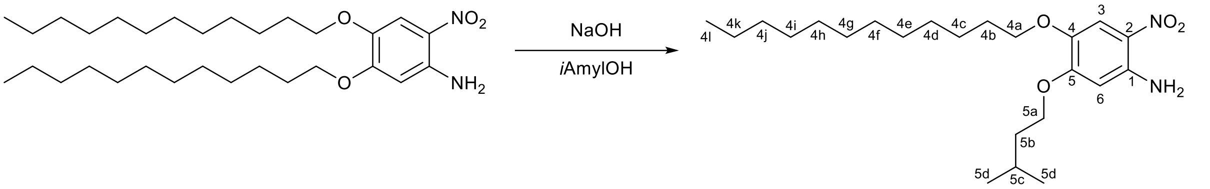

In the round-bottom flask (50 mL of volume) 4,5-bis(dodecyloxy)-2-nitroaniline (803 mg, 1.58 mmol), 3-methylbutan-1-ol (35 mL, 28.35 g, 200 eq.) and freshly ground sodium hydroxide (632 mg. 10 eq.) were placed. The mixture was then heated to 90 °C in an oil bath for 24 h. To the hot mixture, acetic acid (1 mL, 11 eq.) was added, and the solvent was removed on a rotary evaporator. The crude product was purified by column chromatography (silica gel, DCM) with an additional short pad of celite 545 on top of the column.

Received orange solid, m.p. = 73.1 – 76.5 °C. Yield = 476 mg (74 %).

^{1}H NMR (400 MHz, Chloroform-*d*) δ: 7.45 (s, 1H, H_3), 6.40 (s, 2H, H_{NH2}), 6.20 (s, 1H, H_6), 3.96 (t, *J* = 6.6 Hz, 2H, H_{5a}), 3.87 (t, *J* = 6.6 Hz, 2H, H_{4a}), 1.85 – 1.63 (m, 5H, $H_{4b,5b,5c}$), 1.47 – 1.33 (m, 2H, H_{4c}), 1.35 – 1.17 (m, 16H, $H_{4d,4e,4f,4g,4h,4i,4j,4k}$), 0.92 (d, *J* = 6.6 Hz, 6H, H_{5d}), 0.87 – 0.79 (m, 3H, H_{4l}).

^{13}C{^{1}H} NMR (101 MHz, Chloroform-*d*) δ: 157.4 (C_5), 143.4 (C_1), 141.1 (C_4), 124.0 (C_2), 108.2 (C_3), 99.6 (C_6), 69.7 (C_{4a}), 67.7 (C_{5a}), 37.4 (C_{5b}), 31.9 (C_{4j}), 29.7 (C_{4b}), 29.63 (C_{4d}), 29.59 (C_{4g}), 29.57 (C_{4h}), 29.4 (C_{4e}), 29.3 (C_{4f}), 29.1 (C_{4i}), 26.0 (C_{5c}), 25.2 (C_{4c}), 22.7 (C_{4k}), 22.5 (C_{5d}), 14.1 (C_{4l}).

HRMS (ESI) *m/z* Calculated for $C_{23}H_{40}N_2O_4Na$ $[M+Na]^+$: 431.2886, found: 431.2879.

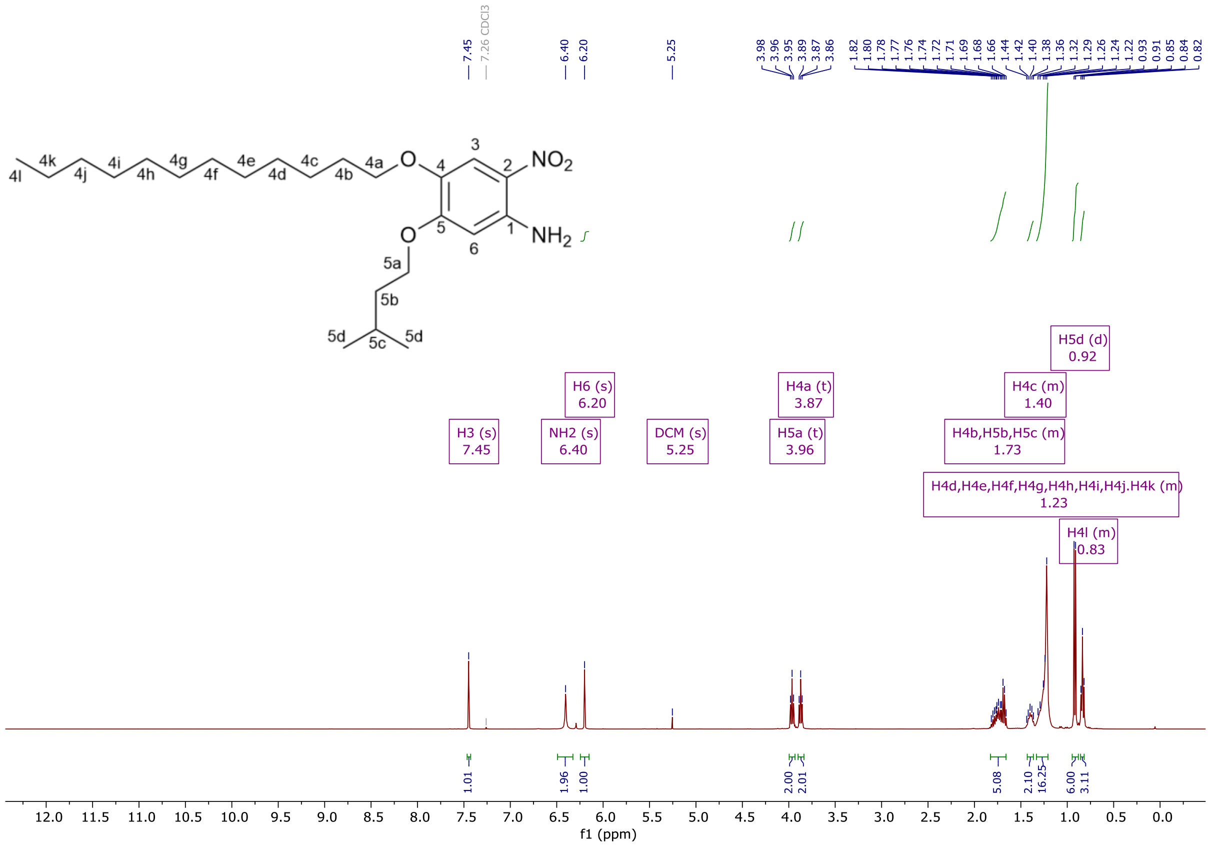

^{1}H NMR (400 MHz, Chloroform-*d*) spectrum of **6c**.

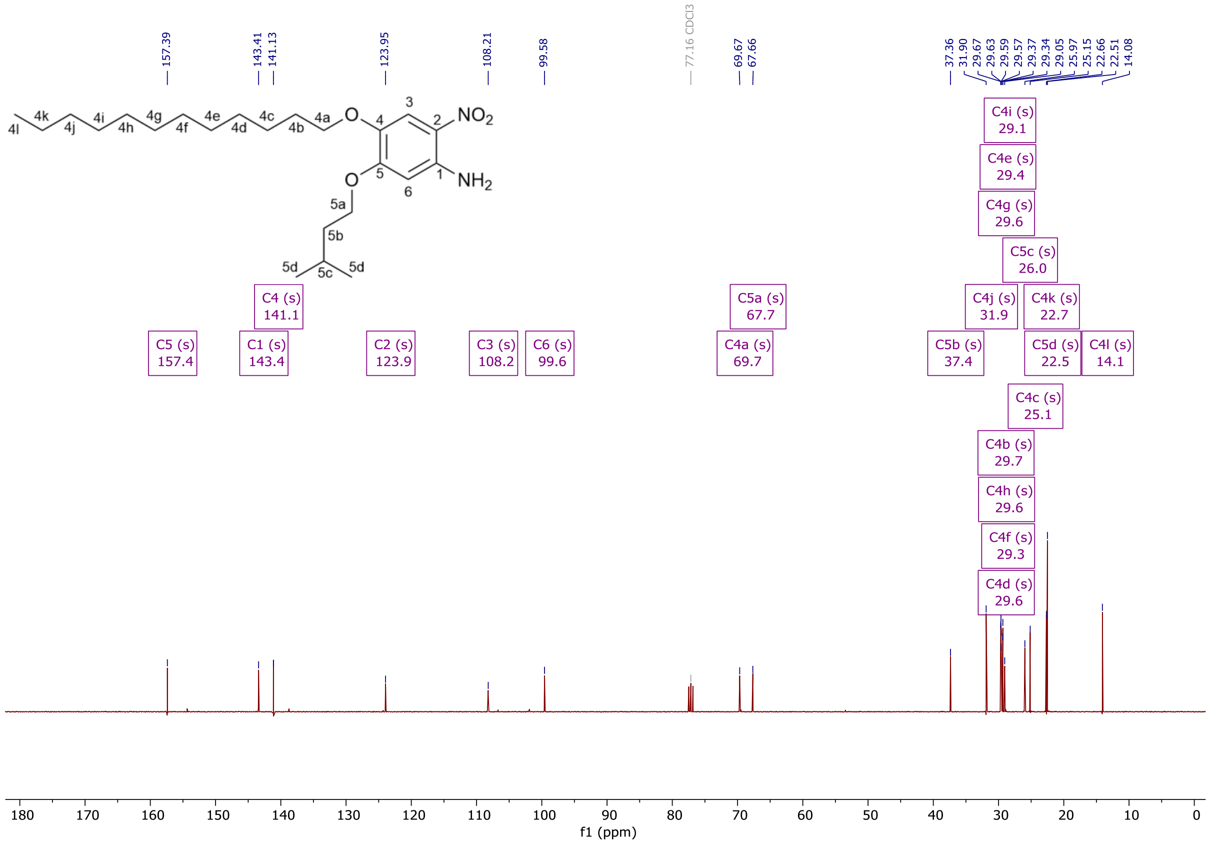

$^{13}C\{^{1}H\}$ NMR (101 MHz, Chloroform-*d*) spectrum of **6c**.

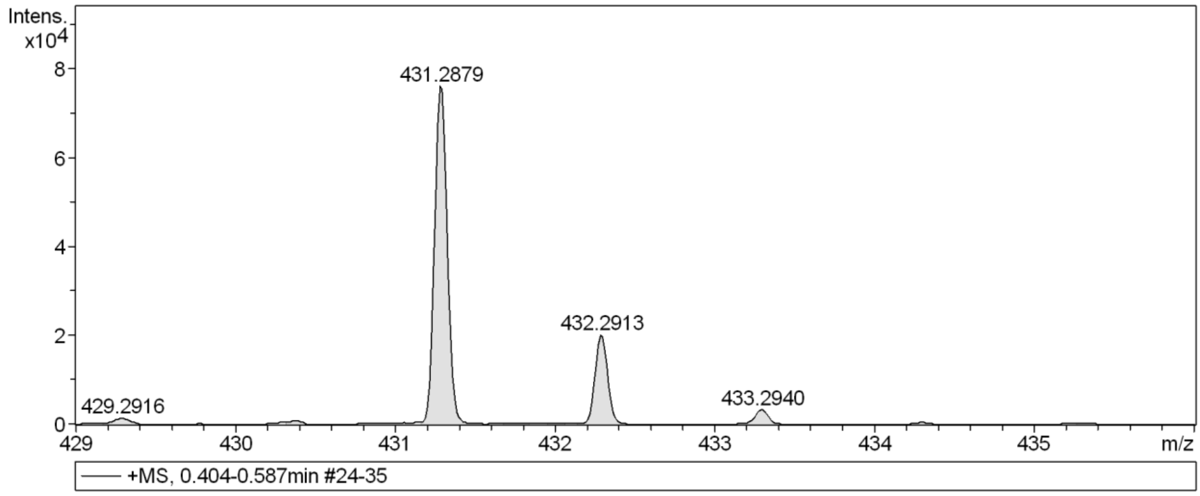

HRMS (ESI+) spectrum of **6c**.

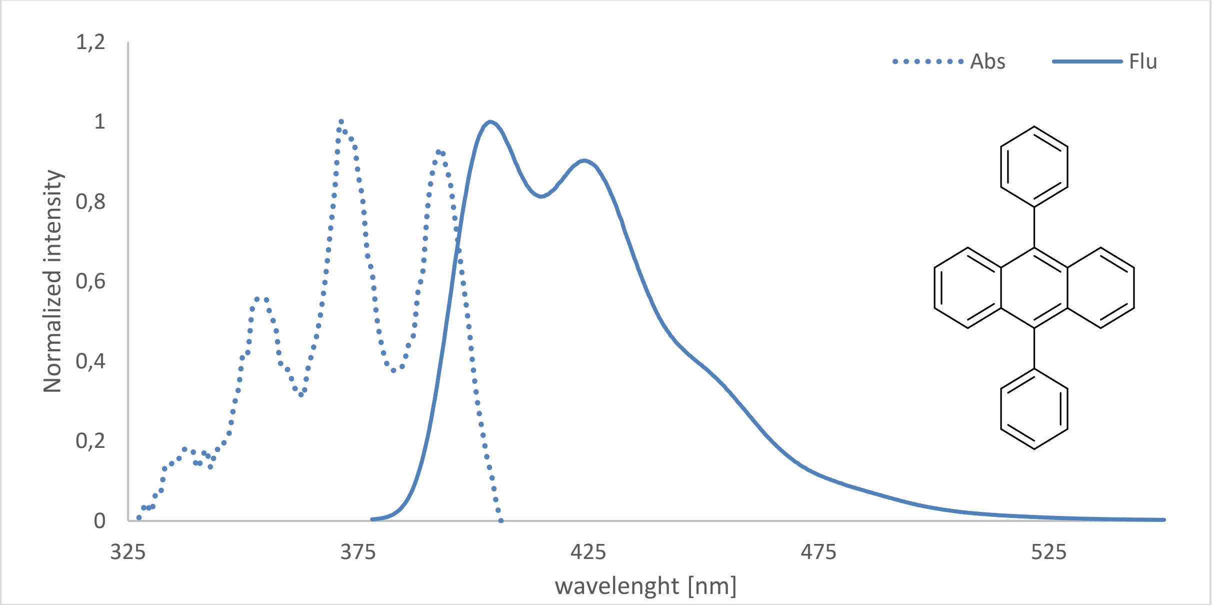

Absorbance and fluorescence (λ_{EX} = 370 nm) spectrum of 9,10-diphenylanthracene (DPA) in cyclohexane. The compound has Φ = 1.00 in cyclohexane.

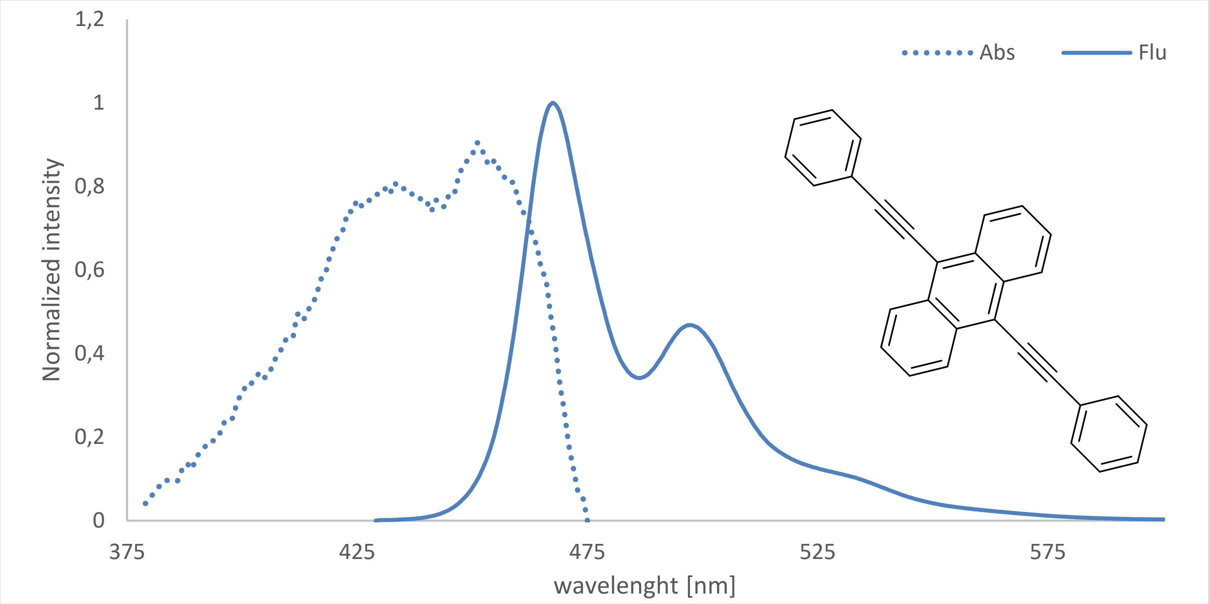

Absorbance and fluorescence (λ_{EX} = 430 nm) spectrum of 9,10-bis(phenylethynyl)anthracene (BPEA) in cyclohexane. The compound has Φ = 1.00 in cyclohexane.

References.

1. Wurth, C., Grabolle, M., Pauli, J., Spieles, M., et. al. Relative and absolute determination of fluorescence quantum yields of transparent samples. *Nat. Protoc.,* **2013**, 8, 1535-1550
2. Robards, K., Haddad, P.R., Jackson P.E., 5 - High-performance Liquid Chromatography—Instrumentation and Techniques. Principles and Practice of Modern Chromatographic Methods, **2004**, 227-303 (ISBN 9780080571782).
3. Taniguchi, M., Lindsey, J. S. Database of Absorption and Fluorescence Spectra of >300 Common Compounds for use in PhotochemCAD. *Photochem. Photobiol.* **2018**, *94*, 290–327.
4. Grolik, J., Ręka, P., Gorczyca, M. et al. Regioselective synthesis of the 4,5-dialkoxy-2-nitroanilines bearing two different alkoxy substituents. *Tetrahedron Lett.* **2022**, *99*, 153830−153836.
5. Ręka P., Grolik J., Stadnicka K. M. et al. Synthesis of Nonsymmetrically Substituted 2,3-Dialkoxyphenazine Derivatives and Preliminary Examination of Their Cytotoxicity. *J. Org. Chem.* **2023**, *88*, 1339−1351.
